# ﻿Discovery of a new tarantula species from the Madrean Sky Islands and the first documented instance of syntopy between two montane endemics (Araneae, Theraphosidae, *Aphonopelma*): a case of prior mistaken identity

**DOI:** 10.3897/zookeys.1210.125318

**Published:** 2024-08-16

**Authors:** Chris A. Hamilton, Brent E. Hendrixson, Karina Silvestre Bringas

**Affiliations:** 1 Department of Entomology, Plant Pathology & Nematology, University of Idaho, Moscow, ID 83844, USA University of Idaho Moscow United States of America; 2 Department of Biology, Millsaps College, Jackson, MS 39210, USA Millsaps College Jackson United States of America

**Keywords:** Biodiversity hotspot, Chiricahua Mountains, conservation, cryptic species, molecular systematics, phylogenomics, spider taxonomy

## Abstract

The Chiricahua Mountains in southeastern Arizona are renowned for their exceptional biodiversity and high levels of endemism. Morphological, genomic, behavioral, and distributional data were used to report the discovery of a remarkable new tarantula species from this range. *Aphonopelmajacobii***sp. nov.** inhabits high-elevation mixed conifer forests in these mountains, but also co-occurs and shares its breeding period with *A.chiricahua*—a related member of the *Marxi* species group—in mid-elevation Madrean evergreen oak and pine-oak woodlands. This marks the first documented case of syntopy between two montane endemics in the Madrean Archipelago and adds to our knowledge of this threatened region’s unmatched tarantula diversity in the United States. An emended diagnosis and redescription for *A.chiricahua* are also provided based on several newly acquired and accurately identified specimens. Phylogenetic analyses of mitochondrial and genomic-scale data reveal that *A.jacobii***sp. nov.** is more closely related to *A.marxi*, a species primarily distributed on the Colorado Plateau, than to *A.chiricahua* or the other Madrean Sky Island taxa. These data provide the evolutionary framework for better understanding the region’s complex biogeographic history (e.g., biotic assembly of the Chiricahua Mountains) and conservation of these spiders.

## ﻿Introduction

The mygalomorph spider genus *Aphonopelma* Pocock, 1901 is the most diverse group within the family Theraphosidae (tarantulas) and currently comprises 54 nominal species (World Spider Catalog 2024). The genus is widely distributed across two major biogeographic realms (i.e., the Nearctic and Neotropics) where it can be found throughout the southern third of the United States, ranging west of the Mississippi River to the Pacific Ocean, and south throughout Mexico and into Central America (but see Turner et al. 2018; Gabriel 2022). In the United States, these spiders are found across a wide range of physical and climatic conditions, from hot and arid desert valleys located near sea level to cool and mesic high-elevation forests (Hamilton et al. 2016).

Of particular interest are species occupying these high-elevation habitats, especially those found in the Madrean Archipelago (colloquially referred to as the Madrean “Sky Islands”, hereafter MSI), a series of isolated mountain ranges that span the cordilleran gap between the Colorado Plateau and Rocky Mountains of the southwestern United States and the Sierra Madre Occidental of northwestern Mexico. Prior to our work on *Aphonopelma* (see Hamilton et al. 2011, 2014, 2016; Hendrixson et al. 2013, 2015; Hendrixson 2019), knowledge concerning the diversity and distribution of tarantulas from this region was largely nonexistent. An early revision of the genus (Smith 1995) did not include samples from any of these mountain ranges, and the only mention of tarantulas occurring in the Madrean Archipelago was limited to two unpublished master’s theses (Beatty 1961; Jung 1975).

Hendrixson et al. (2015) first investigated the diversity of MSI tarantulas based on samples collected from five mountain ranges in southeastern Arizona and southwestern New Mexico. The authors concluded there were three undescribed species that formed a clade: one endemic to the Santa Catalina Mountains, one endemic to the Peloncillo Mountains and surrounding grasslands, and one found more broadly in the Huachuca, Pajarito, and Santa Rita Mountains. Shortly thereafter, a newly collected male specimen from the Chiricahua Mountains turned out to represent yet another distinct species belonging to the group. Hamilton et al. (2016) subsequently described and named these four species *Aphonopelmacatalina* Hamilton, Hendrixson & Bond, 2016, *A.peloncillo* Hamilton, Hendrixson & Bond, 2016, *A.madera* Hamilton, Hendrixson & Bond, 2016, and *A.chiricahua* Hamilton, Hendrixson & Bond, 2016, respectively, and placed them into the *Marxi* species group. Based on these patterns of diversity (i.e., high levels of MSI endemicity), Hendrixson et al. (2015) and Hamilton et al. (2016) hypothesized that the MSI should harbor many more undescribed species, especially in mountain ranges that have been poorly sampled or not sampled at all. Hendrixson (2019) confirmed this prediction with the discovery and description of *A.bacadehuachi* Hendrixson, 2019 from nearby pine-oak woodlands in northeastern Sonora, Mexico.

**Figure 1.**
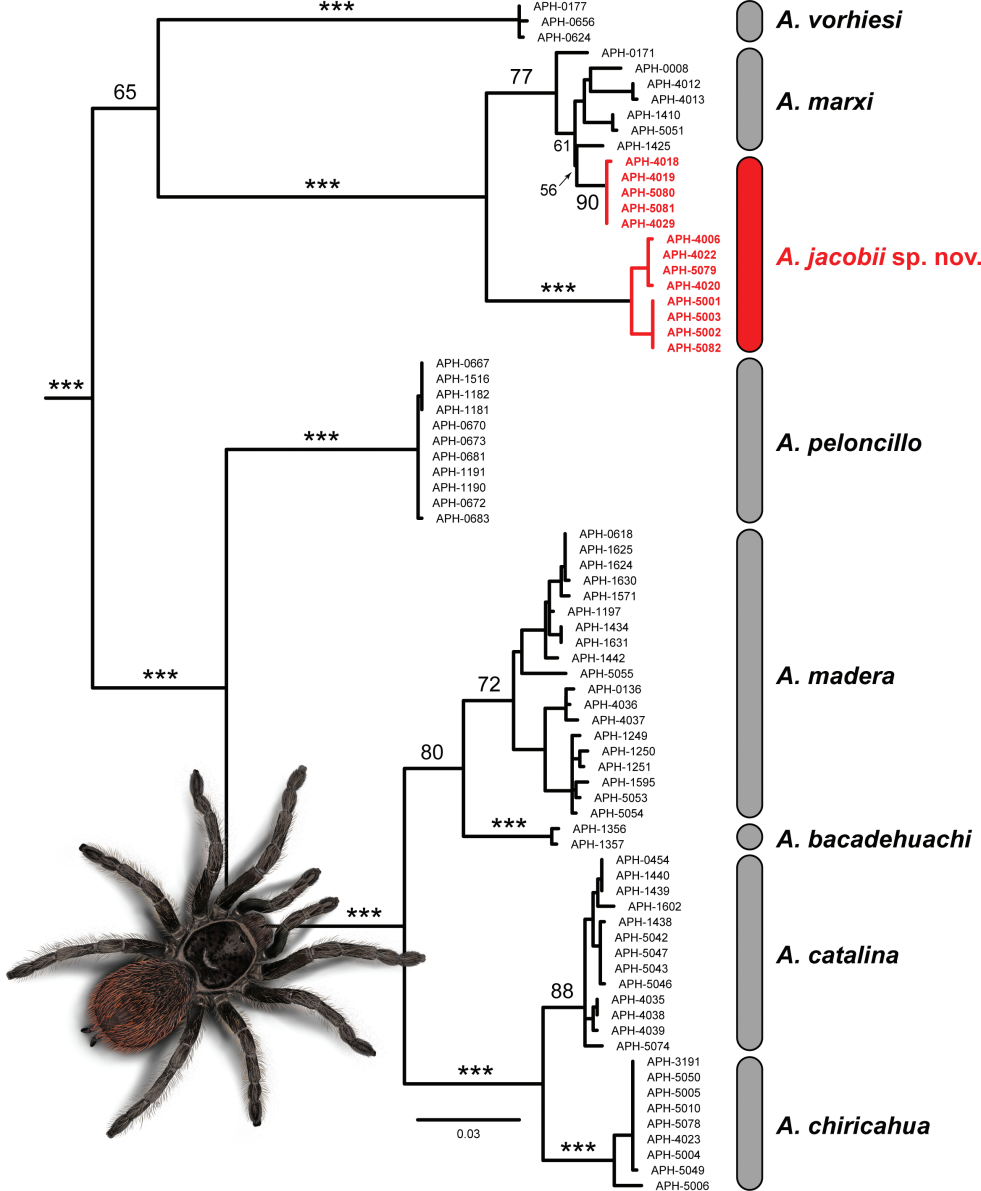
Maximum likelihood phylogeny of the *Marxi* species group based on the mitochondrial gene cytochrome oxidase c subunit I (COX1). The red clades highlight the diphyly of *Aphonopelmajacobii* sp. nov. Bootstrap node support values are indicated along branches of interest (*** indicates branches supported with values ≥ 95).

During a field trip to the Chiricahua Mountains in late October 2018, a series of tarantulas was observed in high-elevation (c. 2364 m) mixed conifer forest. These individuals were tentatively assigned to *A.chiricahua* based on their location (see Jacobi 2019), but preliminary phylogenetic analyses of DNA sequences gathered from the mitochondrial gene cytochrome *c* oxidase subunit I (hereafter COX1) suggested they were distinctly different from that species (i.e., these individuals were genetically divergent from *A.chiricahua* and were not recovered in the same clade; Fig. 1). The following year in late October, we (CAH, BEH) revisited the Chiricahua Mountains and successfully found more individuals from two additional populations. After analyzing genomic-scale data using Ultraconserved Elements (hereafter UCE) and reexamining the morphology of these spiders, we have confirmed that these tarantulas are indeed distinct from *A.chiricahua* and belong to a new species.

The purpose of this study is two-fold: (1) to report the discovery of this new *Marxi* group species and to document the first case of multiple short-range endemic tarantula species inhabiting a single MSI range; and (2) to redescribe *A.chiricahua* based on newly acquired specimens because we have determined that all the material examined in the original description of *A.chiricahua* was misidentified (i.e., actually *A.jacobii* sp. nov.) except for the male holotype (see Hamilton et al. 2016).

## ﻿Materials and methods

### ﻿Museum repositories and comparative material examined

All voucher specimens examined as part of this study are deposited in the American Museum of Natural History, New York, New York (**AMNH**) and the University of Idaho William F. Barr Entomological Museum, Moscow, Idaho (**UIM**). Collection data for all comparative material examined as part of this study are provided in the Suppl. materials 1–3, 9, 10 and Hamilton et al. (2016). For newly examined samples, the accuracy (precision score) of each set of GPS coordinates (reported in decimal degrees) is annotated as a superscript in the material examined sections for each species using the scheme described in Hamilton et al. (2016), as modified from Murphey et al. (2004) and Bond (2012).

### ﻿Measurements, characterization, and illustration of morphological features

All material was preserved in 80% ethanol and assigned a unique alphanumeric voucher number (APH-####) that can be used to cross-reference all images, measurements, and locality data. Abbreviations for all quantitative morphological characters follow Hamilton et al. (2016). Measurements are reported in millimeters and were made with a Leica M125C stereomicroscope using the Leica Application Suite software and a digital camera, or from a Mitutoyo 500-196-30 Advanced Onsite Sensor (AOS) Absolute Scale Digital Caliper. Unless otherwise stated, measurements were taken from left appendages. Lengths of leg segments were taken from the mid-proximal point of articulation to a distal point of the article, as detailed and shown in Hamilton et al. (2016). Quantitative measurements used for diagnosing and describing *Aphonopelmajacobii* sp. nov. were obtained from 14 mature male and 6 mature female specimens, whereas measurements used for re-diagnosing and redescribing *A.chiricahua* were gathered from six mature male and two mature female specimens.

Digital images for the morphological plates (Figs 4, 5, 9, Suppl. materials 6, 7, 11) were made using a Visionary Digital Imaging System (Visionary Digital^TM^, Richmond, VA) where images were recorded at multiple focal planes and then assembled into a single focused image using the computer program Zerene Stacker v. 1.04 (Zerene Systems LLC, Richland, WA). The female genital region was removed from the abdominal wall and the tissues were dissolved using trypsin (incubated overnight at 37 °C in a 1.5-ml microcentrifuge tube); spermathecae were examined and photographed in the manner described above. All images were cropped and toned in Adobe Photoshop (Adobe Systems, Inc.). All morphological measurements and high-resolution photographs are available in the Suppl. materials 9, 11.

### ﻿Evaluation of quantitative morphological features for species diagnoses

Measurements and morphometric ratios that were determined to have non-overlapping ranges between members of the *Marxi* species group were used for establishing morphological diagnoses. Measurements for all previously described members of the *Marxi* species group were obtained from Hamilton et al. (2016) and Hendrixson (2019) and added to a dataset containing the newly generated measurements for *A.jacobii* sp. nov. and *A.chiricahua* (see Suppl. materials 5, 9). As in Hamilton et al. (2016), we investigated 153 ratio combinations for mature males and 135 for mature females. For all measurements and morphometric ratios, we acknowledge that by including additional specimens in the future, these boundaries could change. We have attempted to limit these effects by including specimens that span the breadth of size variation across each species’ distribution.

**Figure 2.**
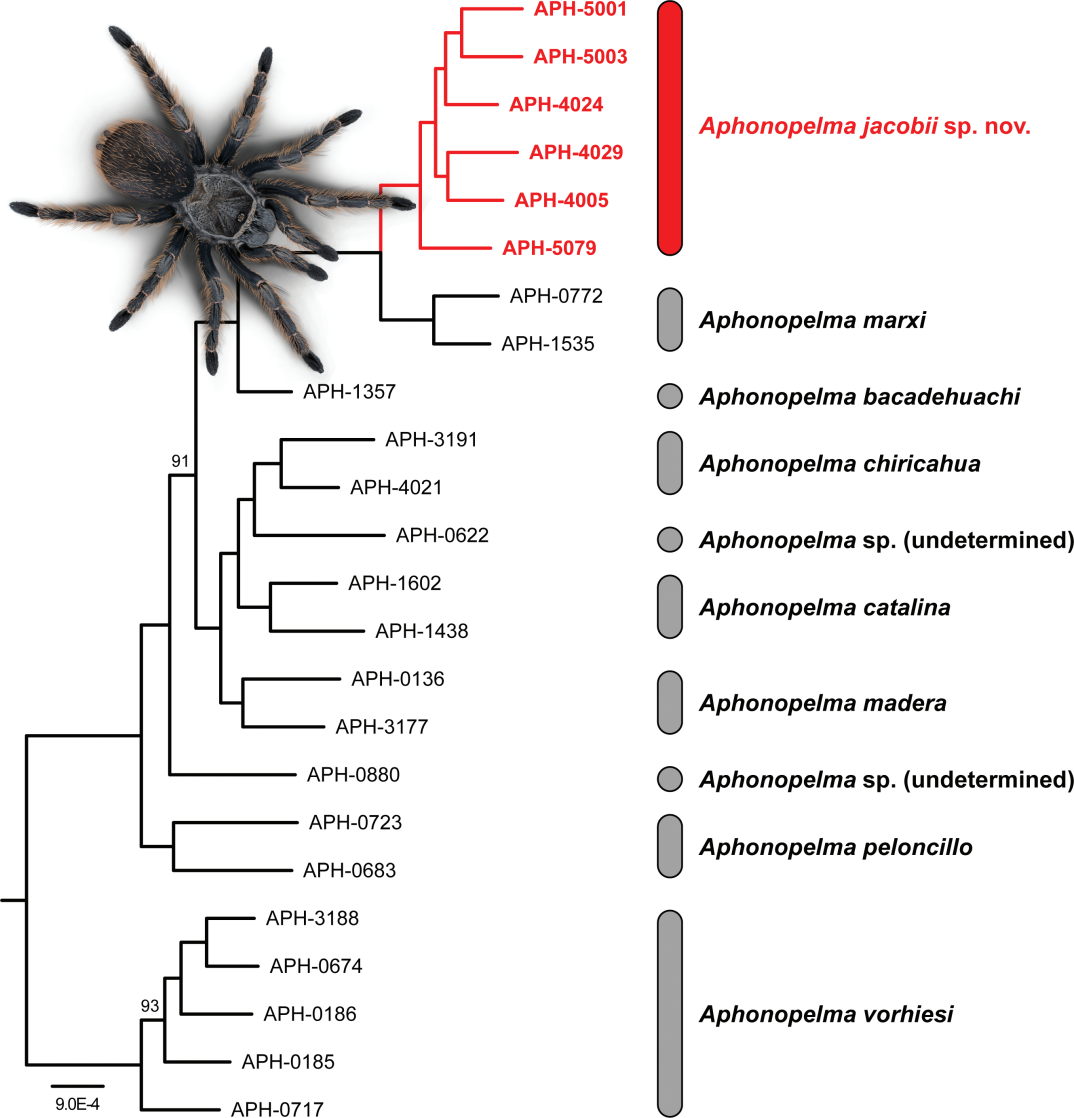
Maximum likelihood phylogeny of the *Marxi* species group based on Ultraconserved Element phylogenomics (UCE). The red clade highlights the monophyly/exclusivity of *Aphonopelmajacobii* sp. nov. Except where noted, all nodes are supported by bootstrap support values ≥ 95. All samples included in this tree, except *A.jacobii* sp. nov. and *A.bacadehuachi*, are the same samples used in the phylogeny from the US *Aphonopelma* revision (Hamilton et al. 2016).

For recently collected specimens of *A.jacobii* sp. nov. and *A.chiricahua*, species assignments were determined based on the results of the phylogenetic analyses (see Figs 1, 2) and verified with morphometrics. Specimens that grouped (without overlapping other species) with APH-3191—the male holotype of *A.chiricahua*—were assigned to that species, whereas specimens that did not group with APH-3191 were assigned to *A.jacobii* sp. nov. For older museum specimens previously identified as *A.chiricahua* (see Hamilton et al. 2016) that did not include genetic data, species assignments for these individuals were determined by performing a discriminant analysis in the statistical software program JMP ver. 17.2.0 (https://www.jmp.com). Separate models for males and females were trained using raw measurements from specimens whose species identities had been confirmed phylogenetically. These models were then used to classify (i.e., identify) the museum specimens as *A.jacobii* sp. nov. or *A.chiricahua*. To explore the extent of morphological variation found within and between species of the *Marxi* species group, we visually examined traditional PCA (principal component analysis) morphospace (see Suppl. material 4) and boxplots of morphometric ratios (see Suppl. material 5) using the R computing environment (https://www.r-project.org). Two-dimensional PCA morphospace was evaluated by plotting PC1 and PC2.

### ﻿Molecular techniques, taxon sampling, and phylogenetic analyses

Legs were removed from all freshly collected material (generally from the R side) and preserved in ≥ 95% ethanol or RNA*later*^TM^ (Qiagen, Valencia, CA, USA) and stored at -80 °C. Genomic DNA was extracted from muscle tissues using the Qiagen DNeasy Tissue Kit^TM^ (Qiagen, Valencia, CA, USA) or Omniprep^TM^ (G-Biosciences) and then qualitatively and quantitatively assessed using agarose gel electrophoresis and a Qubit 2.0 Fluorometer (Thermo Fisher Scientific, Waltham, MA), respectively.

All laboratory procedures for generating COX1 sequence data are described in Hendrixson et al. (2013). The dataset included 77 samples representing all described members of the *Marxi* species group. We obtained sequences from GenBank for the following outgroup taxa: *Ornithoctonushuwena* (= *Cyriopagopusschmidti*) (AY309259) and *Acanthoscurria* sp. (JX946011). Protocols for analyzing the COX1 dataset essentially follow Hendrixson (2019). Alignment of the dataset was straightforward, and sequences were translated and checked for unexpected stop codons in Mesquite v. 3.81 (Maddison and Maddison 2023). We performed a maximum likelihood (ML) phylogenetic analysis in IQ-TREE (Nguyen et al. 2015) through the W-IQ-TREE web server (Trifinopoulos et al. 2016) available at http://iqtree.cibiv.univie.ac.at. The substitution model was set to “Auto” which allows IQ-TREE to determine the best-fit substitution model for the data using ModelFinder (Kalyaanamoorthy et al. 2017); the FreeRate heterogeneity (+R) option was also selected. Clade support was estimated using 1,000 ultrafast bootstrap replicates (Hoang et al. 2018).

The UCE dataset included 24 samples representing all described members of the *Marxi* species group, including two individuals whose species assignment has not yet been determined due to inadequate sampling from their respective areas (APH-0622, APH-0880). Except for *A.bacadehuachi*, each species was represented by at least two individuals—the same individuals used in the Anchored Hybrid Enrichment (AHE) phylogenomic tree from our previous revisionary work (Hamilton et al. 2016). We included six samples of *A.jacobii* sp. nov. covering its entire distribution in the Chiricahuas due to the curious placement of the Barfoot Park population in the COX1 phylogeny (see the Results and Discussion sections below). *Aphonopelmavorhiesi* (Chamberlin & Ivie, 1939) was used as an outgroup to root the phylogeny because our preliminary UCE phylogenomic analyses of the entire *Aphonopelma* genus consistently placed this species as sister to all other species in the *Marxi* species group with strong support (100 bs, unpublished).

To construct the UCE phylogenomic dataset, extracted DNA was sent to Rapid Genomics (Gainesville, FL) for library preparation, UCE hybridization, and high-throughput sequencing. Library preparation was performed for Illumina sequencing utilizing their high-throughput workflow with proprietary chemistry. DNA was sheared to a mean fragment length of ~ 500 bp, fragments were end-repaired and A-tailed, followed by incorporation of unique dual-indexed Illumina adaptors and PCR enrichment. Samples were pooled equimolar and sequenced on a SP flow cell (2 × 250 bp) or a S4 flow cell (2 × 150 bp). Data was assembled with SPAdes (Prjibelski et. al. 2020) and processed using Phyluce v. 1.7.1 (Faircloth 2016) and a combined arachnid-spider hybrid probe set (Starrett et al. 2017; Kulkarni et al. 2020), where match settings for minimum identity and minimum coverage of 95 and 95 (respectively) were used to create a dataset of 1311 loci. These loci were aligned using MUSCLE (Edgar 2022) then internally trimmed using GBlocks with b1, b2, b3, and b4, settings of 0.5, 0.6, 10, and 5, respectively, to remove poorly aligned blocks within the sequences. The data were then additionally cleaned using AMAS ver. 1.02 (Borowiec 2016) and Spruceup v. 2022.2.4 (Borowiec 2019), which removes poorly aligned sequence fragments from individual sequences within alignments. Visual examination of distance distribution plots identified a setting of 0.9 as optimal for trimming, with a 90% occupancy matrix then being generated for use in subsequent analyses. A maximum likelihood-based phylogeny was inferred using IQTree2 (Nguyen et al. 2015; Minh et al. 2020), with the ultrafast bootstrap flag and 1000 replicates for node support values. All analyses were performed on the University of Idaho Research Computing and Data Services (RCDS) high-performance computing cluster.

DNA sequence alignments, phylogenetic trees, and scripts have been deposited in the Suppl. material 8. A list of GenBank accession codes for all COX1 samples and the SRA BioProject number for all raw sequencing data used to generate the UCE loci in this study are provided in the Suppl. material 3.

### ﻿Species concept

The species concept we employ follows the Unified Species Concept discussed by de Queiroz (2005). Where possible, we employ a combination of morphological, genomic, behavioral, and distributional evidence to identify independently evolving lineages.

### ﻿*Quantitative morphological landmarks* (Hamilton et al. 2016: fig. 3)

**Cl** length of the carapace

**Cw** width of the carapace

**LBl** labial length

**LBw** labial width

**F1** femur I length (retrolateral aspect)

**F1w** femur I width

**P1** patella I length

**T1** tibia I length

**M1** metatarsus I length

**A1** tarsus I length

**F3** femur III length (prolateral aspect)

**F3w** femur III width

**P3** patella III length

**T3** tibia III length

**M3** metatarsus III length

**A3** tarsus III length

**F4** femur IV length (prolateral aspect)

**F4w** femur IV width

**P4** patella IV length

**T4** tibia IV length

**M4** metatarsus IV length

**A4** tarsus IV length

**PTl** palpal tibia length (retrolateral aspect)

**PTw** palpal tibia width

**SC3** ratio of the extent of metatarsus III scopulation (length of scopulation/ventral length of metatarsus III)

**SC4** ratio of the extent of metatarsus IV scopulation (length of scopulation/ventral length of metatarsus IV)

### ﻿Data resources

All specimens examined as part of this study are deposited in the William F. Barr Entomological Museum in the Department of Entomology, Plant Pathology and Nematology at the University of Idaho in Moscow, Idaho (**UIM**), and the American Museum of Natural History in New York City, New York (**AMNH**). All prior specimens deposited in the Auburn University Museum of Natural History, Auburn, Alabama (**AUMNH**) have been transferred to UIM. All data (molecular, morphological, geographic, and images) used to establish these species hypotheses can be found in the Suppl. materials. All UCE raw reads can be found on the Sequence Read Archive (SRA) (BioProject ID: PRJNA1099687). All specimen data can be found in the Suppl. materials. High resolution morphological images (Suppl. material 11) are stored on FigShare (doi: 10.6084/m9.figshare.26133724). The data underpinning the analysis reported in this paper are deposited at GBIF, the Global Biodiversity Information Facility, and are available at https://doi.org/10.15468/9sf6jf.

## ﻿Results

### ﻿Discriminant analysis, morphometrics, and PCA

The discriminant analysis performed on both males and females accurately classified every individual of *A.jacobii* sp. nov. and *A.chiricahua* that was used to train the models. These models then classified each “unknown” museum specimen as *A.jacobii* sp. nov. with very high probability (≥ 0.99). Consequently, every older museum specimen previously identified as *A.chiricahua* in Hamilton et al. (2016) (i.e., APH-2097, APH-2101, APH-2102, APH-2105, APH-2480-A, APH-2480-B, and APH-2548) has been transferred to *A.jacobii* sp. nov.

Results of the PCA and comparative morphometric ratios (i.e., boxplots) can be viewed in the Suppl. materials 4, 5. Males of *A.chiricahua* (smaller individuals), *A.jacobii* sp. nov., *A.madera*, and *A.Marxi* separate from most other members of the *Marxi* species group along PC1. And while there is some overlap in PCA morphospace between various combinations of these taxa, it appears there is slight separation between *A.jacobii* sp. nov. and *A.marxi* (which are sister taxa, see below), and between *A.jacobii* sp. nov. and *A.chiricahua* (S4A). Females of *A.jacobii* sp. nov. clearly separate from all other members of the *Marxi* species group along PC1 (S4B). Additionally, there are a number of measurement ratios that can be used to separate species (i.e., they do not overlap) – some of these measurement ratios are used in the species diagnoses (below).

### ﻿Phylogenetic and phylogenomic analyses

The aligned COX1 dataset comprised 906 sites (230 parsimony informative). ModelFinder determined that the best-fit model for the data (based on the Bayesian Information Criterion) was TIM2+F+I+G4. The ML tree topology (log-likelihood score = –4360.8489) (Fig. 1) shows strong support for a clade (97 bs) comprising the five previously described Madrean species (*A.bacadehuachi*, *A.catalina*, *A.chiricahua*, *A.madera*, and *A.peloncillo*). This group is sister to a weakly supported clade (65 bs) comprising *A.vorhiesi* and *A.marxi* + *A.jacobii* sp. nov. COX1 haplotypes for *A.marxi* and *A.jacobii* sp. nov. form a very strongly supported clade (100 bs), as do the haplotypes for *A.catalina* and *A.chiricahua* (99 bs). The most notable results are that (1) *A.chiricahua* and *A.jacobii* sp. nov. do not form a clade and are not the most closely related lineages to each other; and (2) COX1 haplotypes for *A.jacobii* sp. nov. are diphyletic (see red branches in Fig. 1), resulting in *A.marxi* paraphyly (i.e., *A.marxi* sample APH-1425 is sister to *A.jacobii* sp. nov. samples from the Barfoot Park locality). Individuals of *A.jacobii* sp. nov. from Cave Creek Canyon (APH-4006, APH-4020, APH-4022, APH-5079) and the type locality near Onion Saddle (APH-5001, APH-5002, APH-5003, APH-5082) form a very strongly supported clade (100 bs); each population is reciprocally monophyletic with strong support (94 bs and 99 bs, respectively). Individuals of *A.jacobii* sp. nov. from the high-elevation Barfoot Park locality (APH-4018, APH-4019, APH-4029, APH-5080, APH-5081) form a clade (90 bs) nested within *A.marxi*. Support for this internal nesting structure is moderately weak, however (56 bs, 61 bs, 77 bs, respectively, starting at the node that groups the Barfoot Park population with *A.marxi* (APH-1425)).

The Ultraconserved Elements (UCE) phylogeny was inferred from 1311 loci across 24 samples using a combined spider-arachnid probe set originally merged in Maddison et al. (2020). The total length of the concatenated alignment was 1,870,552 bp and mean loci length was 1331 bp (minimum and maximum length of loci were 207 and 2438 bp, respectively). ModelFinder determined the best-fit model for each partition (i.e., UCE locus). The UCE tree topology (Fig. 2) has very strong support (100 bs) throughout; only two nodes received bootstrap values below 95. Fig. 2 identifies strong support (100 bs) for a clade that includes six previously described Madrean species (*A.marxi*, *A.bacadehuachi*, *A.chiricahua*, *A.catalina*, *A.madera*, *A.peloncillo*), two undetermined species, and *A.jacobii* sp. nov. Within the UCE phylogeny, *A.jacobii* sp. nov. is sister to *A.marxi*, with *A.bacadehuachi* sister to those lineages. *Aphonopelmachiricahua* is sister to APH-0622 and *A.catalina*, with *A.madera* being sister to those lineages. Another undetermined species (APH-0880) is the sister lineage to these lineages, and *A.peloncillo* is sister to all other members of the *Marxi* species group except *A.vorhiesi*. Importantly, the UCE tree depicts *A.jacobii* sp. nov. as phylogenetically distant from its syntopic congener, *A.chiricahua*.

The primary differences between the UCE and COX1 phylogenies are the interrelationships between various clades and species (e.g., placement of *A.bacadehuachi* and *A.vorhiesi*) and whether *A.jacobii* sp. nov. and *A.marxi* are reciprocally monophyletic (Fig. 2) or not (Fig. 1). These phylogenies share two important similarities: (1) *A.marxi* and *A.jacobii* sp. nov. form a strongly supported clade (100 bs); and (2) *A.chiricahua* and *A.jacobii* sp. nov. do not form a clade and are not closely related to each other. Unlike COX1, individuals of *A.jacobii* sp. nov. from the high-elevation Barfoot Park (APH-4005, APH-4029) and Onion Saddle (APH-4024, APH-5001, APH-5003) populations in mixed conifer forest are reciprocally monophyletic (95 bs and 99 bs, respectively) and form a clade (95 bs) that is sister to the APH-5079 that was included from a lower-elevation site in Cave Creek Canyon.

### ﻿Taxonomy

**Family Theraphosidae Thorell, 1869**

**Subfamily Theraphosinae Thorell, 1870**

#### ﻿Genus Aphonopelma

Taxon classificationAnimaliaAraneaeTheraphosidae

Pocock, 1901

61410173-E9BC-5E7C-A68E-DDC18A668919

Aphonopelma Pocock, 1901: 553 (type species by original designation Eurypelmaseemanni Pickard-Cambridge, 1897). First synonymized with Rhechostica by Raven (1985: 149).
Rhechostica Simon, 1892: 162 (type species by original designation Homoeommatexense Simon, 1891). Suppressed as a senior synonym of Aphonopelma by ICZN (1991: 166–167).
Delopelma Petrunkevitch, 1939: 567 (type species by original designation Eurypelmamarxi Simon, 1891) (considered a subgenus of Aphonopelma by Chamberlin, 1940: 26). First synonymized with Rhechostica by Raven (1985: 151).
Gosipelma Chamberlin, 1940: 4 (type species by original designation Gosipelmaangusi Chamberlin, 1940). Originally described as a subgenus of Aphonopelma, but never elevated to full generic status. First synonymized with Rhechostica by Raven (1985: 153).
Chaunopelma Chamberlin, 1940: 30 (type species by original designation Delopelmaradinum Chamberlin & Ivie, 1939). First synonymized with Rhechostica by Raven (1985: 151).
Apachepelma Smith, 1995: 45 (type species by original designation Aphonopelmapaloma Prentice, 1992). First synonymized with Aphonopelma by Prentice (1997: 147).

#### ﻿*Marxi* species group (informally designated by Hamilton et al. 2016)

*Aphonopelmabacadehuachi* Hendrixson, 2019

*Aphonopelmacatalina* Hamilton, Hendrixson & Bond, 2016

*Aphonopelmachiricahua* Hamilton, Hendrixson & Bond, 2016

*Aphonopelmajacobii* Hamilton & Hendrixson, 2024, sp. nov.

*Aphonopelmamadera* Hamilton, Hendrixson & Bond, 2016

*Aphonopelmamarxi* (Simon, 1891)

*Aphonopelmapeloncillo* Hamilton, Hendrixson & Bond, 2016

*Aphonopelmavorhiesi* (Chamberlin & Ivie, 1939)

#### ﻿ Aphonopelma jacobii

Taxon classificationAnimaliaAraneaeTheraphosidae

Hamilton & Hendrixson, 2024 sp. nov.

4BC5A9E2-D5FF-5C18-9675-2225F75A4240

https://zoobank.org/28ABD35B-FB05-4ADF-ABF0-622ED48DAC71

**Figure 3.**
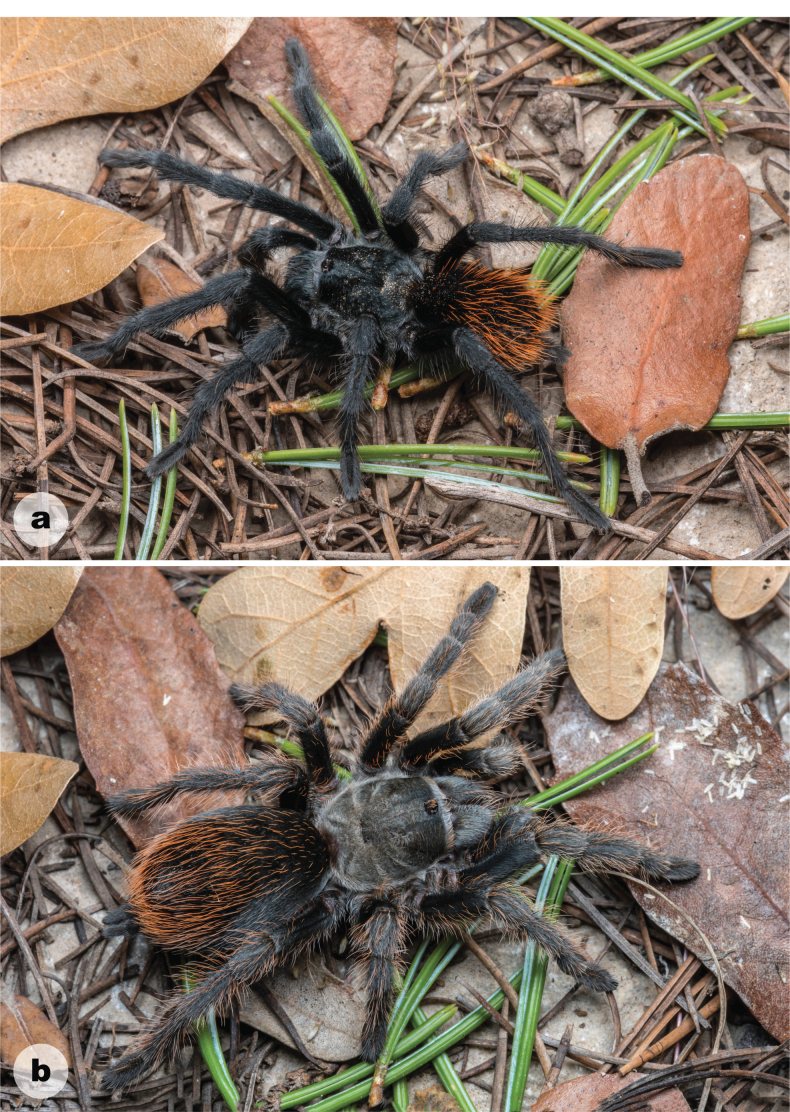
Live habitus of *Aphonopelmajacobii* sp. nov. **a** male holotype (APH-5002) **b** female paratype (APH-5001).

**Figure 4.**
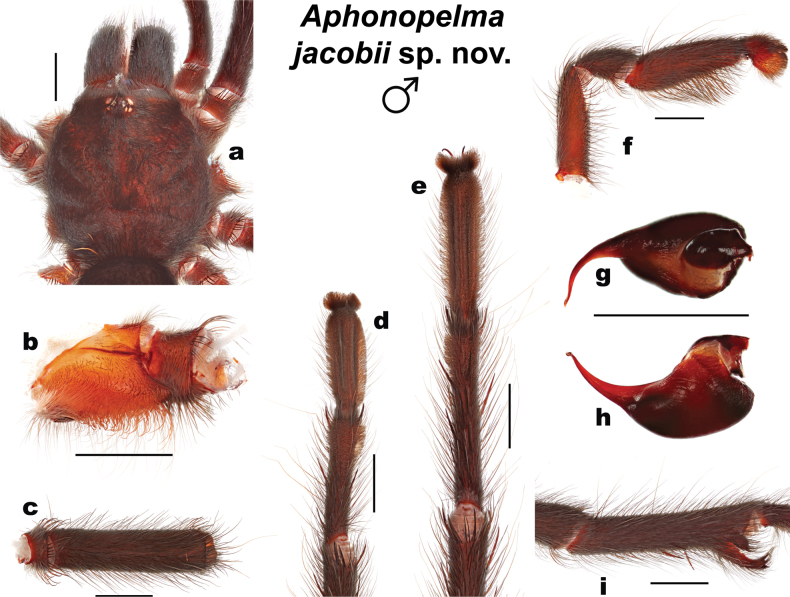
Morphology of *Aphonopelmajacobii* sp. nov. (male holotype, APH-5002) **a** carapace, dorsal view **b** coxa of leg I, prolateral view **c** femur of leg III, dorsal view **d** metatarsus and tarsus of leg III, ventral view **e** metatarsus and tarsus of leg IV, ventral view **f** pedipalp, prolateral view **g** palpal bulb, dorsal view **h** palpal bulb, retrolateral view **i** tibia of leg I showing mating clasper, prolateral view. Scale bars: 2 mm.

**Figure 5.**
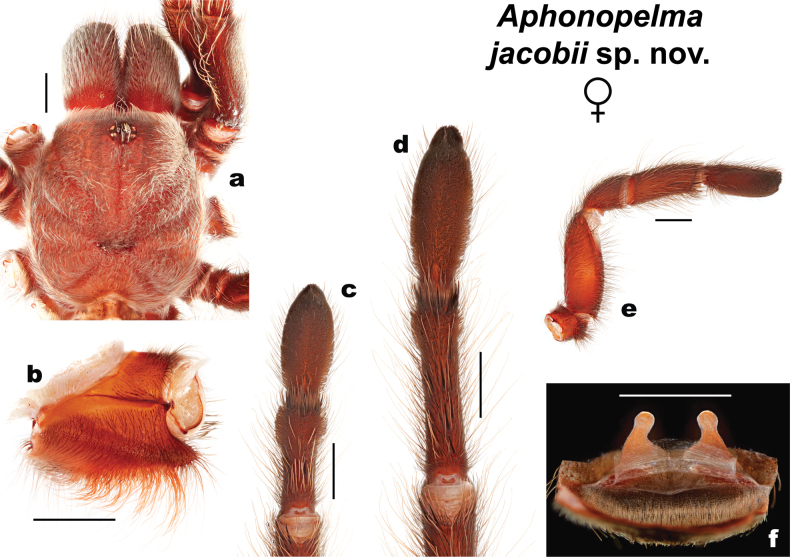
Morphology of *Aphonopelmajacobii* sp. nov. (female paratype, APH-5001) **a** carapace, dorsal view **b** coxa of leg I, prolateral view **c** metatarsus and tarsus of leg III, ventral view **d** metatarsus and tarsus of leg IV, ventral view **e** pedipalp, prolateral view **f** spermathecae. Scale bars: 2 mm.

**Figure 6.**
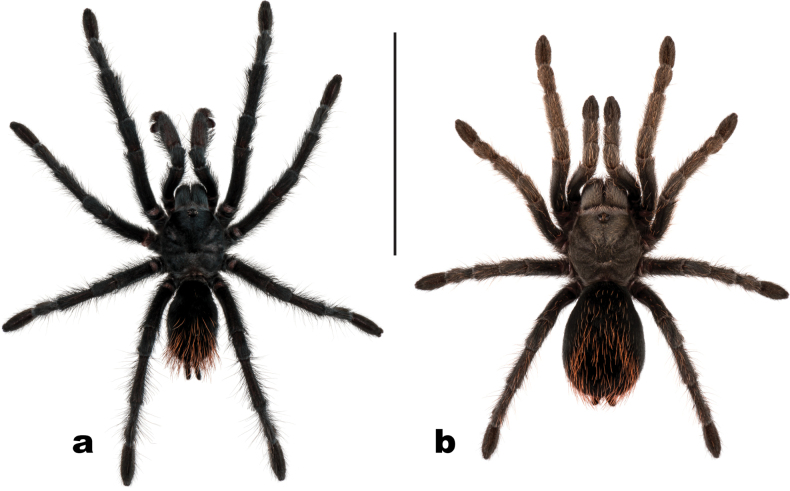
Dorsal habitus of *Aphonopelmajacobii* sp. nov. **a** male (APH-4022) **b** female (APH-4018). Scale bar: 25 mm.

**Figure 7.**
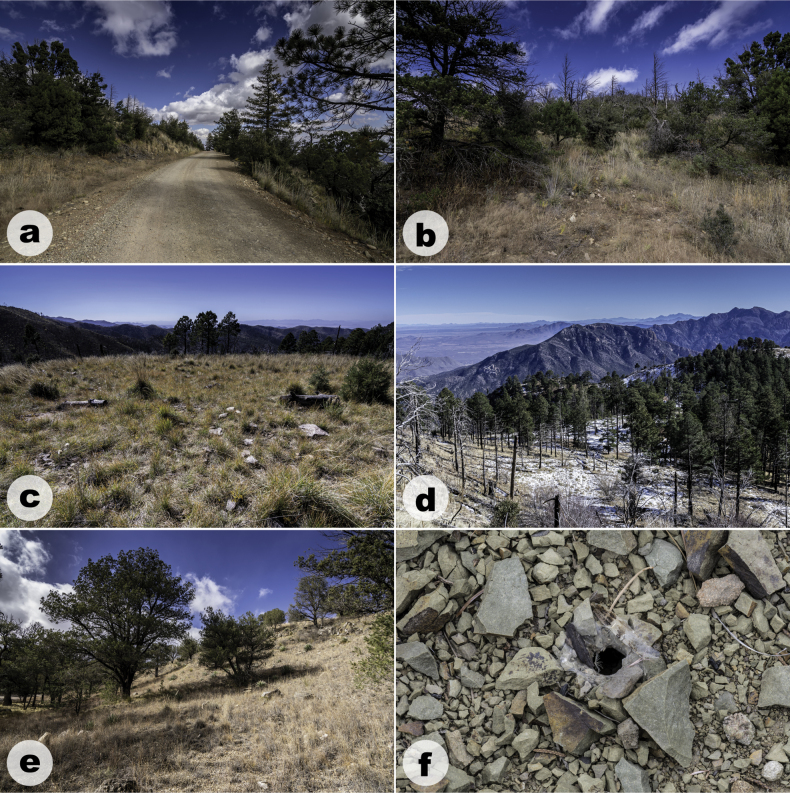
Habitat images of *Aphonopelmajacobii* sp. nov. from the Chiricahua Mountains, Cochise County, Arizona **a, b** type locality along Forest Road 42D above Onion Saddle **c, d** Barfoot Park **e** along Forest Road 42A **f** open burrow at the type locality. Photographs of images **d** and **e** provided by Michael A. Jacobi.

Figs 3 , 4 , 5 , 6 , 7 , 11

Aphonopelma chiricahua , in part: Hamilton et al. 2016: 90, 91, 93–95, fig. 38 (APH-2097, misidentification); 95 (APH-2101, APH-2102, APH-2105, APH-2480-A, APH-2480-B, APH-2548, misidentifications).

##### Type material.

***Holotype*.** United States • ♂; Arizona, Cochise County, Chiricahua Mountains, along Forest Road 42D above Onion Saddle; 31.92838°N, 109.26311°W^1^; 2364 m; 31 Oct. 2018; Brent E. Hendrixson & Michael A. Jacobi leg.; UIM; APH-5002.

***Paratype*.** United States • 1 ♀; same data as for holotype; UIM; APH-5001 • 1 ♂; same data as for holotype; AMNH; APH-5003.

##### Etymology.

The specific epithet is a patronym in honor of our friend, Michael A. Jacobi, who facilitated many of our field trips into the Chiricahua Mountains in 2018 and 2019. In addition, he generously carried out field work in the MSI on our behalf and discovered many important specimens, including the first female burrows of *A.chiricahua* and this remarkable new species. His tireless work in the field and passion for natural history have immensely helped improve our knowledge of tarantula biology and biodiversity in the Chiricahua Mountains and surrounding areas.

##### Diagnosis.

*Aphonopelmajacobii* sp. nov. is a member of the *Marxi* species group and can be distinguished by a combination of morphological, genomic, behavioral, and distributional features. This species is a mid- to late-fall breeder endemic to the Chiricahua Mountains in southeastern Arizona. Nuclear DNA identifies *A.jacobii* sp. nov. as a monophyletic lineage (Fig. 2) that is sister to *A.marxi* (distributed along the Colorado Plateau) and phylogenetically distinct from the other tarantula species endemic to the Chiricahua Mountains (i.e., *A.chiricahua*). *Aphonopelmajacobii* sp. nov. is probably the only tarantula species encountered in the high-elevation mixed conifer forests of the Chiricahua Mountains, but its distribution overlaps with *A.chalcodes* Chamberlin, 1940, *A.chiricahua*, *A.gabeli* Smith, 1995, and *A.vorhiesi* at lower elevations in the oak and pine-oak woodlands.

**Figure 8.**
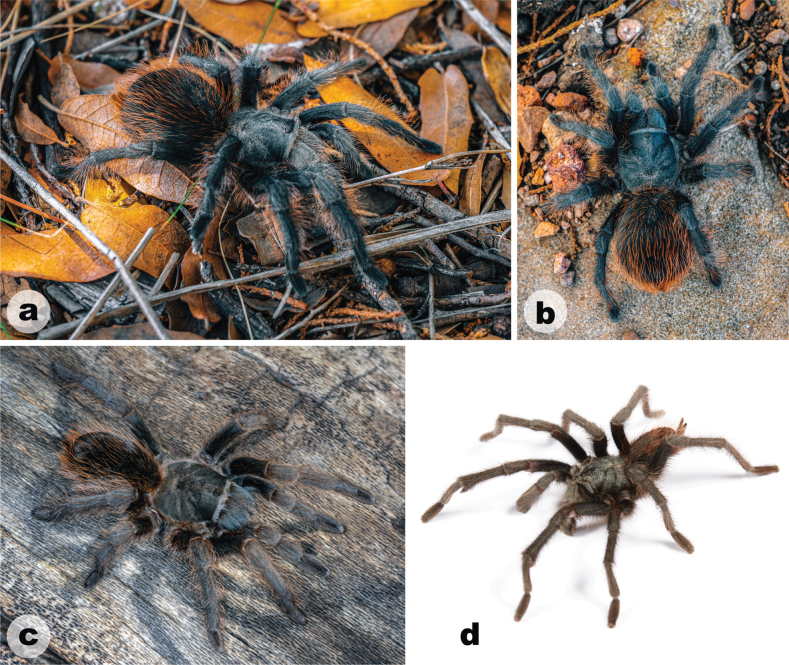
Live habitus of *Aphonopelmachiricahua***a, b** in situ female (APH-5400) **c** female (APH-5050) **d** male (APH-5144). Photographs of images **a** and **b** provided by Leonardo Chávez; photograph of image **c** provided by Michael A. Jacobi.

*Aphonopelmajacobii* sp. nov. is readily distinguished from adult *A.chalcodes* and *A.gabeli* by coloration and size (Fig. 3; Hamilton et al. 2016: figs 30, 45). Males of the new species are similar in appearance to *A.vorhiesi* due to their shared coloration (i.e., general black body with bright orange or red setae on the abdomen, Fig. 3a; Hamilton et al. 2016: fig. 142), but are noticeably smaller (Cl 6.708–8.955 vs 10.018–13.980), possess a larger A3/M4 ratio (0.659–0.790 vs 0.469–0.566), and breed later in the fall (October–November vs July–October) (note: males of *A.vorhiesi* found in October are generally worn and faded whereas males of *A.jacobii* sp. nov. are lively and vibrant). Males of *A.jacobii* sp. nov. and *A.chiricahua* are very similar morphologically and behaviorally. They possess similar coloration (Figs 3a, 8d) and share a common breeding period, but the new species does separate slightly from *A.chiricahua* in PCA morphospace (S4A), is statistically smaller (Cl 7.679 ± 0.71 vs 9.864 ± 2.00, *t* (18) = 3.6964, *P* = 0.0017), possesses a slightly smaller T1/P4 ratio (2.175–2.545 vs 2.576–2.991), and has a proportionally shorter embolus relative to the palpal bulb (Fig. 4g, h; Hamilton et al. 2016: fig. 37g, h). Males of *A.jacobii* sp. nov. can be further distinguished from other members of the *Marxi* species group by the following important ratios and measurements: T1/P4 (2.175–2.545) is smaller than *A.catalina* (2.814–3.033); A3/M4 (0.659–0.790) is larger than *A.bacadehuachi* (0.495), *A.catalina* (0.477–0.520), *A.madera* (0.540–0.602), and *A.peloncillo* (0.457–0.581); and Bl_r (2.505–3.061) is smaller than *A.marxi* (3.194–3.781). Additional ratios that might be useful for separating males of *A.jacobii* sp. nov. from various other members of the *Marxi* species group include Cl/A3, Cl/M3, PTl/M3, PTl/M4, and T1/F3 (see Suppl. material 5).

Females of *A.jacobii* sp. nov. are noticeably smaller than *A.chiricahua* and *A.vorhiesi* (Cl 7.621–9.018 v. 14.230–15.530 and 11.230–16.380, respectively), separate in PCA morphospace (S4B), and possess slightly different coloration (Figs 3b, 8a–c; Hamilton et al. 2016: fig. 142). Furthermore, females of the new species can be distinguished from other members of the *Marxi* species group by the following important ratio: M3/F4 (0.509–0.534) is smaller than *A.bacadehuachi* (0.613), *A.catalina* (0.582–0.604), *A.madera* (0.550–0.616), *A.marxi* (0.550–0.598), *A.peloncillo* (0.598–0.655), and *A.vorhie*s*i* (0.587–0.657). Additional ratios that might be useful for separating females of *A.jacobii* sp. nov. from various other members of the *Marxi* species group include F1/T4, M1/A3, SC4, and T1/T4 (see Suppl. material 5).

##### Description of male holotype

**(APH-5002: Figs 3a, 4).** Specimen collected alive wandering on road, preserved in 80% ethanol; original coloration faded due to preservation (Fig. 3a). Left legs I, III, IV, and left pedipalp removed for measurements and photographs; stored in vial with specimen. Right leg III removed for DNA and stored at -80 °C at UIM. General coloration: black or faded black. Cephalothorax: Cl 6.708, Cw 6.509; densely clothed with black/faded black pubescence, appressed to surface; fringe covered in long setae not closely appressed to surface; foveal groove medium deep and slightly procurved; pars cephalica region rises gradually from foveal groove, gently arching anteriorly toward ocular area; AER procurved, PER slightly recurved; normal sized chelicerae; clypeus generally straight but extends forward on a slight curve near the eyes; LBl 0.942, LBw 1.596; sternum hirsute, clothed with short black/brown, densely packed setae. Abdomen: densely clothed in short black/brown pubescence with numerous longer, paler setae interspersed (generally red or orange in vita, Fig. 3a); dense dorsal patch of black Type I urticating setae (Cooke et al. 1972); ventral setae same as dorsal. Legs: Hirsute; densely clothed with short, similar length black/brown setae, and longer setae dorsally. Metatarsus I slightly curved. F1 7.478; F1w 1.836; P1 2.780; T1 6.482; M1 4.543; A1 4.251; L1 length 25.534; F3 5.765; F3w 1.823; P3 2.438; T3 4.445; M3 4.559; A3 4.475; L3 length 21.682; F4 7.082; F4w 1.554; P4 2.573; T4 6.199; M4 6.068; A4 4.980; L4 length 26.902; femur III is normal, not noticeably swollen or wider than other legs (Fig. 4c). All tarsi fully scopulate. Extent of metatarsal scopulation: leg III (SC3) = 48.8%, leg IV (SC4) = 29.3% (Fig. 4d, e). Six ventral spinose setae (megaspines), one prolateral spinose seta, and four ventral spinose setae at the apical edge on metatarsus III; nine ventral spinose setae (megaspines), two prolateral and one retrolateral spinose setae, and eight ventral spinose setae at the apical edge on metatarsus IV; one prolateral megaspine and two ventral megaspines are present on the prolateral tibia of the mating clasper (tibia I); two megaspines, that project anteriorly, can be found at the apex of each tibial apophyses (Fig. 4i). Coxa I: prolateral surface a mix of fine hair-like and very thin tapered setae (Fig. 4b). Pedipalps: hirsute; densely clothed in the same setal color as the other legs, with numerous longer ventral setae; one spinose seta at the apical, prolateral femur and two spinose setae on the prolateral tibia (Fig. 4f); PTl 4.627, PTw 1.763. When extended, embolus tapers with a gentle curve to the retrolateral side near apex, embolus slender, no keels (Fig. 4g, h).

***Male variation*** (*n* = 14). Cl 6.708–8.955 (7.679 ± 0.71), Cw 6.254–8.654 (7.467 ± 0.23), LBl 0.684–1.340 (0.930 ± 0.05), LBw 0.985–1.971 (1.513 ± 0.09), F1 7.145–9.585 (8.220 ± 0.19), F1w 1.821–2.596 (2.124 ± 0.06), P1 2.780–3.825 (3.225 ± 0.08), T1 5.851–7.851 (6.863 ± 0.54), M1 4.090–5.524 (4.807 ± 0.11), A1 3.572–4.975 (4.307 ± 0.10), L1 length 23.568–31.239 (27.422 ± 0.59), F3 5.591–7.285 (6.396 ± 0.14), F3w 1.688–2.478 (2.012 ± 0.06), P3 2.304–3.177 (2.620 ± 0.07), T3 4.162–5.726 (4.812 ± 0.12), M3 4.379–5.807 (4.916 ± 0.11), A3 3.955–5.389 (4.724 ± 0.09), L3 length 20.391–27.358 (23.468 ± 0.50), F4 6.648–9.006 (7.728 ± 0.18), F4w 1.554–2.349 (1.90 ± 0.06), P4 2.524–3.516 (2.861 ± 0.08), T4 5.784–7.380 (6.647 ± 0.13), M4 5.772–8.177 (6.762 ± 0.16), A4 4.944–6.379 (5.464 ± 0.11), L4 length 25.672–34.458 (29.463 ± 0.62), PTl 4.420–5.822 (5.113 ± 0.11), PTw 1.763–2.419 (2.041 ± 0.05), SC3 ratio 0.414–0.609 (0.533 ± 0.01), SC4 ratio 0.283–0.404 (0.351 ± 0.01), coxa I setae = fine/very thin and tapered, femur III condition = normal, not noticeably swollen or wider than other legs.

##### Description of female paratype

**(APH-5001: Figs 3b, 5).** Specimen collected alive from burrow, preserved in 80% ethanol; original coloration faded due to preservation (Fig. 5a). Left legs I, III, IV, and pedipalp removed for photographs and measurements; stored in vial with specimen. Right leg III removed for DNA and stored at -80 °C at UIM. Genital plate with spermathecae removed and cleared, stored in vial with specimen. General coloration: black or faded black and brown. Cephalothorax: Cl 9.018, Cw 8.908; hirsute, densely clothed with black/faded black, pubescence closely appressed to surface; fringe densely covered in longer setae; foveal groove medium deep and slightly procurved; pars cephalica region gently rises from thoracic furrow, arching anteriorly towards ocular area; AER procurved, PER slightly recurved; robust chelicerae, clypeus extends forward on a slight curve; LBl 1.403, LBw 2.110; sternum hirsute, clothed with shorter black/faded black setae. Abdomen: densely clothed dorsally in short black setae with numerous longer, paler setae interspersed (generally red or orange in vita, Fig. 3b); dense dorsal patch of black Type I urticating setae (Cooke et al. 1972); ventral setae same as dorsal. Spermathecae (Fig. 5f): paired and separated, tapering from wide bases (not fused) and slightly curving medially towards capitate bulbs. Legs: very hirsute, particularly ventrally; densely clothed in short and medium black/brown pubescence, with longer setae colored similarly as the long abdominal setae; F1 7.661; F1w 2.481; P1 3.568; T1 5.864; M1 3.553; A1 3.854; L1 length 24.500; F3 6.206; F3w 2.032; P3 2.628; T3 4.230; M3 4.191; A3 4.340; L3 length 21.595; F4 7.885; F4w 2.122; P4 2.969; T4 6.156; M4 6.079; A4 4.845; L4 length 27.904. All tarsi fully scopulate. Extent of metatarsal scopulation: leg III (SC3) = 50.4%, leg IV (SC4) = 27.3% (Fig. 5d). Two ventral spinose setae (megaspines) and six ventral spinose setae at the apical edge on metatarsus III; eight ventral spinose setae (megaspines), one prolateral spinose setae, and six ventral spinose setae at the apical edge on metatarsus IV. Coxa I: prolateral surface a mix of fine hair-like and very thin tapered setae (Fig. 5b). Pedipalps (Fig. 5e): Densely clothed in the same setal color as the other legs; two prolateral megaspines (one of these apical) and two apical ventral megaspines are present on the palpal tibia.

***Female variation*** (*n* = 6). Cl 7.621–9.018 (8.320 ± 0.44), Cw 7.433–8.908 (8.171 ± 0.47), LBl 1.261–1.403 (1.332 ± 0.04), LBw 1.978–2.110 (2.044 ± 0.04), F1 6.692–7.661 (7.177 ± 0.31), F1w 2.131–2.481 (2.306 ± 0.11), P1 2.885–3.568 (3.227 ± 0.22), T1 4.952–5.864 (5.399 ± 0.32), M1 3.230–3.553 (3.392 ± 0.10), A1 3.530–3.854 (3.692 ± 0.10), L1 length 21.659–24.500 (23.080 ± 0.90), F3 5.383–6.206 (5.795 ± 0.26), F3w 1.808–2.032 (1.920 ± 0.07), P3 2.408–2.628 (2.518 ± 0.07), T3 3.847–4.230 (4.039 ± 0.12), M3 3.608–4.191 (3.900 ± 0.18), A3 4.104–4.340 (4.222 ± 0.07), L3 length 19.350–21.595 (20.473 ± 0.71), F4 6.867–7.855 (7.361 ± 0.31), F4w 1.968–2.122 (2.045 ± 0.05), P4 2.596–2.969 (2.783 ± 0.12), T4 5.630–6.156 (5.893 ± 0.17), M4 5.309–6.079 (5.694 ± 0.24), A4 4.741–4.845 (4.793 ± 0.03), L4 length 25.143–27.904 (26.524 ± 0.87), SC3 ratio 0.505–0.571 (0.538 ± 0.02), SC4 ratio 0.273–0.275 (0.274 ± 0.01), coxa I setae = fine/very thin and tapered. Spermathecae variation as in Fig. 5f, Suppl. material 6 (and Hamilton et al. 2016: fig. 38 for APH-2097).

##### Other material.

United States – Arizona • Cochise County • 1♀; Chiricahua Mountains, Southwest Research Station; 30 Nov. 1965; Jon Jenson leg.; AMNH; APH-2097 • 1♂; Chiricahua Mountains, Cave Creek Canyon; 30 Nov. 1963; V. Roth leg.; AMNH; APH-2101 • 1♂; Chiricahua Mountains, Upper Cave Creek Canyon; 1966; Marlene Posedly leg.; AMNH; APH-2102 • 1♂; Chiricahua Mountains, Southwest Research Station; 31 Oct. 1956; E. Ordway leg.; AMNH; APH-2105 • 1♂; Chiricahua Mountains, Sunny Flat; 30 Oct. 1971; V. Roth leg.; AMNH; APH-2480-A • 1♂; Chiricahua Mountains, Southwest Research Station; 20 Nov. 1971; V. Roth leg.; AMNH; APH-2480-B • 1♂; Chiricahua Mountains, Rustler and Long Park; 4 Nov. 1970; Joan Harper leg.; AMNH; APH-2548 • 1 imm.; Chiricahua Mountains, Barfoot Park Helispot; 31.91516°N, 109.28504°W^1^; 2493 m; 27 Oct. 2019; Tom Patterson, Brent E. Hendrixson, Chris A. Hamilton, Michael A. Jacobi, Chad Campbell & Wyatt Mendez leg.; UIM; APH-4005 • 1 imm.; Chiricahua Mountains, along Forest Road 42D above Onion Saddle; 31.92838°N, 109.26311°W^1^; 2364 m; 20 Oct. 2019; Wyatt Mendez leg.; UIM; APH-4024 • 1♀; Chiricahua Mountains, on hillside along Forest Road 42A; 31.88062°N, 109.22087°W^1^; 1717 m; 27 Oct. 2019; Chris A. Hamilton, Brent E. Hendrixson, Michael A. Jacobi, Wyatt Mendez, Chad Campbell & Tom Patterson leg.; UIM; APH-4006 • 2♀; Chiricahua Mountains, Barfoot Park Helispot; 31.91516°N, 109.28504°W^1^; 2493 m; 27 Oct. 2019; Wyatt Mendez, Brent E. Hendrixson, Chris A. Hamilton, Michael A. Jacobi, Chad Campbell & Tom Patterson leg.; UIM; APH-4018; AMNH; APH-4019 • 1♂; Chiricahua Mountains, along Forest Road 42; 31.88139°N, 109.18732°W^1^; 1593 m; 26 Oct. 2019; Chris A. Hamilton & Brent E. Hendrixson leg.; UIM; APH-4020 • 1♂; Chiricahua Mountains, 1 Pogo Hill; 31.88061°N, 109.20386°W^1^; 1662 m; 27 Oct. 2019; Chris A. Hamilton, Brent E. Hendrixson & Wyatt Mendez leg.; UIM; APH-4022 • 1♂; Chiricahua Mountains, Barfoot Park Helispot; 31.91516°N, 109.28504°W^1^; 2493 m; 26 Oct. 2019; Chad Campbell, Michael A. Jacobi & Tom Patterson leg.; UIM; APH-4029 • 1♀; Chiricahua Mountains, 1 Pogo Hill; 31.88061°N, 109.20386°W^1^; 1662 m; 9 Sept. 2019; Wyatt Mendez leg.; UIM; APH-5079 • 2♂; Chiricahua Mountains, Barfoot Park Helispot; 31.91516°N, 109.28504°W^1^; 2493 m; 3 Nov. 2019; Wyatt Mendez leg.; UIM; APH-5080, APH-5081 • 1♂; Chiricahua Mountains, along Forest Road 42D above Onion Saddle; 31.92838°N, 109.26311°W^1^; 2364 m; 8 Nov. 2019, Wyatt Mendez leg.; UIM; APH-5082.

##### Distribution and natural history.

*Aphonopelmajacobii* sp. nov. is endemic to the Chiricahua Mountains (Figs 7, 11) in southeastern Arizona where it has been encountered in plant communities ranging from mid-elevation Madrean evergreen oak woodlands in Cave Creek Canyon (Fig. 7e) to high-elevation mixed conifer forests near Onion Saddle (Fig. 7a, b) and Barfoot Park (Fig. 7c, d). To our knowledge, it is the only tarantula in the Chiricahua Mountains encountered above the pine-oak woodland zone. The highest confirmed elevation record for this species—as observed by us—is just below 2500 m at Barfoot Park, but other sightings suggest it is found perhaps as high as 2700 m near Rustler and Long Parks (see APH-2548, misidentified as *A.chiricahua* in Hamilton et al. 2016). In the United States, only *A.marxi* has been reported from higher elevations (~ 2830 m in the Chuska Mountains of northeastern New Mexico, APH-0452 in Hamilton et al. 2016), but still far below the elevation records for the remarkable neotropical tarantula genera *Antikuna* Kaderka, Ferretti, West, Lüddecke & Hüsser, 2021 (4689 m, Kaderka et al. 2021), *Hapalotremus* Simon, 1903 (4524 m, Ferretti et al. 2018), *Bistriopelma* Kaderka, 2015 (4398 m, Kaderka 2015), and *Euathlus* Ausserer, 1875 (4153 m, Quispe-Colca and Ferretti 2021) from the South American Andes.

Mature female and immature individuals of *A.jacobii* sp. nov. have only been extracted from burrows (i.e., specimens have not been observed beneath rocks or other surface debris). Burrows are generally located in meadows or exposed patches of soil with limited overstory structure. This perhaps allows their burrows to receive more direct sunlight to maintain higher temperatures in these otherwise cool habitats. Burrow entrances of mature females measure c. 15 mm in diameter and have been observed with (Fig. 7f) and without traces of silk along the perimeter. The breeding period for this species appears to be limited. Mature males are active during October and November, similar to other fall-breeding members of the *Marxi* species group in southeastern Arizona, including *A.chiricahua* (Hendrixson et al. 2015; Hamilton et al. 2016). In fact, males of *A.jacobii* sp. nov. and *A.chiricahua* (APH-4020 and APH-4023, respectively) were observed within 250 m of each other on consecutive days in late October 2019. Males are most frequently encountered during daylight hours, but one individual (APH-4022) was observed wandering on a mild evening (c. 20 °C) during early twilight. Two other males (APH-5002, APH-5003) were observed at the type locality on a breezy and cool morning (~ 5–10 °C, ~ 1030 hrs).

The discovery of *A.jacobii* sp. nov. documents the first known case of syntopy between five species of *Aphonopelma* (i.e., the distributions of *A.jacobii* sp. nov., *A.chalcodes*, *A.chiricahua*, *A.gabeli*, and *A.vorhiesi* overlap in Cave Creek Canyon). As noted above, the breeding periods of *A.jacobii* sp. nov. and *A.chiricahua*—but not *A.chalcodes*, *A.gabeli*, or *A.vorhiesi*—coincide with each other. It is unknown how these two species maintain cohesion and reproductive isolation in the face of significant overlap between their distributions and breeding periods. Future studies should investigate the various factors that promote selection for prezygotic or postzygotic reproductive barriers and reduce potential hybridization between these synchronously breeding populations (see also Prentice 1997).

#### ﻿ Aphonopelma chiricahua

Taxon classificationAnimaliaAraneaeTheraphosidae

Hamilton, Hendrixson & Bond, 2016

F2326415-38F0-5363-8D97-11DB8F14709F

**Figure 9.**
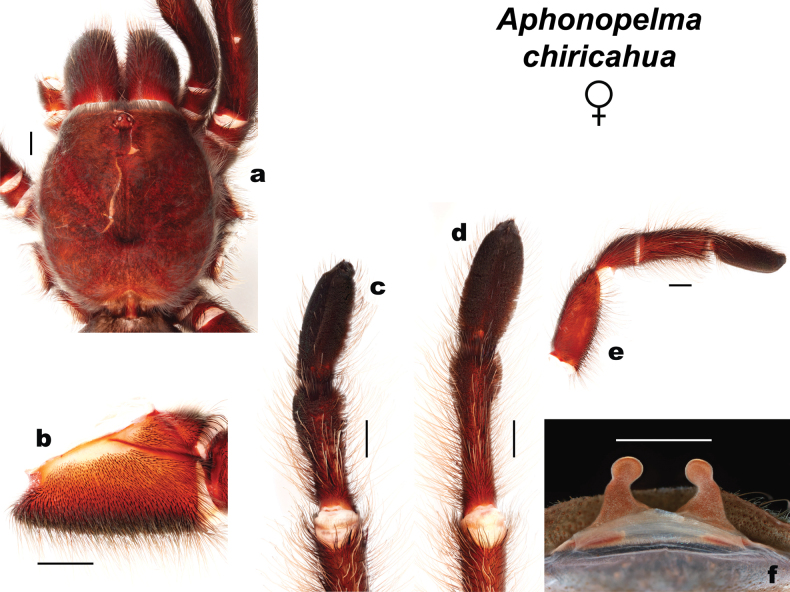
Morphology of *Aphonopelmachiricahua* (female, APH-5400) **a** carapace, dorsal view **b** coxa of leg I, prolateral view **c** metatarsus and tarsus of leg III, ventral view **d** metatarsus and tarsus of leg IV, ventral view **e** pedipalp, prolateral view **f** spermathecae. Scale bars: 2 mm.

**Figure 10.**
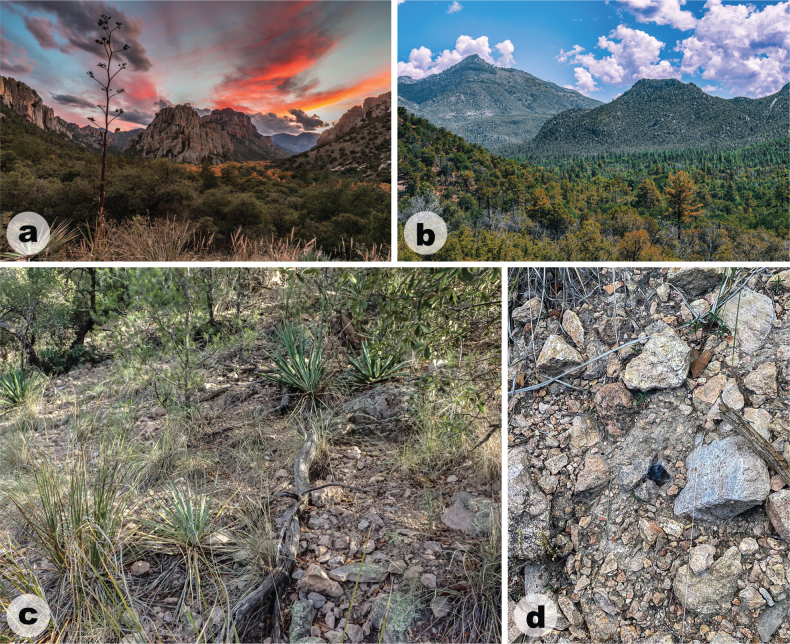
Habitat images of *Aphonopelmachiricahua* from the Chiricahua Mountains, Cochise County, Arizona **a, b** Madrean pine-oak woodland near the Cathedral Vista Trail off Forest Road 42 **c** rocky and grassy microhabitat near the Cathedral Vista Trail **d** silk-covered burrow of a mature female (APH-5400). Photographs of images **b** and **d** provided by Leonardo Chávez.

**Figure 11.**
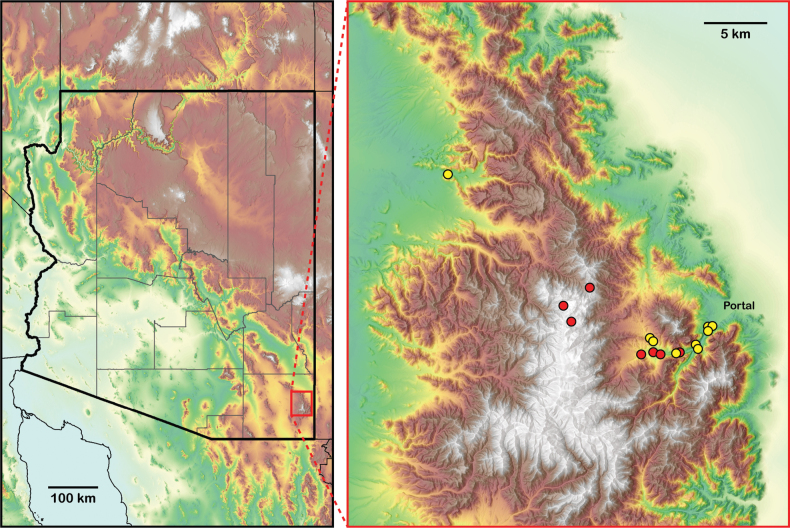
Map showing the known distribution of *Aphonopelmajacobii* sp. nov. (red circles) and *A.chiricahua* (yellow circles) in the Chiricahua Mountains, Cochise County, Arizona.

Figs 8 , 9 , 10 , 11

Aphonopelma chiricahua Hamilton et al. 2016: 90–93, 95–98, figs 37, 39.

##### Type material.

***Holotype*.** United States • ♂; Arizona, Cochise County, Chiricahua Mountains, Cave Creek Canyon, 1.6 km past the Cathedral Vista Trailhead along Forest Road 42 (toward the Southwest Research Station); 31.88133°N, 109.18797°W^4^; 1600 m; 14 Nov. 2013; Helen Snyder leg.; UIM; APH-3191.

##### Remarks.

In the original description of *A.chiricahua*, Hamilton et al. (2016: 98) stated: “Of particular note is the size of the holotype male and paratype female; the two specimens probably represent opposite extremes on the size spectrum for what is possible in this species. The rather large holotype male was chosen because it was a fresh specimen and could be associated with molecular data that was unique from all other samples in the area, at the time. The female, though small, is sexually mature (based on spermathecal development).” Based on the body size, morphology, and collection data for the female paratype of *A.chiricahua* (APH-2097), we have determined that the specimen was misidentified and should now be considered *A.jacobii* sp. nov. Similarly, except for the male holotype (APH-3191), we have determined that the male specimens of *A.chiricahua* reported in Hamilton et al. (2016) were misidentified and should be considered *A.jacobii* sp. nov. too. Consequently, a redescription and emended diagnosis for *A.chiricahua* are necessary to reassess limits of morphological variation in the species. The following redescription of *A.chiricahua* is based on several newly acquired individuals (mature males and females) whose identities have been confirmed by comparing their COX1 and UCE sequence data to the male holotype (Figs 1, 2; unpublished data).

##### Emended diagnosis.

*Aphonopelmachiricahua* is a member of the *Marxi* species group and can be distinguished by a combination of morphological, genomic, behavioral, and distributional features. This species is a mid- to late-fall breeder endemic to the Chiricahua Mountains in southeastern Arizona. Mitochondrial and nuclear DNA identifies *A.chiricahua* as a monophyletic lineage (Figs 1, 2) that is sister to *A.catalina* (and an undetermined species) and phylogenetically distinct from the other tarantula species endemic to the Chiricahua Mountains (i.e., *A.jacobii* sp. nov.). *Aphonopelmachiricahua* is found in oak and pine-oak woodlands where its distribution overlaps with *A.chalcodes*, *A.gabeli*, *A.jacobii* sp. nov., and *A.vorhiesi*.

For features that can be used to distinguish *A.chiricahua* from *A.jacobii* sp. nov., refer to the diagnosis of the latter species provided above. When in doubt, the identity of both species (including immature specimens) can be readily confirmed with COX1 barcoding. *Aphonopelmachiricahua* is readily distinguished from *A.chalcodes* and *A.gabeli* by coloration (Fig. 8; Hamilton et al. 2016: figs 30, 45). Males of *A.chiricahua* are similar in appearance to *A.vorhiesi* due to their shared coloration (i.e., general black body with bright orange or red setae on the abdomen, Fig. 8d; Hamilton et al. 2016: fig. 142), but possess a larger A3/M4 ratio (0.643–0.697 vs 0.469–0.566), and breed later in the fall (October–December vs July–October) (note: males of *A.vorhiesi* found in October are generally worn and faded whereas males of *A.chiricahua* are lively and vibrant). Males of *A.chiricahua* can be further distinguished from other members of the *Marxi* species group by the following important ratios: A3/M4 (0.643–0.697) is larger than *A.bacadehuachi* (0.495), *A.catalina* (0.477–0.520), *A.madera* (0.540–0.602), and *A.peloncillo* (0.457–0.581); and F1/T1 (1.118–1.196) is slightly smaller than *A.marxi* (1.199–1.297). Additional ratios that might be useful for separating males of *A.chiricahua* from various other members of the *Marxi* species group include Cl/A3, Cl/M3, F3/M4, PTl/M3, PTl/M4, PTl/w, and T1/F3 (see Suppl. material 5).

Females of *A.chiricahua* are similar in appearance to *A.vorhiesi* due to their overlapping body sizes (Cl 14.230–15.530 v. 11.230–16.380) but possess slightly different coloration (Fig. 8a–c; Hamilton et al. 2016: fig. 142) and a larger T1/P4 ratio (2.217–2.311 vs 1.774–2.091). Furthermore, females of *A.chiricahua* can be distinguished from other members of the *Marxi* species group by the following important ratio: T1/P4 (2.217–2.311) is larger than *A.bacadehuachi* (0.781), *A.catalina* (1.985–2.045), *A.madera* (1.854–2.097), *A.marxi* (1.909–2.108), and *A.peloncillo* (1.704–2.013). Additional ratios that might be useful for separating females of *A.chiricahua* from various other members of the *Marxi* species group include SC4 and M1/M4 (see Suppl. material 5).

##### Redescription of male holotype

**(APH-3191 Hamilton et al. 2016: figs 36, 37).** Specimen collected alive wandering on road, preserved in 80% ethanol; original coloration faded due to preservation (Hamilton et al. 2016: fig. 37a). Left legs I, III, IV, and left pedipalp removed for measurements and photographs; stored in vial with specimen. Right legs III and IV removed for DNA and stored at -80 °C at UIM. General coloration: black or faded black. Cephalothorax: Cl 11.420, Cw 11.220; densely clothed with black/faded black pubescence, slightly appressed to surface and longer than lower elevation species, slight iridescence; fringe covered in long setae not closely appressed to surface; foveal groove medium deep and straight; pars cephalica region rises gradually from foveal groove, gently arching anteriorly toward ocular area; AER slightly procurved, PER very slightly recurved; normal sized chelicerae; clypeus slightly extends forward on a curve; LBl 1.37, LBw 1.61; sternum hirsute, clothed with medium black, densely packed setae. Abdomen: densely clothed in short black/brown pubescence with numerous longer, paler setae interspersed (generally red or orange in vita, Hamilton et al. 2016: fig. 36), longer with a more hirsute appearance than lower elevation species; dense dorsal patch of black Type I urticating setae (Cooke et al. 1972); ventral setae same as dorsal. Legs: hirsute; densely clothed with medium length black/brown setae, and longer setae ventrally. Metatarsus I slightly curved. F1 12.72; F1w 3.28; P1 4.95; T1 11.37; M1 7.61; A1 6.16; L1 length 42.812; F3 9.53; F3w 2.98; P3 4.11; T3 7.60; M3 7.79; A3 6.84; L3 length 35.878; F4 11.41; F4w 3.20; P4 4.41; T4 9.67; M4 10.28; A4 7.78; L4 length 43.559; femur III is normal, not noticeably swollen or wider than other legs (Hamilton et al. 2016: fig. 37c). All tarsi fully scopulate. Extent of metatarsal scopulation: leg III (SC3) = 65.5%, leg IV (SC4) = 37.9% (Hamilton et al. 2016: fig. 37d, e). Three ventral spinose setae (megaspines), one retrolateral spinose seta, and five ventral spinose setae at the apical edge on metatarsus III; nine ventral spinose setae (megaspines), one prolateral spinose setae, and three ventral spinose setae at the apical edge on metatarsus IV; two ventral megaspines are present on mating clasper (tibia I); three megaspines, that project anteriorly, can be found on the ventral tibial apophysis (Hamilton et al. 2016: fig. 37i). Coxa I: prolateral surface a mix of fine hair-like and thin/very thin tapered setae (Hamilton et al. 2016: fig. 37b). Pedipalps: hirsute; densely clothed in the same setal color as the other legs, with numerous longer ventral setae; one spinose seta at the apical, prolateral femur and four spinose setae on the prolateral tibia (Hamilton et al. 2016: fig. 37f); PTl 7.34, PTw 2.82. When extended, embolus tapers with a gentle curve to the retrolateral side near apex, embolus slender, no keels (Hamilton et al. 2016: fig. 37g, h).

***Male variation*** (*n* = 6). Cl 7.673–12.230 (9.864 ± 2.00), Cw 6.968–11.620 (9.295 ± 0.87), LBl 0.886–1.368 (1.147 ± 0.08), LBw 1.609–2.019 (1.773 ± 0.07), F1 8.560–13.229 (10.767 ± 0.86), F1w 1.892–3.281 (2.465 ± 0.22), P1 3.180–4.947 (3.965 ± 0.31), T1 7.529–11.372 (9.396 ± 0.72), M1 4.452–7.911 (6.307 ± 0.61), A1 3.911–6.605 (5.279 ± 0.49), L1 length 27.681–43.106 (35.713 ± 2.96), F3 6.325–9.882 (8.167 ± 0.70), F3w 1.673–3.038 (2.499 ± 0.25), P3 2.397–4.112 (3.180 ± 0.29), T3 4.605–7.673 (6.275 ± 0.55), M3 4.603–7.919 (6.414 ± 0.62), A3 4.452–6.952 (5.702 ± 0.49), L3 length 22.517–35.878 (29.738 ± 2.61), F4 7.650–12.048 (9.817 ± 0.83), F4w 1.638–3.205 (2.348 ± 0.24), P4 2.593–4.414 (3.368 ± 0.29), T4 6.314–10.272 (8.129 ± 0.66), M4 6.384–10.378 (8.616 ± 0.76), A4 4.967–7.880 (6.415 ± 0.51), L4 length 28.192–43.726 (36.345 ± 2.98), PTl 4.885–7.529 (6.375 ± 0.50), PTw 1.933–3.171 (2.536 ± 0.20), SC3 ratio 0.542–0.656 (0.59 ± 0.02), SC4 ratio 0.220–0.416 (0.324 ± 0.03), coxa I setae = fine/very thin and tapered, femur III condition = normal, not noticeably swollen or wider than other legs.

##### Description of new female exemplar

**(APH-5400: Figs 8a, b, 9).** Specimen collected live from burrow, preserved in 80% ethanol; original coloration faded due to preservation (Fig. 9a). Left legs I, III, IV, and pedipalp removed for photographs and measurements; stored in vial with specimen. Genital plate with spermathecae removed and cleared, stored in vial with specimen. General coloration: dark brown and faded black. Cephalothorax: Cl 15.530, Cw 14.350; hirsute, densely clothed with brown/black pubescence closely appressed to surface; fringe densely covered in longer setae; foveal groove medium deep and slightly procurved; pars cephalica region gently rises from thoracic furrow, arching anteriorly toward ocular area; carapace was cracked during specimen measurements; AER slightly procurved, PER recurved; robust chelicerae, clypeus is generally straight but extends forward on a slight curve in front of the eyes; LBl 1.65, LBw 2.69; sternum hirsute, clothed with medium short brown setae. Abdomen: densely clothed dorsally in black/brown setae with numerous longer, paler setae interspersed (generally red or orange in vita, Fig. 8a, b); dense dorsal patch of black Type I urticating setae (Cooke et al. 1972); ventral setae shorter than dorsal. Spermathecae (Fig. 9f): paired and separated, tapering from wide bases (not fused) and slightly curving medially towards capitate bulbs. Legs: hirsute; densely clothed in short and medium black/brown pubescence; F1 12.486, F1w 4.127, P1 5.202, T1 9.772, M1 6.865, A1 6.037, L1 length 40.362, F3 10.323, F3w 3.768, P3 4.409, T3 6.94, M3 6.828, A3 7.108, L3 length 35.608, F4 11.915, F4w 3.634, P4 4.228, T4 9.58, M4 9.569, A4 6.976, L4 length 42.268. All tarsi fully scopulate. Extent of metatarsal scopulation: leg III (SC3) = 41.6%, leg IV (SC4) = 43.3% (Fig. 9c, d). Three ventral spinose setae (megaspines), one retrolateral spinose seta, and two ventral spinose setae at the apical edge on metatarsus III; four ventral spinose setae (megaspines), one prolateral spinose setae, and three ventral spinose setae at the apical edge on metatarsus IV. Coxa I: prolateral surface a mix of fine hair-like and thin tapered setae (Fig. 9b). Pedipalps (Fig. 9e): densely clothed in the same setal color as the other legs; one megaspine at the apical edge of the prolateral femur, five prolateral megaspines on the tibia (two on the apical edge), one ventral megaspine on the tibia.

***Female variation*** (*n* = 2). Cl 14.230–15.530 (14.880 ± 0.65), Cw 12.960–14.350 (13.655 ± 0.69), LBl 1.62–1.65 (1.635 ± 0.01), LBw 2.690–2.874 (2.782 ± 0.09), F1 11.362–12.486 (11.924 ± 0.56), F1w 4.058–4.127 (4.093 ± 0.03), P1 5.121–5.202 (5.162 ± 0.04), T1 9.599–9.772 (9.686 ± 0.09), M1 6.151–6.865 (6.508 ± 0.36), A1 5.838–6.037 (5.938 ± 0.10), L1 length 38.071–40.362 (39.217 ± 1.15), F3 8.980–10.323 (9.652 ± 0.67), F3w 3.190–3.768 (3.479 ± 0.29), P3 3.737–4.409 (4.073 ± 0.34), T3 6.319–6.940 (6.630 ± 0.31), M3 6.761–6.828 (6.795 ± 0.03), A3 5.657–7.108 (6.383 ± 0.73), L3 length 31.454–35.608 (33.531 ± 2.08), F4 11.749–11.915 (11.832 ± 0.08), F4w 3.418–3.634 (3.526 ± 0.11), P4 4.228–4.329 (4.279 ± 0.05), T4 9.439–9.580 (9.510 ± 0.07), M4 9.162–9.569 (9.366 ± 0.20), A4 6.697–6.976 (6.837 ± 0.14), L4 length 41.376–42.268 (41.822 ± 0.45), SC3 ratio 0.417–0.524 (0.47 ± 0.05), SC4 ratio 0.408–0.434 (0.421 ± 0.01), coxa I setae = fine/thin and tapered. Spermathecae variation as in Fig. 9f, Suppl. material 7.

##### Other material.

United States – Arizona • Cochise County • 1 imm.; Chiricahua Mountains, along Forest Road 42; 31.89129°N, 109.21079°W^1^; 1693 m; 26 Oct. 2019; Brent E. Hendrixson, Chris A. Hamilton, Michael A. Jacobi, Chad Campbell & Tom Patterson leg.; UIM; APH-4021 • 1♂; Chiricahua Mountains, along Forest Road 42; 31.88151°N, 109.18972°W^1^; 1608 m; 27 Oct. 2019; Wyatt Mendez leg.; UIM; APH-4023 • 1♂; Chiricahua Mountains, Cave Creek Canyon, along Forest Road 42; 31.90136°N, 109.15945°W^1^; 1501 m; 31 Oct. 2018; Brent E. Hendrixson & Michael A. Jacobi leg.; UIM; APH-5004 • 1♂; Chiricahua Mountains, Cave Creek Canyon, along Forest Road 42; 31.90152°N, 109.15932°W^1^; 1501 m; 11 Oct. 2018; Michael A. Jacobi leg., UIM; APH-5005 • 1♂; Chiricahua Mountains, 1 km from entrance to Chiricahua National Monument; 32.00918°N, 109.38123°W^1^; 1574 m; 30 Oct. 2018; Brent E. Hendrixson & Michael A. Jacobi leg.; UIM; APH-5006 • 1 imm.; Chiricahua Mountains, Cave Creek Visitor Information Center restroom; 31.89902°N, 109.16243°W^1^; 1512 m; 17 Oct. 2018; Michael A. Jacobi leg.; UIM; APH-5010 • 1♂; Chiricahua Mountains, Cave Creek Canyon, along Forest Road 42 at horse corral; 31.89820°N, 109.16286°W^1^; 1515 m; 16 Nov. 2018; Michael A. Jacobi leg.; AMNH; APH-5049 • 1♀; Chiricahua Mountains, on hillside along Forest Road 42; 31.89057°N, 109.21072°W^1^; 1720 m; 21 June 2018; Michael A. Jacobi leg.; UIM; APH-5050 • 1 imm.; Chiricahua Mountains, Cave Creek Canyon, Cathedral Vista Point Trail; 31.88529°N, 109.17266°W^1^; 1567 m; 11 Nov. 2019; Wyatt Mendez & Walter Schoepfle leg.; UIM; APH-5078 • 1♂; Chiricahua Mountains, Cave Creek Visitor Information Center; 31.89916°N, 109.16204°W^4^; 1506 m; 9 Nov. 2021; David Jasper leg.; UIM; APH-5126 • 1♂; Chiricahua Mountains, along Forest Road 42; 31.88151°N, 109.19304°W^4^; 1618 m; 12 Oct. 2021; Wyatt Mendez leg.; UIM; APH-5144 • 1♀; Chiricahua Mountains, Cave Creek Canyon, Cathedral Vista Point area; 31.88434°N, 109.17143°W^1^; 1634 m; 30 Aug. 2023; Chris A. Hamilton, Leonardo Chávez & Wyatt Mendez leg.; UIM; APH-5400.

##### Distribution and natural history.

*Aphonopelmachiricahua* is endemic to the Chiricahua Mountains (Figs 10, 11) in southeastern Arizona where it has been encountered in mid-elevation Madrean evergreen oak woodlands and pine-oak woodlands (Fig. 10a–c; ~ 1500–1720 m). Hamilton et al. (2016) noted that very little was known about the natural history of *A.chiricahua*. At the time, we had never observed this species in the field despite having spent hundreds of person-hours searching for it during the preceding decade. The lack of observations prompted us to hypothesize that “these spiders probably seek refuge under rocks and rarely place silk around their burrow entrances” (Hamilton et al. 2016: 95, 97). We now know that this is not entirely the case. The burrows of two mature females, one found in June 2018 (APH-5050) as reported by Jacobi (2018) and the other found in August 2023 (APH-5400; Fig. 10d), were indeed covered by a dense mat of silk. Both burrows were found in exposed grassy areas among scattered woodland vegetation. Nevertheless, mature females remain incredibly difficult to find. We are unsure whether this is because females are rare, if they are simply exceptionally good at concealing their burrow entrances, or both. Immature specimens have been found under rocks (APH-4021), inside small burrows located along vertical banks (APH-5078), and inside human-made structures (APH-5010). Mature males are diurnally active in October, November, and perhaps early December (see https://www.facebook.com/watch/?v=785835144804065 for an observation tentatively attributed to this species that was shared by the staff at Chiricahua National Monument).

## ﻿Discussion

### ﻿Non-monophyly of MSI endemics in the Chiricahua Mountains

The phylogenetic trees presented in Figs 1, 2 revealed two interesting results. First, the COX1 and UCE datasets both indicate that *A.jacobii* sp. nov. and *A.marxi* form a strongly supported clade. These results were unexpected because prior studies (Hendrixson et al. 2015; Hamilton et al. 2016; Hendrixson 2019) showed that the Madrean-affiliated taxa (i.e., *A.bacadehuachi*, *A.catalina*, *A.chiricahua*, *A.madera*, and *A.peloncillo*) belonged to a clade exclusive of *A.marxi*, a denizen of the Colorado Plateau (albeit weakly supported in Hamilton et al. 2016). Furthermore, monophyly of the Madrean species has been corroborated by samples we have analyzed from a handful of other MSI ranges in Arizona and Mexico (unpublished data), so it was expected that newly discovered endemic species from the MSI might follow a similar pattern and group with the Madrean taxa. In hindsight, however, the close relationship between *A.jacobii* sp. nov. and *A.marxi* is not surprising because biotic affinities between the Colorado Plateau and Chiricahua Mountains are well established, especially for taxa found at higher elevations (e.g., Linsley et al. 1961; Bennett et al. 1996). The rather “distant” relationship between *A.jacobii* sp. nov. and *A.chiricahua* is interesting because it suggests that *A.jacobii* sp. nov. and *A.chiricahua* have assimilated into the woodland and forest biota of the Chiricahua Mountains independently (see Agnarsson et al. 2016 for a similar result in Madagascan *Anelosimus* spiders), rather than having diverged in situ, as has been inferred for other invertebrate lineages with limited vagility that are endemic to specific MSI ranges (e.g., Weaver et al. 2010; Jochim et al. 2020).

The other intriguing result is that *A.jacobii* sp. nov. is diphyletic and *A.marxi* is paraphyletic in the COX1 analysis (Fig. 1). Mitochondrial haplotypes from the *A.jacobii* sp. nov. Barfoot Park population are embedded within a clade that includes all sampled haplotypes of *A.marxi*, whereas the Onion Saddle and Cave Creek Canyon populations of *A.jacobii* sp. nov. form a strongly supported clade that is sister to the *A.marxi* + Barfoot Park clade. This is in striking contrast to the UCE analysis (Fig. 2) which recovered both species as reciprocally monophyletic. These cases of “species-level polyphyly” and “paraphyly” (i.e., gene-tree/species-tree incongruence) (see Funk and Omland 2003) are likely due to ancient admixture and asymmetric mitochondrial introgression (e.g., Horoiwa et al. 2021) between *A.marxi* and *A.jacobii* sp. nov., a phenomenon hypothesized to have occurred multiple times in *Aphonopelma* (Hamilton et al. 2016), including two other species from this region (*A.vorhiesi* and *A.chalcodes*). These two species are currently separated by c. 100 km of unsuitable habitat (i.e., arid valleys and hills consisting of semidesert grasslands and desert scrub), but cooler climates in the past may have permitted contact between them. Extensive coniferous woodlands persisted at lower elevations during the Pleistocene (Van Devender and Spaulding 1979) and could have provided habitat connectivity between the Chiricahua Mountains and the uplands of the Mogollon Rim and Colorado Plateau. Further sampling and population structure analyses will be necessary to address this matter (see Newton et al. 2020, 2023; Starrett et al. 2024).

### ﻿Tarantula biodiversity of the Chiricahua Mountains

The Chiricahua Mountains are located at the intersection of the southern Colorado Plateau and Rocky Mountains, northern Sierra Madre Occidental, eastern Sonoran Desert, and western Chihuahuan Desert. As such, the massif’s biota is extensively influenced by each of these ecoregions, resulting in a mosaic of diversity unlike that of any other region in the United States. Additionally, as part of the Madrean Archipelago, woodland and forest habitats in the Chiricahua Mountains are physically isolated from those of other MSI ranges, leading to the evolution of numerous short-range endemic species that are restricted to them. Taken together, these mountains are part of the Madrean pine-oak woodlands biodiversity hotspot (Mittermeier et al. 2004) and are among the most biodiverse regions in the United States (Coronado Planning Partnership 2008), so it is no surprise that tarantulas show similar patterns of high diversity.

The Chiricahua Mountains and surrounding foothills, canyons, and grasslands are home to the most diverse assemblage of tarantula species anywhere in the United States. Eight of the 30 (27%) described species have been documented from this region: two endemic species whose closest relatives have affinity to the Colorado Plateau and Sierra Madre Occidental (*A.jacobii* sp. nov. and *A.chiricahua*, respectively); one species with affinity to the Sonoran Desert (*A.chalcodes*); four species with affinity to the Chihuahuan Desert and associated grasslands (*A.gabeli*, *A.hentzi* (Girard, 1852), *A.parvum* Hamilton, Hendrixson & Bond, 2016, and *A.peloncillo*); and one species that is more broadly distributed throughout the Sonoran and Chihuahuan deserts (*A.vorhiesi*). Outside of the Madrean Archipelago, no more than three species of tarantulas inhabit any other region of comparable size in the United States (e.g., the Arizona Transition Zone near Payson; Hamilton et al. 2016). NOTE: Five species have been purportedly observed in the vicinity of Del Rio, Texas (Hamilton et al. 2016; also search https://www.inaturalist.org), but many of the records (in iNaturalist) need to be verified with physical and genetic vouchers.

### ﻿Conservation of tarantulas in the Chiricahua Mountains

As is the case with other MSI species (see Hamilton et al. 2016), it is difficult to fully assess the distribution and abundance—and therefore the conservation status—of *A.jacobii* sp. nov. and *A.chiricahua* because these tarantulas are challenging to find and many sections of the massif remain unsampled; the Chiricahua Mountains are the largest range in the U.S.-MSI region, and many locations are difficult to access due to their remoteness and rugged terrain. In addition, we lack basic life history, survivorship, and fecundity data for these spiders. However, despite being restricted to a single mountain range, which presents its own set of risks, these species are afforded some degree of protection due to the range’s remoteness, and management by the U.S. Forest Service (Coronado National Forest, Douglas Ranger District) and U.S. National Park Service (Chiricahua National Monument).

Some important threats facing the Chiricahua Mountains and these tarantulas include exurban development in the San Simon Valley and Portal area, destructive recreational activities (e.g., offroad vehicles, degradation of undeveloped campsites), fire (natural and human-caused) due to fuel accumulation and fire suppression, invasive species, and climate change (i.e., increasing temperatures and decreasing precipitation amounts and patterns) (Coronado Planning Partnership 2008). A key advantage that *A.jacobii* sp. nov. may have compared to other MSI species is that it can be found in a variety of plant communities spanning at least 1100 m in elevation. However, the high-elevation population near Barfoot Park may be the most vulnerable. Warming temperatures and climate change have impacted the MSI biota in significant ways (see Brusca et al. 2013; Wiens et al. 2019), and these conditions are expected to continue “pushing” MSI species higher in elevation for the foreseeable future (Yanahan and Moore 2019). Graham et al. (2020) showed that tarantulas (*A.marxi*) have responded to past climate change by moving into suitable habitats as they became available, but if the Barfoot Park population is locally adapted to cooler mountain-top conditions, they can only continue moving higher in elevation until they are “pushed off” (i.e., local extinction) as their suitable habitats and temperature preferences disappear (see also Wiens et al. 2019).

There is also some concern that *A.jacobii* sp. nov. will be quickly introduced into the tarantula pet trade. We are aware that commercial field collectors will likely use the information contained in this article as a “treasure map” (see Stuart et al. 2006) for exploiting these spiders. Following the publication of our monograph documenting the diversity and distribution of *Aphonopelma* in the United States (Hamilton et al. 2016), each of the MSI species we described in that publication (i.e., *A.catalina*, *A.chiricahua*, *A.madera*, and *A.peloncillo*)—as well as several other species we reported GPS coordinates for—have since appeared on various pet trade lists. Market demand for *A.jacobii* sp. nov. may likewise be driven by its striking colors (Fig. 3), novelty, and docile disposition (Marshall et al. 2022; Fukushima et al. 2023). The Barfoot Park population is also found near a popular location for smuggling twin-spotted rattlesnakes (Prival 2007; Coronado Planning Partnership 2008), so the ease of access to these spiders’ habitat by unethical collectors is concerning. Additional sampling and long-term monitoring across the Chiricahua Mountains are necessary to assess the impact of these various factors on *A.jacobii* sp. nov. and *A.chiricahua*.

## ﻿Conclusions

The discovery of *A.jacobii* sp. nov. in the Chiricahua Mountains is exciting and noteworthy because it documents the first known case of multiple short-range endemic tarantula species inhabiting a single MSI range. This species also adds to our knowledge of tarantulas distributed in the Madrean pine-oak woodlands biodiversity hotspot (Mittermeier et al. 2004) and shifts the way we must approach assessing this group’s diversity moving forward. Our prior work on the Madrean-affiliated taxa suggested that only one species inhabited each MSI range (Hendrixson et al. 2015; Hamilton et al. 2016; unpublished data for other MSI ranges), similar to what has been observed in co-distributed scorpions (Bryson et al. 2013; Ayrey 2020; Jochim et al. 2020; Myers and Ayrey 2021), but we must reconsider this hypothesis in light of these new findings by revisiting previously sampled ranges and putting significant effort into ranges that have not yet been sampled. Future work on this group must address these sampling gaps, especially if we are to document this threatened ecoregion’s tarantula biodiversity and gain further insight into the complex biogeographic history and conservation needs of these spiders. These results also further underscore the importance of applying integrative methods for identifying cryptic diversity and delimiting species in a group of spiders that has historically been referred to as a “nomenclatural and taxonomic nightmare” (Raven 1990: 126).

## Supplementary Material

XML Treatment for Aphonopelma

XML Treatment for Aphonopelma jacobii

XML Treatment for Aphonopelma chiricahua

## ﻿Additional information

### Author ORCIDs

Chris A. Hamilton https://orcid.org/0000-0001-7263-0755

Brent E. Hendrixson https://orcid.org/0000-0003-1759-6405

Karina Silvestre Bringas https://orcid.org/0009-0003-3485-2279

### Data availability

All of the data that support the findings of this study are available in the main text or Supplementary Information.

## Supplementary materials

Supplementary material 1 Comparative genetic vouchers examined Data type docx https://binary.pensoft.net/file/1115082This dataset is made available under the Open Database License (http://opendatacommons.org/licenses/odbl/1.0/). The Open Database License (ODbL) is a license agreement intended to allow users to freely share, modify, and use this Dataset while maintaining this same freedom for others, provided that the original source and author(s) are credited.Chris A. Hamilton, Brent E. Hendrixson, Karina Silvestre Bringas

Supplementary material 2 A list of all morphological vouchers examined in this study Data type xlsx https://binary.pensoft.net/file/1115083This dataset is made available under the Open Database License (http://opendatacommons.org/licenses/odbl/1.0/). The Open Database License (ODbL) is a license agreement intended to allow users to freely share, modify, and use this Dataset while maintaining this same freedom for others, provided that the original source and author(s) are credited.Chris A. Hamilton, Brent E. Hendrixson, Karina Silvestre Bringas

Supplementary material 3 GenBank and SRA accession numbers for all genetic vouchers examined in this study Data type xlsx https://binary.pensoft.net/file/1115084This dataset is made available under the Open Database License (http://opendatacommons.org/licenses/odbl/1.0/). The Open Database License (ODbL) is a license agreement intended to allow users to freely share, modify, and use this Dataset while maintaining this same freedom for others, provided that the original source and author(s) are credited.Chris A. Hamilton, Brent E. Hendrixson, Karina Silvestre Bringas

Supplementary material 4 Scatterplots of the first (PC1) and second (PC2) principal components based on measurements of mature male and female members of the *Marxi* species group Data type pdf https://binary.pensoft.net/file/1115085This dataset is made available under the Open Database License (http://opendatacommons.org/licenses/odbl/1.0/). The Open Database License (ODbL) is a license agreement intended to allow users to freely share, modify, and use this Dataset while maintaining this same freedom for others, provided that the original source and author(s) are credited.Chris A. Hamilton, Brent E. Hendrixson, Karina Silvestre Bringas

Supplementary material 5 Boxplots comparing various morphometric ratios based on measurements of mature male and female members of the *Marxi* species group Data type zip https://binary.pensoft.net/file/1115086This dataset is made available under the Open Database License (http://opendatacommons.org/licenses/odbl/1.0/). The Open Database License (ODbL) is a license agreement intended to allow users to freely share, modify, and use this Dataset while maintaining this same freedom for others, provided that the original source and author(s) are credited.Chris A. Hamilton, Brent E. Hendrixson, Karina Silvestre Bringas

Supplementary material 6 Variation in spermathecae of female *Aphonopelmajacobii* sp. nov. Data type jpg https://binary.pensoft.net/file/1115087This dataset is made available under the Open Database License (http://opendatacommons.org/licenses/odbl/1.0/). The Open Database License (ODbL) is a license agreement intended to allow users to freely share, modify, and use this Dataset while maintaining this same freedom for others, provided that the original source and author(s) are credited.Chris A. Hamilton, Brent E. Hendrixson, Karina Silvestre Bringas

Supplementary material 7 Morphology of *Aphonopelmachiricahua* (female, APH-5050) Data type jpg https://binary.pensoft.net/file/1115088This dataset is made available under the Open Database License (http://opendatacommons.org/licenses/odbl/1.0/). The Open Database License (ODbL) is a license agreement intended to allow users to freely share, modify, and use this Dataset while maintaining this same freedom for others, provided that the original source and author(s) are credited.Chris A. Hamilton, Brent E. Hendrixson, Karina Silvestre Bringas

Supplementary material 8 Phylogenomics files Data type zip https://binary.pensoft.net/file/1115089This dataset is made available under the Open Database License (http://opendatacommons.org/licenses/odbl/1.0/). The Open Database License (ODbL) is a license agreement intended to allow users to freely share, modify, and use this Dataset while maintaining this same freedom for others, provided that the original source and author(s) are credited.Chris A. Hamilton, Brent E. Hendrixson, Karina Silvestre Bringas

Supplementary material 9 Measurements for *Aphonopelmachiricahua* and *A.jacobii* specimens Data type txt https://binary.pensoft.net/file/1115090This dataset is made available under the Open Database License (http://opendatacommons.org/licenses/odbl/1.0/). The Open Database License (ODbL) is a license agreement intended to allow users to freely share, modify, and use this Dataset while maintaining this same freedom for others, provided that the original source and author(s) are credited.Chris A. Hamilton, Brent E. Hendrixson, Karina Silvestre Bringas

Supplementary material 10 Specimen locality information for *Aphonopelmachiricahua* and *A.jacobii* specimens Data type xlsx https://binary.pensoft.net/file/1115091This dataset is made available under the Open Database License (http://opendatacommons.org/licenses/odbl/1.0/). The Open Database License (ODbL) is a license agreement intended to allow users to freely share, modify, and use this Dataset while maintaining this same freedom for others, provided that the original source and author(s) are credited.Chris A. Hamilton, Brent E. Hendrixson, Karina Silvestre Bringas
