# Supplementary material for: ﻿Discovery of a new tarantula species from the Madrean Sky Islands and the first documented instance of syntopy between two montane endemics (Araneae, Theraphosidae, Aphonopelma): a case of prior mistaken identity
Source: Zookeys. 2024 Aug 16;1210:61–98. doi: 10.3897/zookeys.1210.125318 (PMC11344175; doi:10.3897/zookeys.1210.125318)
Supplement: Supplementary material 1 — Comparative genetic vouchers examined [file zookeys-1210-061_article-125318__-s001.docx]

**S1: Comparative Genetic Vouchers Examined**

*Museum Repositories*

- American Museum of Natural History, New York, New York (**AMNH**)
- University of Idaho William F. Barr Entomological Museum, Moscow, Idaho (**UIM**)

***Aphonopelma bacadehuachi* Hendrixson 2019**

**MEXICO: SONORA: *Municipio de Bacadéhuachi:*** Sierra de Bacadéhuachi, Rincón de Guadalupe, 14.9 km (by air) ENE of Bacadéhuachi, Arroyo Campo los Padres (Rio

Riito drainage) (N29.84806°, W108.99417°, 1800 m)^1^, 2.viii.2011, leg. Thomas R. Van Devender,♂ holotype (APH-1357),♀ paratype (APH-1356), UIM.

***Aphonopelma catalina* Hamilton, Hendrixson & Bond 2016**

**UNITED STATES: ARIZONA: *Pima County:*** Santa Catalina Mountains, Bug Spring Trail (N32.34544°, W110.71602°, 1603 m)^4^, 28.xii.2008, leg. Paul E. Marek & Charity Hall, 1♂ (APH-0454), UIM; Santa Catalina Mountains, Bug Spring Trail (N32.34544°, W110.71602°, 1603 m)^4^, 11.xii.2011, leg. Jillian Cowles & Bill Savary, 1♂ (APH-1438), UIM; Santa Catalina Mountains, Bug Spring Trail (N32.34544°, W110.71602°, 1603 m)^1^, 17.xii.2011, leg. Brent E. Hendrixson & Thomas Martin,♂ holotype (APH-1440), UIM,♂ paratype (APH-1439), AMNH; Santa Catalina Mountains, Bug Spring Trail (N32.34544°, W110.71602°, 1603 m)^1^, 9.xi.2012, leg. Brent E. Hendrixson,♀ paratype (APH-1602), UIM; Santa Catalina Mountains, along Mount Lemmon Road (N32.47536°, W110.72505°, 1665 m)^1^, 8.xi.2018, leg. Tim Burkhardt & Adam Noel, 1♀ (APH-4035), UIM; Santa Catalina Mountains, along Mount Lemmon Road (N32.47493°, W110.72531°, 1677 m)^1^, 1.xi.2019, leg. Chris A. Hamilton, Brent E. Hendrixson & Wyatt Mendez, 1 imm. (APH-4038), UIM; Santa Catalina Mountains, along Mount Lemmon Road (N32.47536°, W110.72505°, 1665 m)^1^, 1.xi.2019, leg. Brent E. Hendrixson, Chris A. Hamilton & Wyatt Mendez, 1 imm. (APH-4039), UIM; Santa Catalina Mountains, Bug Spring Trail (N32.34645°, W110.71540°, 1615 m)^1^, 3.xii.2018, leg. Brent E. Hendrixson, Michael A. Jacobi, Paul Kaufman & Karla Kaufman, 1♂ (APH-5042), UIM; Santa Catalina Mountains, Bug Spring Trail (N32.34638°, W110.71552°, 1615 m)^1^, 3.xii.2018, leg. Brent E. Hendrixson, Michael A. Jacobi, Paul Kaufman & Karla Kaufman, 1♂ (APH-5043), UIM; Santa Catalina Mountains, Bug Spring Trail (N32.34977°, W110.71127°, 1662 m)^1^, 6.xii.2018, leg. Brent E. Hendrixson & Michael A. Jacobi, 1♂ 1♀ (APH-5046, APH-5047), UIM; Rincon Mountains, Miller Canyon (N32.15327°, W110.48689°, 1296 m)^1^, 9.xi.2019, leg. Chris McCreedy, 1♂ (APH-5074), UIM.

*Remarks:* We report the first record of *A. catalina* from the Rincon Mountains (APH-5074). *Aphonopelma catalina* is only the second MSI species documented from more than a single mountain range (the other species is *A. madera* from the Santa Rita, Huachuca, and Pajarito Mountains; see Hendrixson et al. 2015 and Hamilton et al. 2016).

***Aphonopelma madera* Hamilton, Hendrixson, Bond 2016**

**UNITED STATES: ARIZONA: *Cochise County:*** Huachuca Mountains, Ash Canyon (N31.38339°, W110.24486°, 1612 m)^4^, xi.2010 (day not available), leg. Jim Murray, 1♂ (APH-1249), UIM; Huachuca Mountains, along Garden Canyon Road (N31.47306°, W110.35111°, 1637 m)^1^, 4.xii.2010, no collector name available, 2♂ (APH-1250, APH-1251); UIM; Huachuca Mountains, along Carr Canyon Road (N31.44966°, W110.28203°, 1590 m)^1^, 8.xi.2012, leg. Brent E. Hendrixson, 1♂ (APH-1595), UIM; Huachuca Mountains, Ramsey Vista Campground (N31.42933°, W110.30394°, 2252 m)^1^, 28.viii.2018, leg. Brent E. Hendrixson, 1♂ (APH-5053), UIM; Huachuca Mountains, Miller Canyon (N31.42456°, W110.26091°, 1596 m)^1^, 29.viii.2018, leg. Brent E. Hendrixson, 1♂ (APH-5054), UIM. ***Pima County:*** Santa Rita Mountains, Madera Canyon, Madera Picnic Area (N31.72695°, W110.88040°, 1480 m)^1^, 10.vii.2009, leg. Brent E. Hendrixson, Jon Davenport & Nate Davis, 1 imm. (APH-0618), UIM; 1 km N Santa Cruz County line along Madera Canyon Road (N31.73588°, W110.88232°, 1402 m)^1^, 8.xi.2010, leg. June Olberding, 1♂ (APH-1434), UIM; Santa Rita Mountains, Madera Canyon, Bog Springs Campground (N31.72733°, W110.87502°, 1540 m)^1^, 27.x.2012, leg. Brent E. Hendrixson,♀ paratype (APH-1571), AMNH; Santa Rita Mountains, Madera Canyon, along Madera Canyon Road (N31.72891°, W110.88046°, 1467 m)^1^, 15.xi.2012, leg. Brent E. Hendrixson, 1♀ (APH-1197), UIM; Santa Rita Mountains, Madera Canyon, Madera Picnic Area (N31.72722°, W110.88081°, 1474 m)^1^, 15.xi.2012, leg. Brent E. Hendrixson, 1♀ (APH-1625), UIM; Santa Rita Mountains, Madera Canyon, Bog Springs Campground (N31.72733°, W110.87502°, 1540 m)^1^, 15.xi.2012, leg. Brent E. Hendrixson, 1♂ (APH-1630), UIM; Santa Rita Mountains, Madera Canyon, Whitehouse Picnic Area (N31.73340°, W110.88249°, 1423 m)^1^, 15.xi.2012, leg. Brent E. Hendrixson, 1♂ (APH-1631), UIM; Santa Rita Mountains, Madera Canyon, along road to Bog Springs Campground (N31.72812°, W110.87882°, 1492 m)^1^, 12.xi.2013, leg. Chris A. Hamilton & Brent E. Hendrixson,♂ holotype (APH-3177), UIM. ***Santa Cruz County:*** Pajarito Mountains, specific locality not available, date of collection not available, leg. David Kandeyeli, 1♀ (APH-0136); Santa Rita Mountains, along Mount Hopkins Road (N31.67632°, W110.88341°, 2100 m)^1^, 28.vii.2010, leg. Brent E. Hendrixson, Brendon Barnes & Nate Davis, 1 imm. (APH-1197), UIM; Patagonia, along Pennsylvania Avenue (N31.54024°, W110.75853°, 1232 m)^1^, 18.xii.2011, leg. Brent E. Hendrixson & Thomas Martin, 1♂ (APH-1442), UIM; Pajarito Mountains, Walker Canyon (N31.36871°, W111.06826°, 1259 m)^1^, 1.ii.2019, leg. Tim Burkhardt, 1♀ (APH-4036), UIM; Pajarito Mountains, Walker Canyon (N31.36871°, W111.06826°, 1259 m)^1^, 25.viii.2019, leg. Adam Noel, 1♂ (APH-4037), UIM; Santa Rita Mountains, Upper Madera Canyon (N31.71345°, W110.87442°, 1644 m)^1^, 2.x.2018, leg. Brent E. Hendrixson, 1♀ (APH-5055), UIM.

*Remarks:* Three sexually mature males (APH-4037, APH-5053, APH-5054) were found in late August 2018 and 2019, indicating that the breeding period for this species begins earlier than previously understood by Hendrixson et al. (2015) and Hamilton et al. (2016). We still characterize *A. madera* as a “fall breeder” but acknowledge that its breeding period begins in late summer. One of these males (APH-5053) was found in pine forest above 2250 m in the Huachuca Mountains. This observation represents the highest reported elevation for this species, surpassing the previous record of a specimen (APH-1197) found in the Santa Rita Mountains by c. 150 m.

***Aphonopelma marxi* (Simon 1891)**

**UNITED STATES: ARIZONA: *Coconino County:*** Mogollon Rim, along Highway-260 E of Payson (N34.29877°, W110.86005°, 2300 m)^4^, date of collection not available, leg. Brandon Anderson, 1 imm. (APH-0171), UIM; 10.8 km SE Highway-64 along Highway-180 (N35.60450°, W112.04095°, 1874 m)^1^, 7.x.2009, leg. Brent E. Hendrixson & Thomas Martin, 1♂ (APH-0772), UIM; Grand Canyon National Park, South Rim Trail (N36.06057°, W112.12431°, 2083 m)^1^, 14.x.2011, leg. Brent E. Hendrixson & Krissy E. Rehm, 1♂ (APH-1425), UIM. ***Gila County:*** Tonto National Forest, Pinal Mountains, near Pioneer Pass (N33.28911°, W110.79381°, 1709 m)^1^, 31.x.2019, leg. Brent E. Hendrixson & Chris A. Hamilton, 1♂ 1♀ (APH-4012, APH-4013), UIM. **COLORADO: *Montrose County:*** Nucla (N38.26991°, W108.54767°, 1768 m)^4^, 9.x.2004, leg. Peggy Case, 1♂ (APH-0008), UIM. **NEW MEXICO: *Cibola County:*** El Morro National Monument, along Highway-53 (N35.04339°, W108.34357°, 2185 m)^1^, 9.x.2011, leg. Brent E. Hendrixson & Thomas Martin, 1♂ (APH-1410), UIM. ***Grant County:*** Gila National Forest, along Highway-15 north of Pinos Altos near Cherry Creek Campground (N32.91478°, W108.22472°, 2078 m)^1^, 30.ix.2018, leg. Brent E. Hendrixson, 1♀ (APH-5051), UIM. ***Rio Arriba County:*** 4.5 km S El Rito along Highway-554 (N36.304638°, W106.18364°, 2039 m)^1^, 6.x.2012, leg. Brent E. Hendrixson, 1♂ (APH-1535), UIM.

*Remarks:* We report the first records of *A. marxi* from the Pinal Mountains in central Arizona (APH-4012, APH-4013) and Pinos Altos in southwestern New Mexico (APH-5051). These collection sites represent the southernmost records for this species in Arizona and New Mexico, respectively.

***Aphonopelma peloncillo* Hamilton, Hendrixson, Bond 2016**

**UNITED STATES: ARIZONA: *Cochise County:*** 50 km SW New Mexico state line along Highway-80 (N31.46999°, W109.35916°, 1327 m)^1^, 18.viii.2009, leg. Alice Abela, 1♂ (APH-0723), UIM. **NEW MEXICO:** ***Hidalgo County:*** Peloncillo Mountains, Clanton Draw area (N31.52238°, W108.98037°, 1653 m)^1^, 14.vii.2009, leg. Brent E. Hendrixson & Nate Davis, 1 imm. (APH-0667), UIM; 1.3 km SW Highway-338/County Road C001 along County Road C004 (N31.54450°, W108.88201°, 1557 m)^1^, 14.vii.2009, leg. Brent E. Hendrixson & Nate Davis, 1♂ (APH-0670), UIM; 6 km N C004 along Highway-338/County Road C001 (N31.60708°, W108.86708°, 1512 m)^1^, 14.vii.2009, leg. Brent E. Hendrixson & Nate Davis,♂ holotype (APH-0672), UIM; 6 km N C004 along Highway-338/County Road C001 (N31.60708°, W108.86708°, 1512 m)^1^, 14.vii.2009, leg. Brent E. Hendrixson & Nate Davis, 1♂ (APH-0673), UIM; Peloncillo Mountains, Clanton Draw area (N31.52238°, W108.98037°, 1653 m)^1^, 16.vii.2009, leg. Brent E. Hendrixson & Nate Davis, 2♀ (APH-0681, APH-0683), UIM; Peloncillo Mountains, Coronado National Forest (N31.51867°, W108.98260°, 1653 m)^1^, 26.vii.2010, leg. Brent E. Hendrixson, Brendon Barnes & Nate Davis,♀ paratype (APH-1181), AMNH; Peloncillo Mountains, Coronado National Forest (N31.51867°, W108.98260°, 1653 m)^1^, 26.vii.2010, leg. Brent E. Hendrixson, Brendon Barnes & Nate Davis, 1♀ (APH-1182), UIM; 11.4 km N C004 along Highway-338/County Road C001 (N31.65237°, W108.84711°, 1491 m)^1^, 26.vii.2010, leg. Brent E. Hendrixson, Brendon Barnes & Nate Davis,♂ paratype (APH-1190), AMNH; 11.4 km N C004 along Highway-338/County Road C001 (N31.65237°, W108.84711°, 1491 m)^1^, 26.vii.2010, leg. Brent E. Hendrixson, Brendon Barnes & Nate Davis, 1♂ (APH-1191), UIM; 4 km SW Highway-338/County Road C001 along County Road C004 (N31.52871°, W108.90033°, 1593 m)^1^, 8.ix.2012, leg. Brent E. Hendrixson, 1♀ (APH-1516), UIM.

***Aphonopelma vorhiesi* (Chamberlin & Ivie 1939)**

**UNITED STATES: ARIZONA: *Graham County:*** Tanque Road near Highway-191 (N32.60413°, W109.68170°, 1185 m)^1^, 11.vii.2009, leg. Brent E. Hendrixson & Nate Davis, 1 imm. (APH-0624), UIM. ***Pima County:*** Tucson, 995 E Arbab Court (N32.11585°, W110.79889°, 885 m)^2^, 29.viii.2007, leg. Sandi Sowers, 1♂ (APH-0177), UIM; N of Tucson just past Highway-77 split along Highway-79 (N32.57446°, W110.94732°, 1047 m)^1^, 14.xi.2013, leg. Chris A. Hamilton & Brent E. Hendrixson, 1♀ (APH-3188), UIM. ***Santa Cruz County:*** Patagonia Mountains, along Harshaw Road (N31.52809°, W110.71141°, 1284 m)^1^, 9.ix.2007, leg. Manny Rubio, 1♂ (APH-0185), UIM; Patagonia Mountains, along Harshaw Road (N31.53125°, W110.71818°, 1279 m)^1^, viii.2007 (day not available), leg. Manny Rubio, 1♂ (APH-0186), UIM; c. 6.4 km S Peña Blanca Lake, 24.vii.2009, leg. Paul Bollinger, 1 imm. (APH-0717), UIM. **NEW MEXICO: *Doña Ana County:*** Organ Mountains, Aguirre Springs Road (N32.39919°, W106.54844°, 1507 m)^1^, 13.vii.2009, leg. Brent E. Hendrixson & Nate Davis, 1♂ (APH-0656), UIM. ***Hidalgo County:*** 3.7 km S I-10 along Hwy-80 (N32.20438°, W108.94972°, 1293 m)^1^, 15.vii.2009, leg. Brent E. Hendrixson & Nate Davis, 1♀ (APH-0674), UIM.

***Aphonopelma* sp. (undetermined)**

**UNITED STATES: ARIZONA: *Cochise County:*** Cochise Cemetery along Highway-191 near Willcox Playa (N32.09383°, W109.91015°, 1279 m)^1^, 11.vii.2009, leg. Brent E. Hendrixson & Nate Davis, 1♀ (APH-0622), UIM; Huachuca Mountains, Copper Canyon, 2007 (month and day not available), leg. Josh Richards, 1♀ (APH-0880), UIM.
