# Supplementary figures and images for: ﻿Discovery of a new tarantula species from the Madrean Sky Islands and the first documented instance of syntopy between two montane endemics (Araneae, Theraphosidae, Aphonopelma): a case of prior mistaken identity
Source: Zookeys. 2024 Aug 16;1210:61–98. doi: 10.3897/zookeys.1210.125318 (PMC11344175; doi:10.3897/zookeys.1210.125318)

A

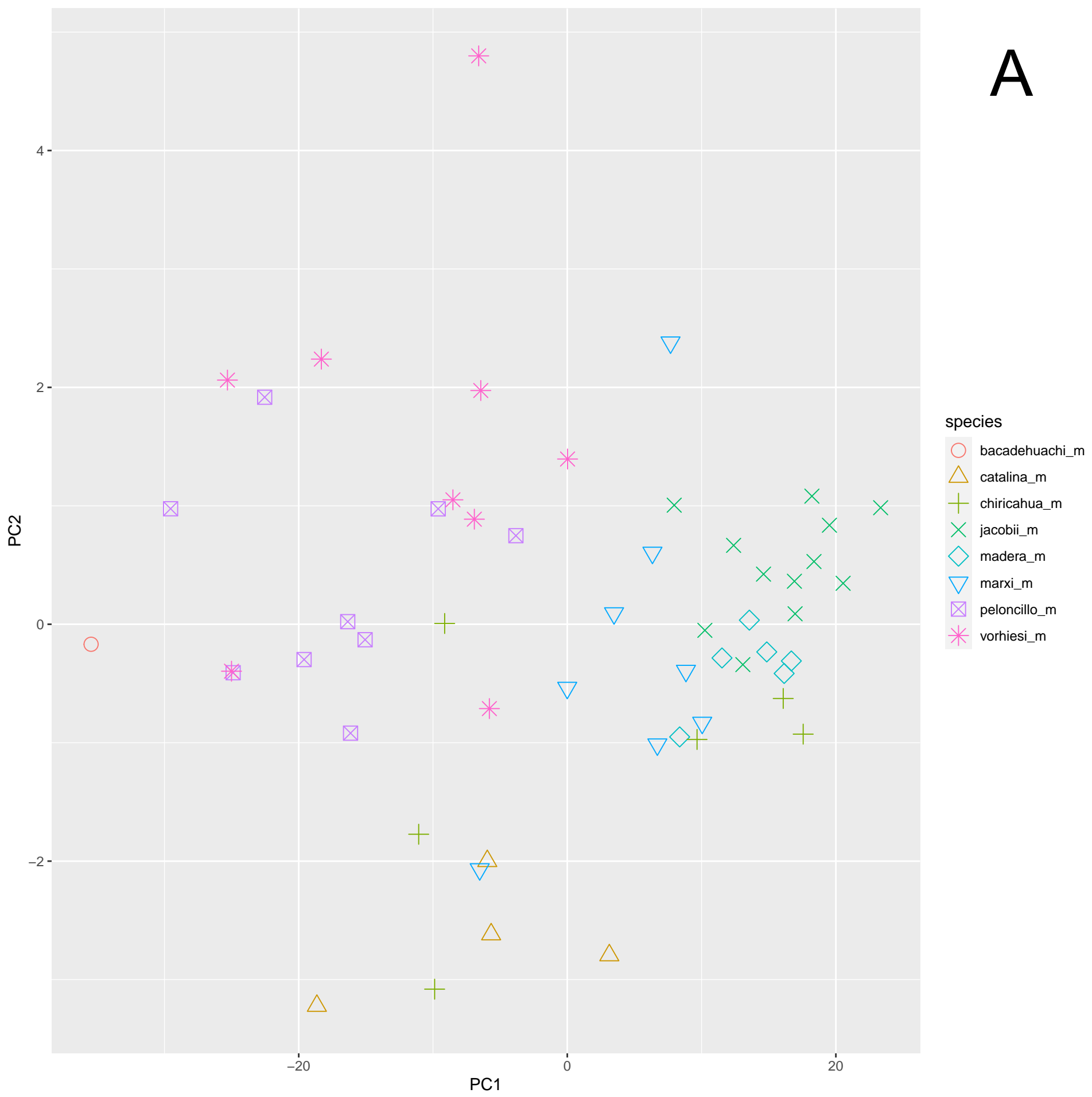

B

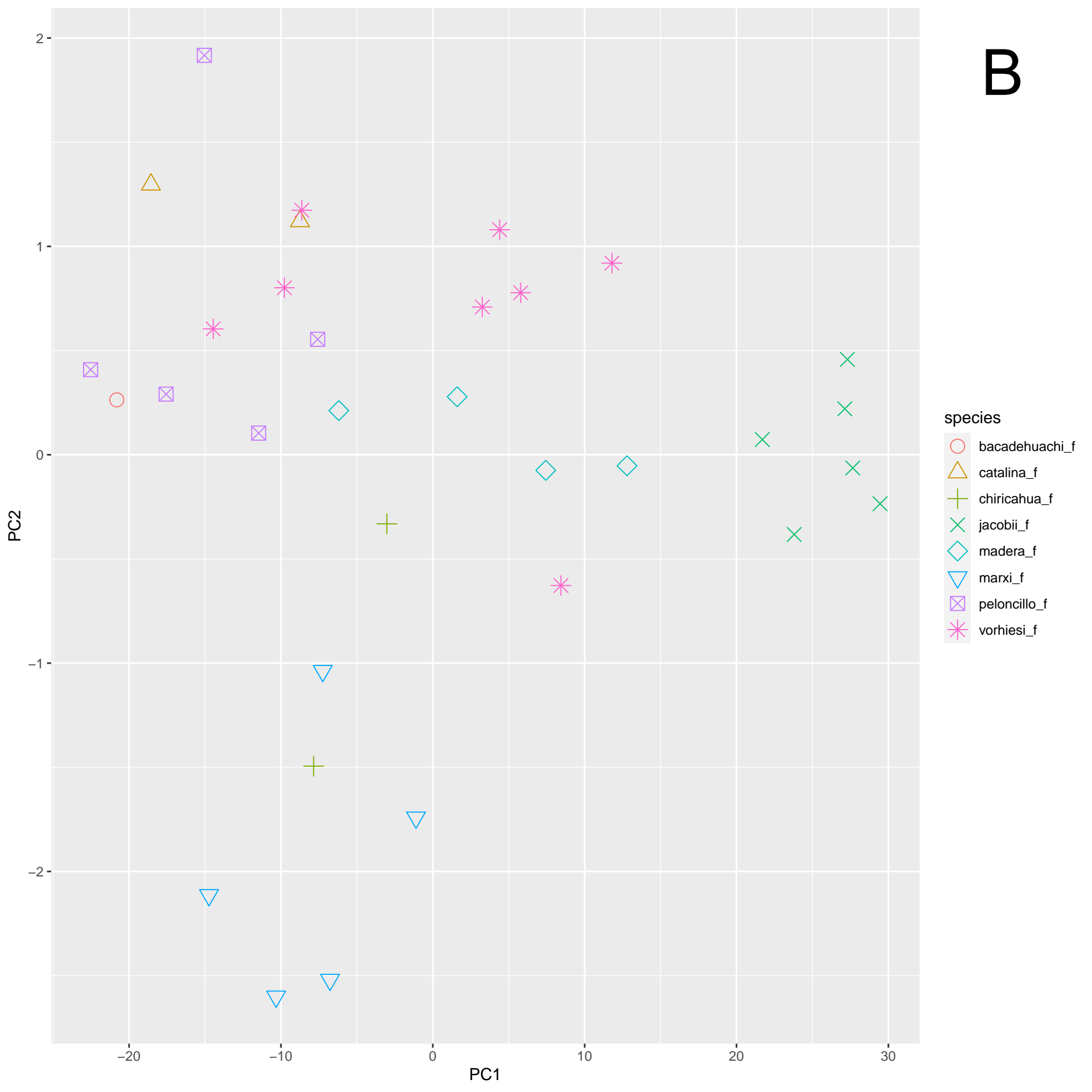

Supplement: Supplementary material 4 — Scatterplots of the first (PC1) and second (PC2) principal components based on measurements of mature male and female members of the Marxi species group [file zookeys-1210-061_article-125318__-s004.pdf]

Marxi group species

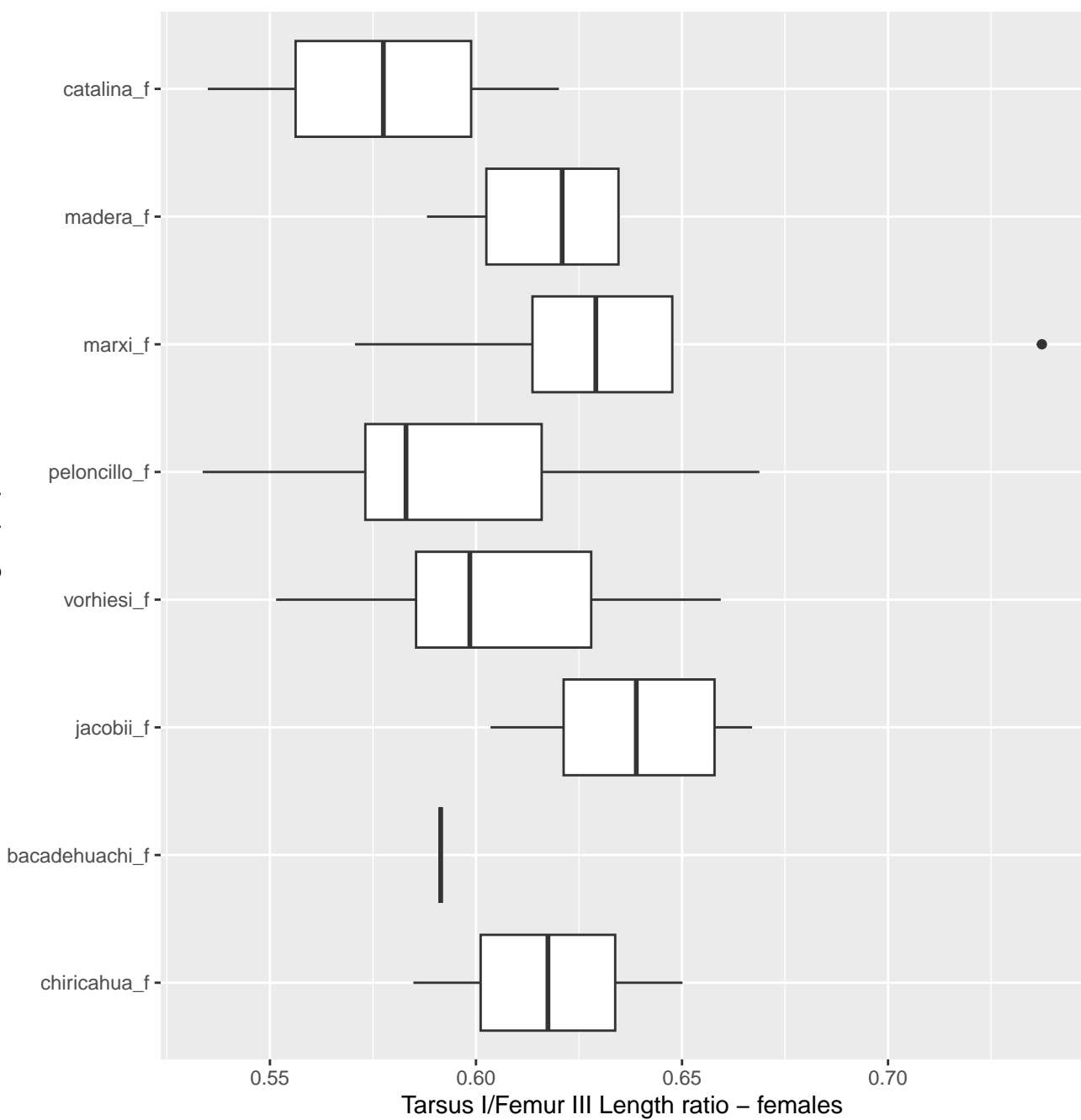

Supplement: Supplementary material 5 — Boxplots comparing various morphometric ratios based on measurements of mature male and female members of the Marxi species group [file zookeys-1210-061_article-125318__-s005.zip › Suppl5/Marxi_group_females/PDFs/A1F3_ratio_females.pdf]

Marxi group species

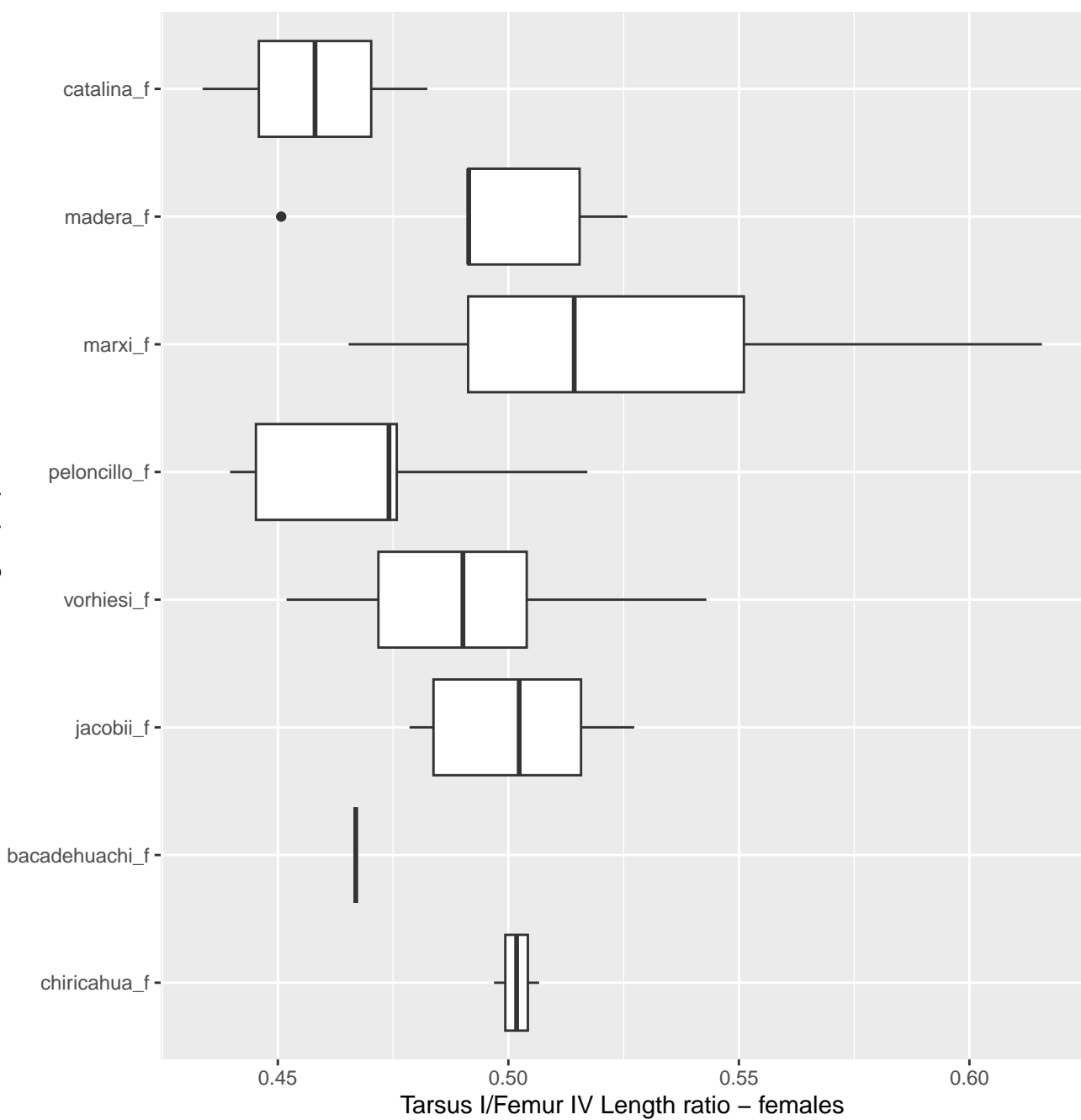

Supplement: Supplementary material 5 — Boxplots comparing various morphometric ratios based on measurements of mature male and female members of the Marxi species group [file zookeys-1210-061_article-125318__-s005.zip › Suppl5/Marxi_group_females/PDFs/A1F4_ratio_females.pdf]

Marxi group species

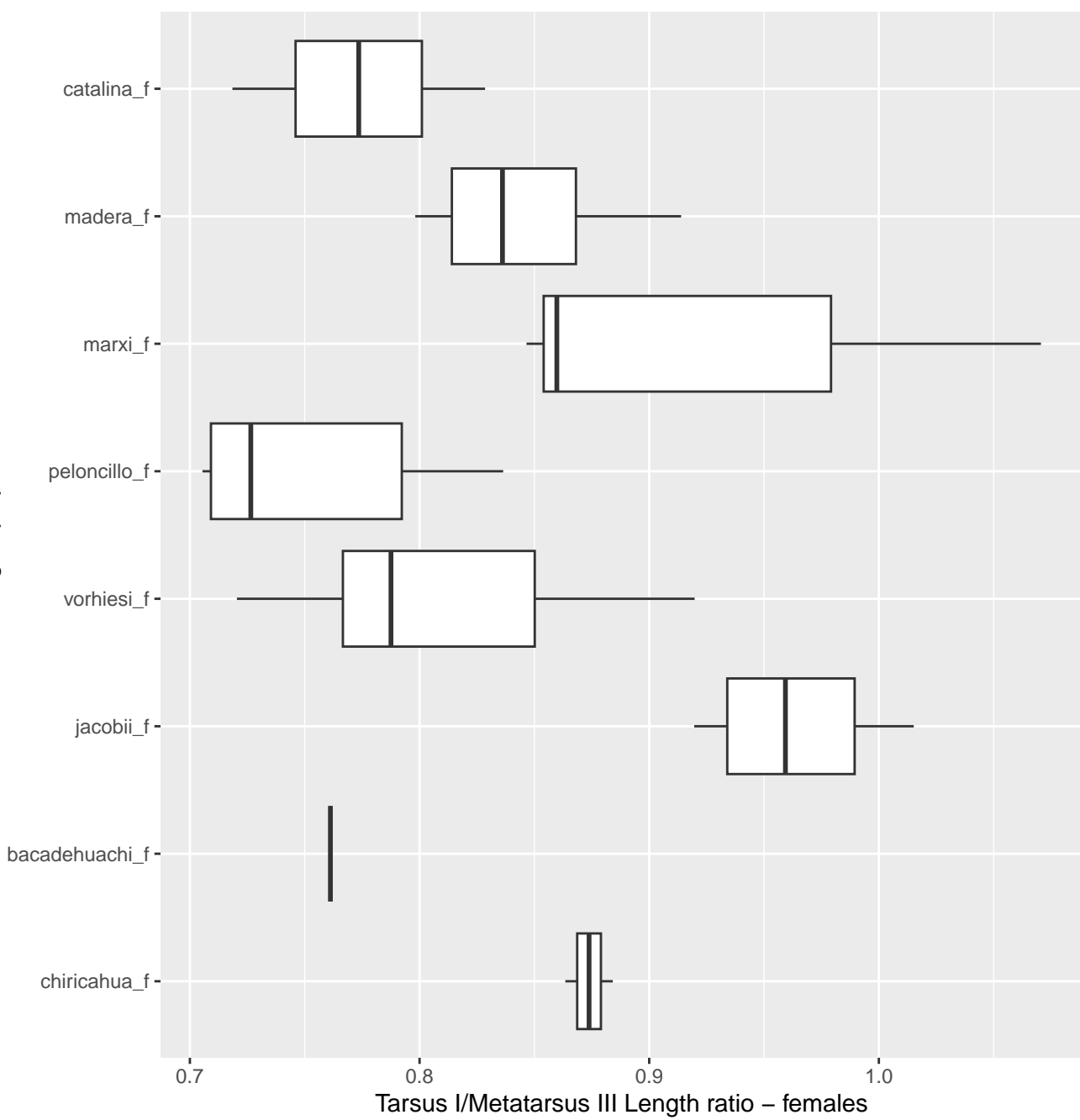

Supplement: Supplementary material 5 — Boxplots comparing various morphometric ratios based on measurements of mature male and female members of the Marxi species group [file zookeys-1210-061_article-125318__-s005.zip › Suppl5/Marxi_group_females/PDFs/A1M3_ratio_females.pdf]

Marxi group species

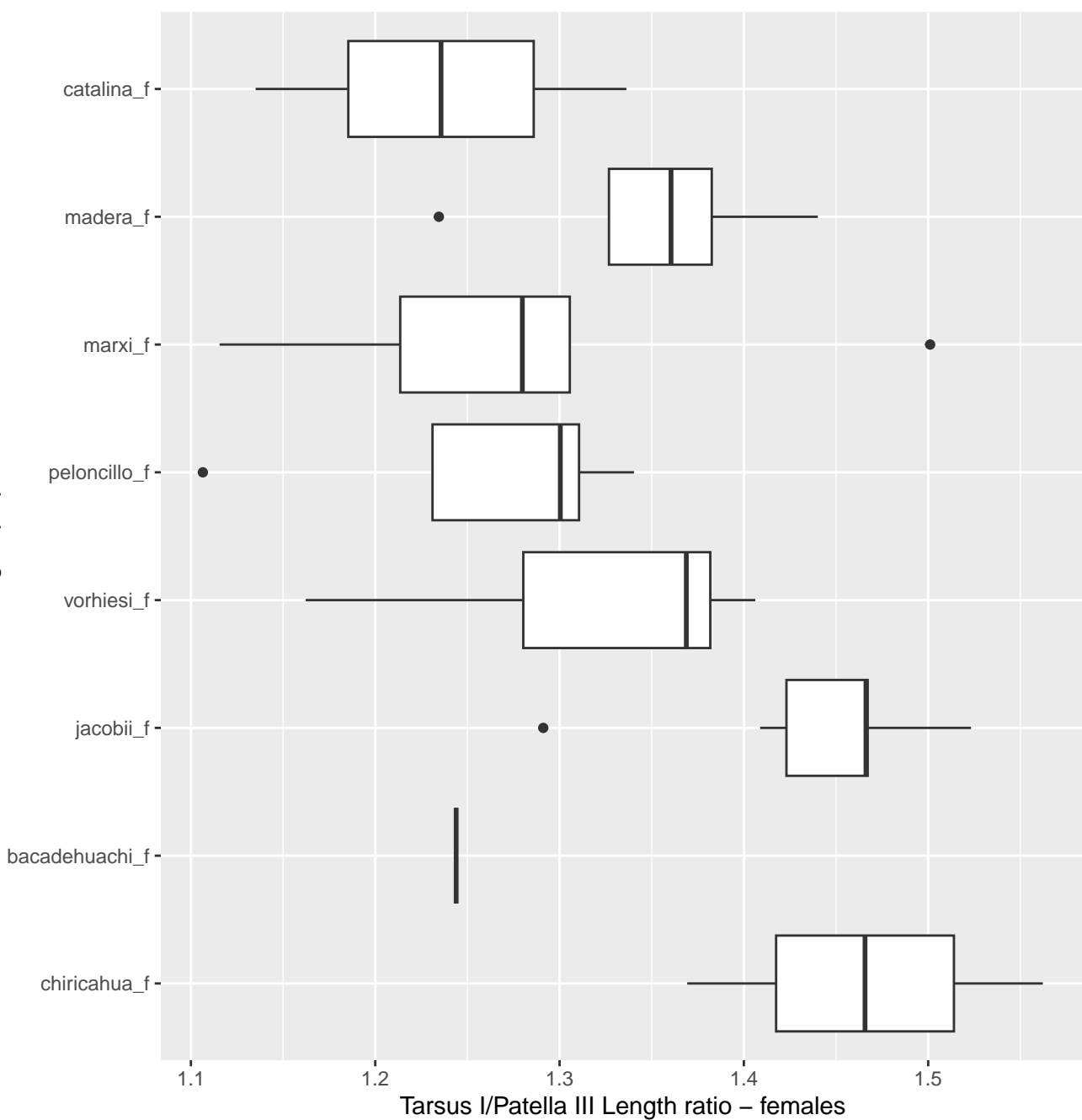

Supplement: Supplementary material 5 — Boxplots comparing various morphometric ratios based on measurements of mature male and female members of the Marxi species group [file zookeys-1210-061_article-125318__-s005.zip › Suppl5/Marxi_group_females/PDFs/A1P3_ratio_females.pdf]

Marxi group species

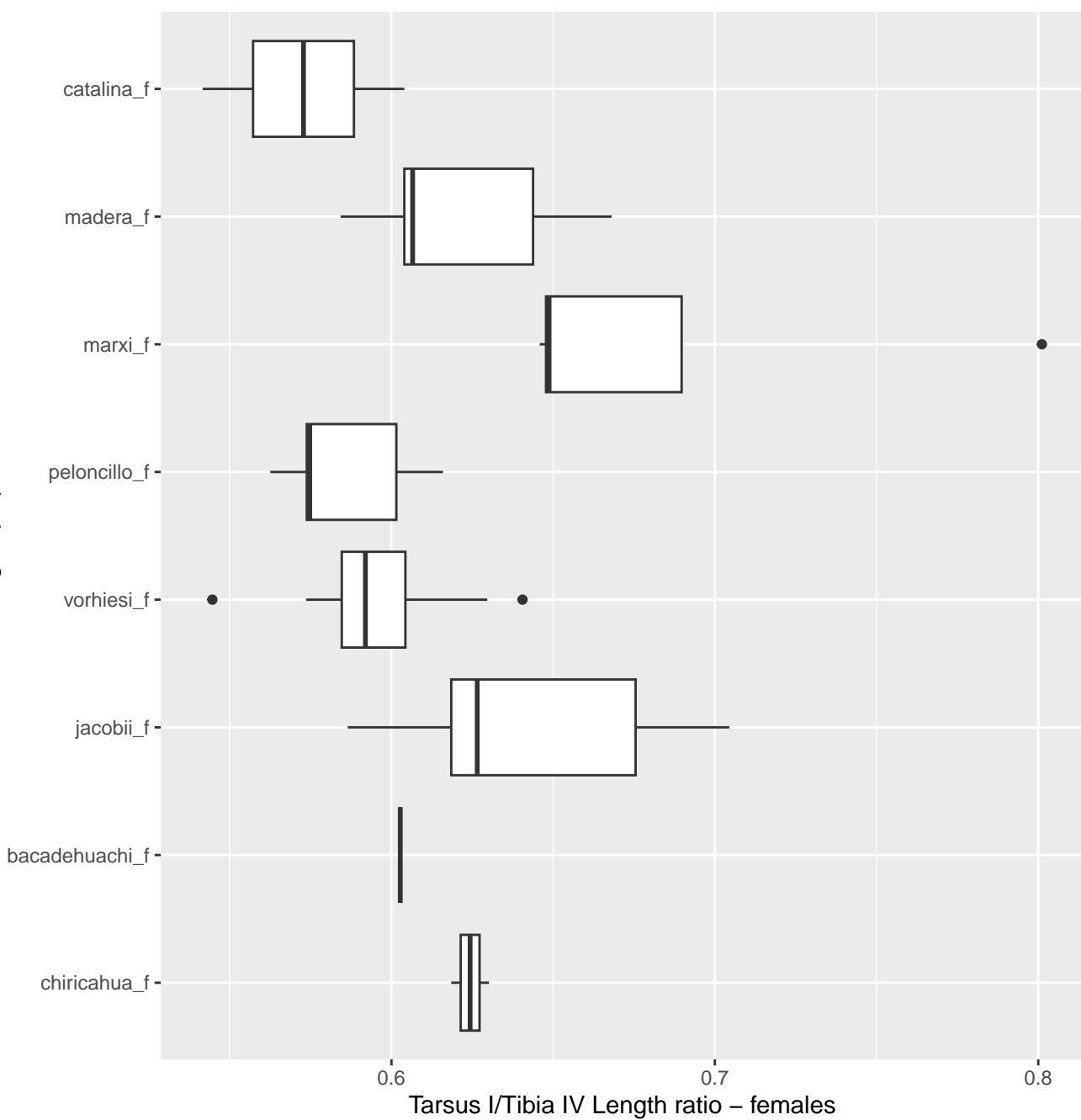

Supplement: Supplementary material 5 — Boxplots comparing various morphometric ratios based on measurements of mature male and female members of the Marxi species group [file zookeys-1210-061_article-125318__-s005.zip › Suppl5/Marxi_group_females/PDFs/A1T4_ratio_females.pdf]

Marxi group species

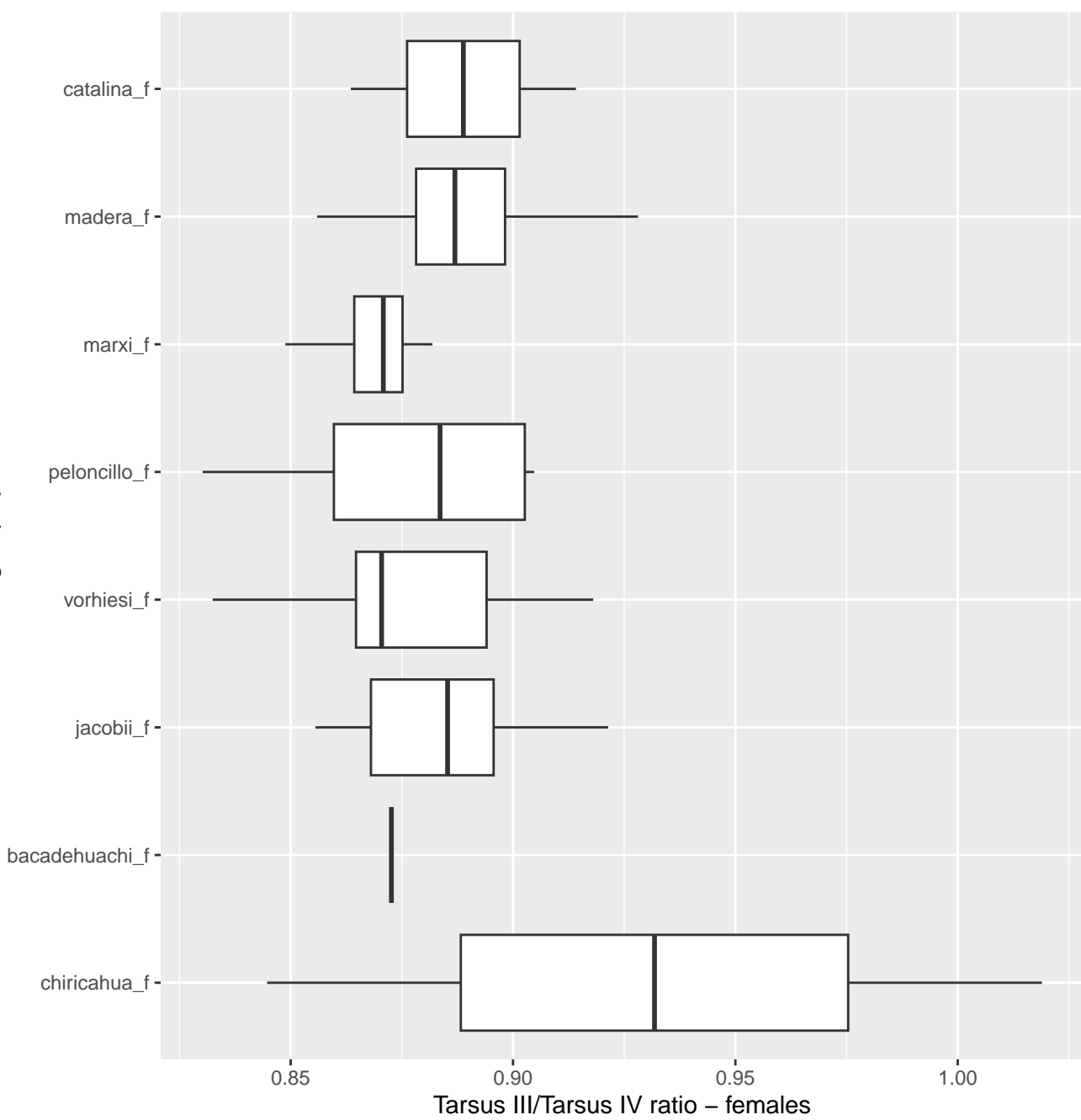

Supplement: Supplementary material 5 — Boxplots comparing various morphometric ratios based on measurements of mature male and female members of the Marxi species group [file zookeys-1210-061_article-125318__-s005.zip › Suppl5/Marxi_group_females/PDFs/A3A4_ratio_females.pdf]

Marxi group species

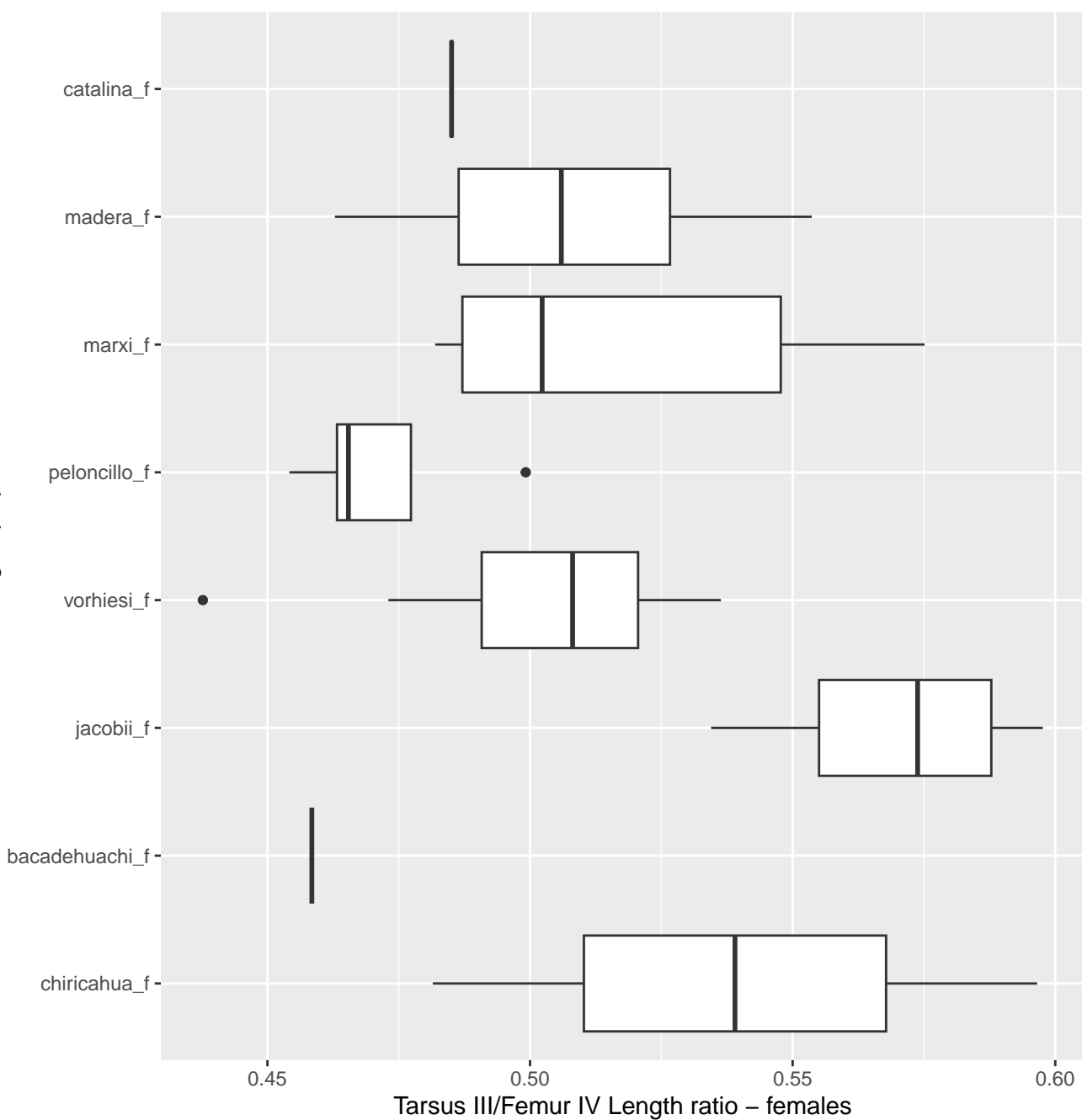

Supplement: Supplementary material 5 — Boxplots comparing various morphometric ratios based on measurements of mature male and female members of the Marxi species group [file zookeys-1210-061_article-125318__-s005.zip › Suppl5/Marxi_group_females/PDFs/A3F4_ratio_females.pdf]

Marxi group species

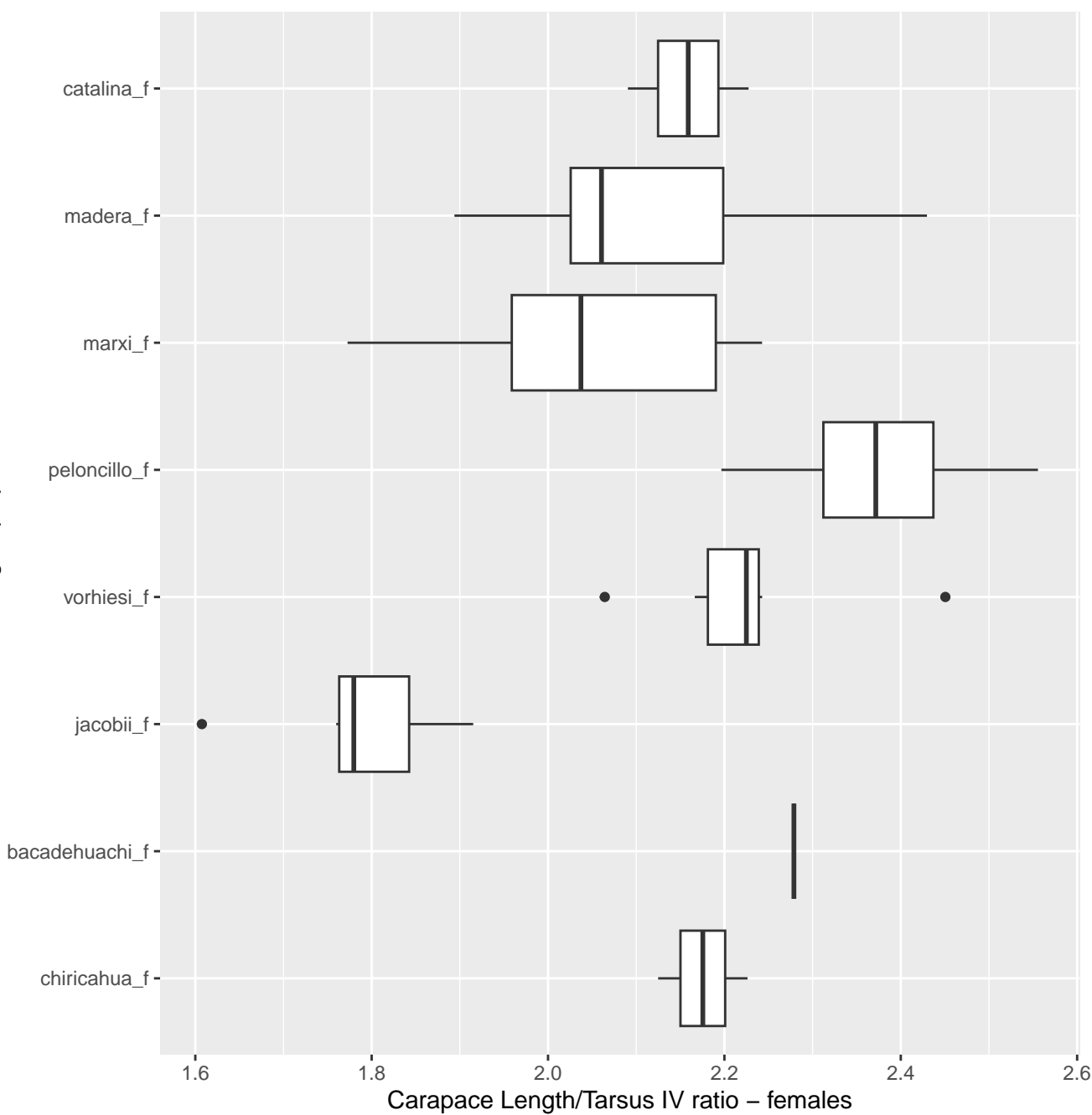

Supplement: Supplementary material 5 — Boxplots comparing various morphometric ratios based on measurements of mature male and female members of the Marxi species group [file zookeys-1210-061_article-125318__-s005.zip › Suppl5/Marxi_group_females/PDFs/ClA4_ratio_females.pdf]

Marxi group species

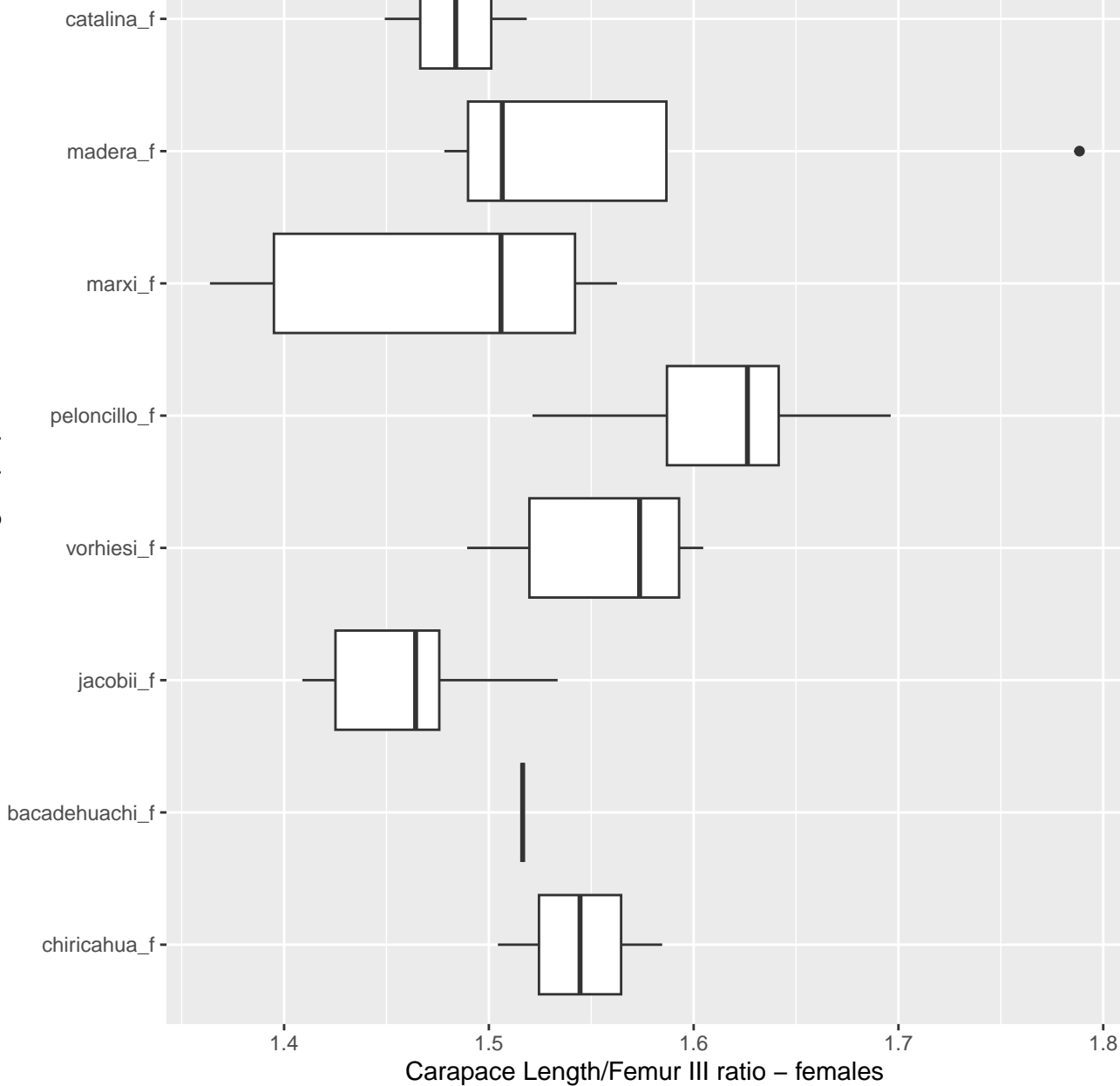

Supplement: Supplementary material 5 — Boxplots comparing various morphometric ratios based on measurements of mature male and female members of the Marxi species group [file zookeys-1210-061_article-125318__-s005.zip › Suppl5/Marxi_group_females/PDFs/ClF3_ratio_females.pdf]

Marxi group species

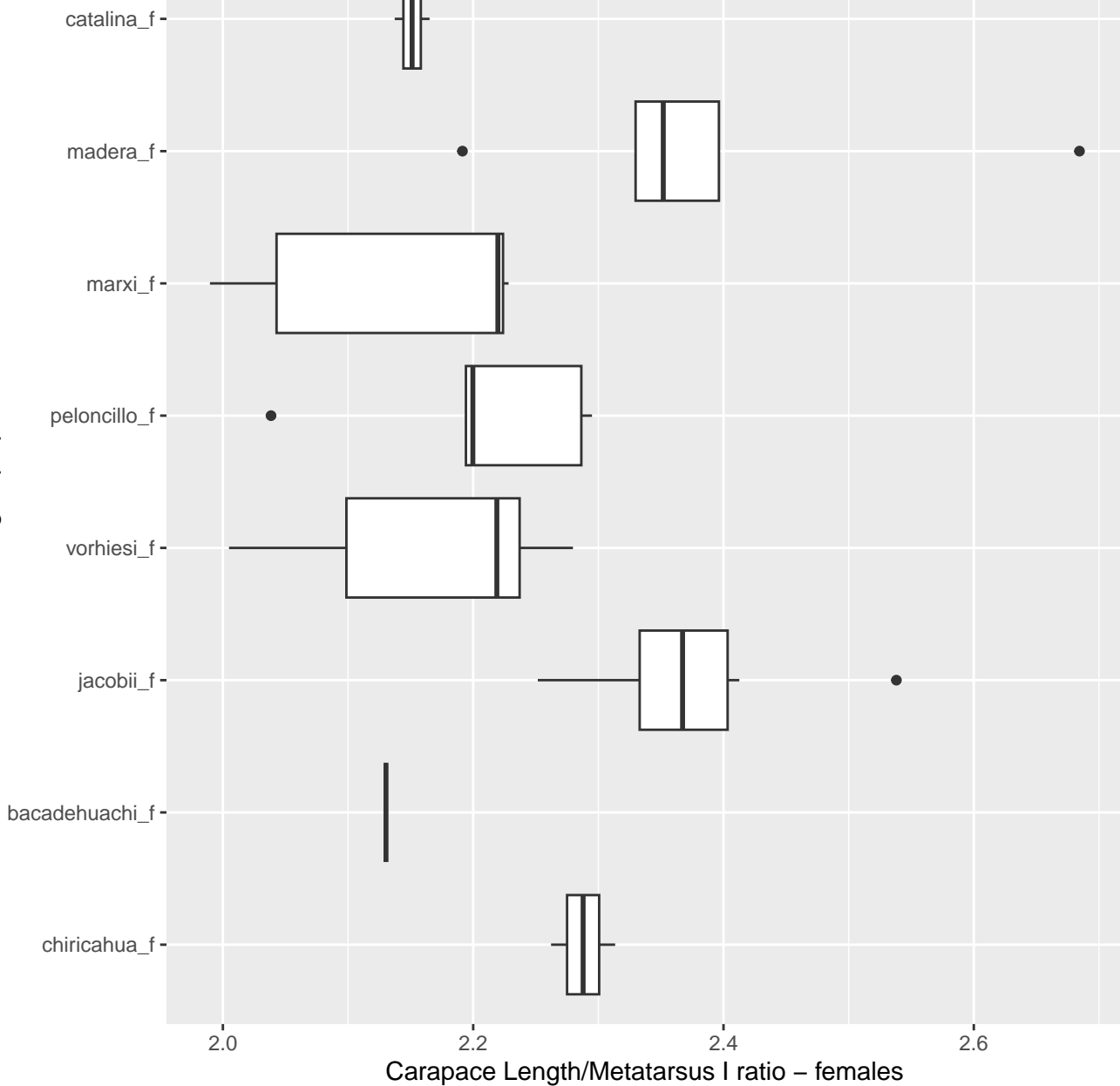

Supplement: Supplementary material 5 — Boxplots comparing various morphometric ratios based on measurements of mature male and female members of the Marxi species group [file zookeys-1210-061_article-125318__-s005.zip › Suppl5/Marxi_group_females/PDFs/ClM1_ratio_females.pdf]

Marxi group species

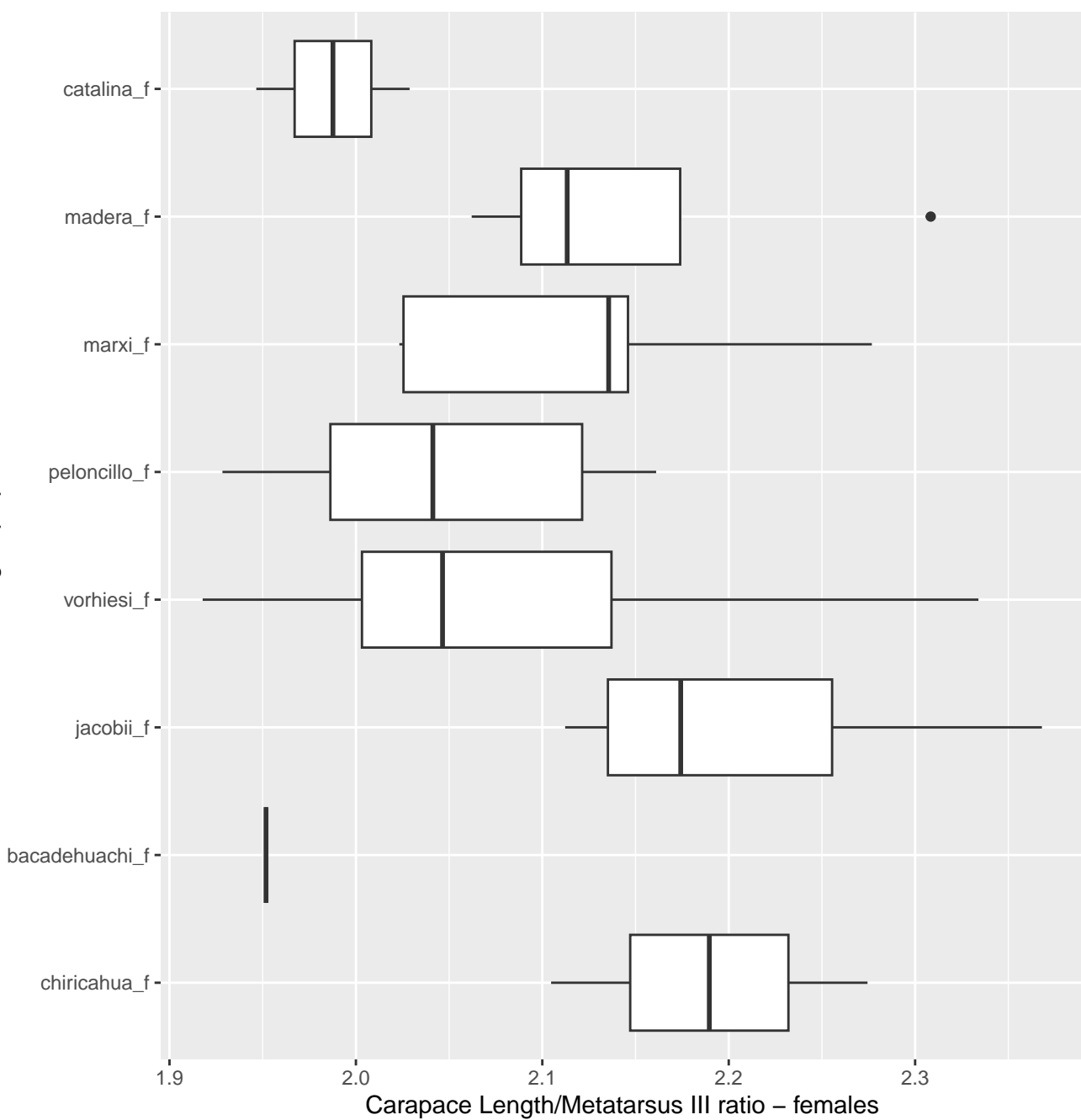

Supplement: Supplementary material 5 — Boxplots comparing various morphometric ratios based on measurements of mature male and female members of the Marxi species group [file zookeys-1210-061_article-125318__-s005.zip › Suppl5/Marxi_group_females/PDFs/ClM3_ratio_females.pdf]

Marxi group species

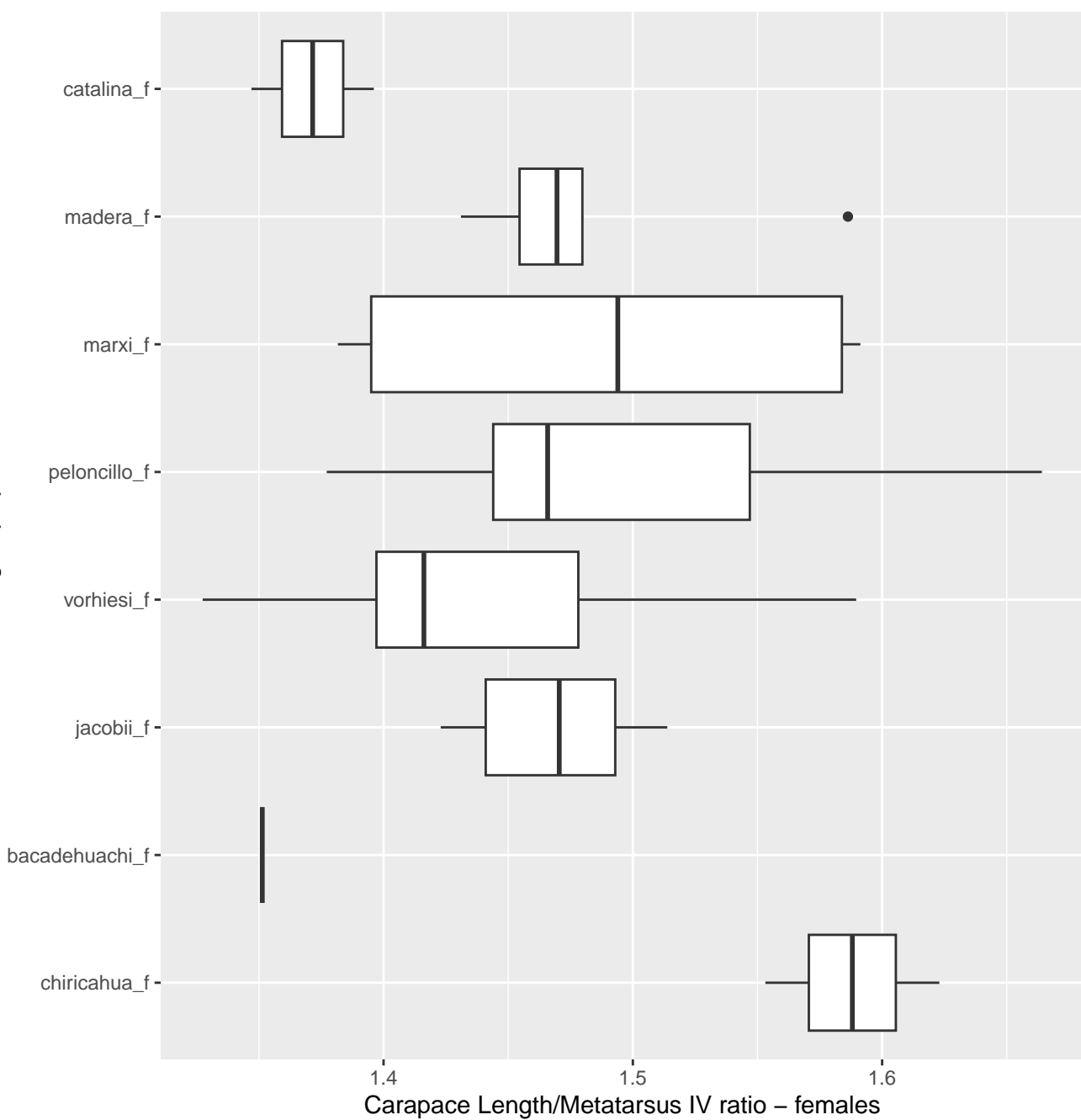

Supplement: Supplementary material 5 — Boxplots comparing various morphometric ratios based on measurements of mature male and female members of the Marxi species group [file zookeys-1210-061_article-125318__-s005.zip › Suppl5/Marxi_group_females/PDFs/ClM4_ratio_females.pdf]

Marxi group species

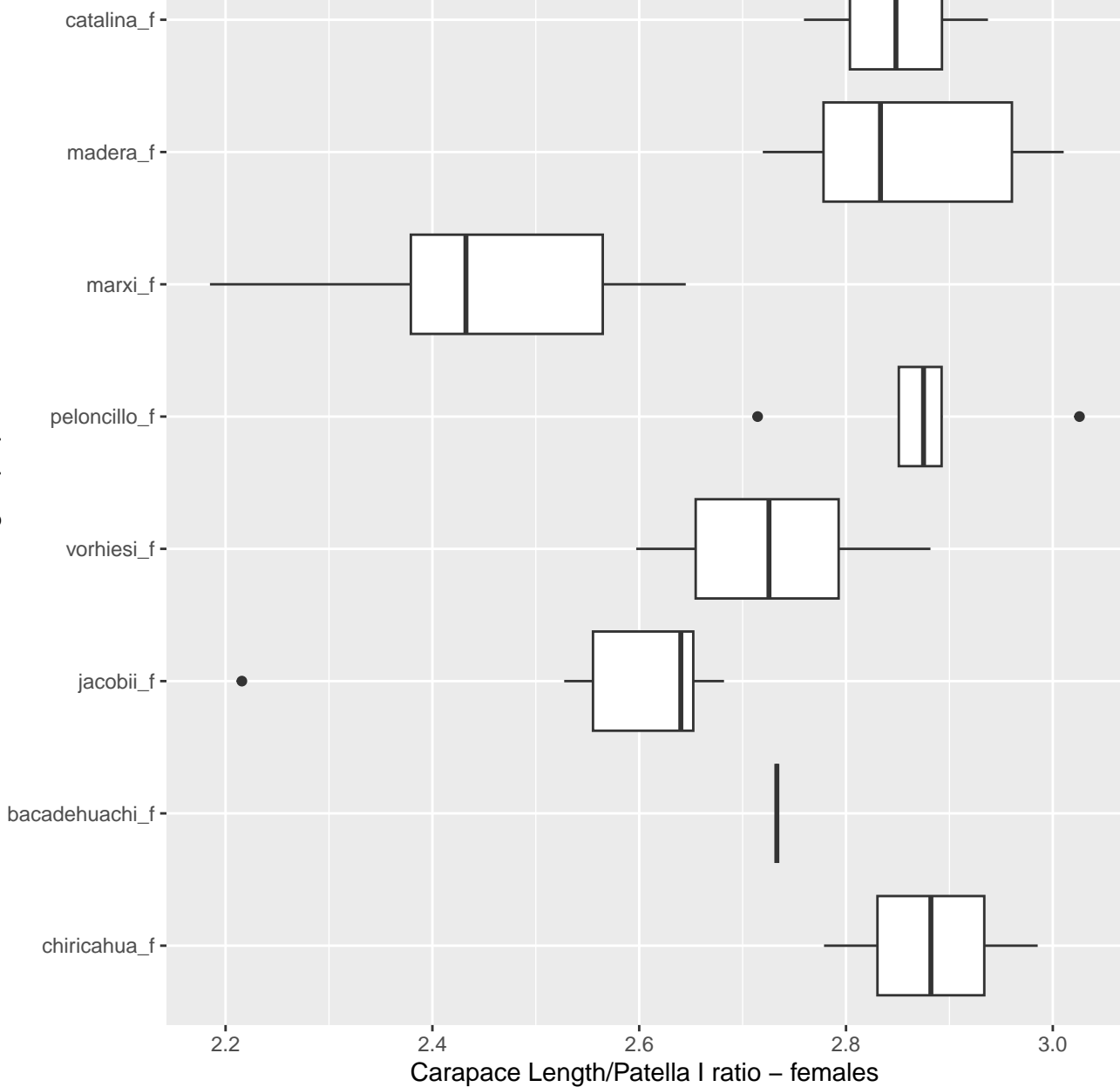

Supplement: Supplementary material 5 — Boxplots comparing various morphometric ratios based on measurements of mature male and female members of the Marxi species group [file zookeys-1210-061_article-125318__-s005.zip › Suppl5/Marxi_group_females/PDFs/ClP1_ratio_females.pdf]

Marxi group species

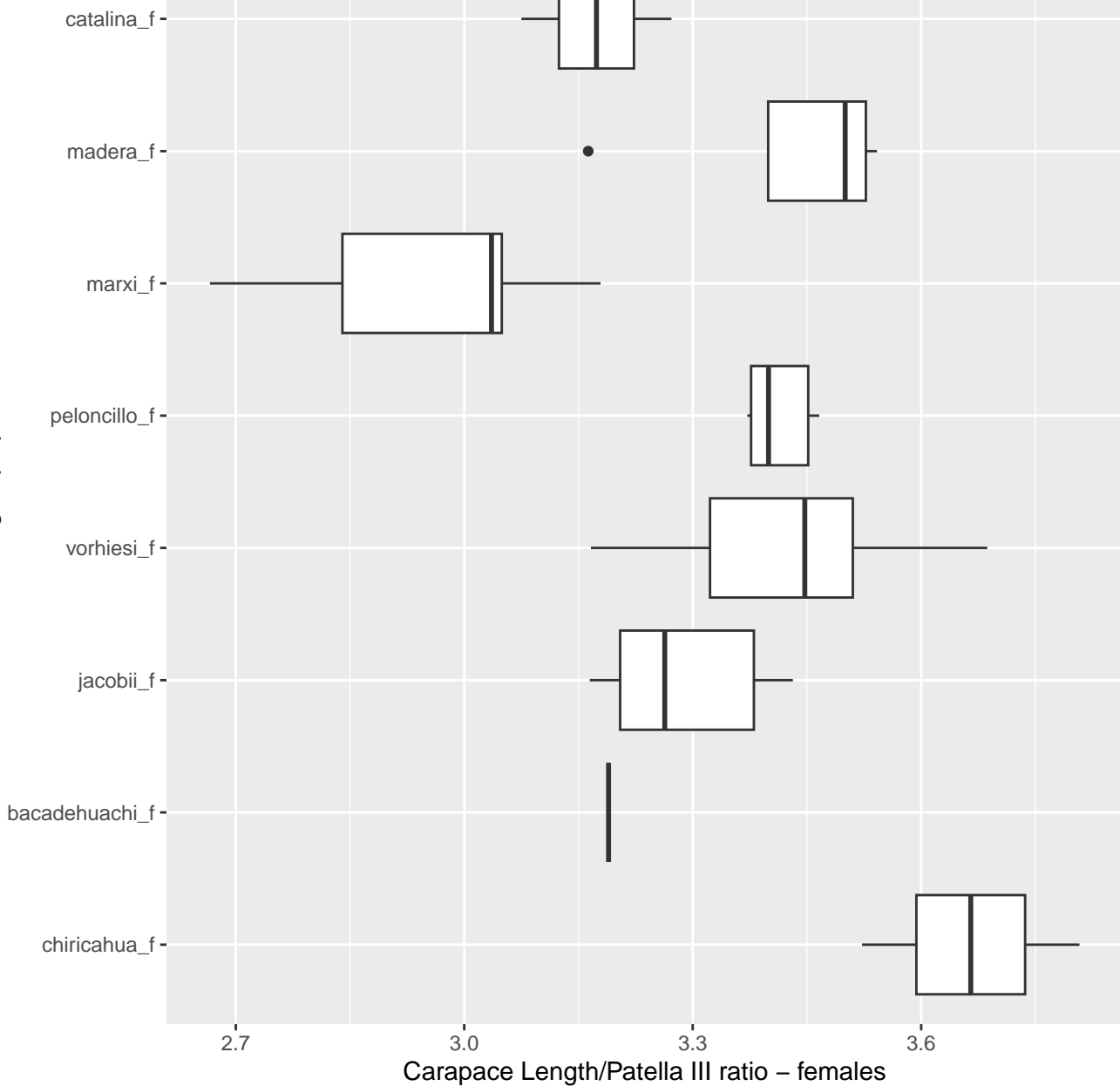

Supplement: Supplementary material 5 — Boxplots comparing various morphometric ratios based on measurements of mature male and female members of the Marxi species group [file zookeys-1210-061_article-125318__-s005.zip › Suppl5/Marxi_group_females/PDFs/ClP3_ratio_females.pdf]

Marxi group species

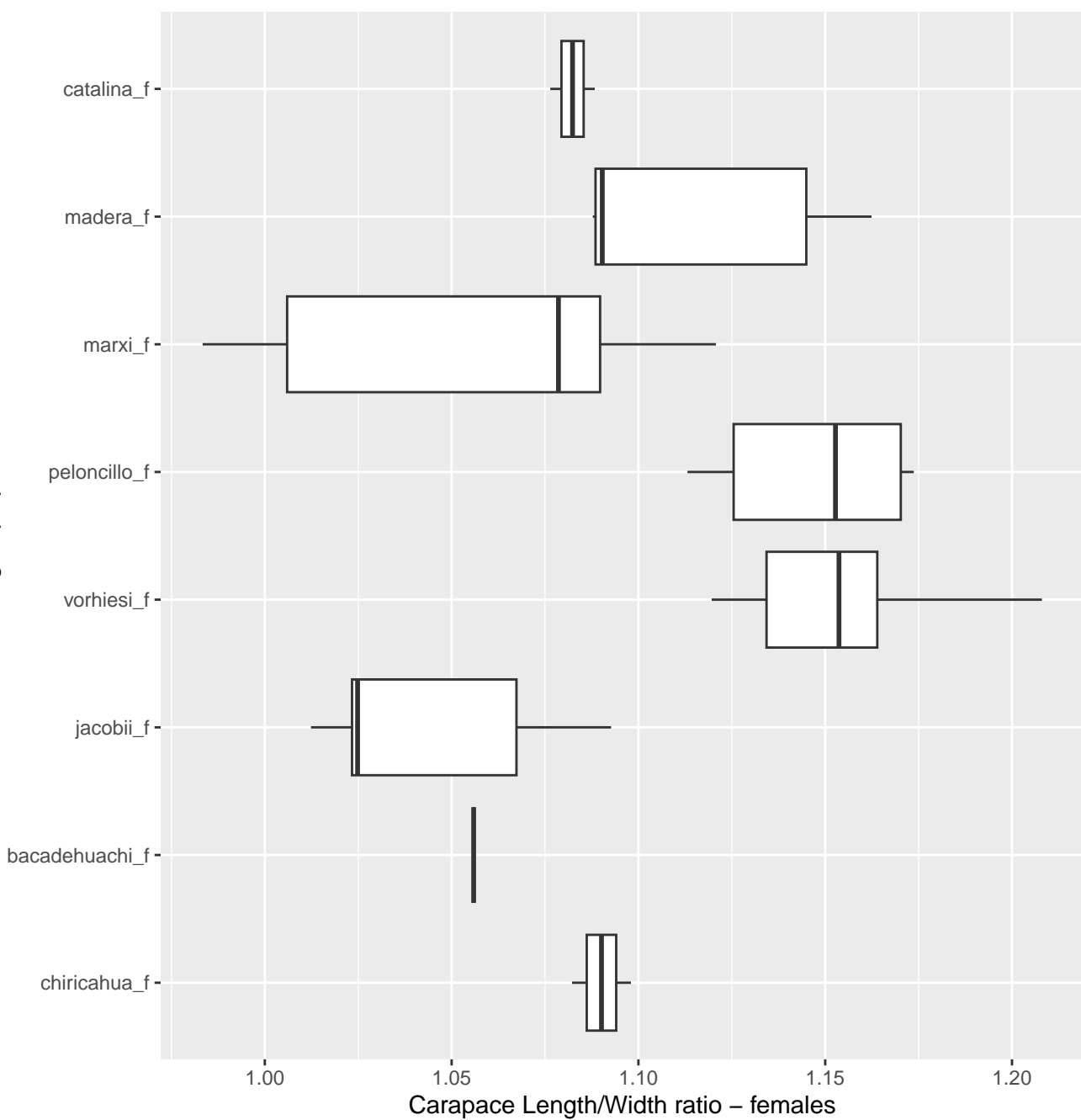

Supplement: Supplementary material 5 — Boxplots comparing various morphometric ratios based on measurements of mature male and female members of the Marxi species group [file zookeys-1210-061_article-125318__-s005.zip › Suppl5/Marxi_group_females/PDFs/Clw_ratio_females.pdf]

Marxi group species

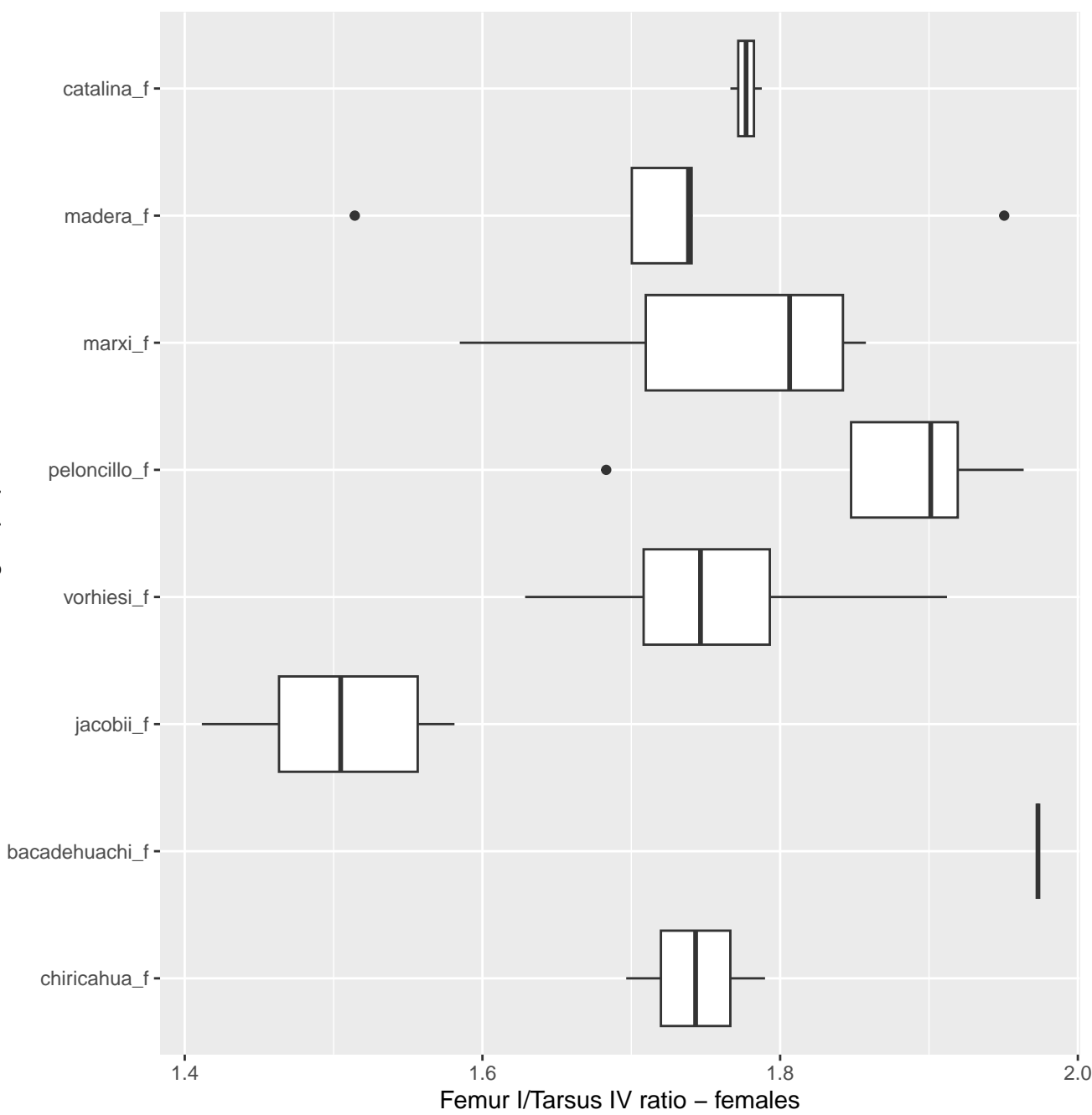

Supplement: Supplementary material 5 — Boxplots comparing various morphometric ratios based on measurements of mature male and female members of the Marxi species group [file zookeys-1210-061_article-125318__-s005.zip › Suppl5/Marxi_group_females/PDFs/F1A4_ratio_females.pdf]

Marxi group species

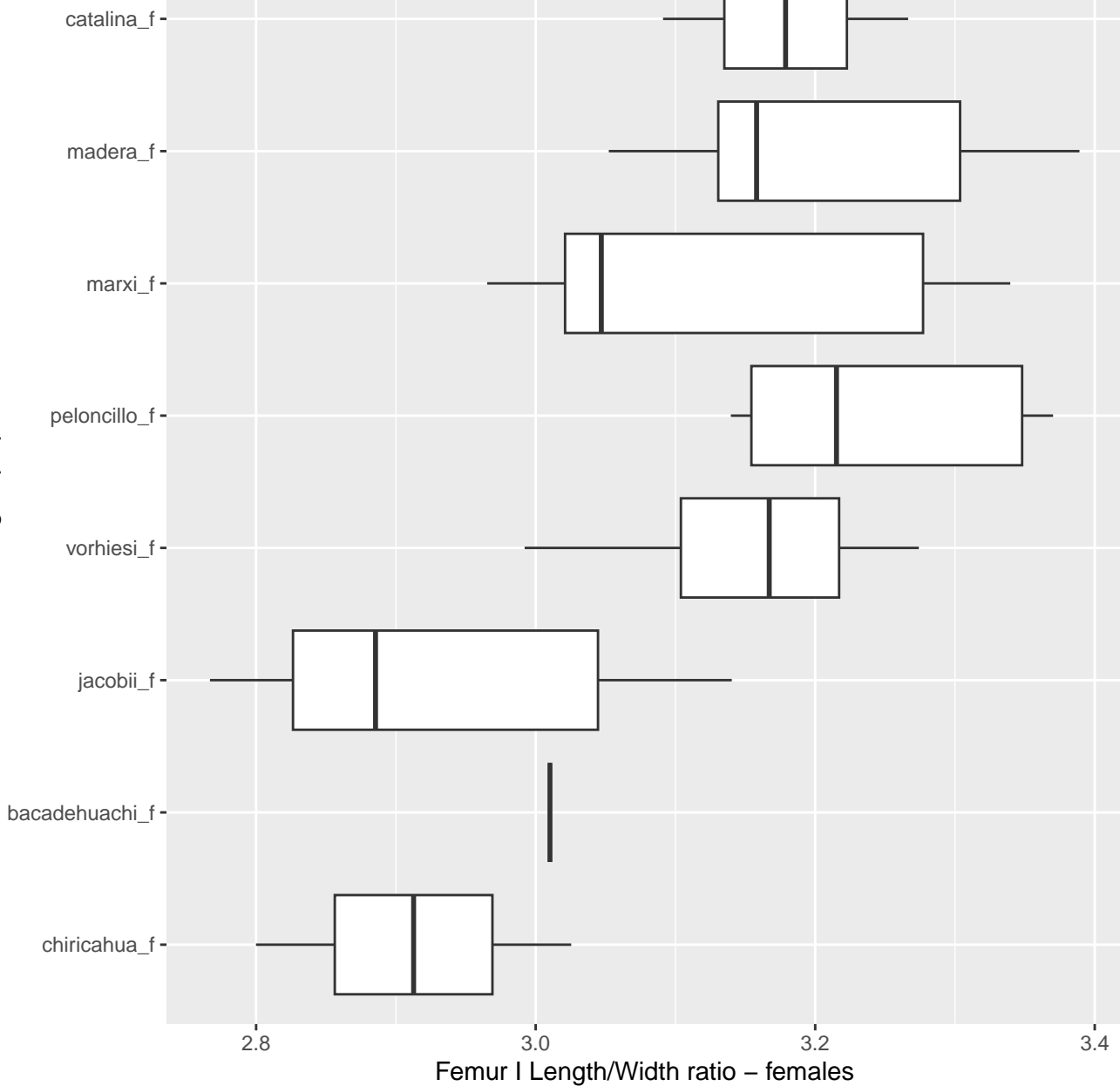

Supplement: Supplementary material 5 — Boxplots comparing various morphometric ratios based on measurements of mature male and female members of the Marxi species group [file zookeys-1210-061_article-125318__-s005.zip › Suppl5/Marxi_group_females/PDFs/F1lw_ratio_females.pdf]

Marxi group species

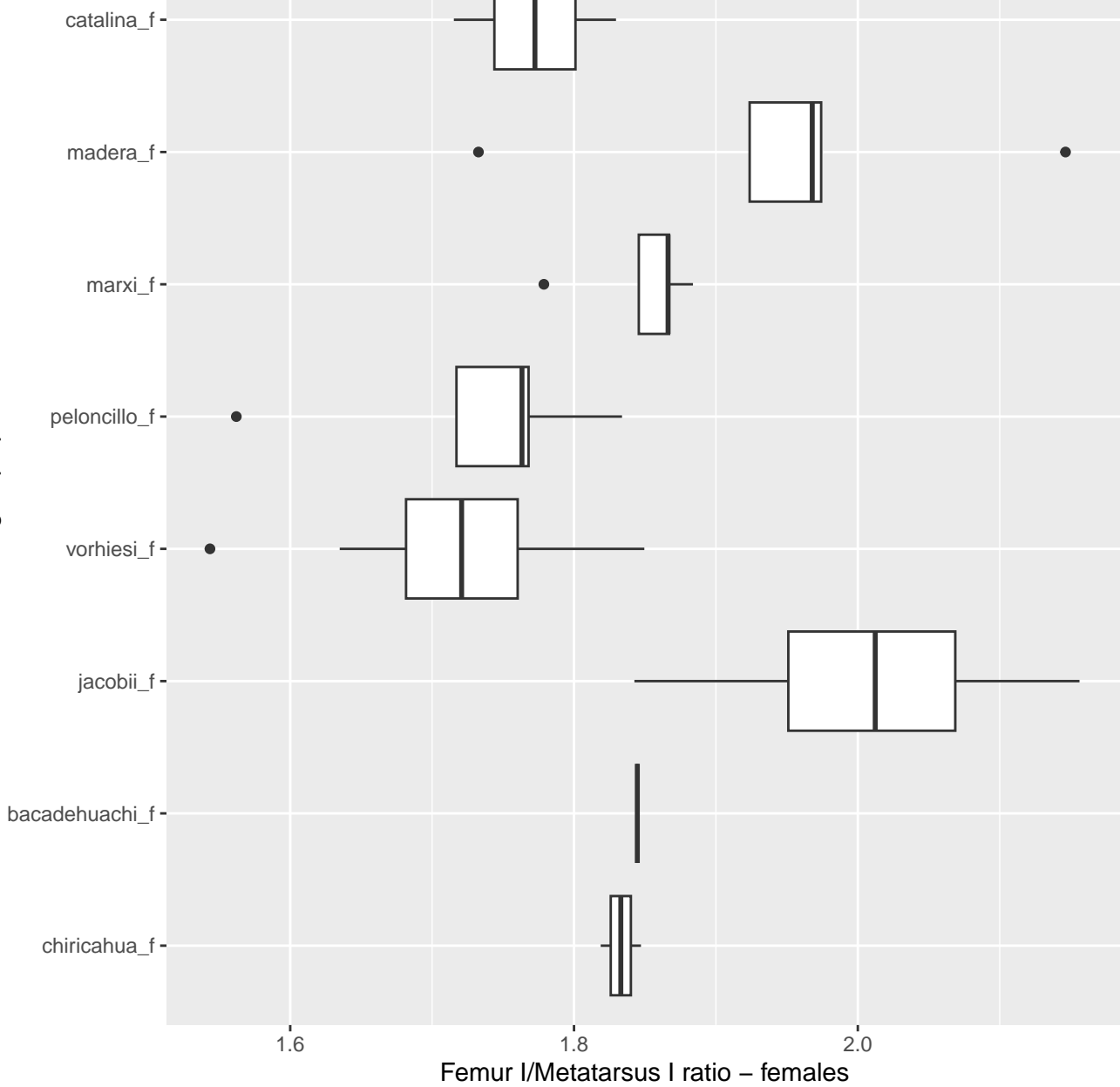

Supplement: Supplementary material 5 — Boxplots comparing various morphometric ratios based on measurements of mature male and female members of the Marxi species group [file zookeys-1210-061_article-125318__-s005.zip › Suppl5/Marxi_group_females/PDFs/F1M1_ratio_females.pdf]

Marxi group species

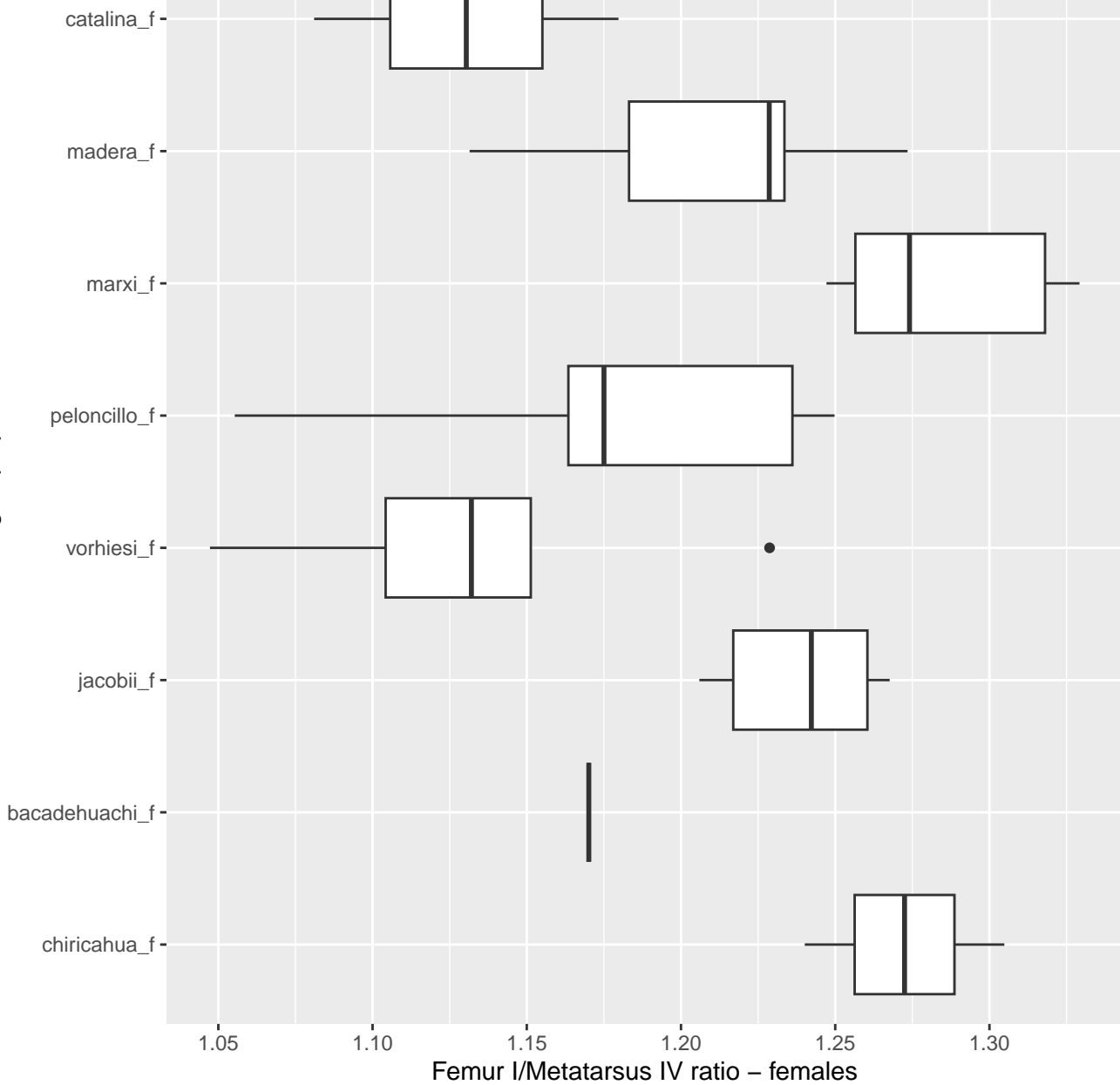

Supplement: Supplementary material 5 — Boxplots comparing various morphometric ratios based on measurements of mature male and female members of the Marxi species group [file zookeys-1210-061_article-125318__-s005.zip › Suppl5/Marxi_group_females/PDFs/F1M4_ratio_females.pdf]

Marxi group species

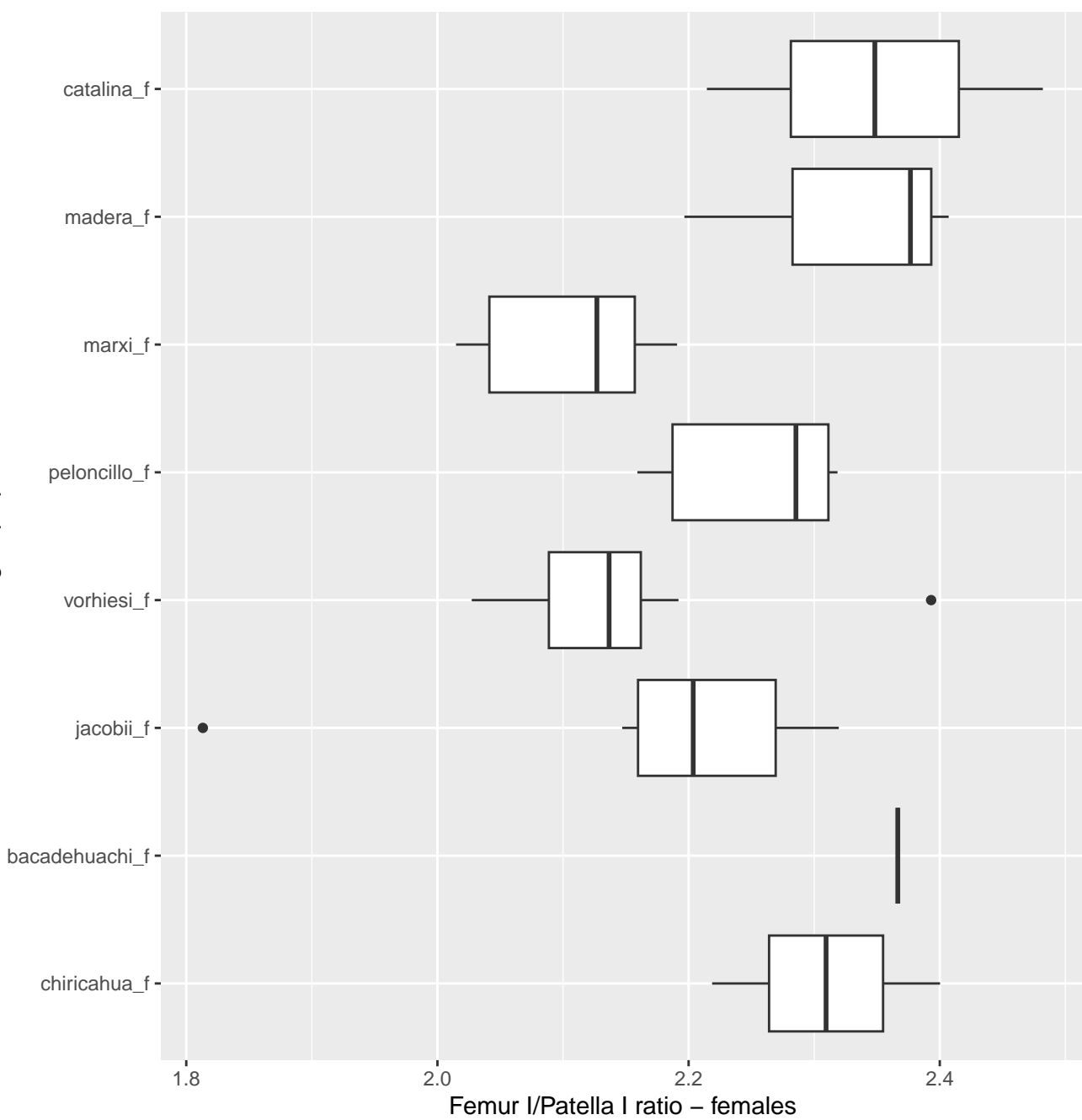

Supplement: Supplementary material 5 — Boxplots comparing various morphometric ratios based on measurements of mature male and female members of the Marxi species group [file zookeys-1210-061_article-125318__-s005.zip › Suppl5/Marxi_group_females/PDFs/F1P1_ratio_females.pdf]

Marxi group species

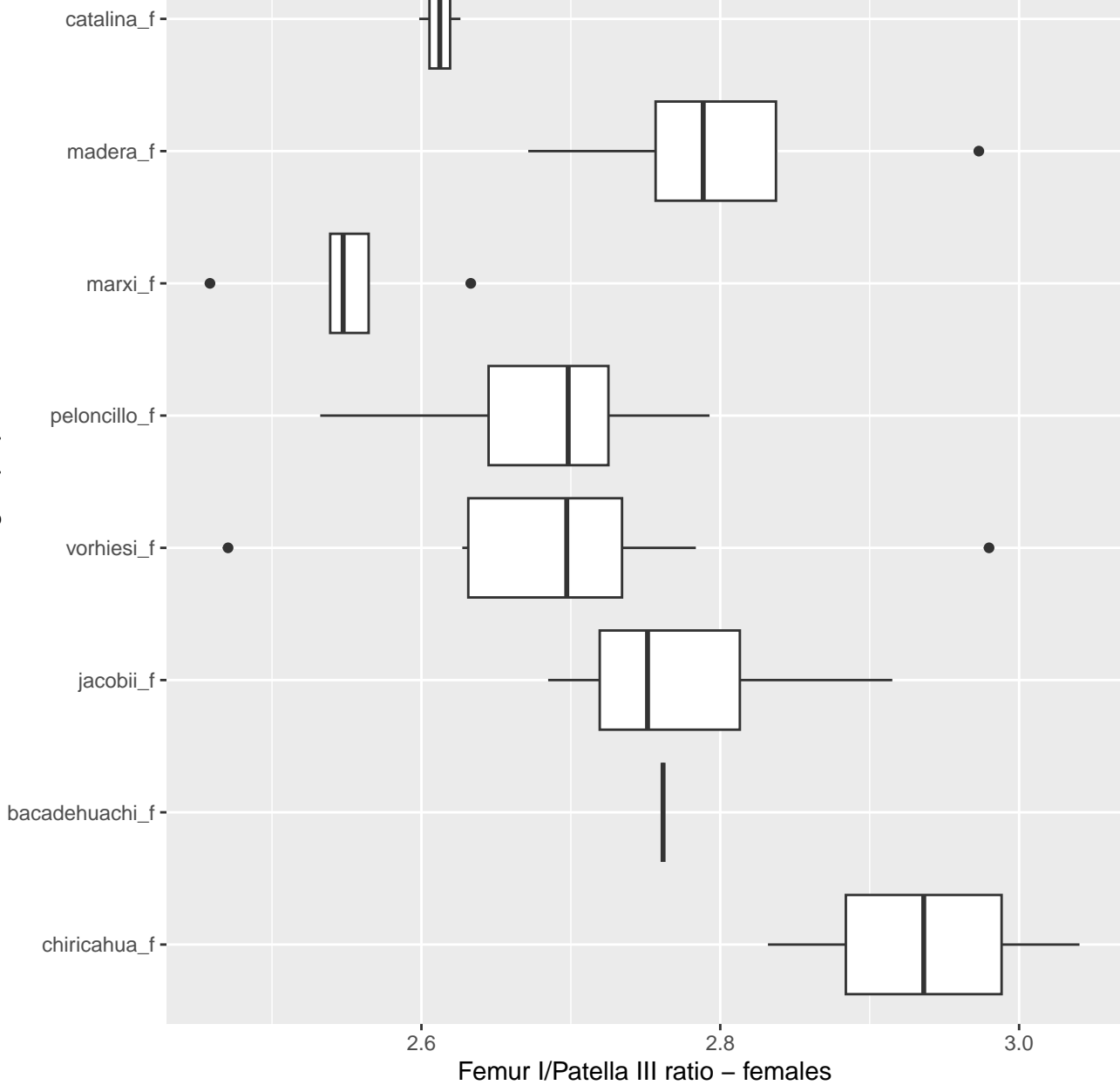

Supplement: Supplementary material 5 — Boxplots comparing various morphometric ratios based on measurements of mature male and female members of the Marxi species group [file zookeys-1210-061_article-125318__-s005.zip › Suppl5/Marxi_group_females/PDFs/F1P3_ratio_females.pdf]

Marxi group species

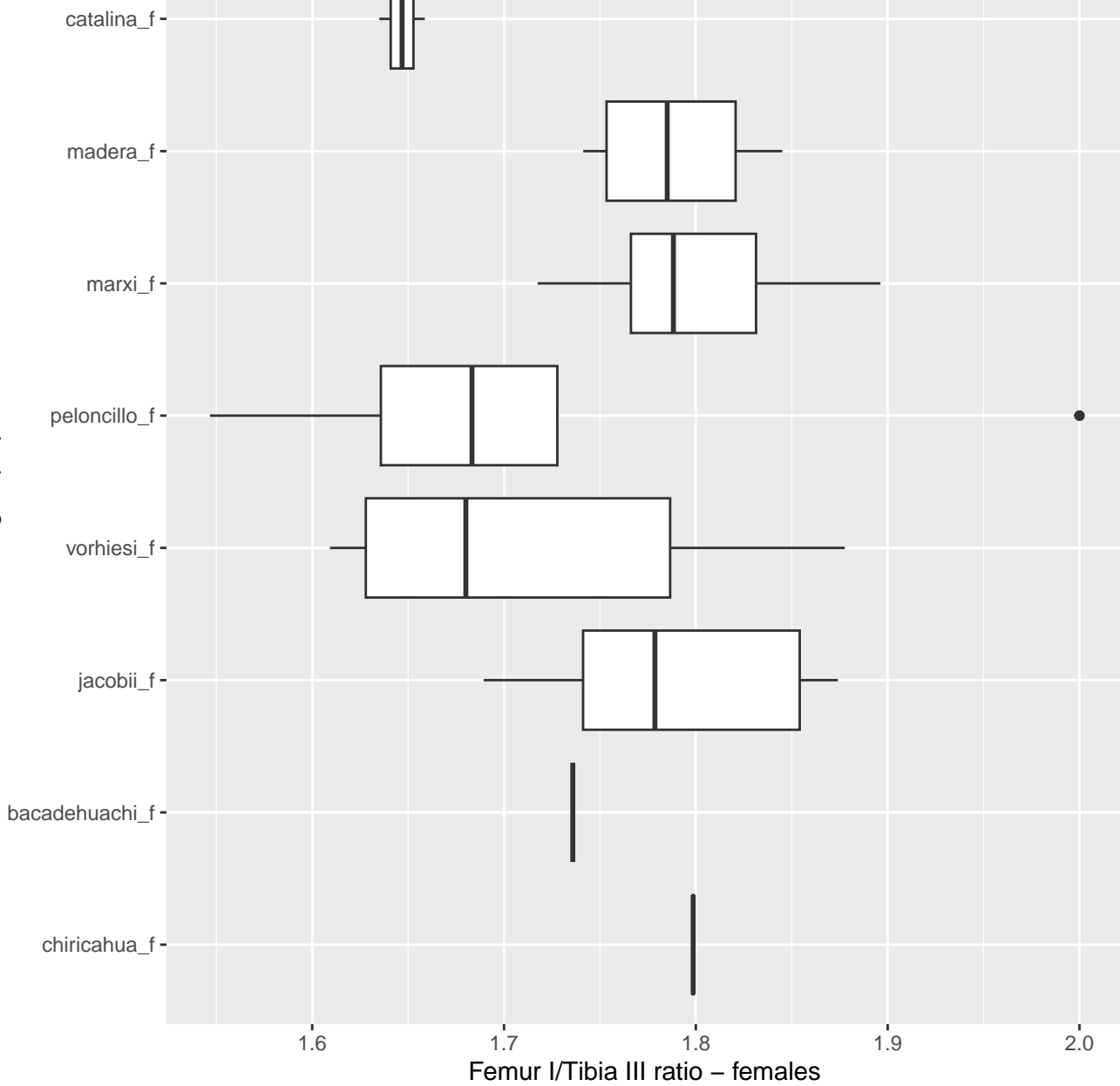

Supplement: Supplementary material 5 — Boxplots comparing various morphometric ratios based on measurements of mature male and female members of the Marxi species group [file zookeys-1210-061_article-125318__-s005.zip › Suppl5/Marxi_group_females/PDFs/F1T3_ratio_females.pdf]

Marxi group species

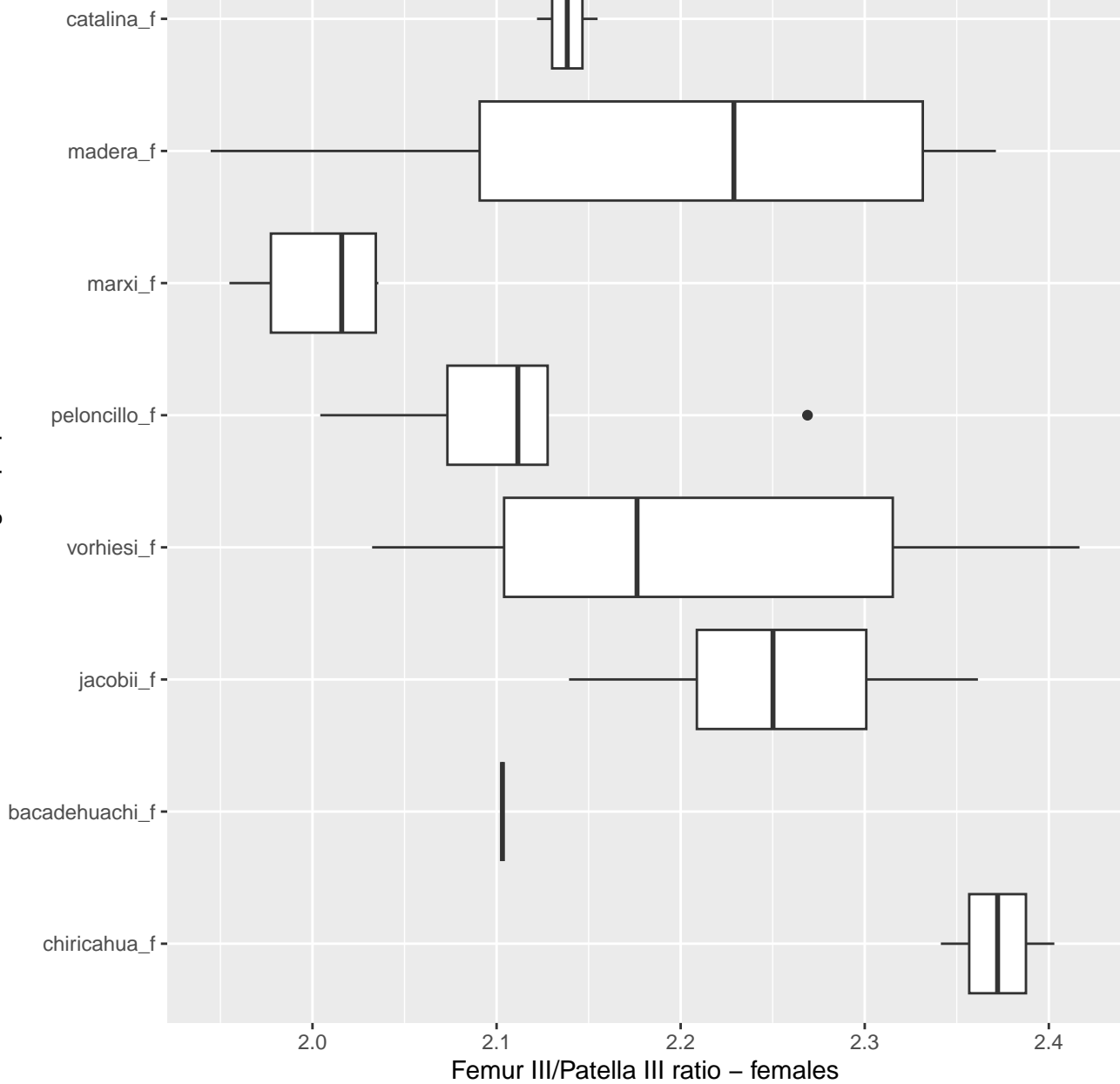

Supplement: Supplementary material 5 — Boxplots comparing various morphometric ratios based on measurements of mature male and female members of the Marxi species group [file zookeys-1210-061_article-125318__-s005.zip › Suppl5/Marxi_group_females/PDFs/F3P3_ratio_females.pdf]

Marxi group species

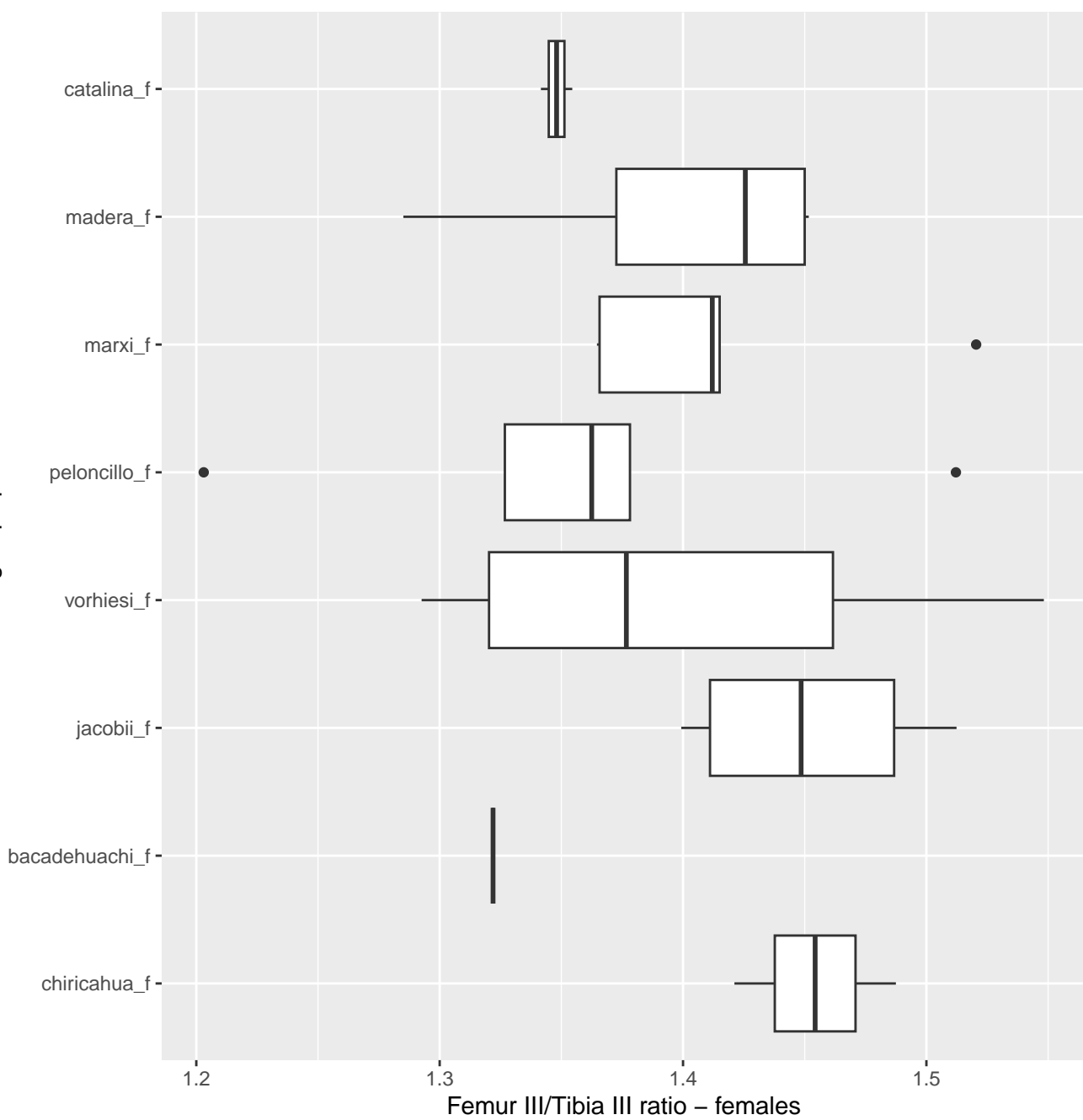

Supplement: Supplementary material 5 — Boxplots comparing various morphometric ratios based on measurements of mature male and female members of the Marxi species group [file zookeys-1210-061_article-125318__-s005.zip › Suppl5/Marxi_group_females/PDFs/F3T3_ratio_females.pdf]

Marxi group species

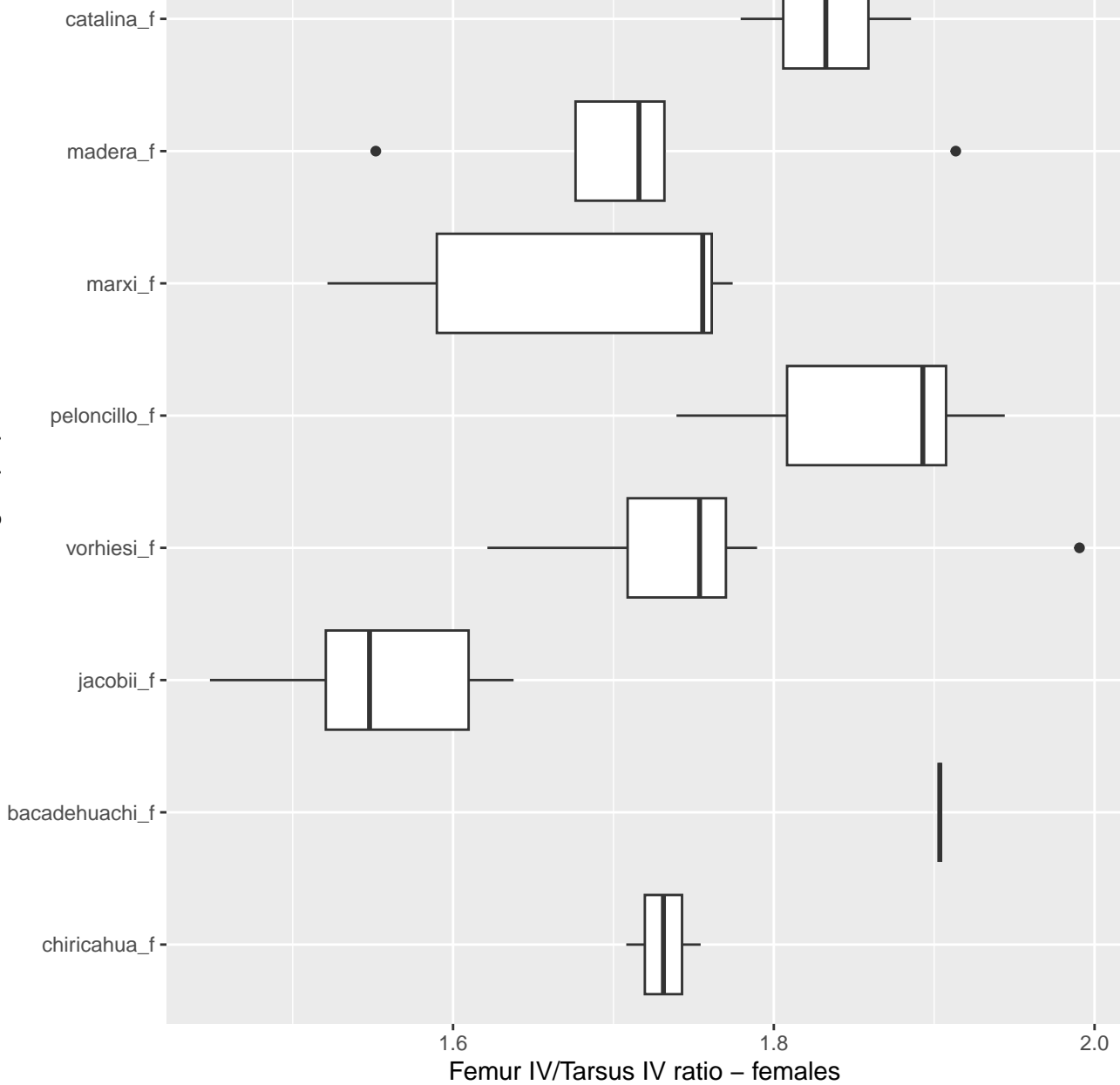

Supplement: Supplementary material 5 — Boxplots comparing various morphometric ratios based on measurements of mature male and female members of the Marxi species group [file zookeys-1210-061_article-125318__-s005.zip › Suppl5/Marxi_group_females/PDFs/F4A4_ratio_females.pdf]

Marxi group species

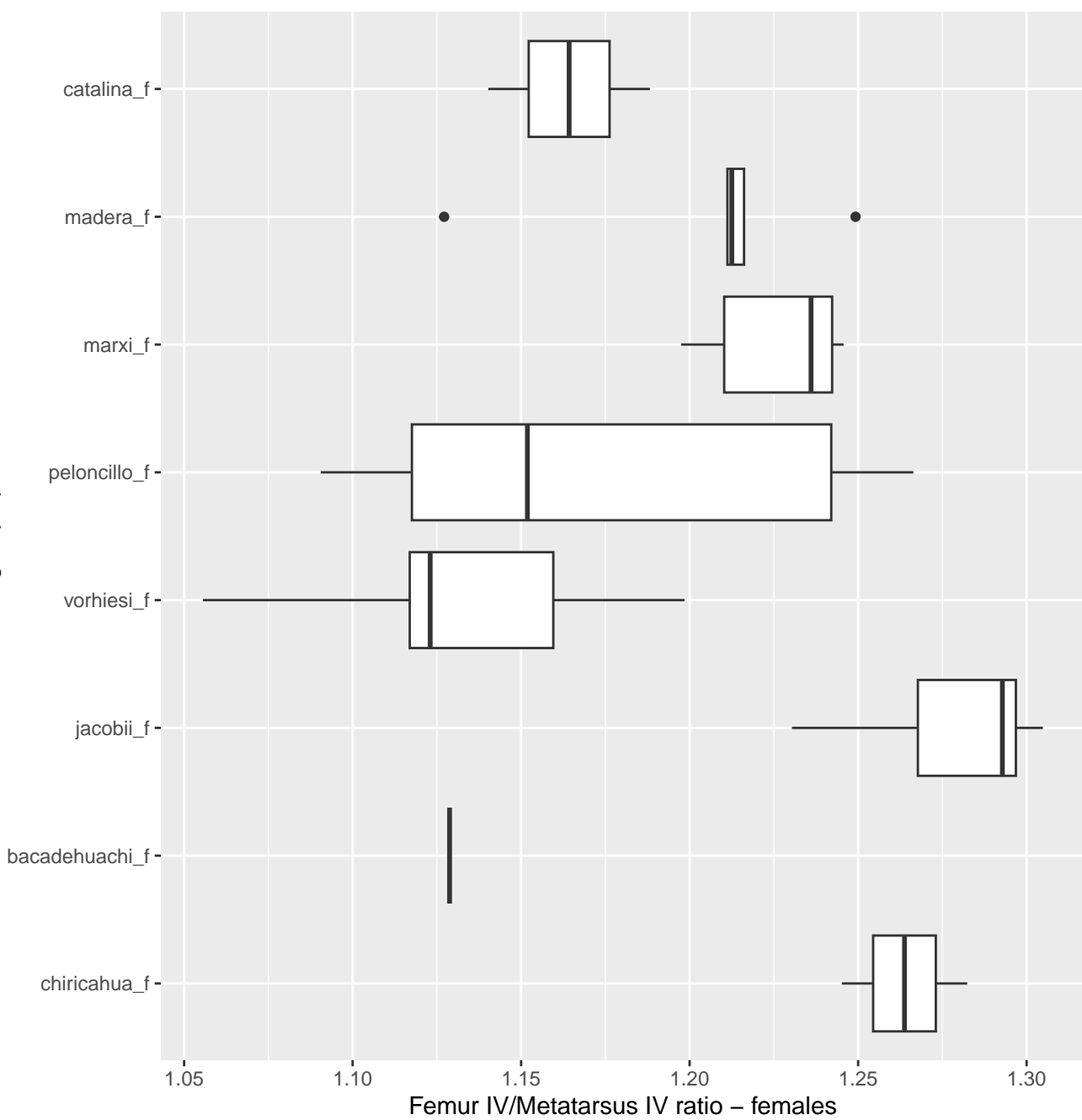

Supplement: Supplementary material 5 — Boxplots comparing various morphometric ratios based on measurements of mature male and female members of the Marxi species group [file zookeys-1210-061_article-125318__-s005.zip › Suppl5/Marxi_group_females/PDFs/F4M4_ratio_females.pdf]

Marxi group species

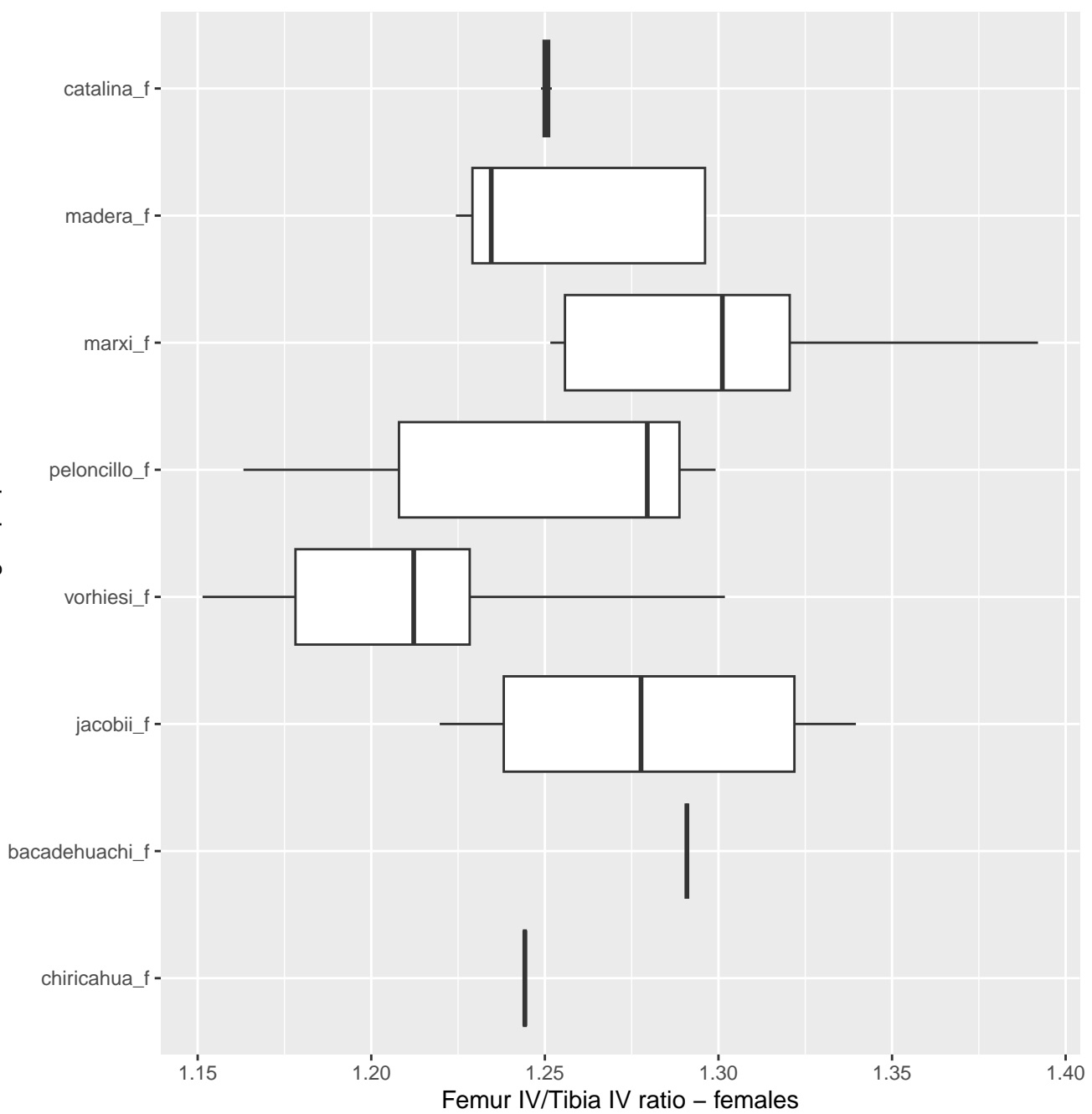

Supplement: Supplementary material 5 — Boxplots comparing various morphometric ratios based on measurements of mature male and female members of the Marxi species group [file zookeys-1210-061_article-125318__-s005.zip › Suppl5/Marxi_group_females/PDFs/F4T4_ratio_females.pdf]

Marxi group species

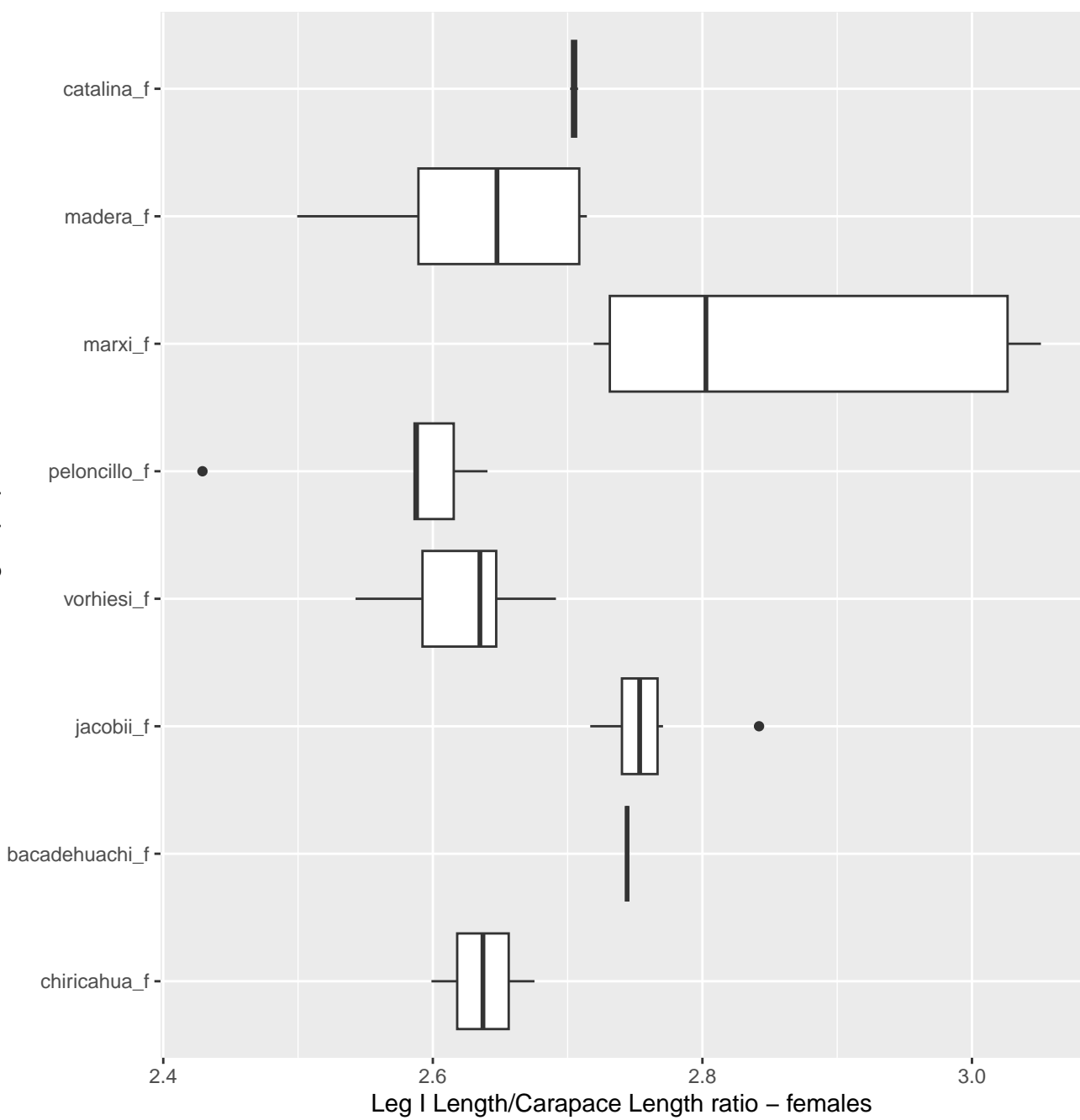

Supplement: Supplementary material 5 — Boxplots comparing various morphometric ratios based on measurements of mature male and female members of the Marxi species group [file zookeys-1210-061_article-125318__-s005.zip › Suppl5/Marxi_group_females/PDFs/L1Cl_ratio_females.pdf]

Marxi group species

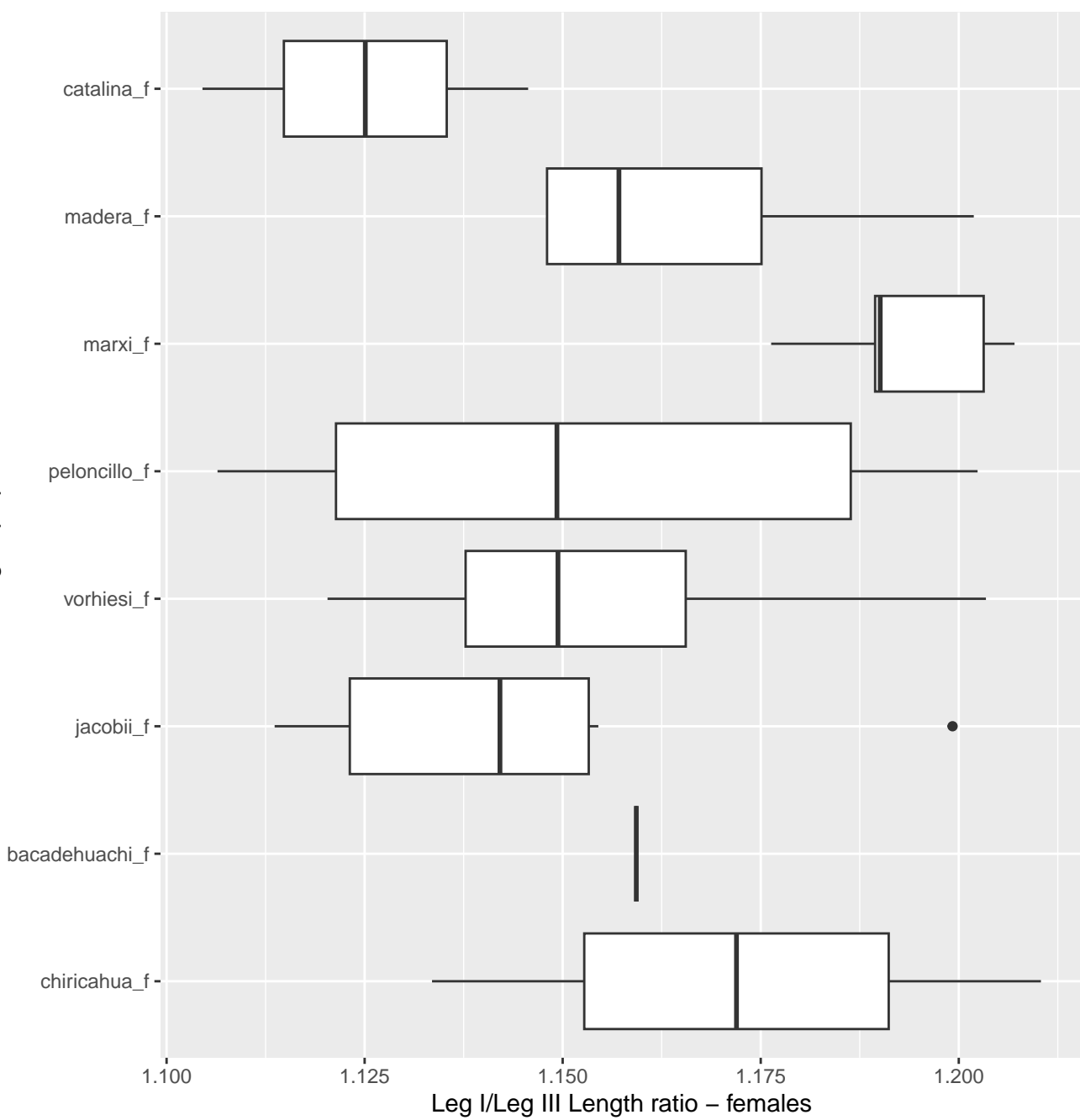

Supplement: Supplementary material 5 — Boxplots comparing various morphometric ratios based on measurements of mature male and female members of the Marxi species group [file zookeys-1210-061_article-125318__-s005.zip › Suppl5/Marxi_group_females/PDFs/L1L3_ratio_females.pdf]

Marxi group species

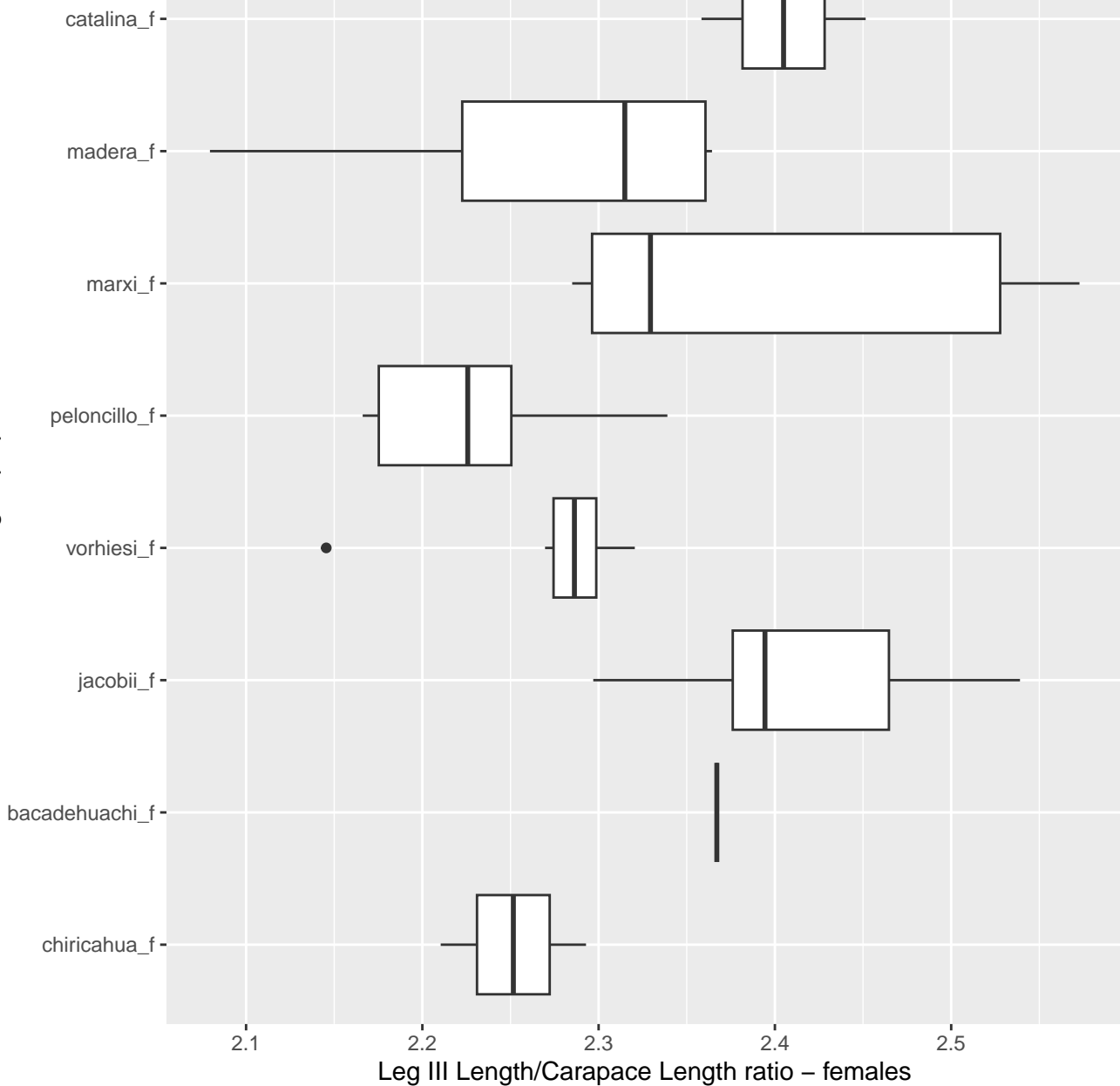

Supplement: Supplementary material 5 — Boxplots comparing various morphometric ratios based on measurements of mature male and female members of the Marxi species group [file zookeys-1210-061_article-125318__-s005.zip › Suppl5/Marxi_group_females/PDFs/L3Cl_ratio_females.pdf]

Marxi group species

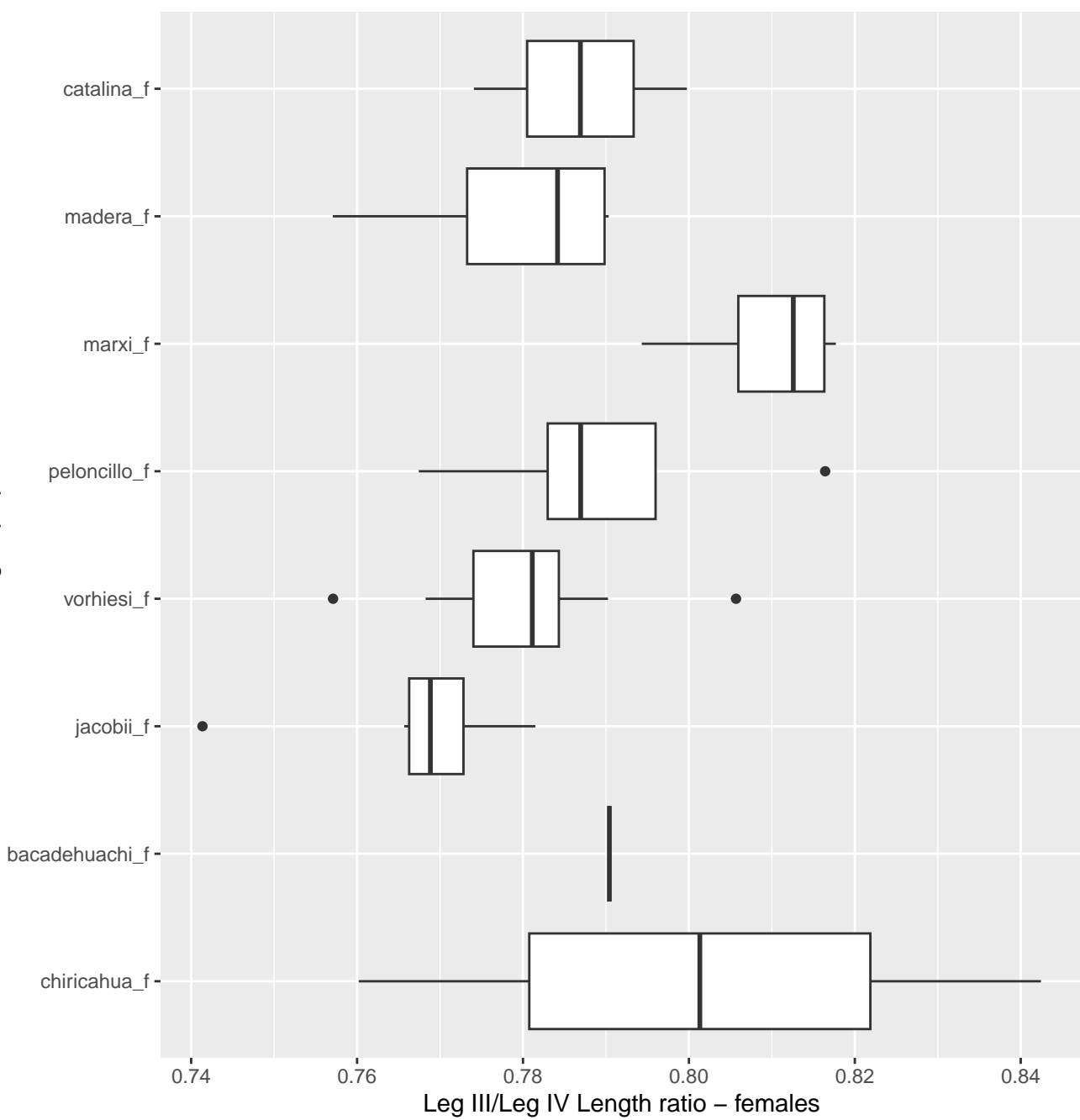

Supplement: Supplementary material 5 — Boxplots comparing various morphometric ratios based on measurements of mature male and female members of the Marxi species group [file zookeys-1210-061_article-125318__-s005.zip › Suppl5/Marxi_group_females/PDFs/L3L4_ratio_females.pdf]

Marxi group species

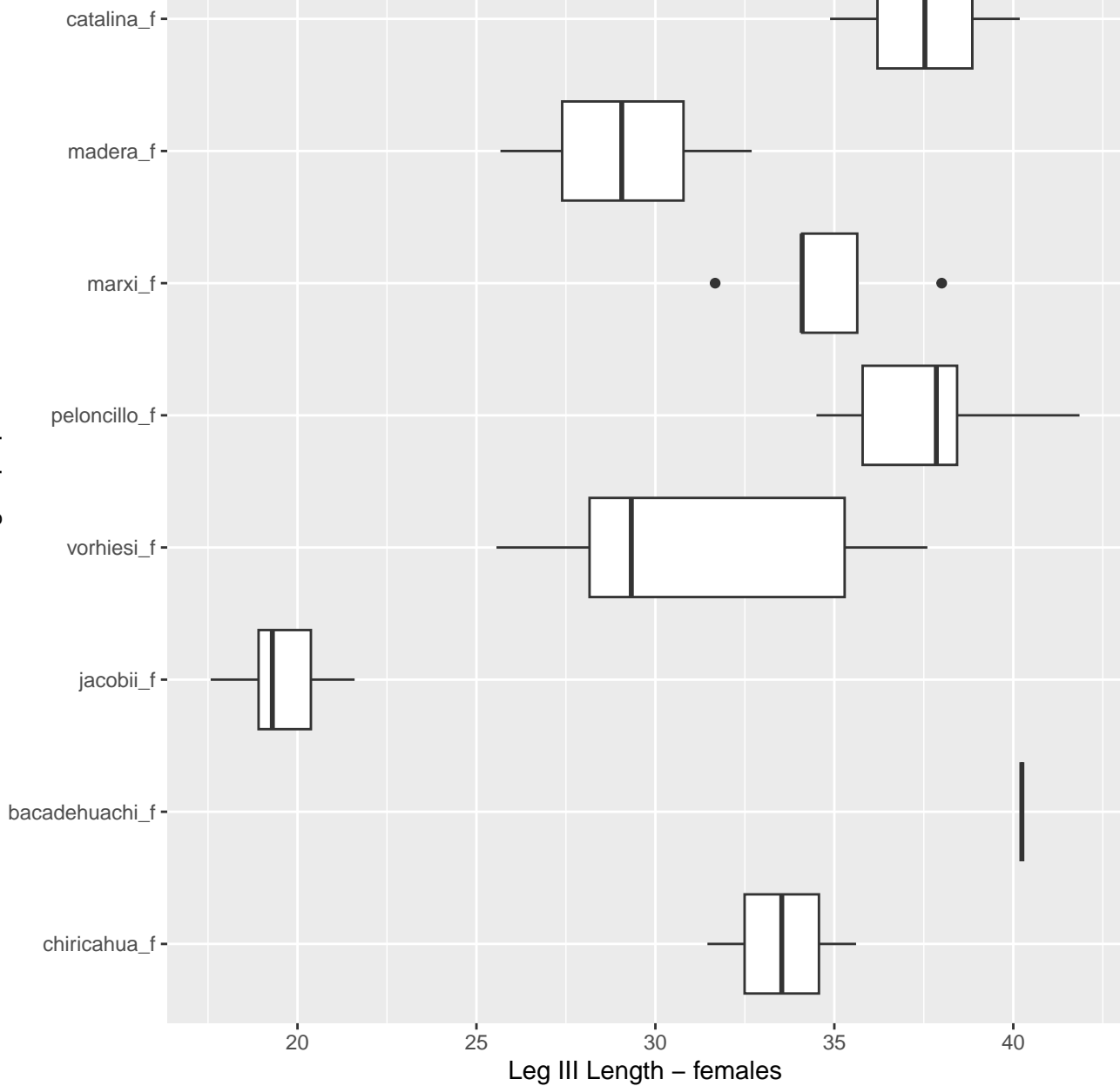

Supplement: Supplementary material 5 — Boxplots comparing various morphometric ratios based on measurements of mature male and female members of the Marxi species group [file zookeys-1210-061_article-125318__-s005.zip › Suppl5/Marxi_group_females/PDFs/L3_length_females.pdf]

Marxi group species

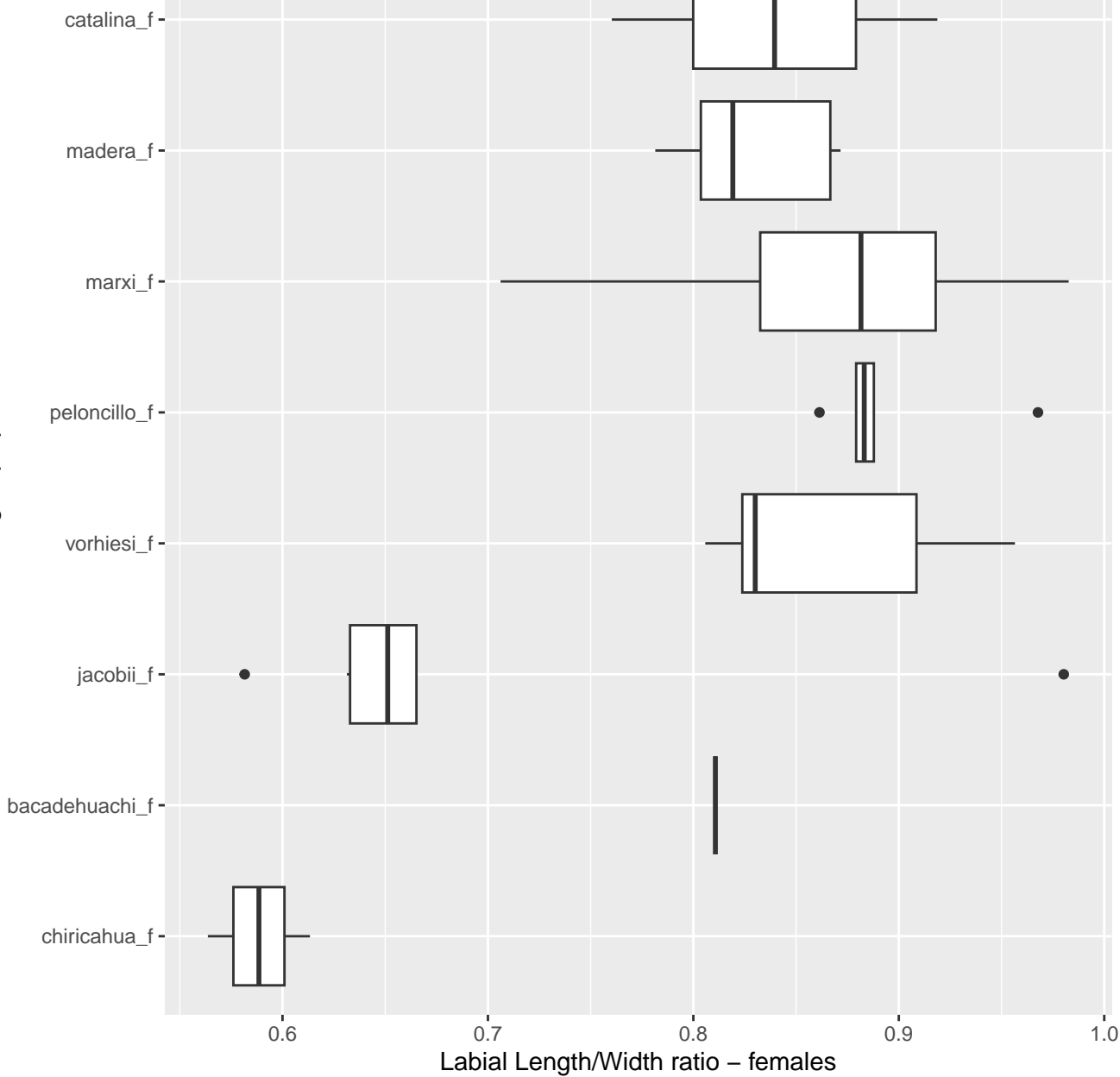

Supplement: Supplementary material 5 — Boxplots comparing various morphometric ratios based on measurements of mature male and female members of the Marxi species group [file zookeys-1210-061_article-125318__-s005.zip › Suppl5/Marxi_group_females/PDFs/LBlw_ratio_females.pdf]

Marxi group species

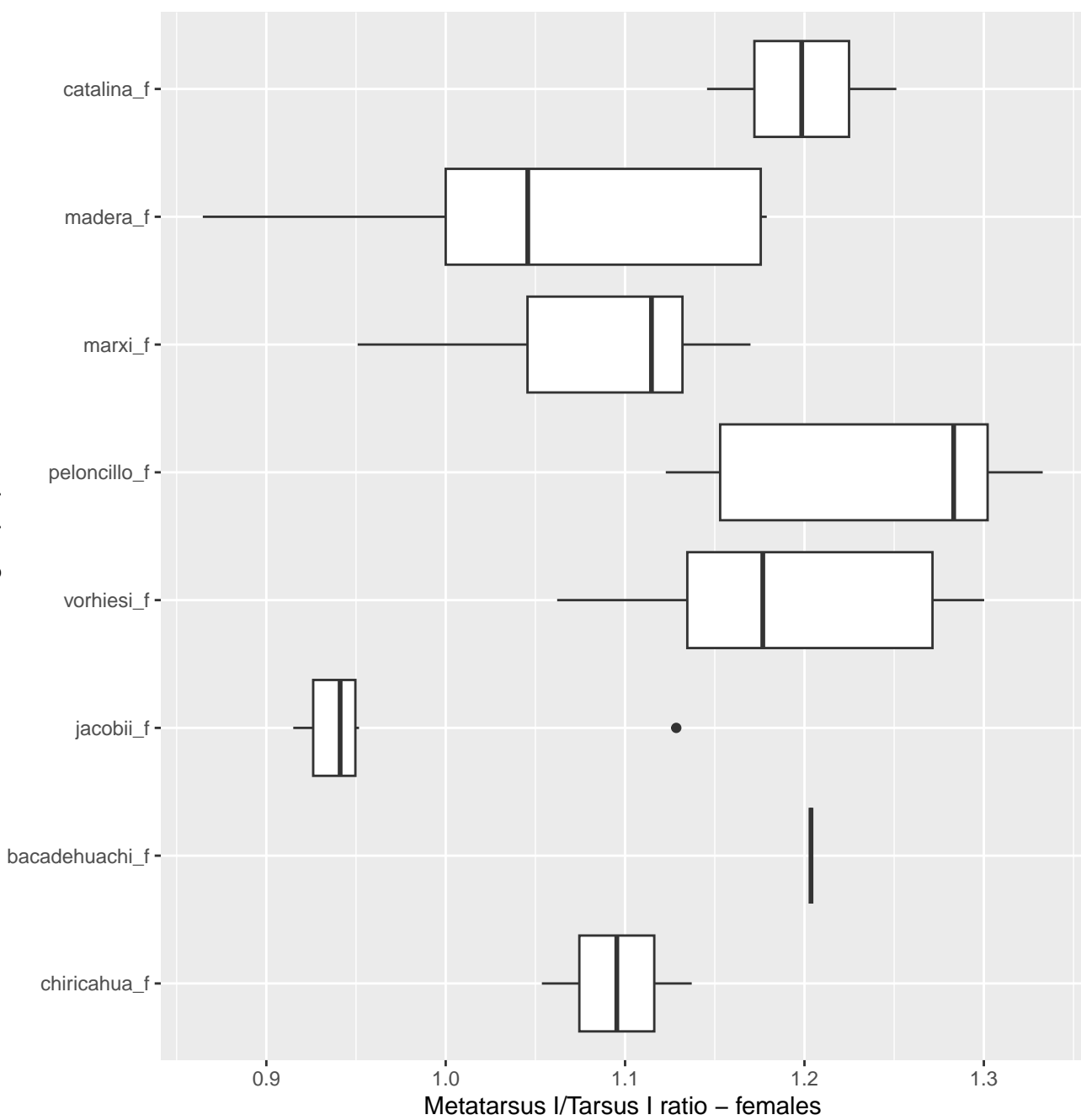

Supplement: Supplementary material 5 — Boxplots comparing various morphometric ratios based on measurements of mature male and female members of the Marxi species group [file zookeys-1210-061_article-125318__-s005.zip › Suppl5/Marxi_group_females/PDFs/M1A1_ratio_females.pdf]

Marxi group species

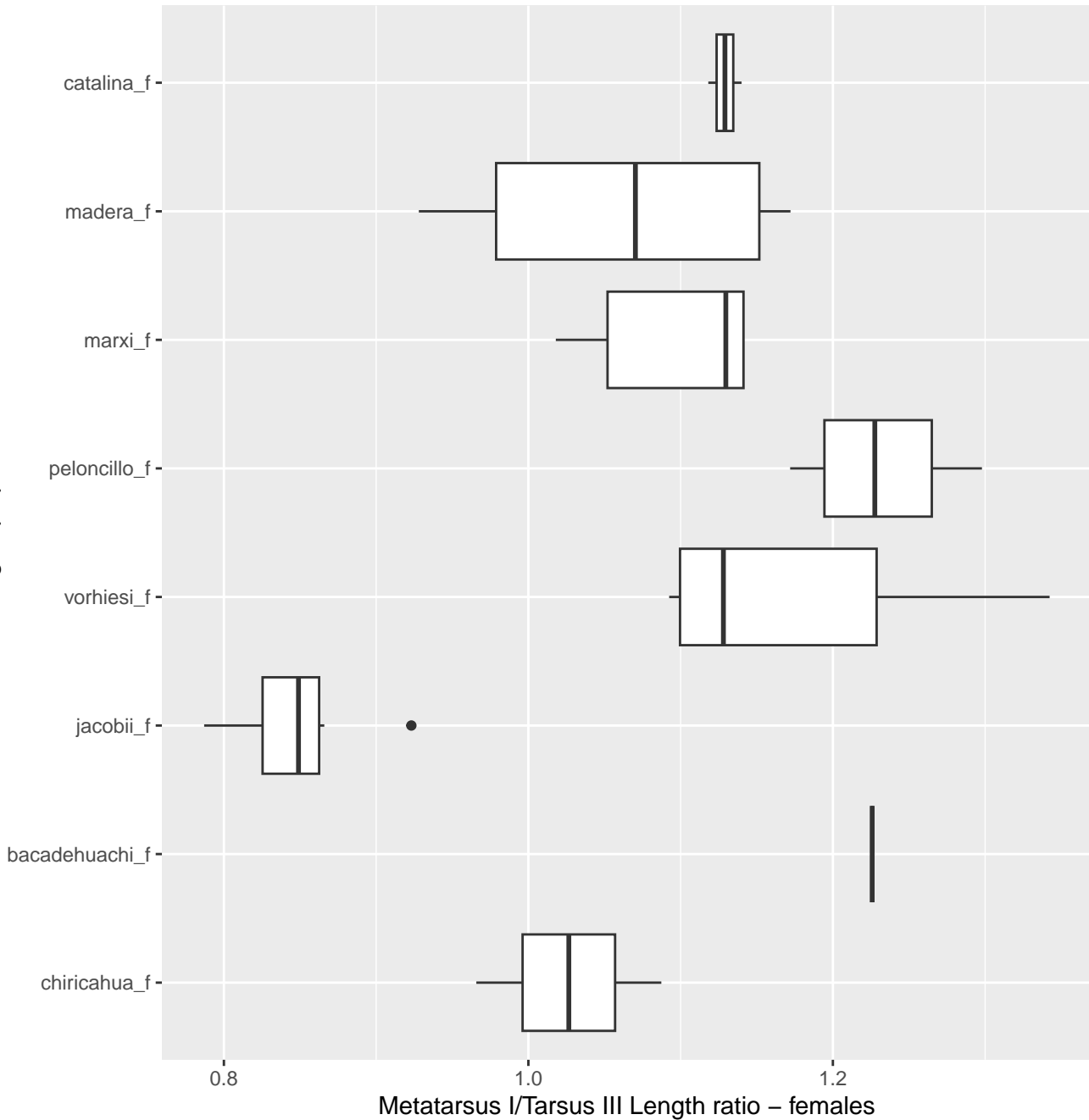

Supplement: Supplementary material 5 — Boxplots comparing various morphometric ratios based on measurements of mature male and female members of the Marxi species group [file zookeys-1210-061_article-125318__-s005.zip › Suppl5/Marxi_group_females/PDFs/M1A3_ratio_females.pdf]

Marxi group species

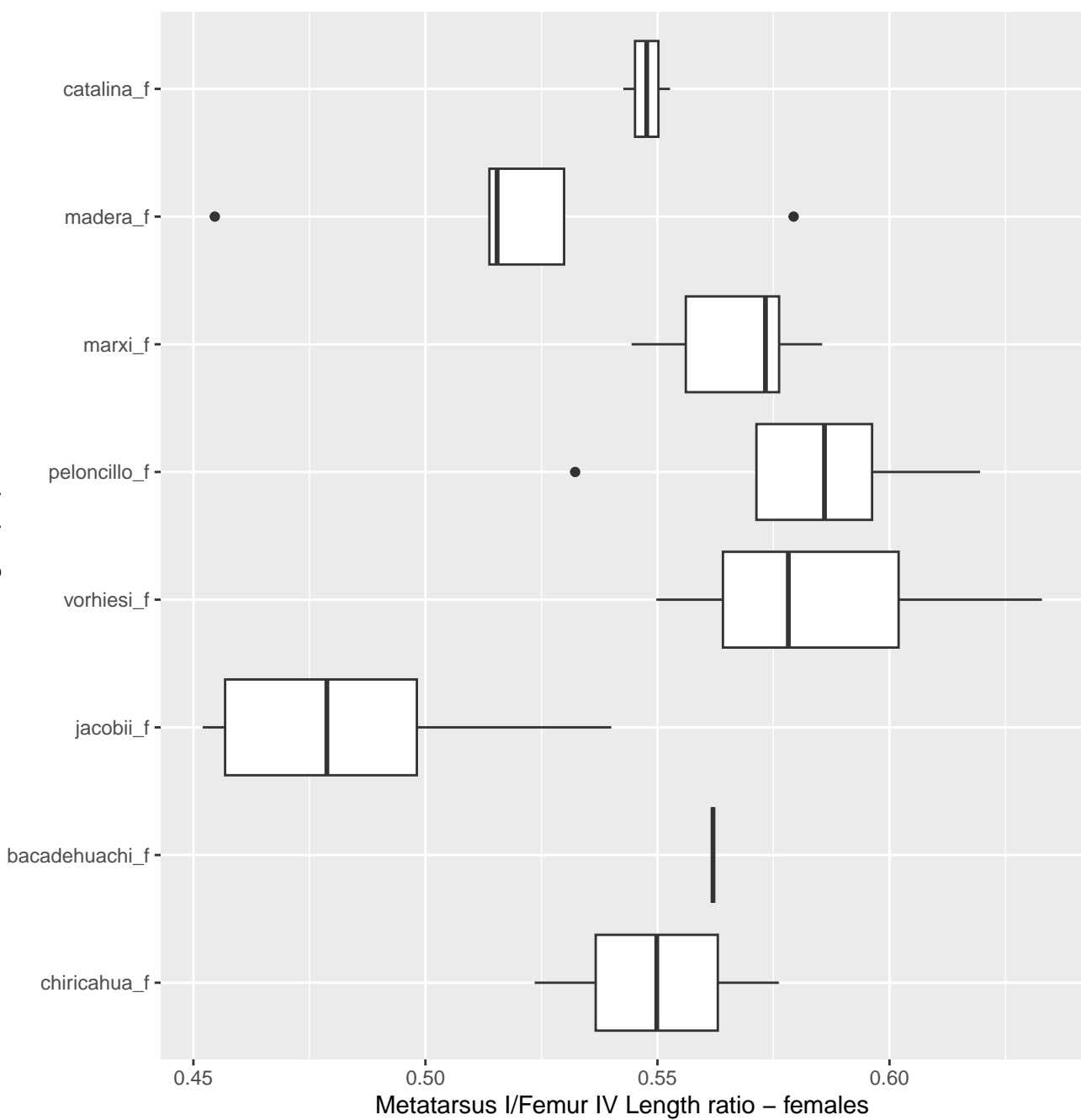

Supplement: Supplementary material 5 — Boxplots comparing various morphometric ratios based on measurements of mature male and female members of the Marxi species group [file zookeys-1210-061_article-125318__-s005.zip › Suppl5/Marxi_group_females/PDFs/M1F4_ratio_females.pdf]

Marxi group species

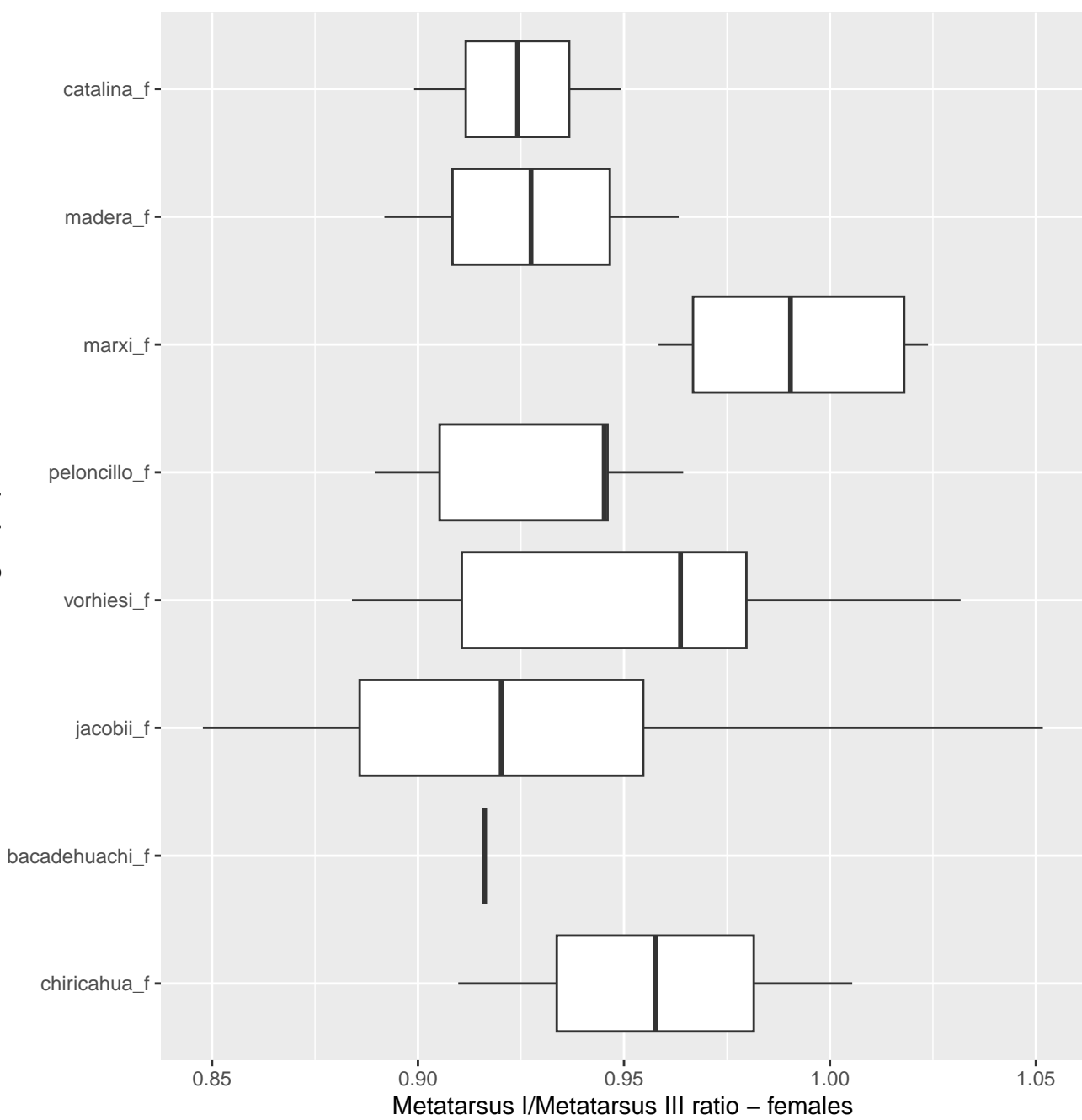

Supplement: Supplementary material 5 — Boxplots comparing various morphometric ratios based on measurements of mature male and female members of the Marxi species group [file zookeys-1210-061_article-125318__-s005.zip › Suppl5/Marxi_group_females/PDFs/M1M3_ratio_females.pdf]

Marxi group species

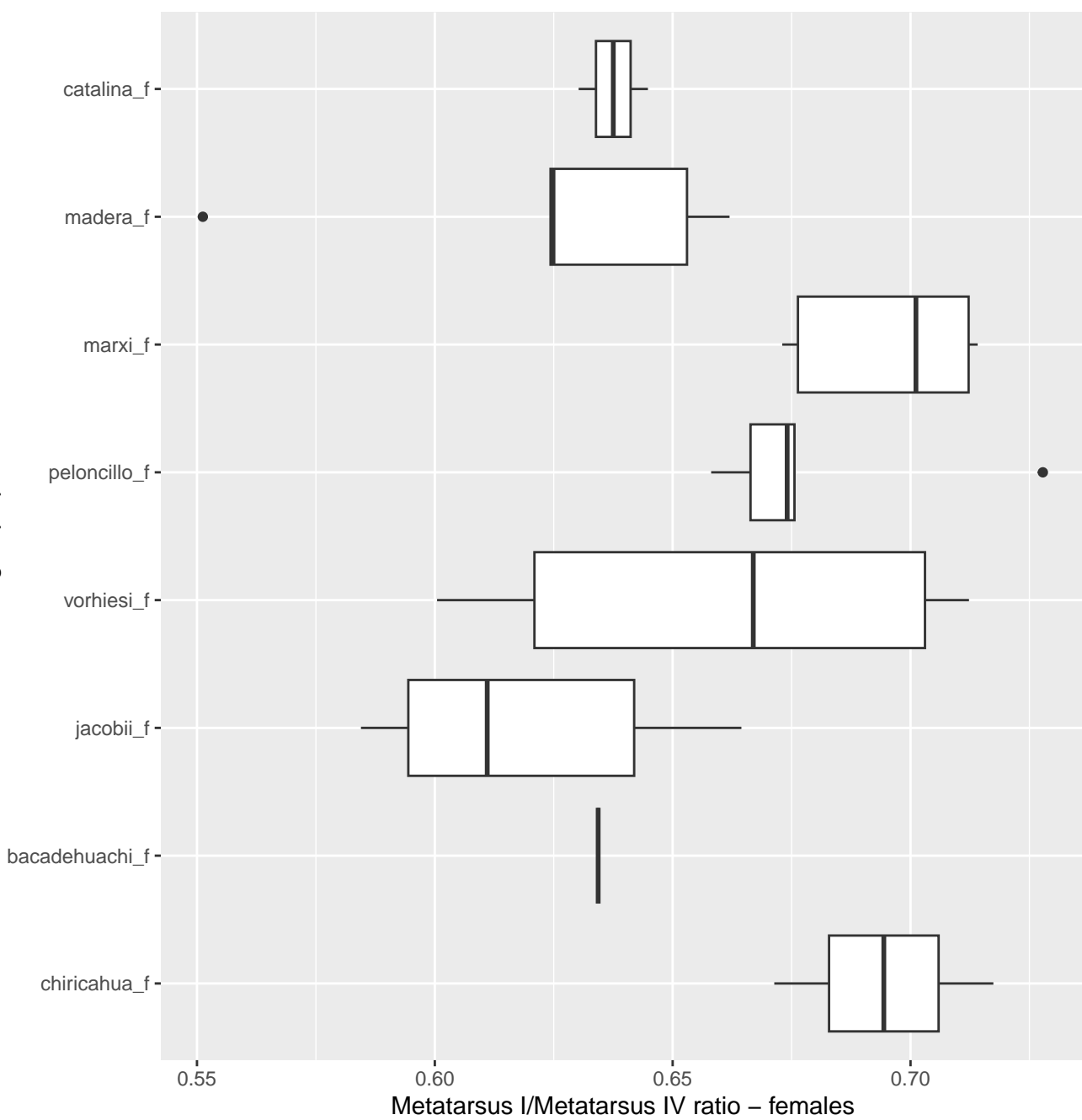

Supplement: Supplementary material 5 — Boxplots comparing various morphometric ratios based on measurements of mature male and female members of the Marxi species group [file zookeys-1210-061_article-125318__-s005.zip › Suppl5/Marxi_group_females/PDFs/M1M4_ratio_females.pdf]

Marxi group species

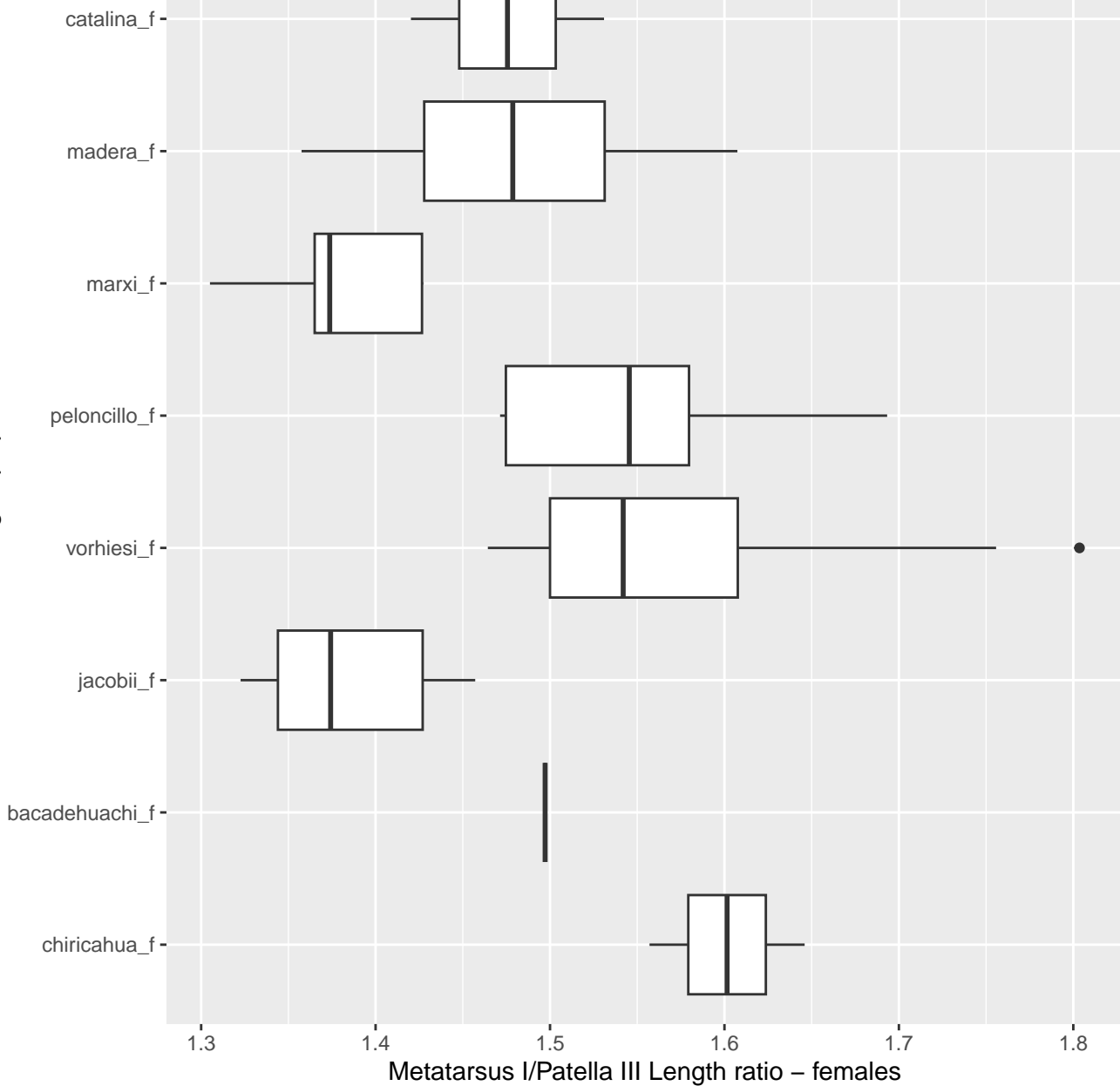

Supplement: Supplementary material 5 — Boxplots comparing various morphometric ratios based on measurements of mature male and female members of the Marxi species group [file zookeys-1210-061_article-125318__-s005.zip › Suppl5/Marxi_group_females/PDFs/M1P3_ratio_females.pdf]

Marxi group species

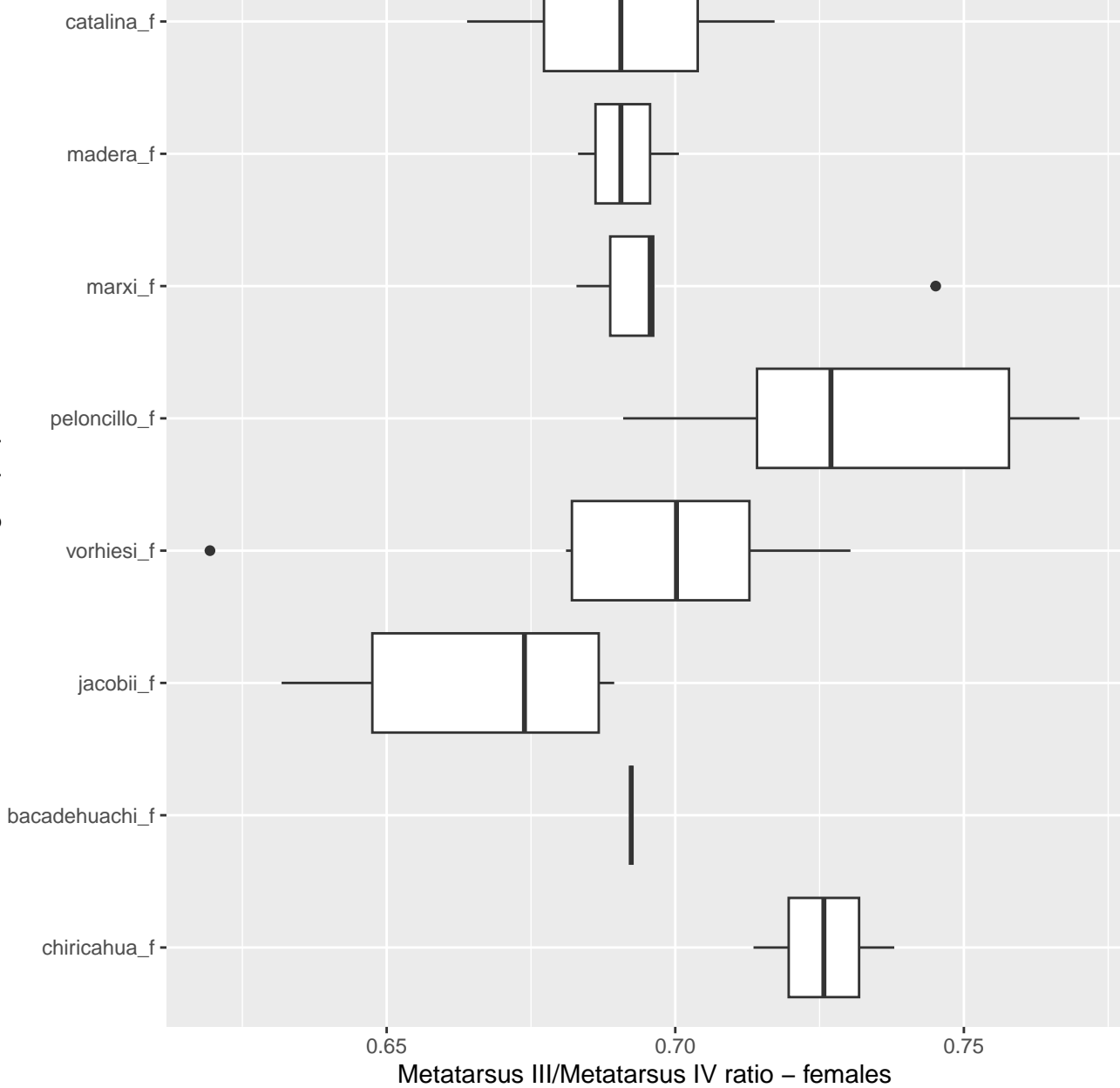

Supplement: Supplementary material 5 — Boxplots comparing various morphometric ratios based on measurements of mature male and female members of the Marxi species group [file zookeys-1210-061_article-125318__-s005.zip › Suppl5/Marxi_group_females/PDFs/M3M4_ratio_females.pdf]

Marxi group species

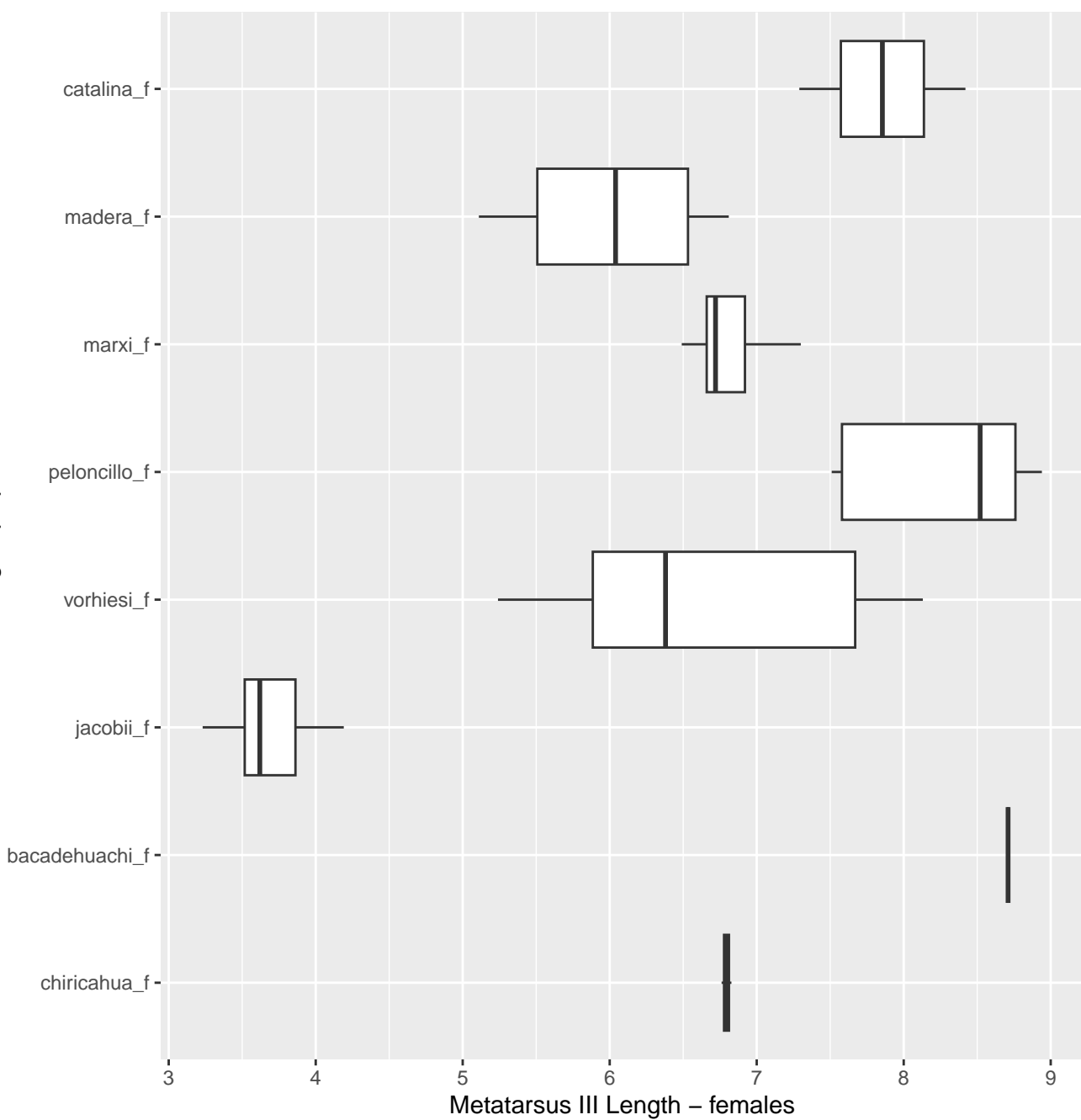

Supplement: Supplementary material 5 — Boxplots comparing various morphometric ratios based on measurements of mature male and female members of the Marxi species group [file zookeys-1210-061_article-125318__-s005.zip › Suppl5/Marxi_group_females/PDFs/M3_females.pdf]

Marxi group species

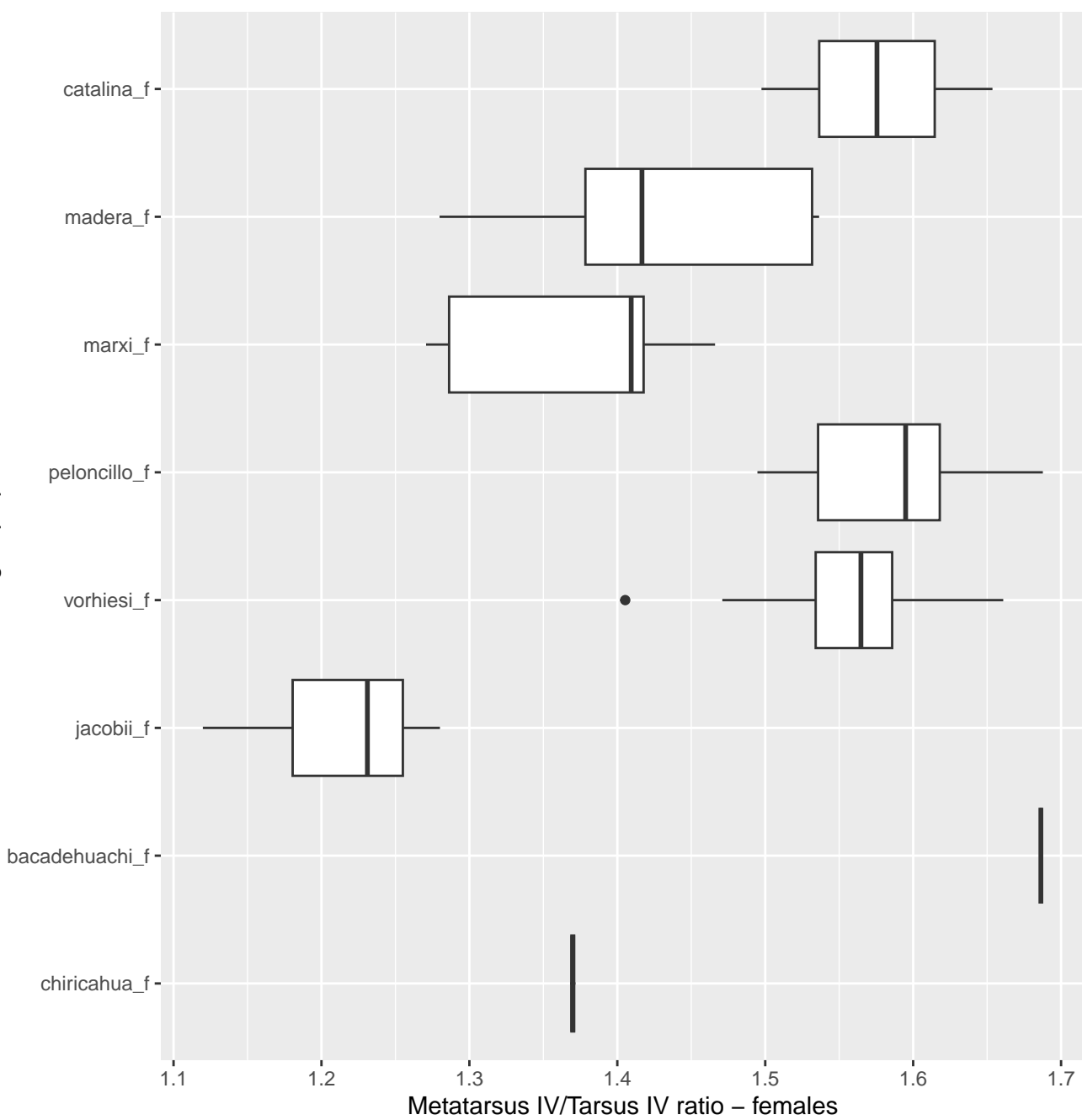

Supplement: Supplementary material 5 — Boxplots comparing various morphometric ratios based on measurements of mature male and female members of the Marxi species group [file zookeys-1210-061_article-125318__-s005.zip › Suppl5/Marxi_group_females/PDFs/M4A4_ratio_females.pdf]

Marxi group species

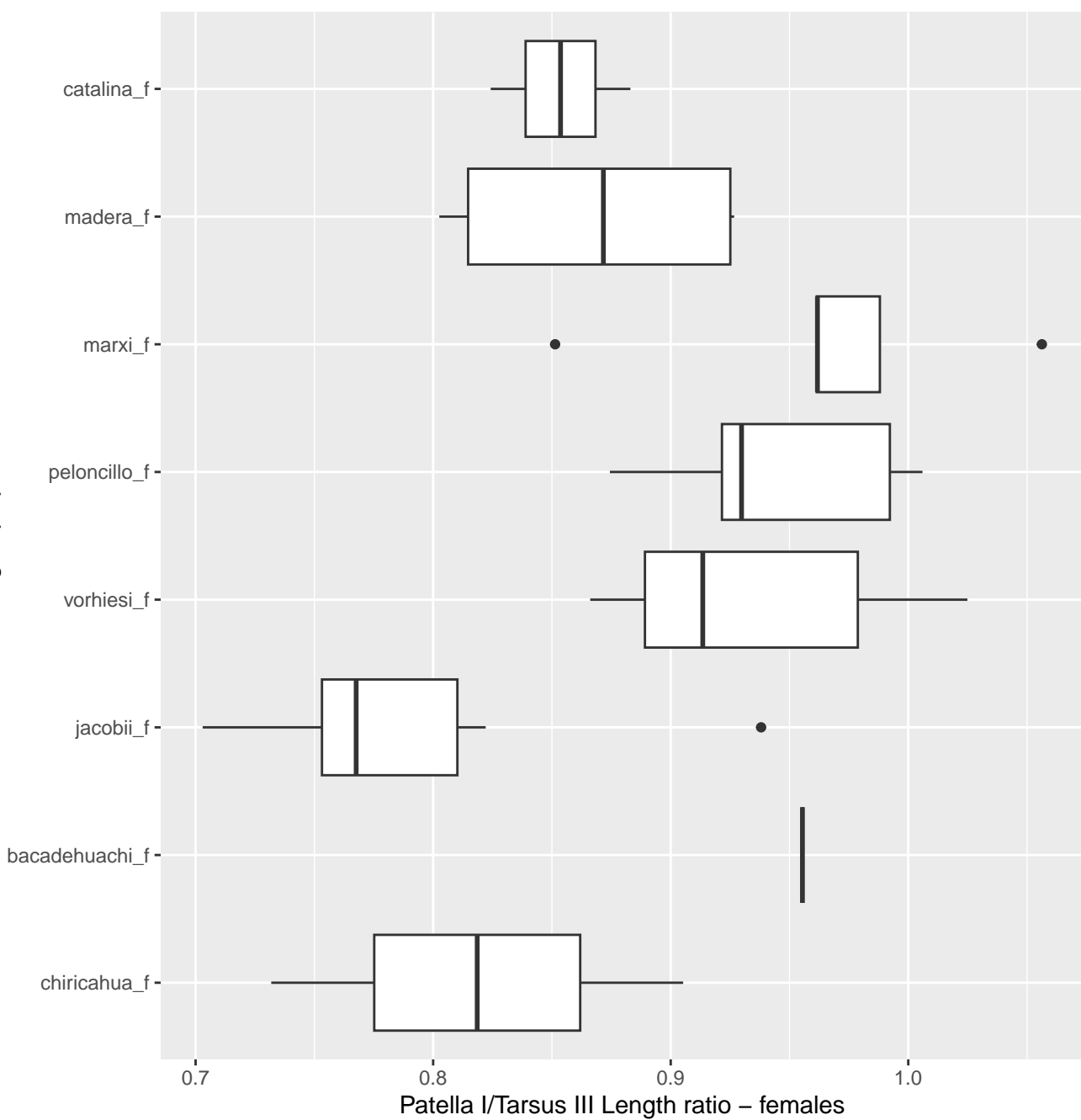

Supplement: Supplementary material 5 — Boxplots comparing various morphometric ratios based on measurements of mature male and female members of the Marxi species group [file zookeys-1210-061_article-125318__-s005.zip › Suppl5/Marxi_group_females/PDFs/P1A3_ratio_females.pdf]

Marxi group species

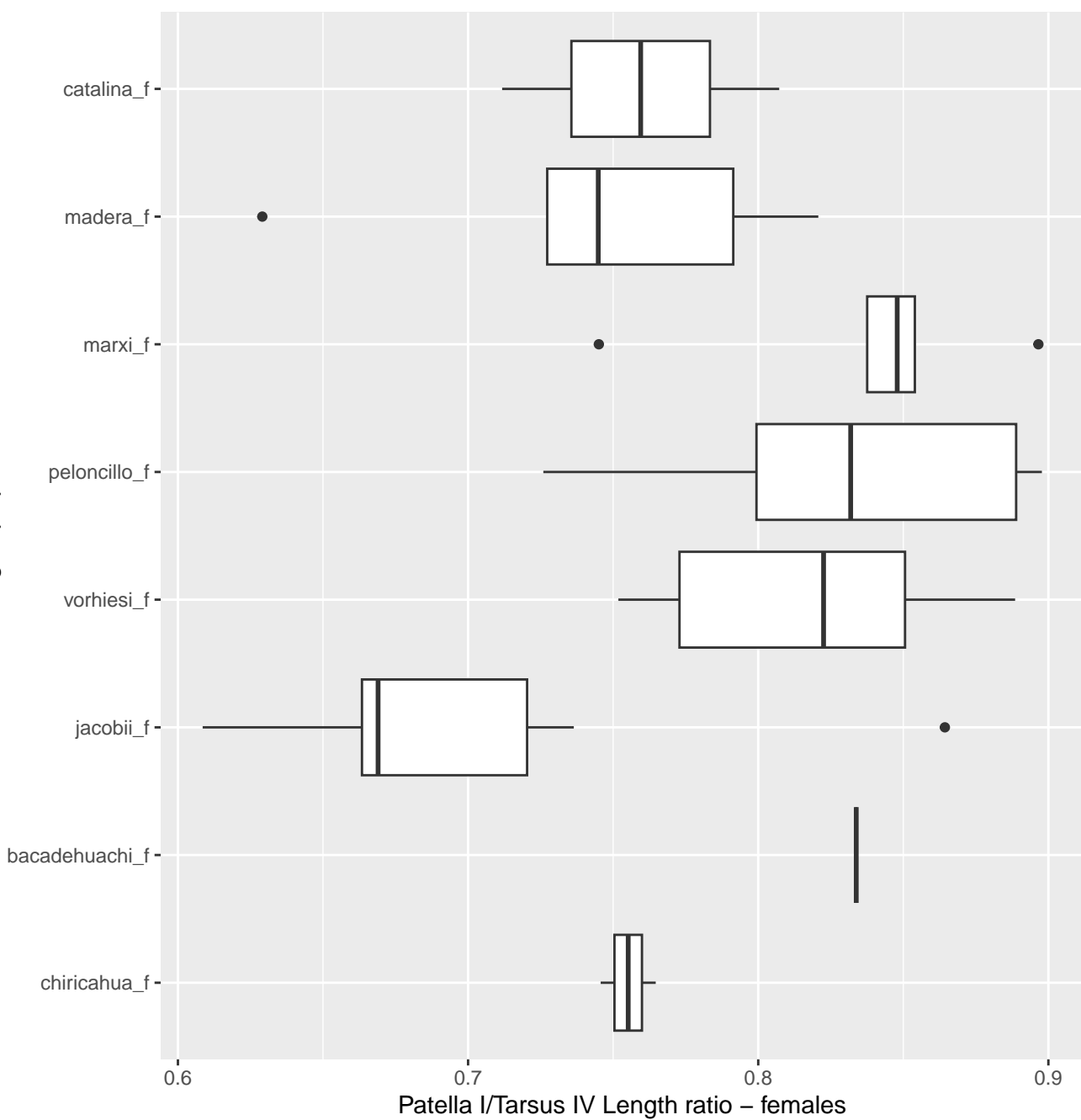

Supplement: Supplementary material 5 — Boxplots comparing various morphometric ratios based on measurements of mature male and female members of the Marxi species group [file zookeys-1210-061_article-125318__-s005.zip › Suppl5/Marxi_group_females/PDFs/P1A4_ratio_females.pdf]

Marxi group species

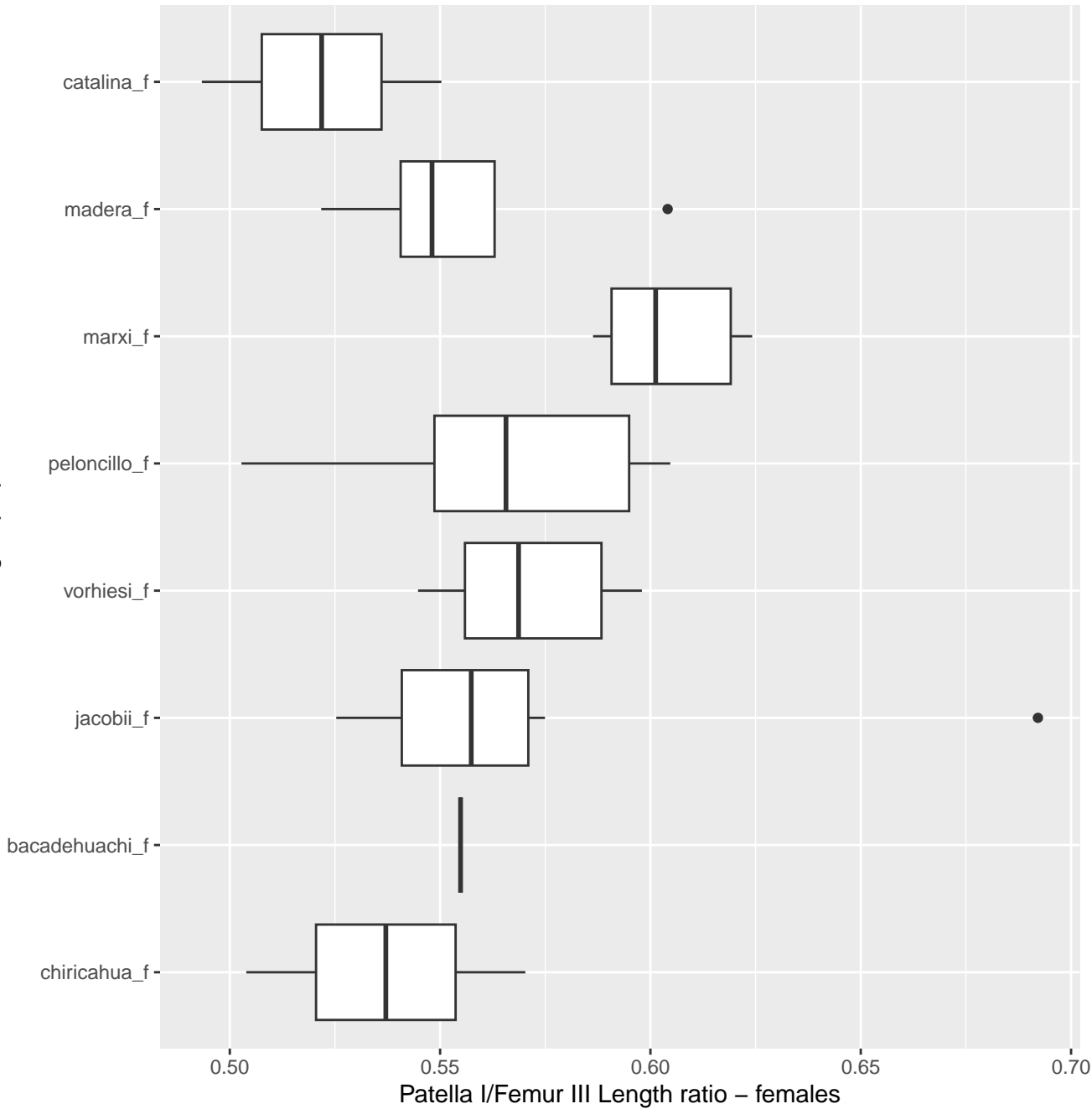

Supplement: Supplementary material 5 — Boxplots comparing various morphometric ratios based on measurements of mature male and female members of the Marxi species group [file zookeys-1210-061_article-125318__-s005.zip › Suppl5/Marxi_group_females/PDFs/P1F3_ratio_females.pdf]

Marxi group species

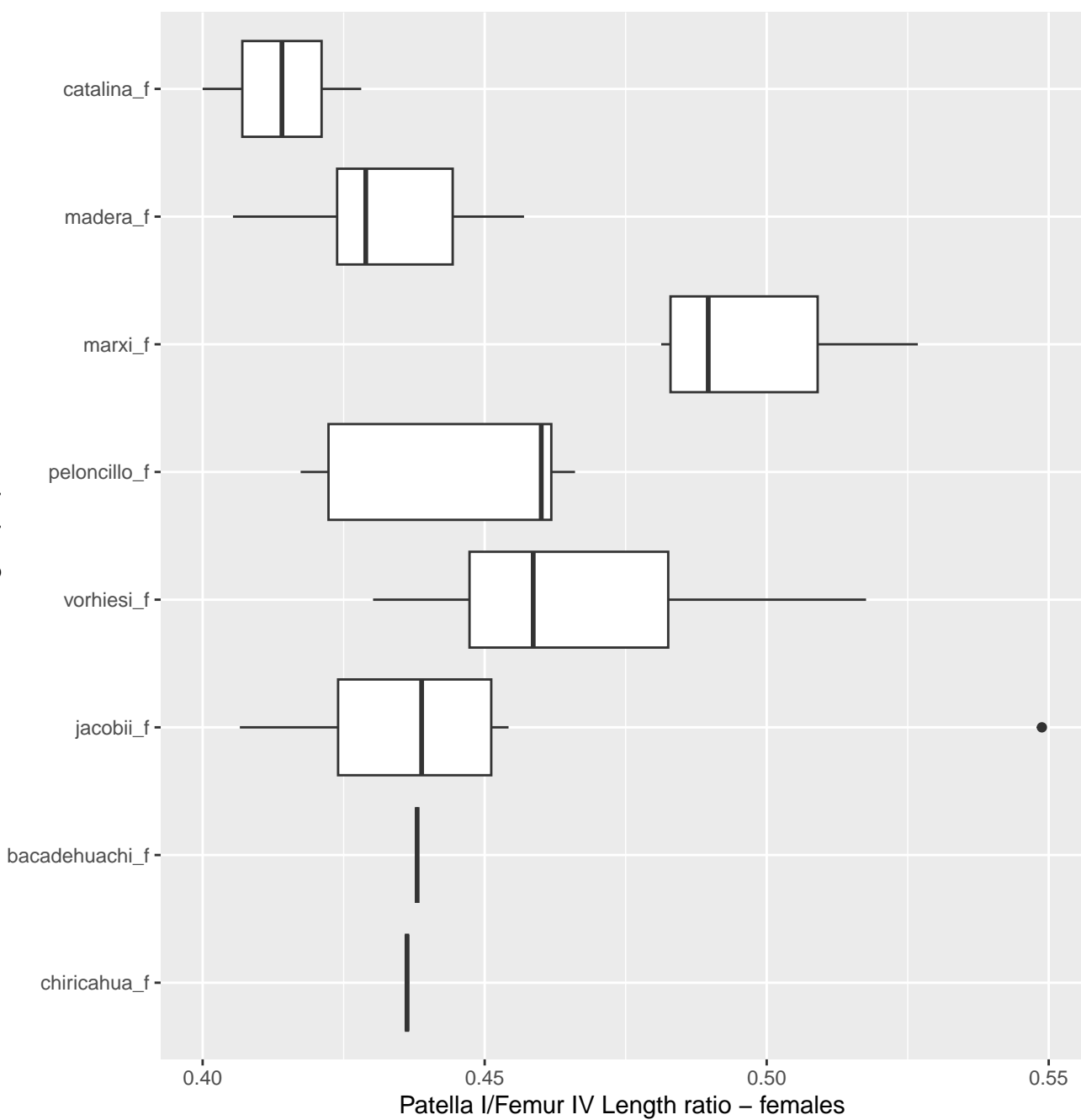

Supplement: Supplementary material 5 — Boxplots comparing various morphometric ratios based on measurements of mature male and female members of the Marxi species group [file zookeys-1210-061_article-125318__-s005.zip › Suppl5/Marxi_group_females/PDFs/P1F4_ratio_females.pdf]

Marxi group species

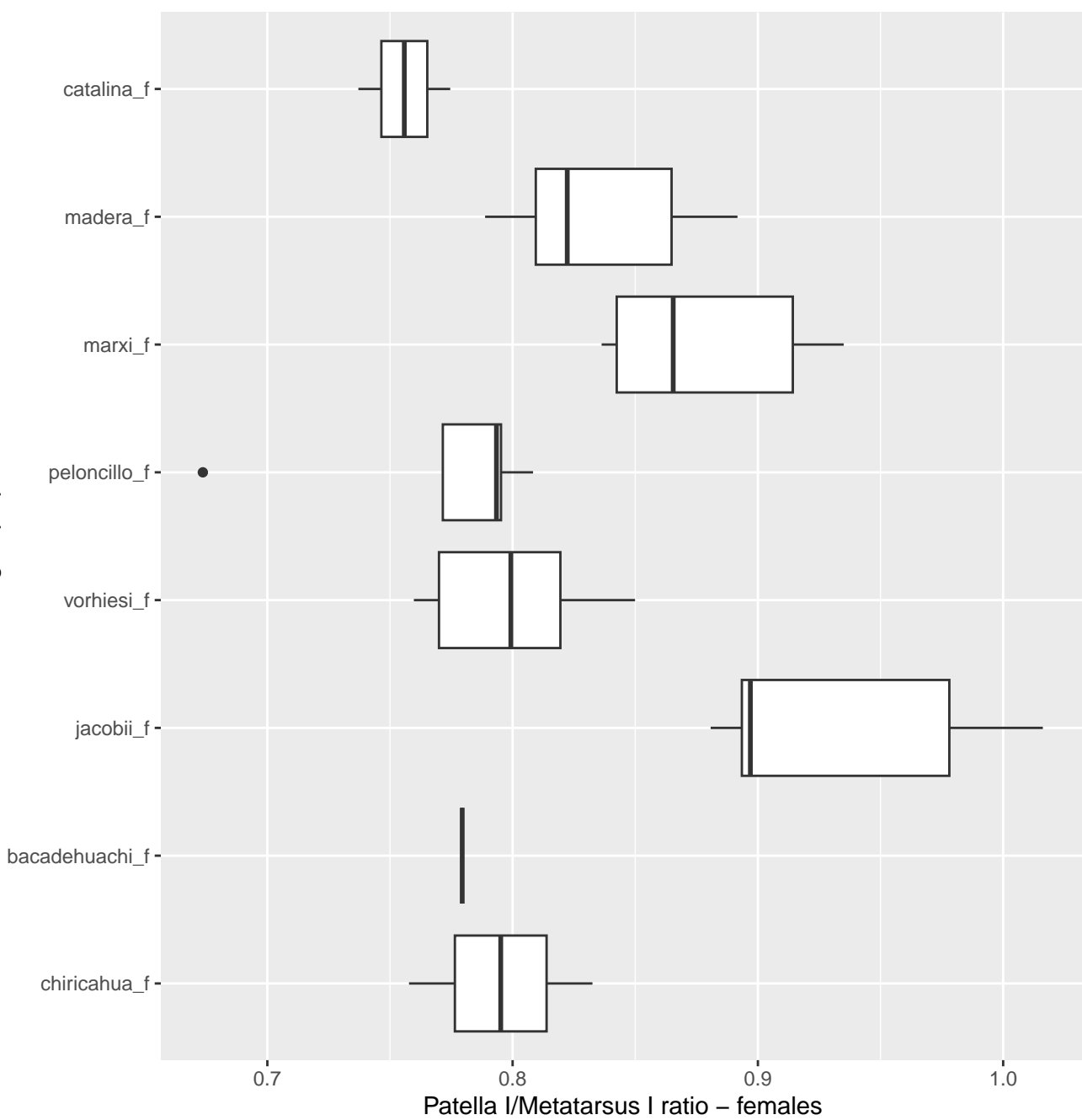

Supplement: Supplementary material 5 — Boxplots comparing various morphometric ratios based on measurements of mature male and female members of the Marxi species group [file zookeys-1210-061_article-125318__-s005.zip › Suppl5/Marxi_group_females/PDFs/P1M1_ratio_females.pdf]

Marxi group species

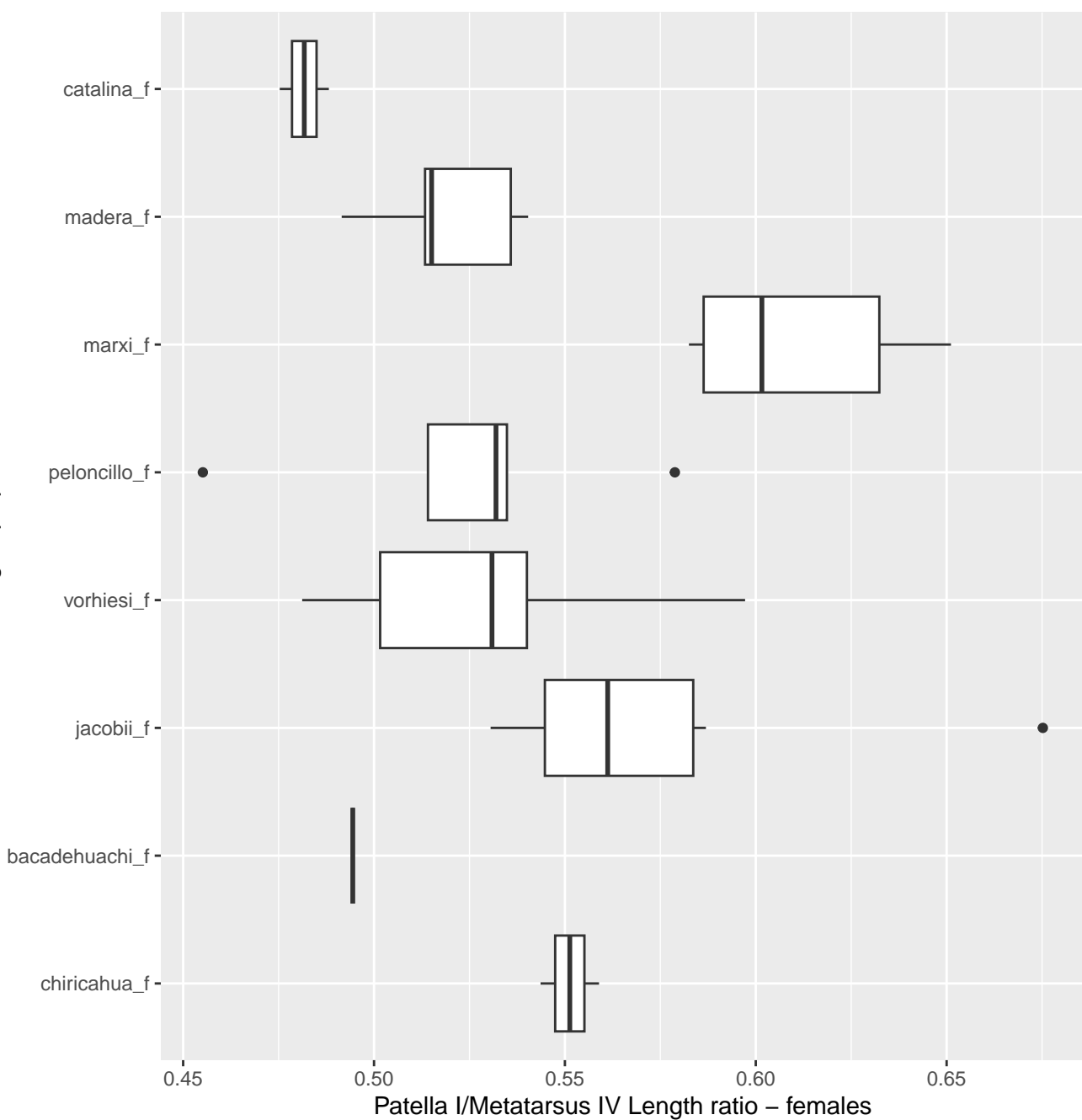

Supplement: Supplementary material 5 — Boxplots comparing various morphometric ratios based on measurements of mature male and female members of the Marxi species group [file zookeys-1210-061_article-125318__-s005.zip › Suppl5/Marxi_group_females/PDFs/P1M4_ratio_females.pdf]

Marxi group species

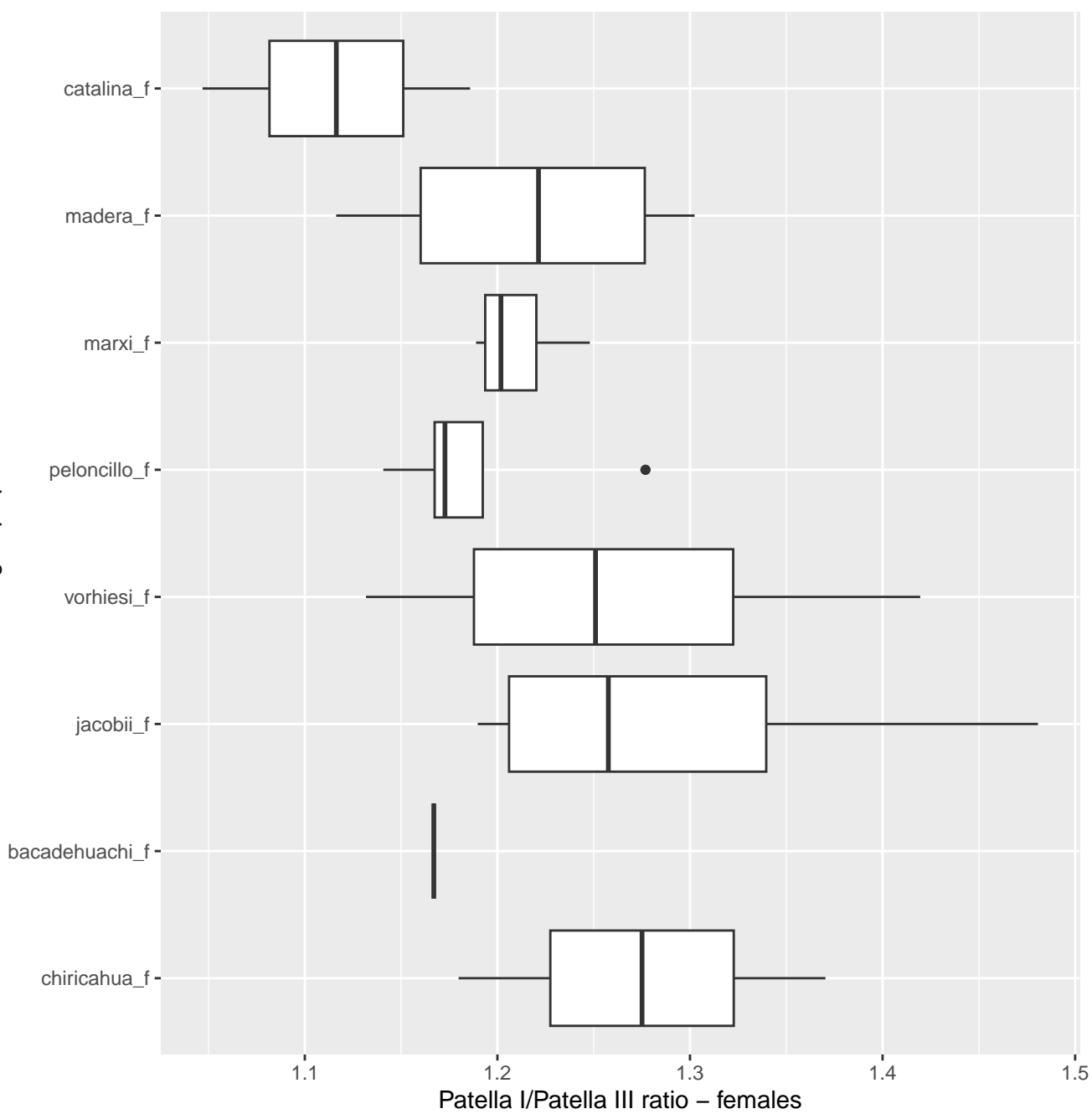

Supplement: Supplementary material 5 — Boxplots comparing various morphometric ratios based on measurements of mature male and female members of the Marxi species group [file zookeys-1210-061_article-125318__-s005.zip › Suppl5/Marxi_group_females/PDFs/P1P3_ratio_females.pdf]

Marxi group species

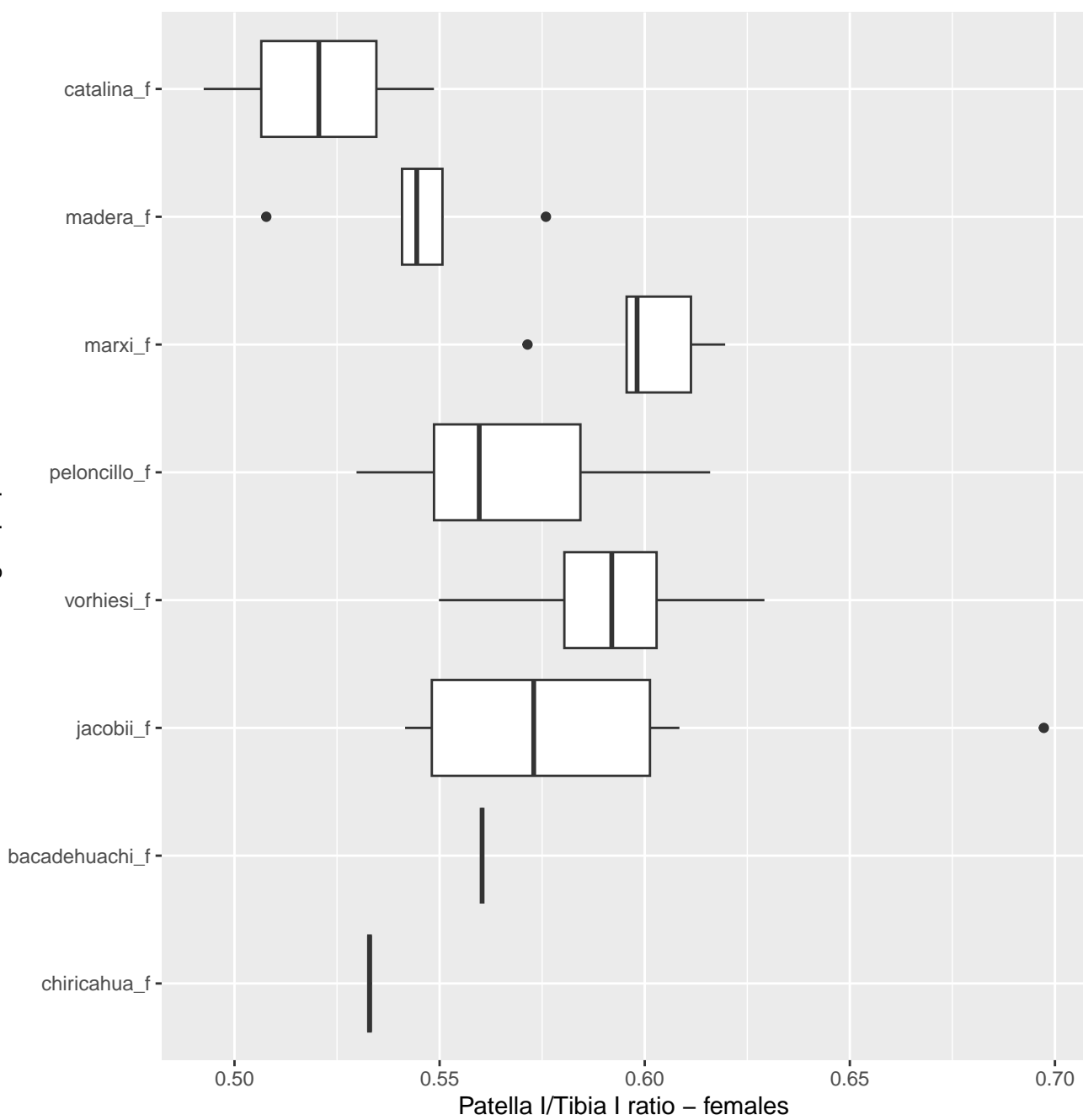

Supplement: Supplementary material 5 — Boxplots comparing various morphometric ratios based on measurements of mature male and female members of the Marxi species group [file zookeys-1210-061_article-125318__-s005.zip › Suppl5/Marxi_group_females/PDFs/P1T1_ratio_females.pdf]

Marxi group species

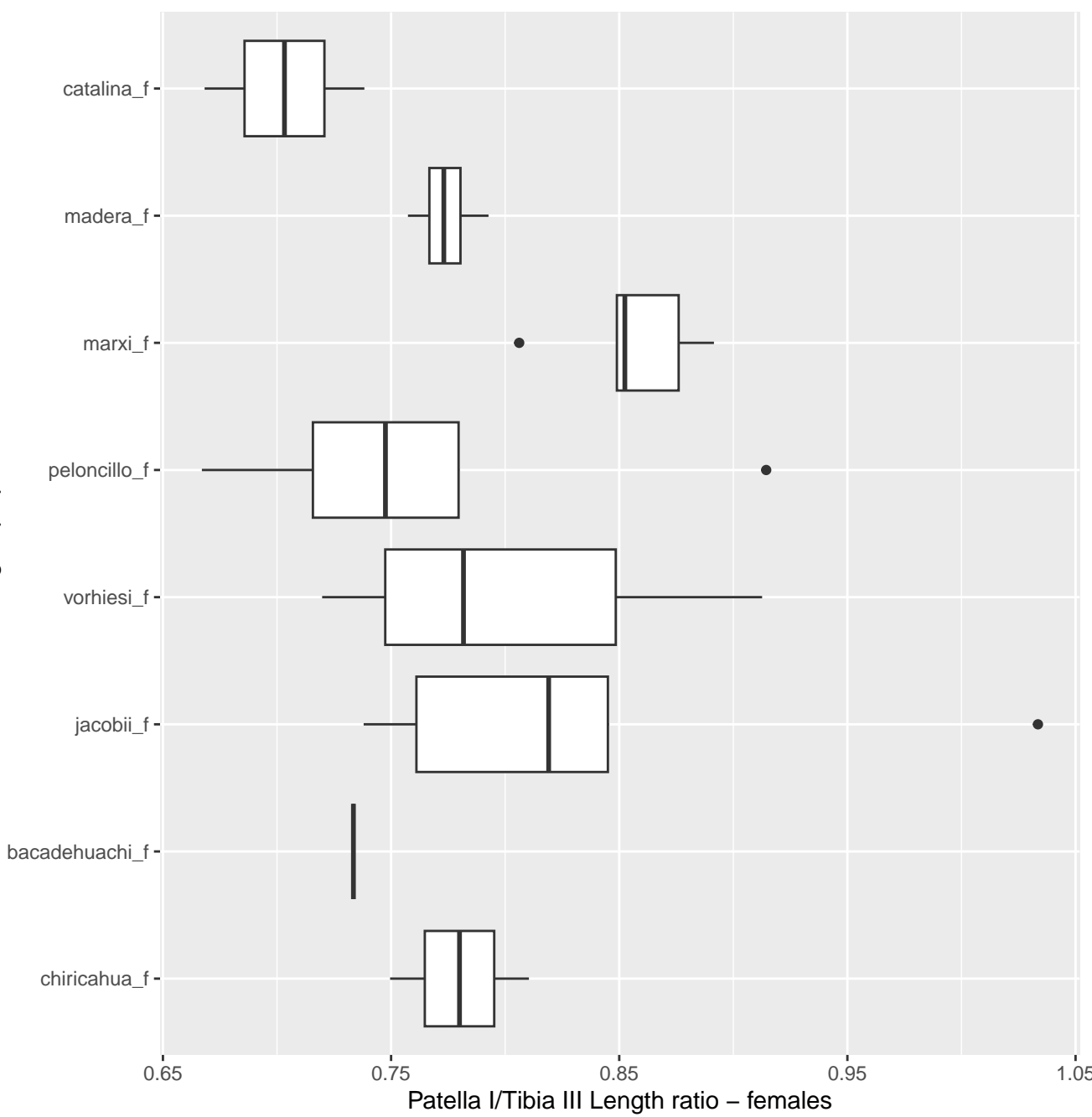

Supplement: Supplementary material 5 — Boxplots comparing various morphometric ratios based on measurements of mature male and female members of the Marxi species group [file zookeys-1210-061_article-125318__-s005.zip › Suppl5/Marxi_group_females/PDFs/P1T3_ratio_females.pdf]

Marxi group species

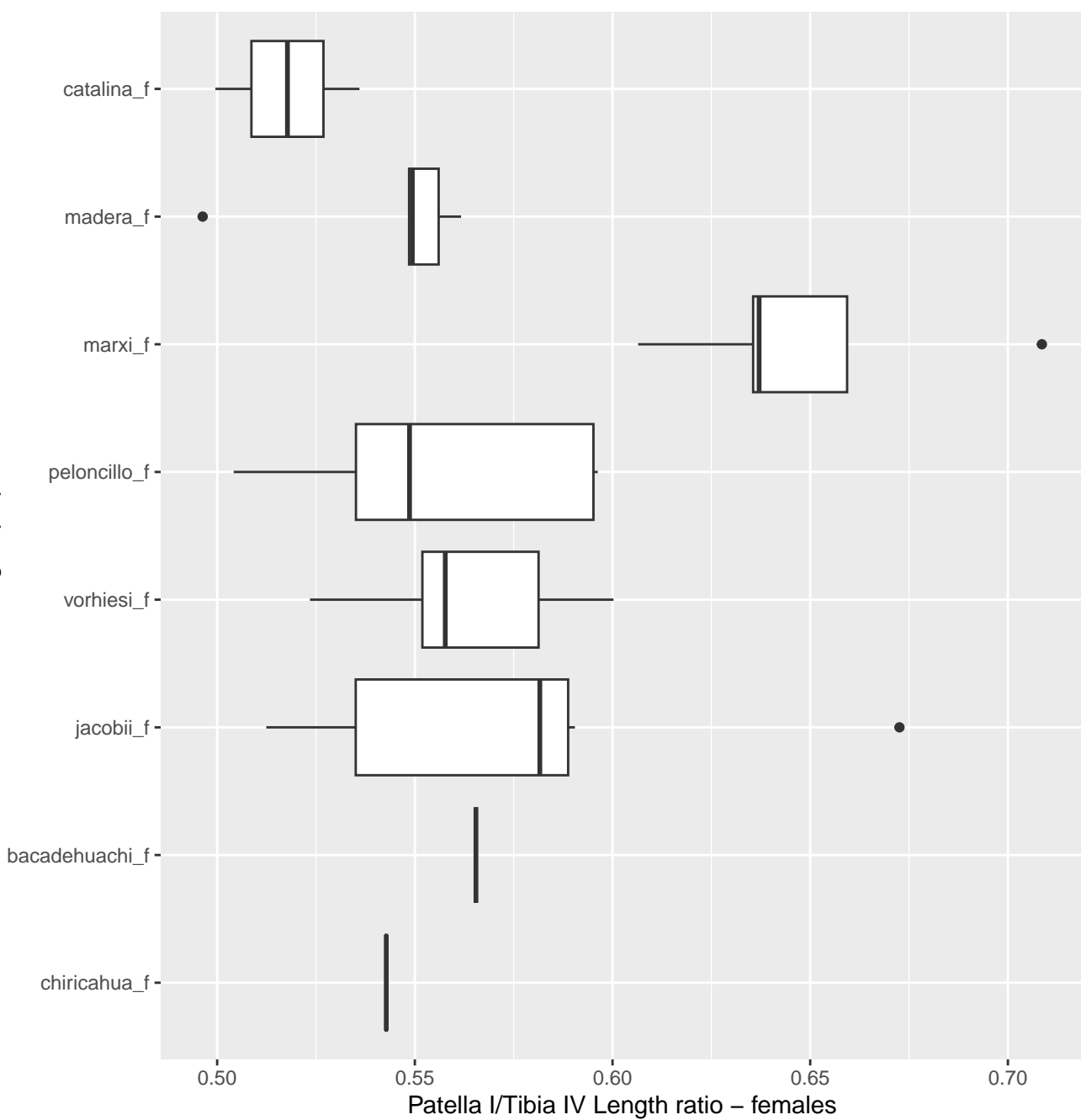

Supplement: Supplementary material 5 — Boxplots comparing various morphometric ratios based on measurements of mature male and female members of the Marxi species group [file zookeys-1210-061_article-125318__-s005.zip › Suppl5/Marxi_group_females/PDFs/P1T4_ratio_females.pdf]

Marxi group species

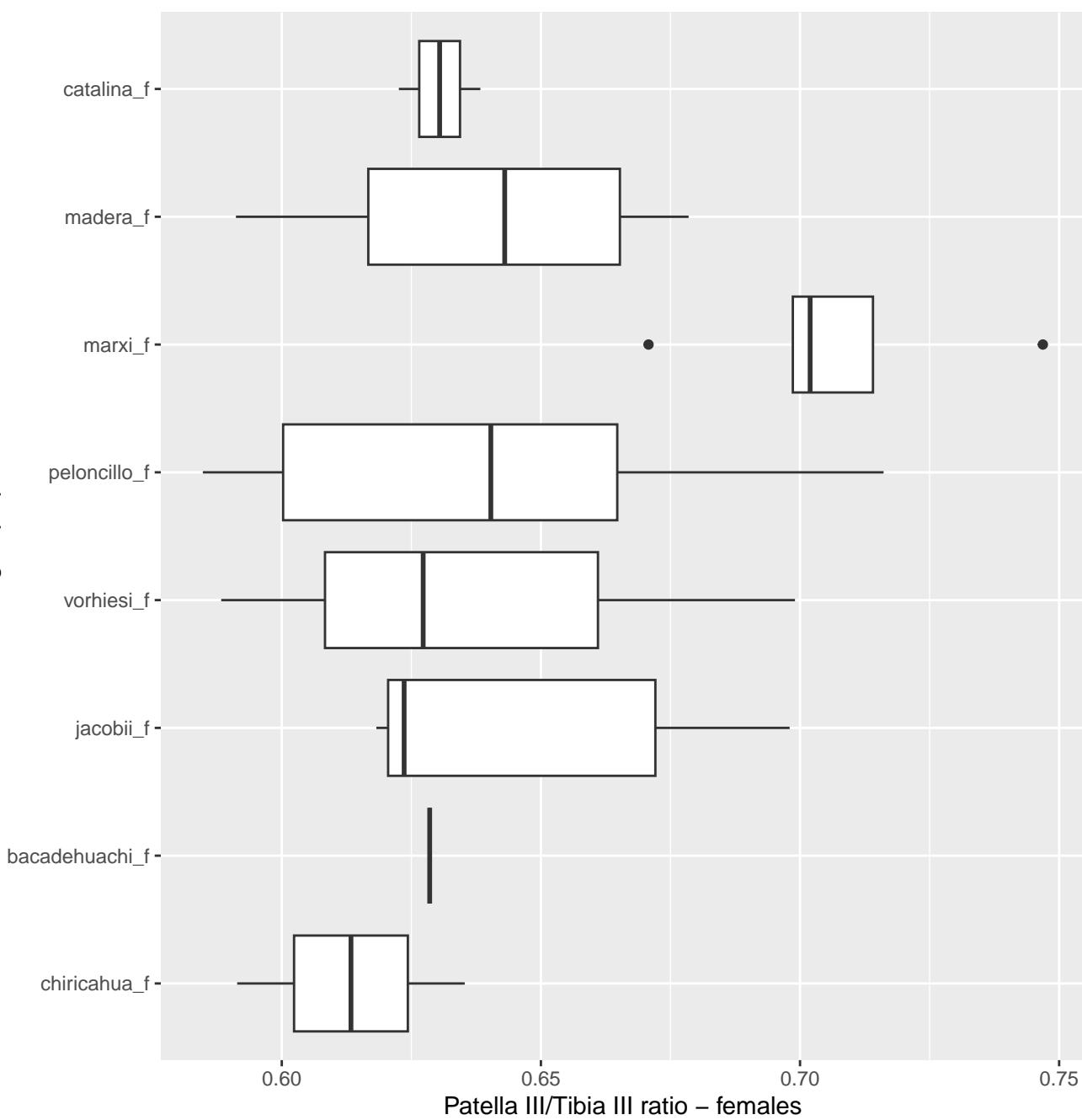

Supplement: Supplementary material 5 — Boxplots comparing various morphometric ratios based on measurements of mature male and female members of the Marxi species group [file zookeys-1210-061_article-125318__-s005.zip › Suppl5/Marxi_group_females/PDFs/P3T3_ratio_females.pdf]

Marxi group species

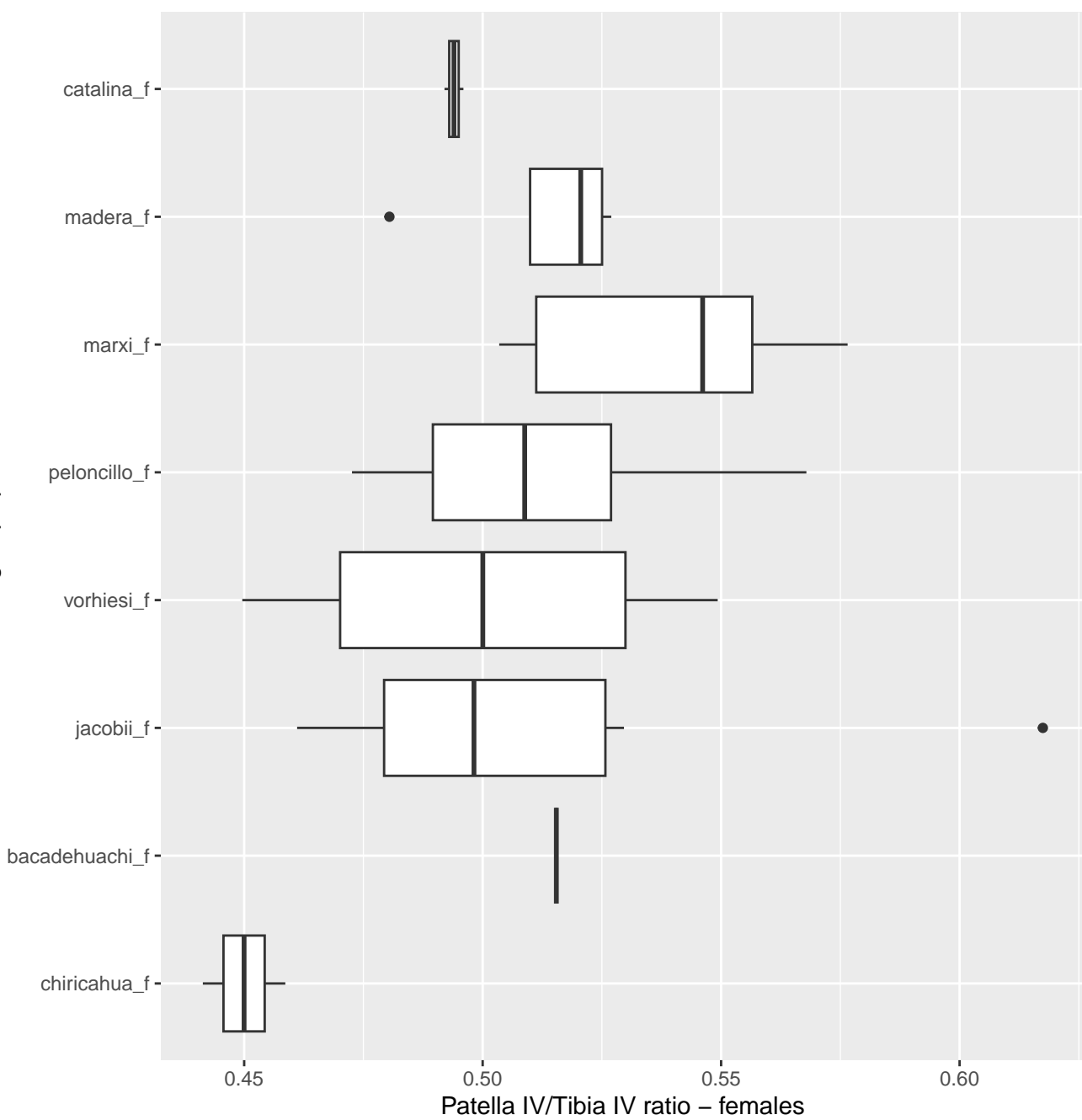

Supplement: Supplementary material 5 — Boxplots comparing various morphometric ratios based on measurements of mature male and female members of the Marxi species group [file zookeys-1210-061_article-125318__-s005.zip › Suppl5/Marxi_group_females/PDFs/P4T4_ratio_females.pdf]

Marxi group species

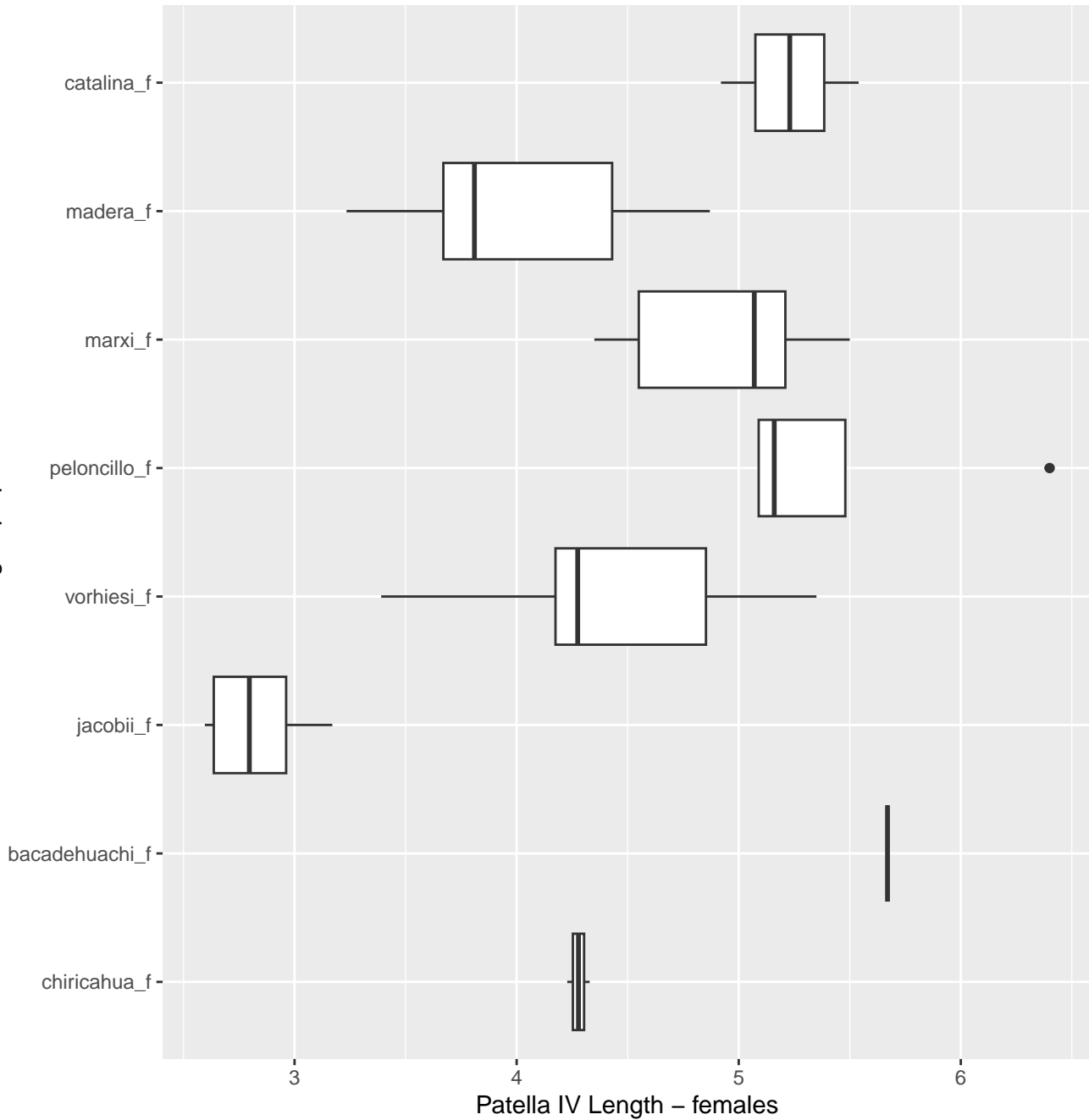

Supplement: Supplementary material 5 — Boxplots comparing various morphometric ratios based on measurements of mature male and female members of the Marxi species group [file zookeys-1210-061_article-125318__-s005.zip › Suppl5/Marxi_group_females/PDFs/P4_females.pdf]

Marxi species group

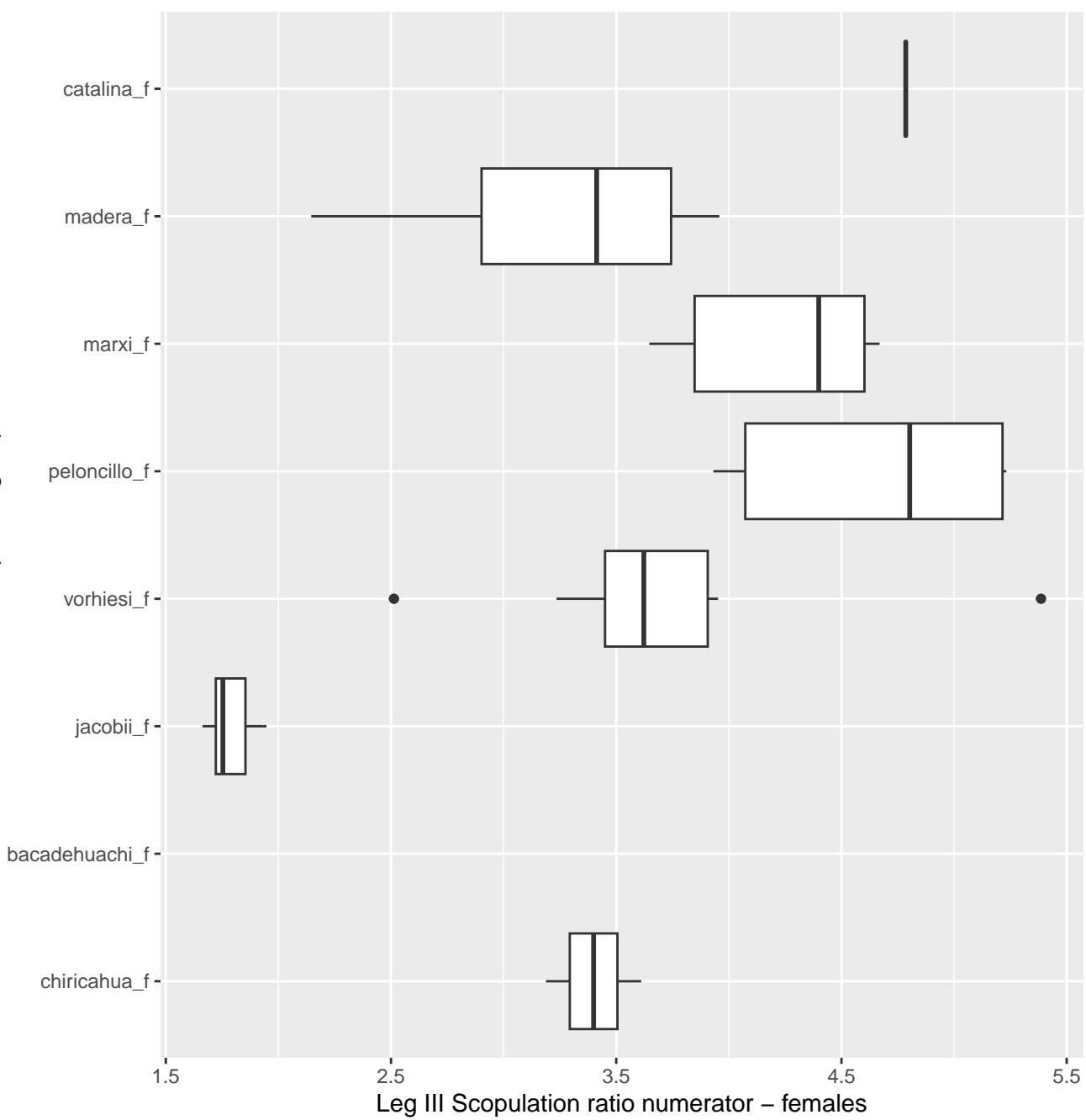

Supplement: Supplementary material 5 — Boxplots comparing various morphometric ratios based on measurements of mature male and female members of the Marxi species group [file zookeys-1210-061_article-125318__-s005.zip › Suppl5/Marxi_group_females/PDFs/SC3_n_females.pdf]

Marxi group species

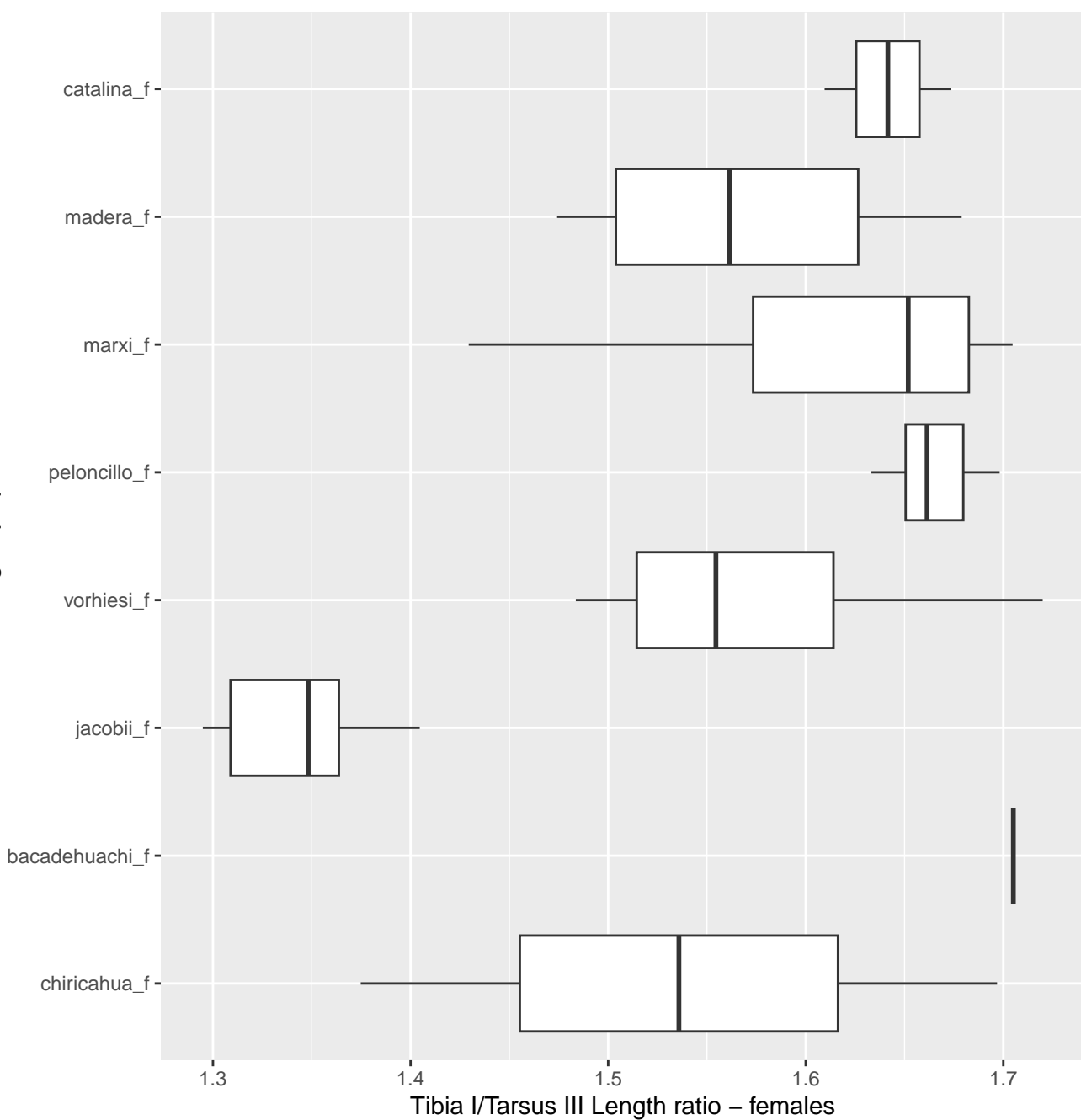

Supplement: Supplementary material 5 — Boxplots comparing various morphometric ratios based on measurements of mature male and female members of the Marxi species group [file zookeys-1210-061_article-125318__-s005.zip › Suppl5/Marxi_group_females/PDFs/T1A3_ratio_females.pdf]

Marxi group species

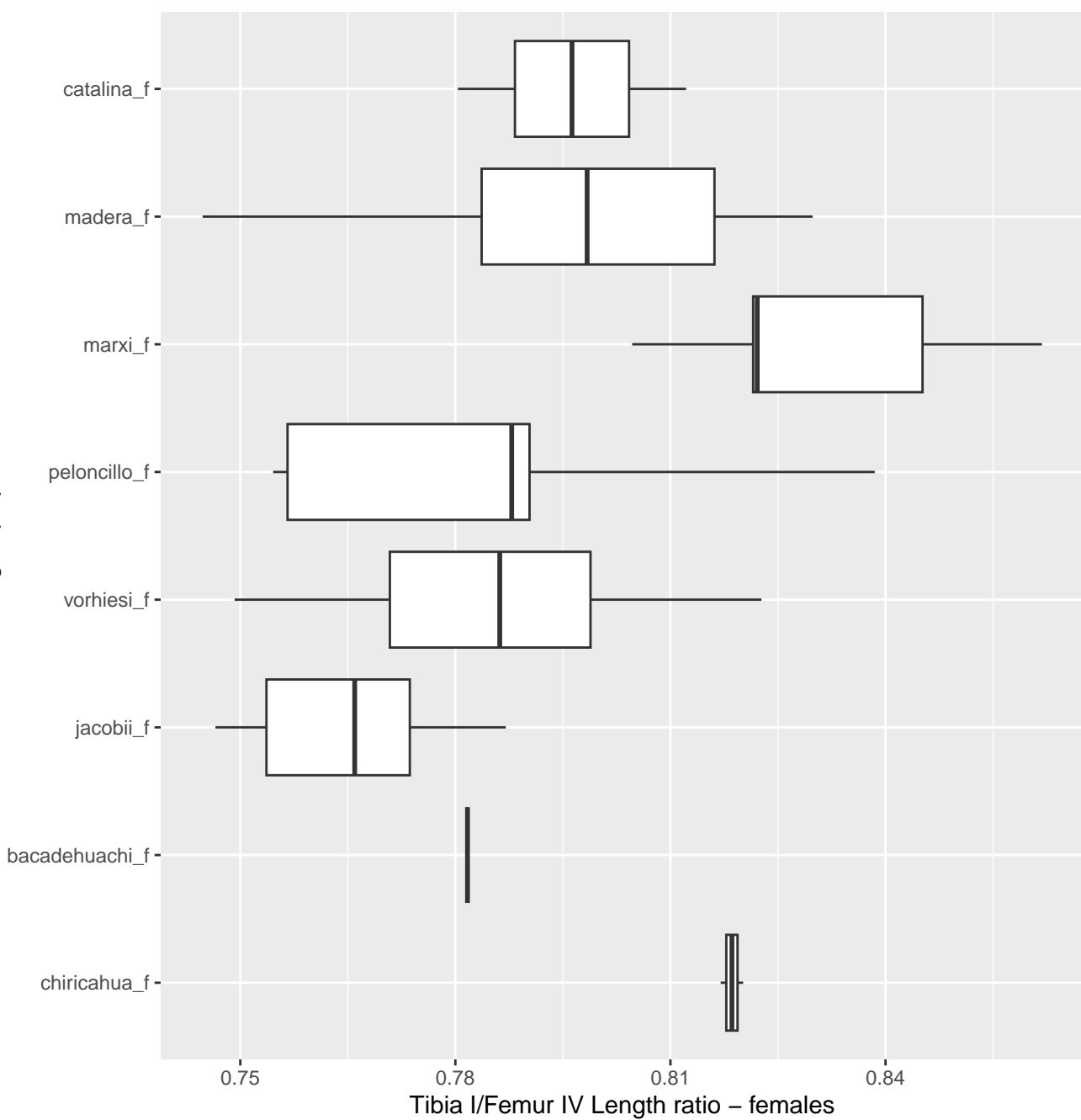

Supplement: Supplementary material 5 — Boxplots comparing various morphometric ratios based on measurements of mature male and female members of the Marxi species group [file zookeys-1210-061_article-125318__-s005.zip › Suppl5/Marxi_group_females/PDFs/T1F4_ratio_females.pdf]

Marxi group species

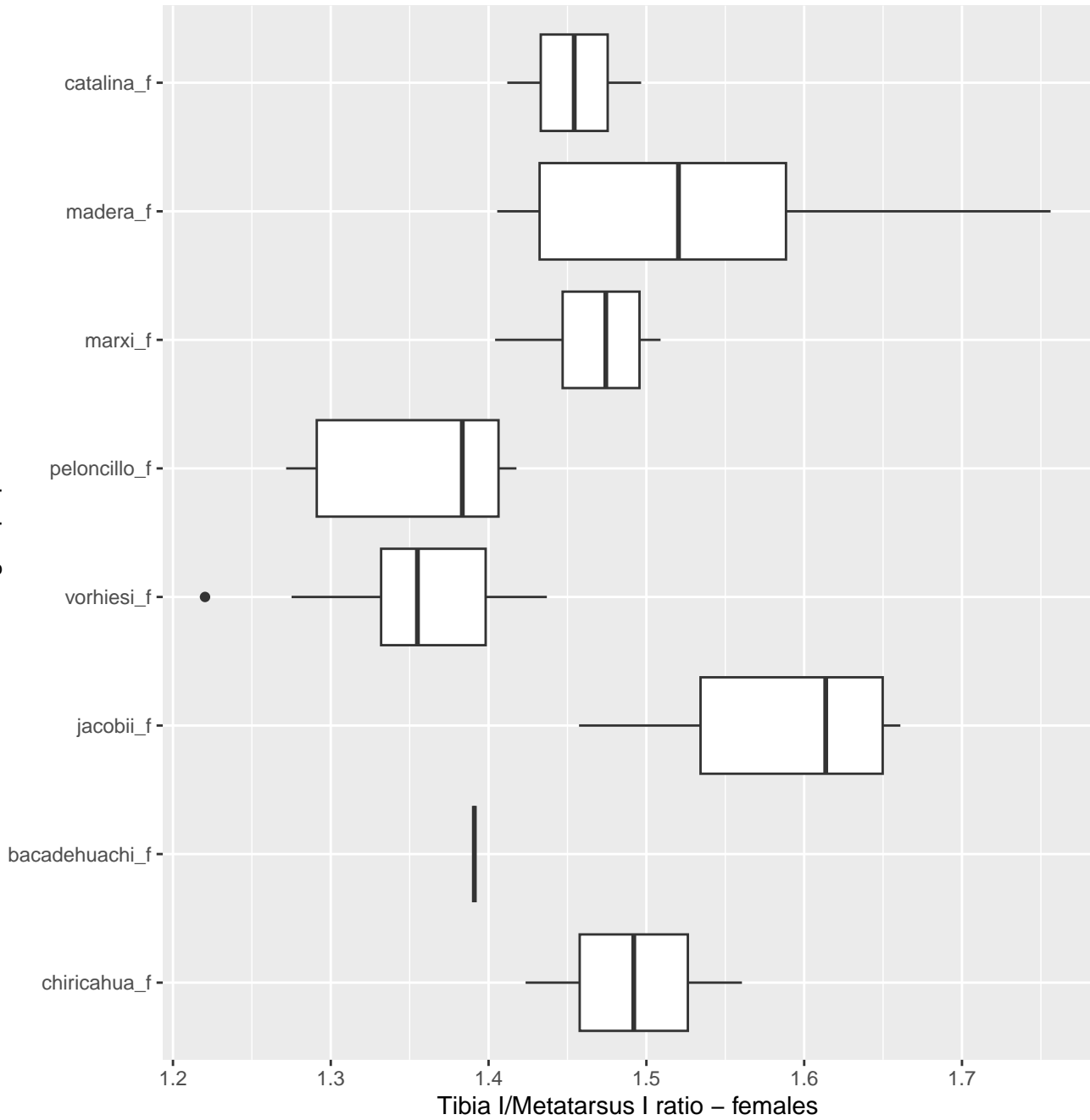

Supplement: Supplementary material 5 — Boxplots comparing various morphometric ratios based on measurements of mature male and female members of the Marxi species group [file zookeys-1210-061_article-125318__-s005.zip › Suppl5/Marxi_group_females/PDFs/T1M1_ratio_females.pdf]

Marxi group species

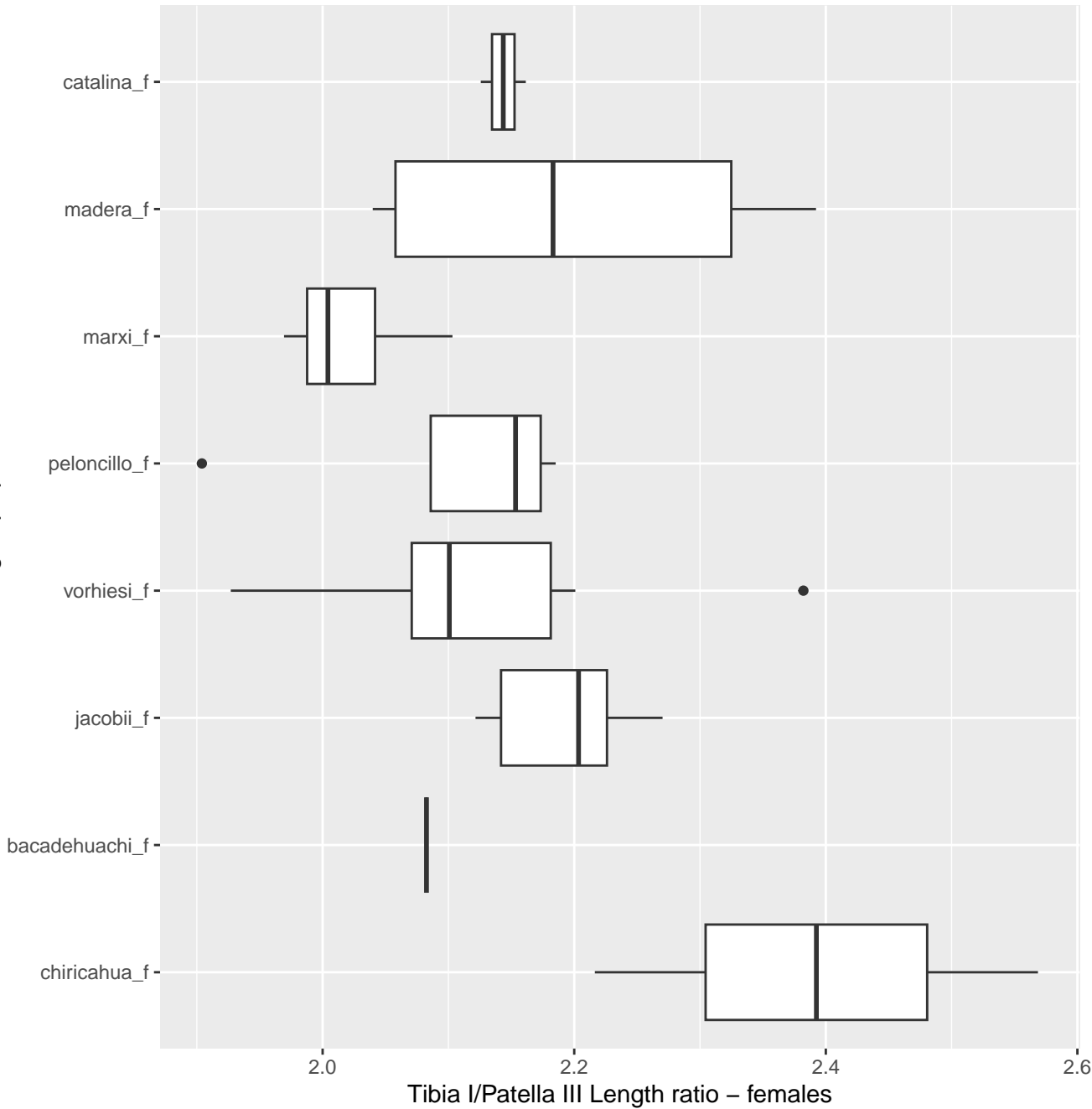

Supplement: Supplementary material 5 — Boxplots comparing various morphometric ratios based on measurements of mature male and female members of the Marxi species group [file zookeys-1210-061_article-125318__-s005.zip › Suppl5/Marxi_group_females/PDFs/T1P3_ratio_females.pdf]

Marxi group species

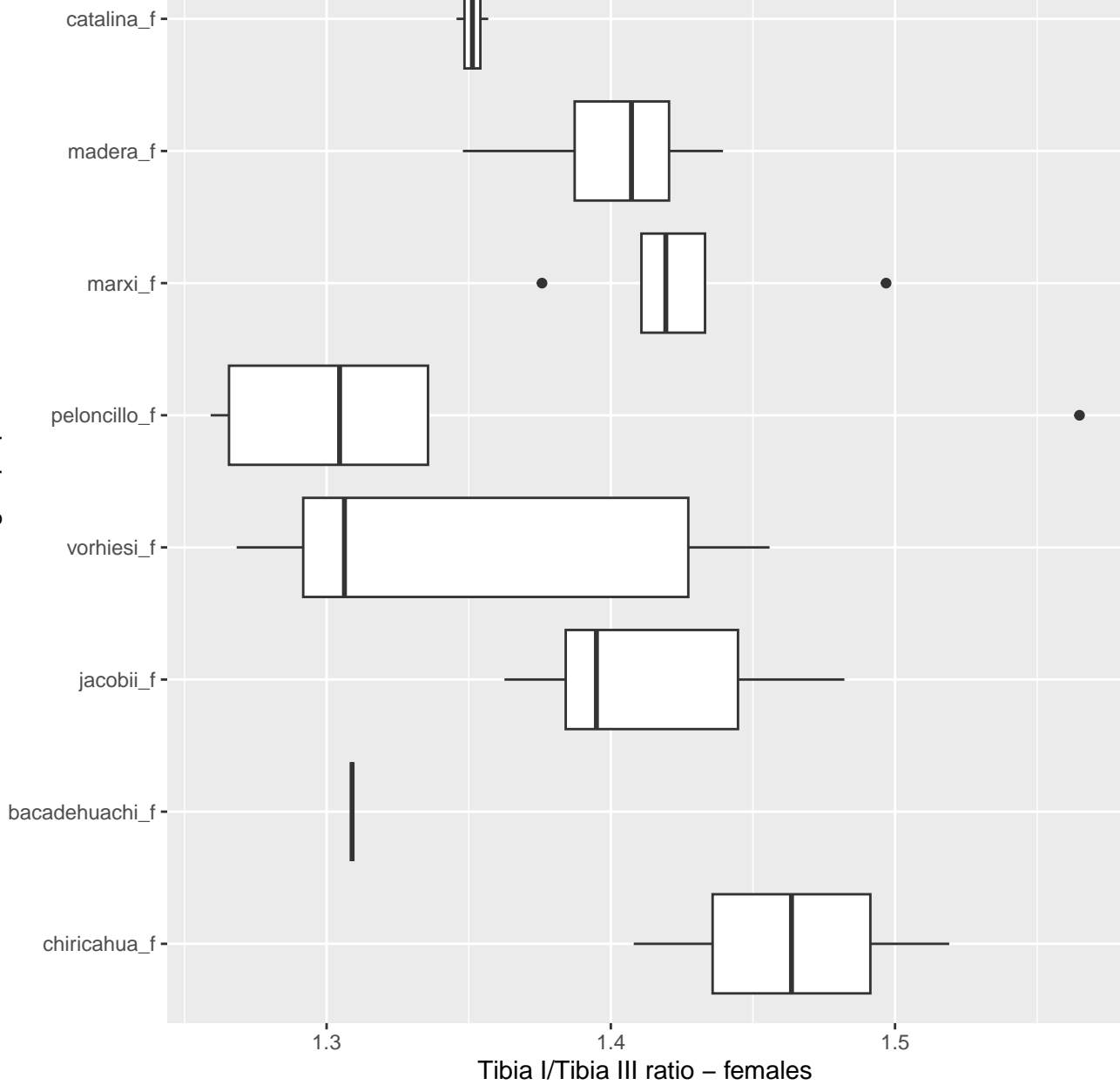

Supplement: Supplementary material 5 — Boxplots comparing various morphometric ratios based on measurements of mature male and female members of the Marxi species group [file zookeys-1210-061_article-125318__-s005.zip › Suppl5/Marxi_group_females/PDFs/T1T3_ratio_females.pdf]

Marxi group species

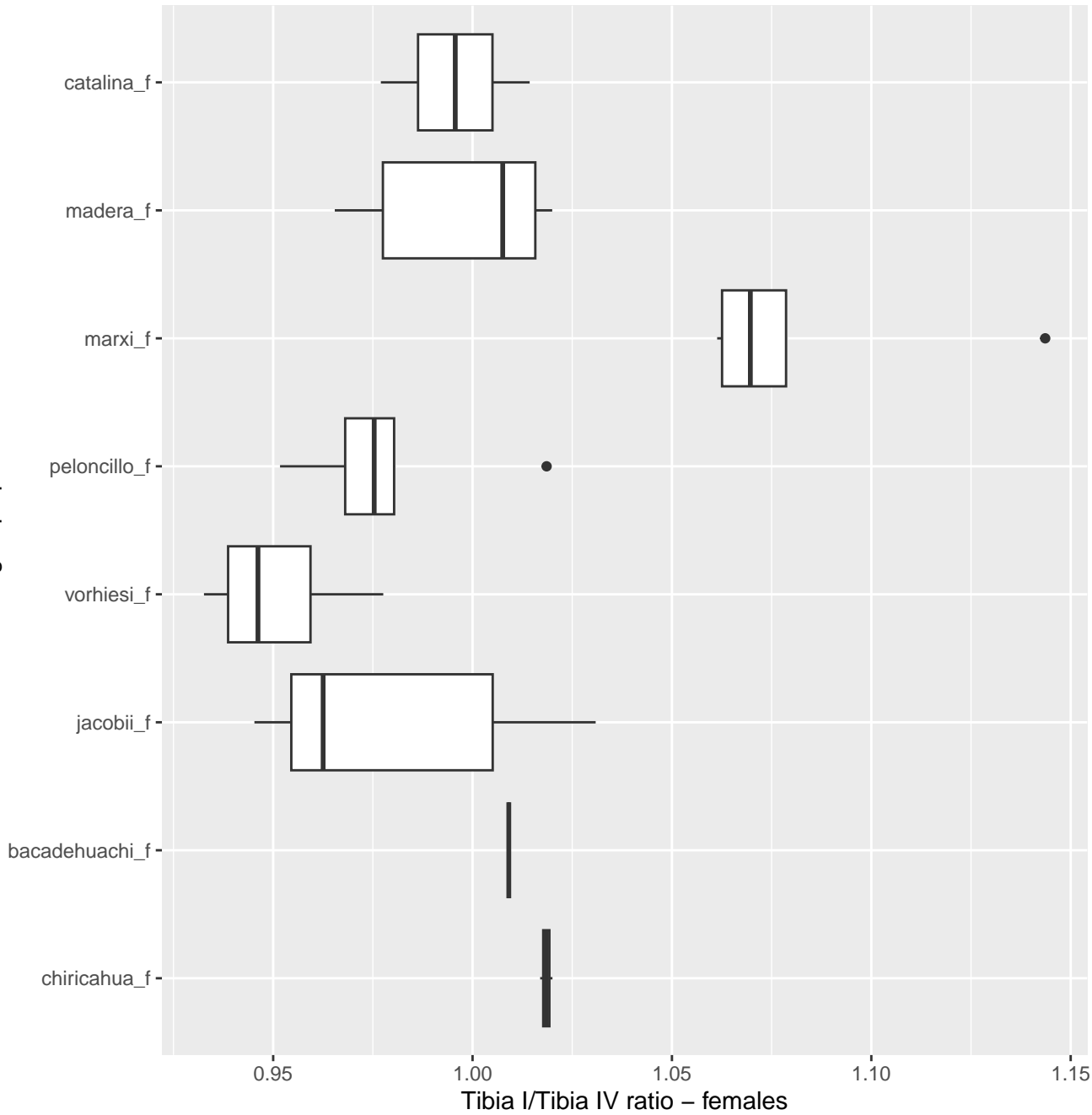

Supplement: Supplementary material 5 — Boxplots comparing various morphometric ratios based on measurements of mature male and female members of the Marxi species group [file zookeys-1210-061_article-125318__-s005.zip › Suppl5/Marxi_group_females/PDFs/T1T4_ratio_females.pdf]

Marxi group species

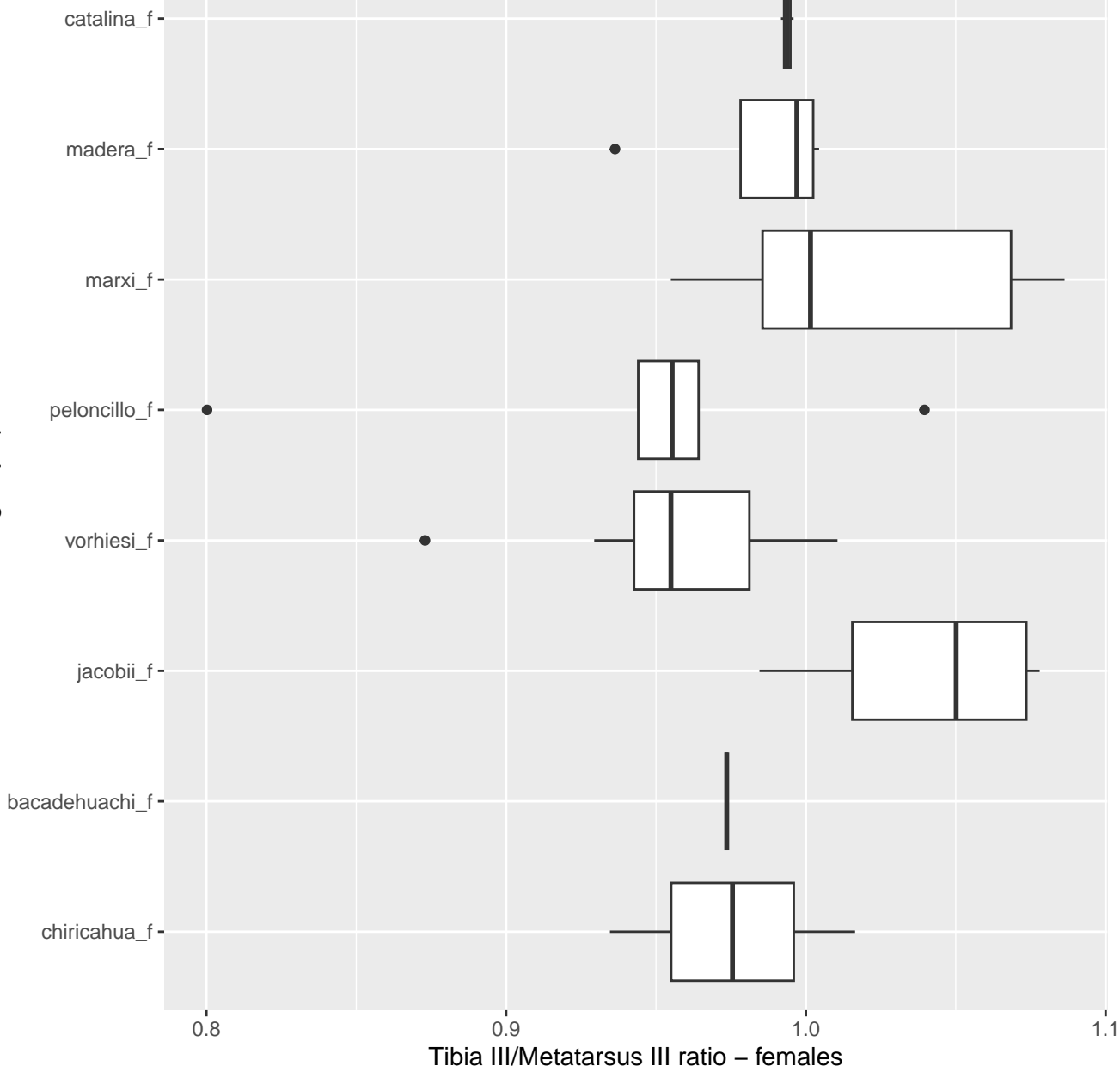

Supplement: Supplementary material 5 — Boxplots comparing various morphometric ratios based on measurements of mature male and female members of the Marxi species group [file zookeys-1210-061_article-125318__-s005.zip › Suppl5/Marxi_group_females/PDFs/T3M3_ratio_females.pdf]

Marxi group species

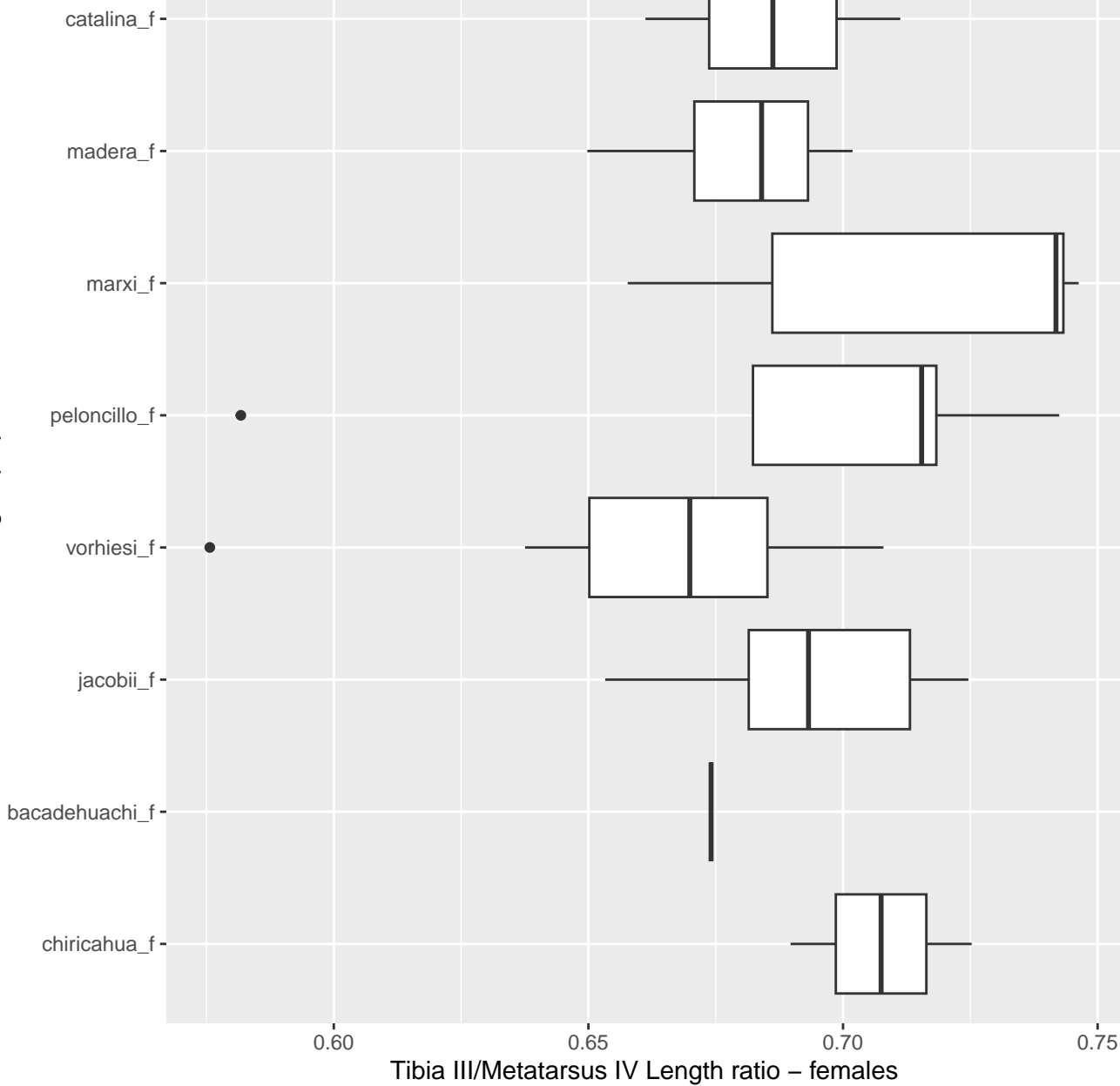

Supplement: Supplementary material 5 — Boxplots comparing various morphometric ratios based on measurements of mature male and female members of the Marxi species group [file zookeys-1210-061_article-125318__-s005.zip › Suppl5/Marxi_group_females/PDFs/T3M4_ratio_females.pdf]

Marxi species group

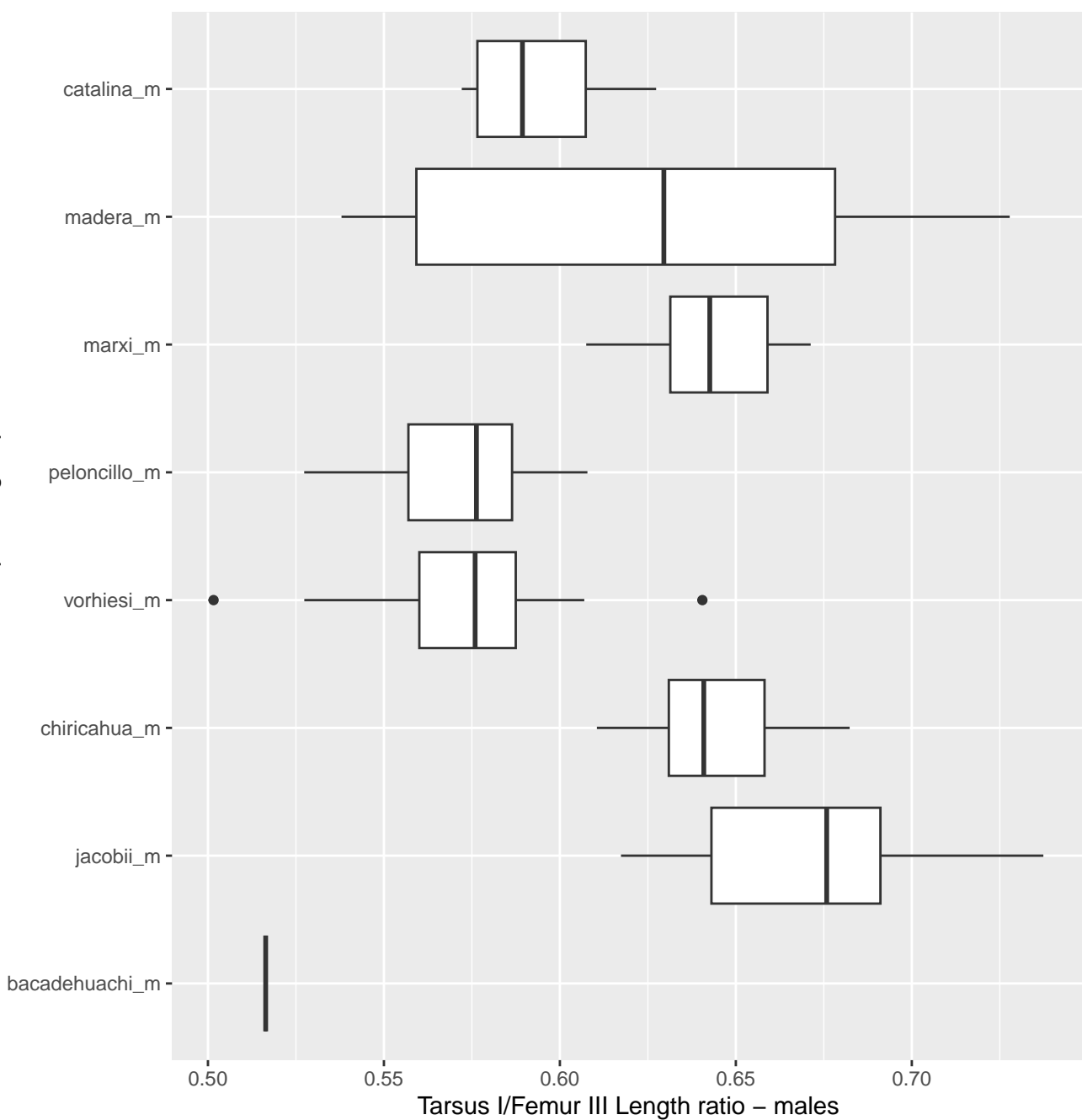

Supplement: Supplementary material 5 — Boxplots comparing various morphometric ratios based on measurements of mature male and female members of the Marxi species group [file zookeys-1210-061_article-125318__-s005.zip › Suppl5/Marxi_group_males/PDFs/A1F3_ratio_males.pdf]

Marxi species group

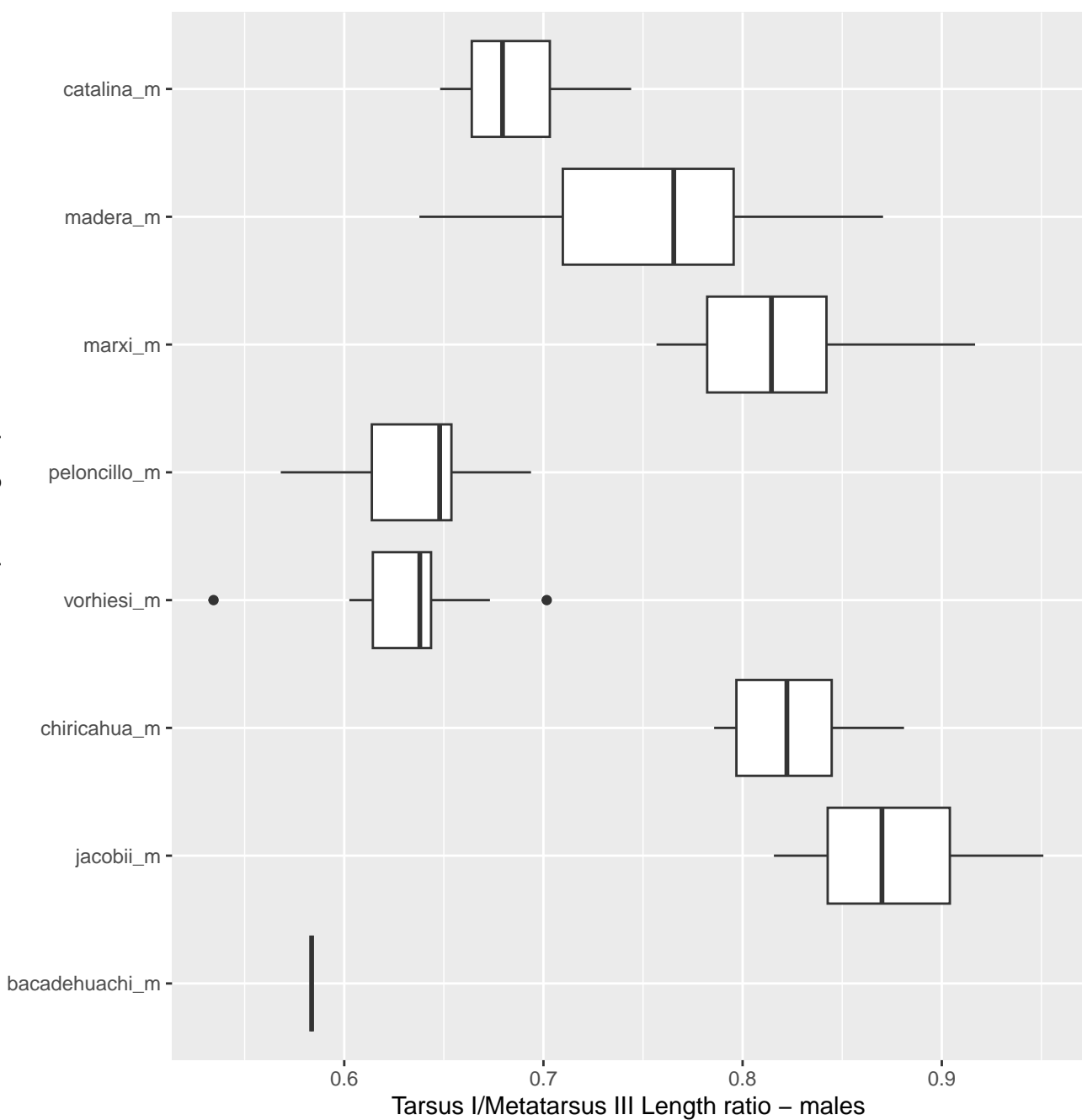

Supplement: Supplementary material 5 — Boxplots comparing various morphometric ratios based on measurements of mature male and female members of the Marxi species group [file zookeys-1210-061_article-125318__-s005.zip › Suppl5/Marxi_group_males/PDFs/A1M3_ratio_males.pdf]

Marxi species group

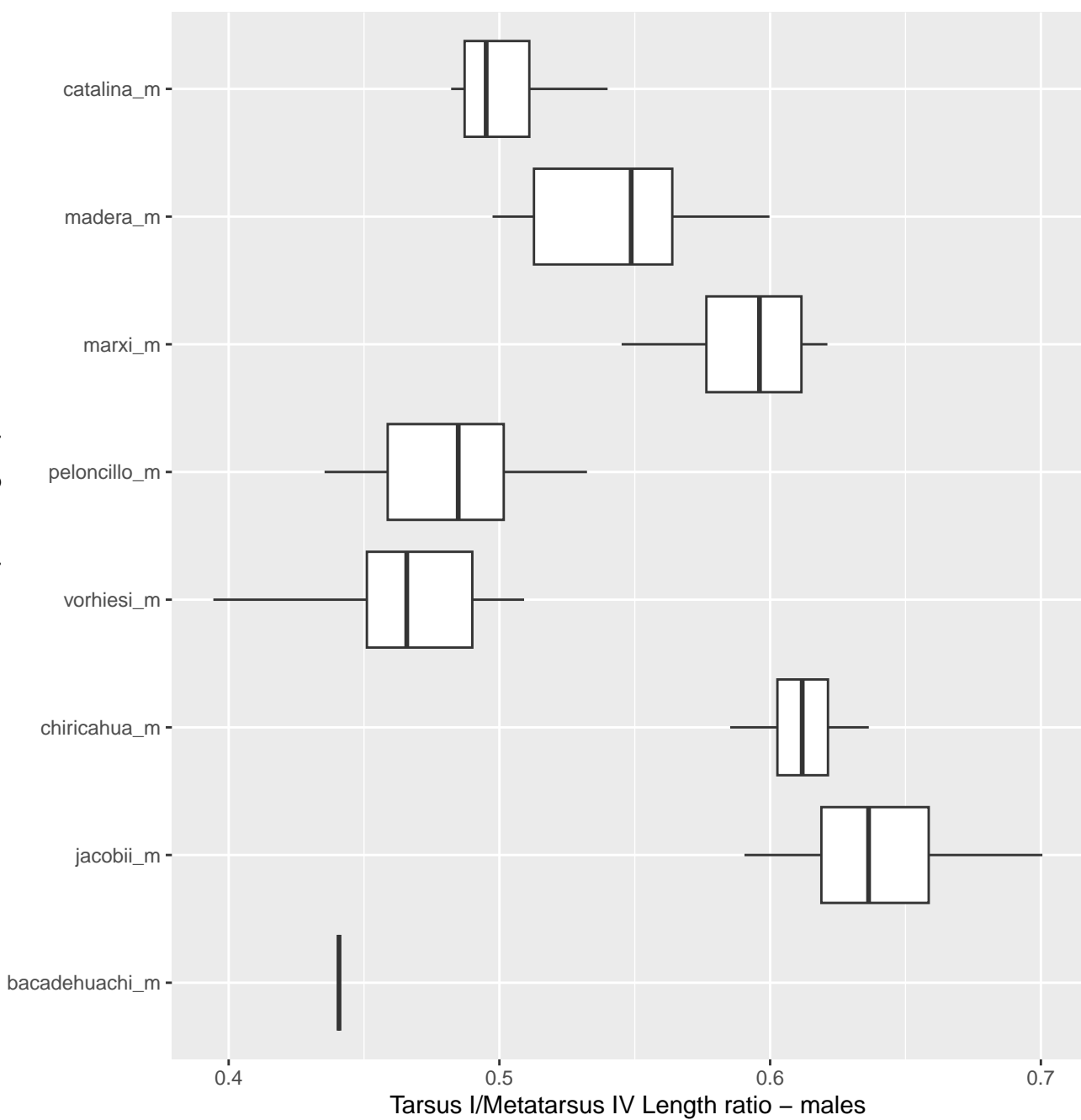

Supplement: Supplementary material 5 — Boxplots comparing various morphometric ratios based on measurements of mature male and female members of the Marxi species group [file zookeys-1210-061_article-125318__-s005.zip › Suppl5/Marxi_group_males/PDFs/A1M4_ratio_males.pdf]

Marxi species group

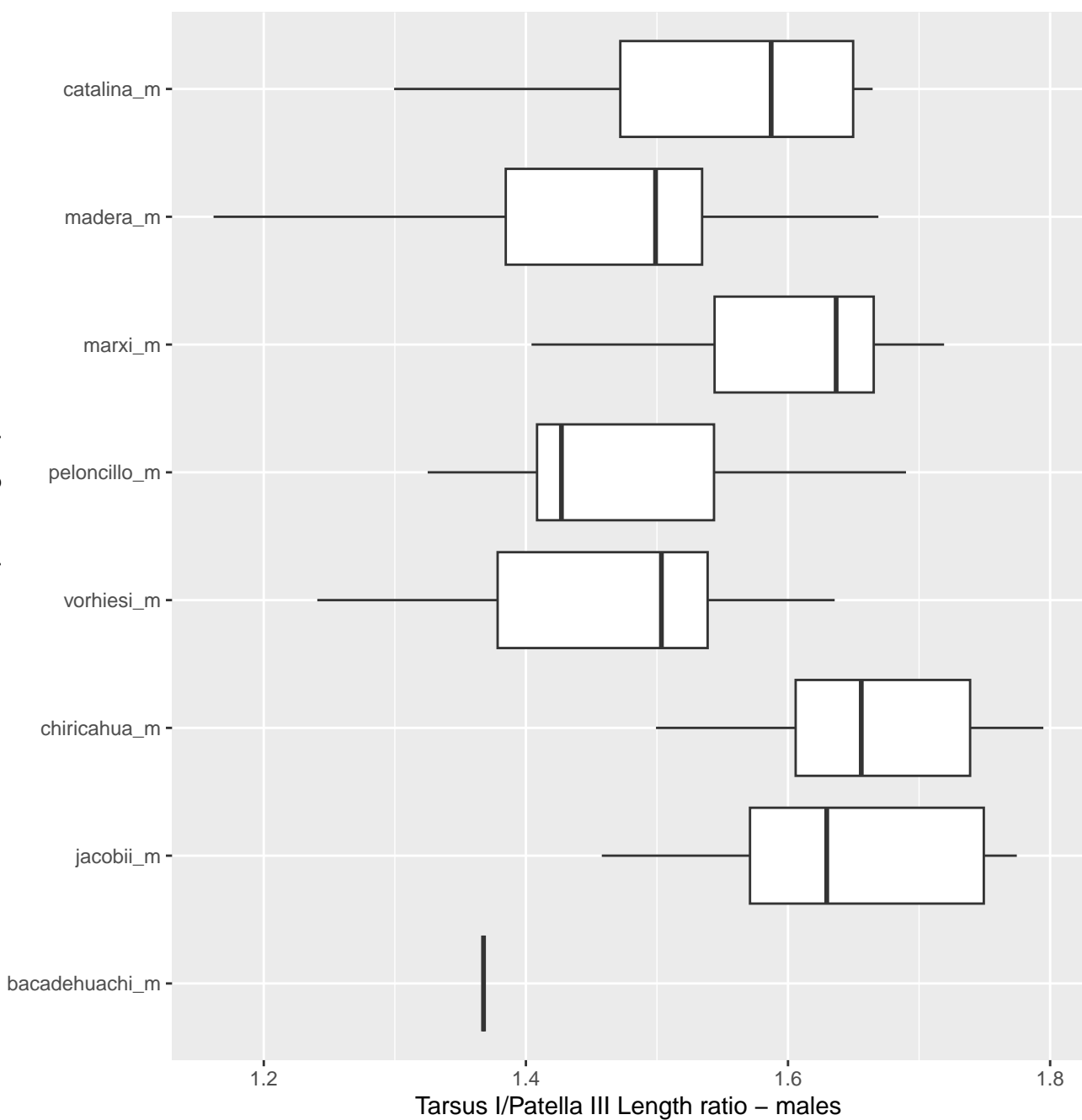

Supplement: Supplementary material 5 — Boxplots comparing various morphometric ratios based on measurements of mature male and female members of the Marxi species group [file zookeys-1210-061_article-125318__-s005.zip › Suppl5/Marxi_group_males/PDFs/A1P3_ratio_males.pdf]

Marxi species group

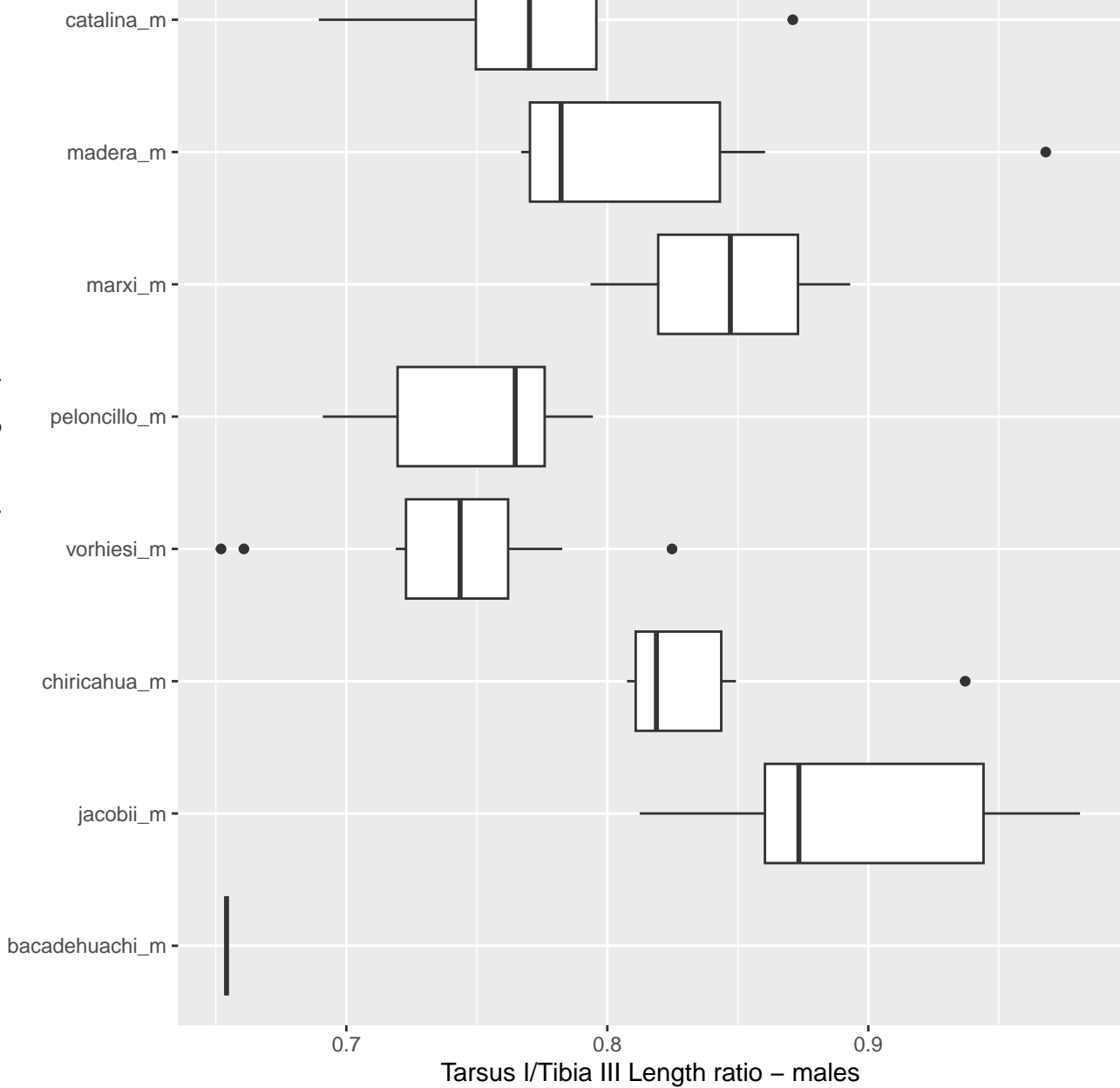

Supplement: Supplementary material 5 — Boxplots comparing various morphometric ratios based on measurements of mature male and female members of the Marxi species group [file zookeys-1210-061_article-125318__-s005.zip › Suppl5/Marxi_group_males/PDFs/A1T3_ratio_males.pdf]

Marxi species group

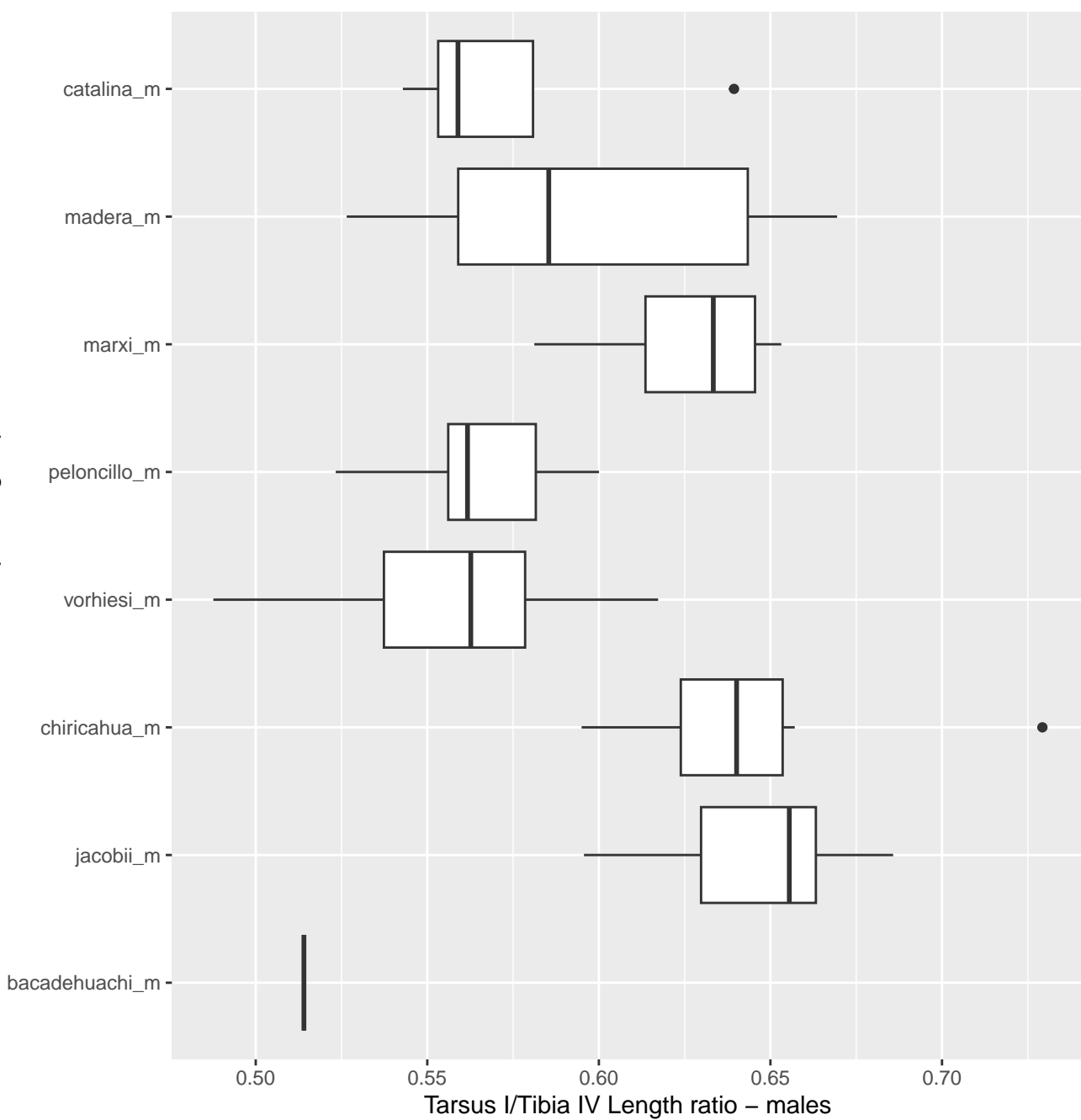

Supplement: Supplementary material 5 — Boxplots comparing various morphometric ratios based on measurements of mature male and female members of the Marxi species group [file zookeys-1210-061_article-125318__-s005.zip › Suppl5/Marxi_group_males/PDFs/A1T4_ratio_males.pdf]

Marxi species group

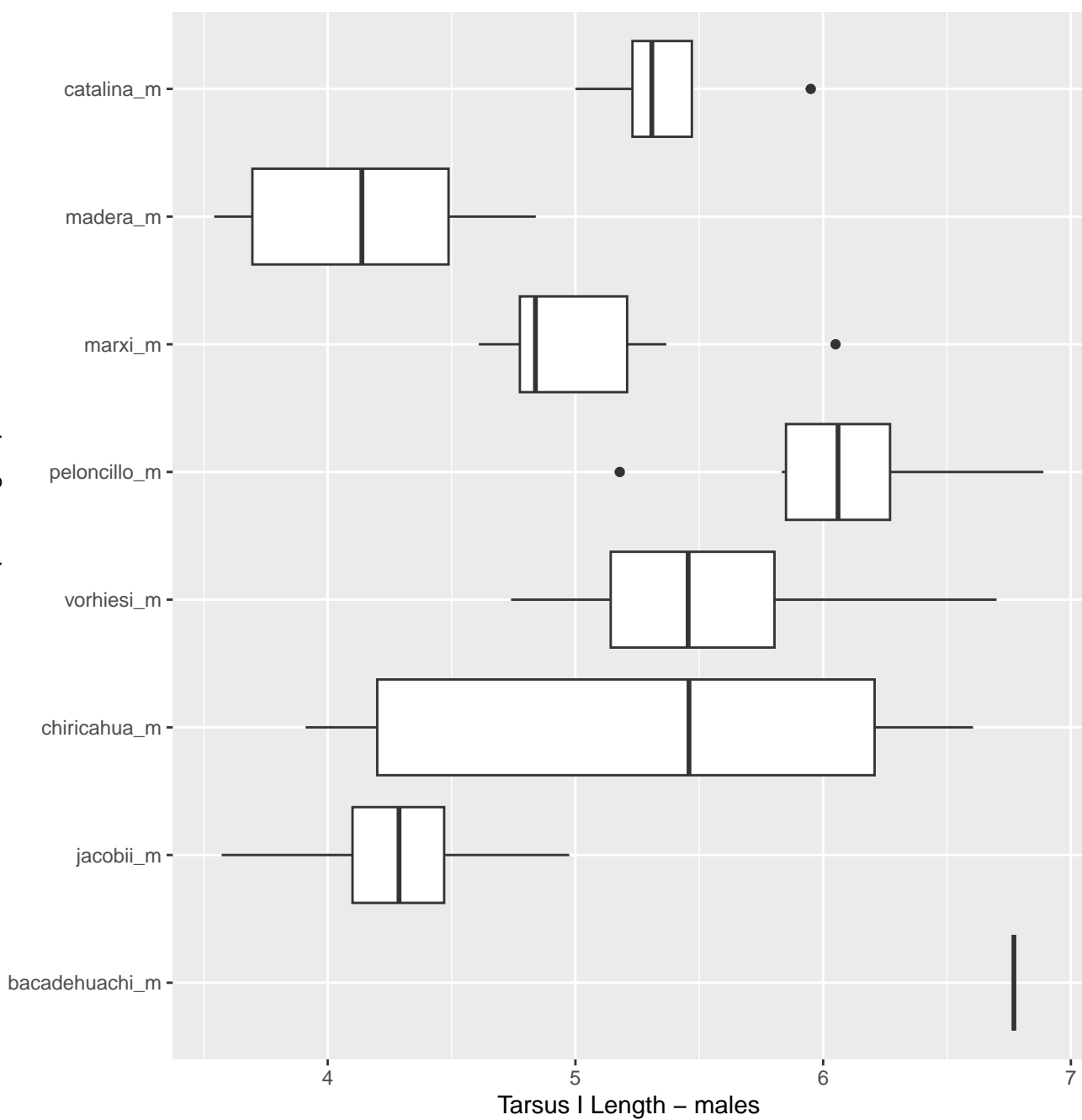

Supplement: Supplementary material 5 — Boxplots comparing various morphometric ratios based on measurements of mature male and female members of the Marxi species group [file zookeys-1210-061_article-125318__-s005.zip › Suppl5/Marxi_group_males/PDFs/A1_males.pdf]

Marxi species group

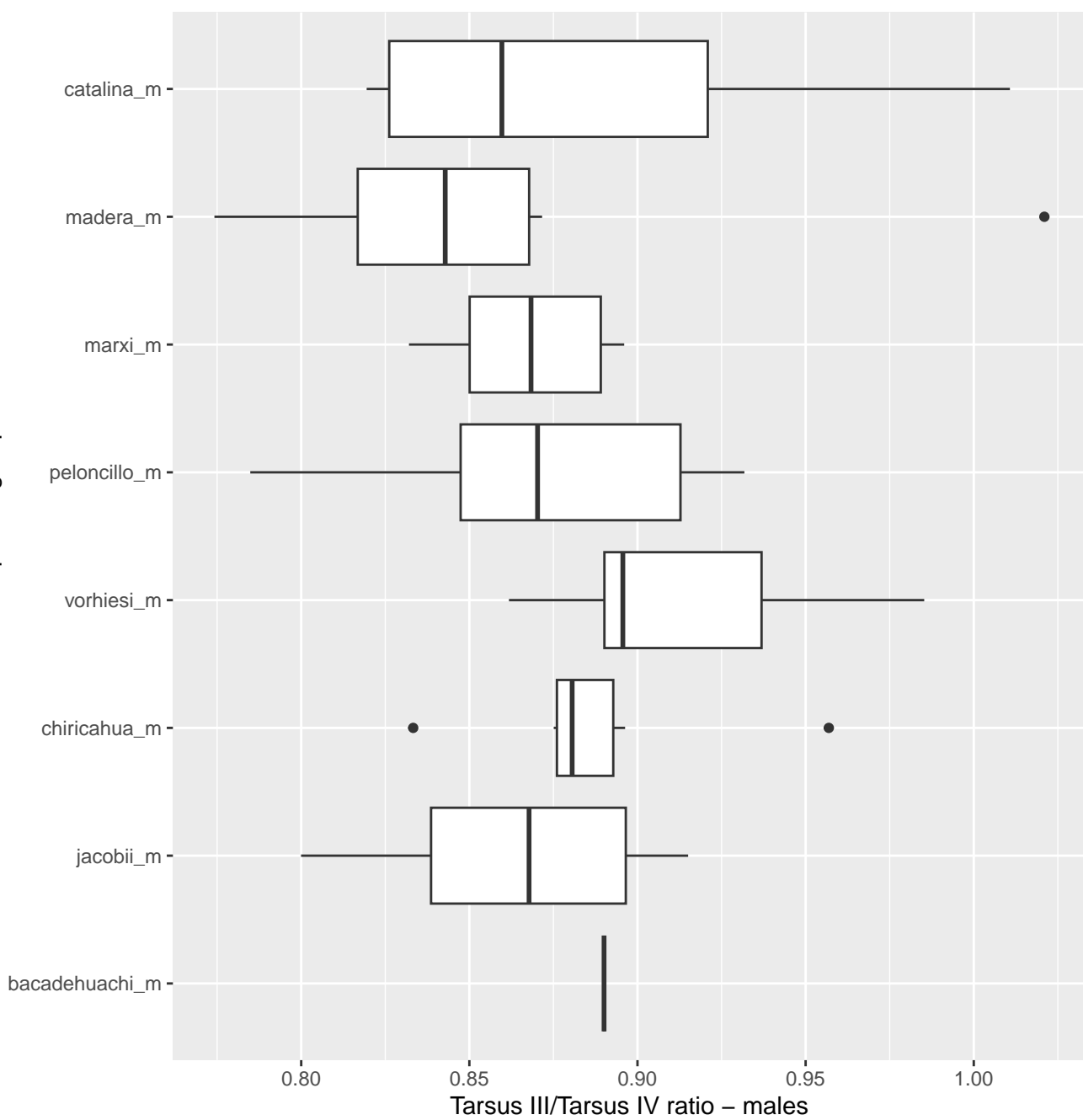

Supplement: Supplementary material 5 — Boxplots comparing various morphometric ratios based on measurements of mature male and female members of the Marxi species group [file zookeys-1210-061_article-125318__-s005.zip › Suppl5/Marxi_group_males/PDFs/A3A4_ratio_males.pdf]

Marxi species group

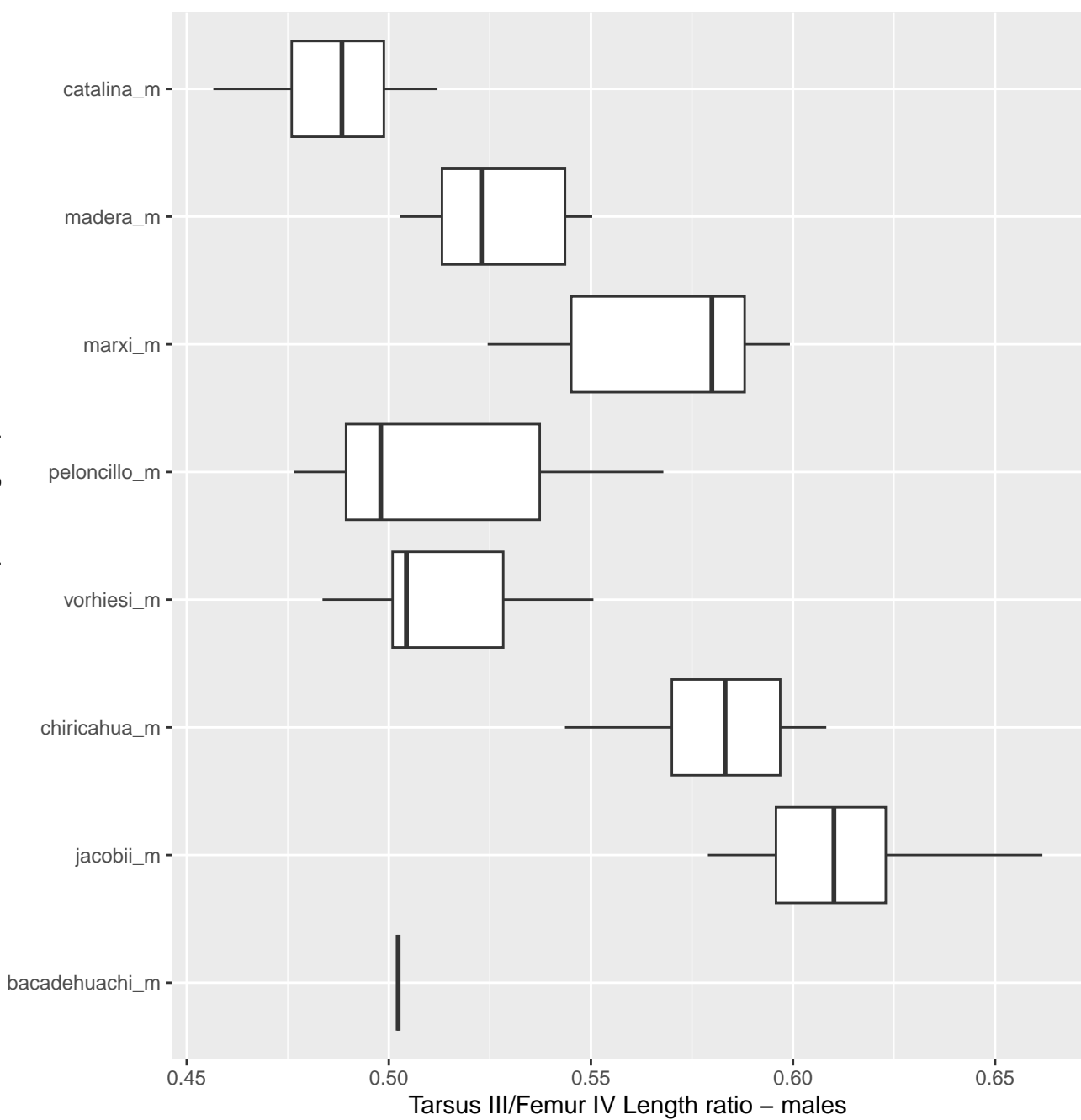

Supplement: Supplementary material 5 — Boxplots comparing various morphometric ratios based on measurements of mature male and female members of the Marxi species group [file zookeys-1210-061_article-125318__-s005.zip › Suppl5/Marxi_group_males/PDFs/A3F4_ratio_males.pdf]

Marxi species group

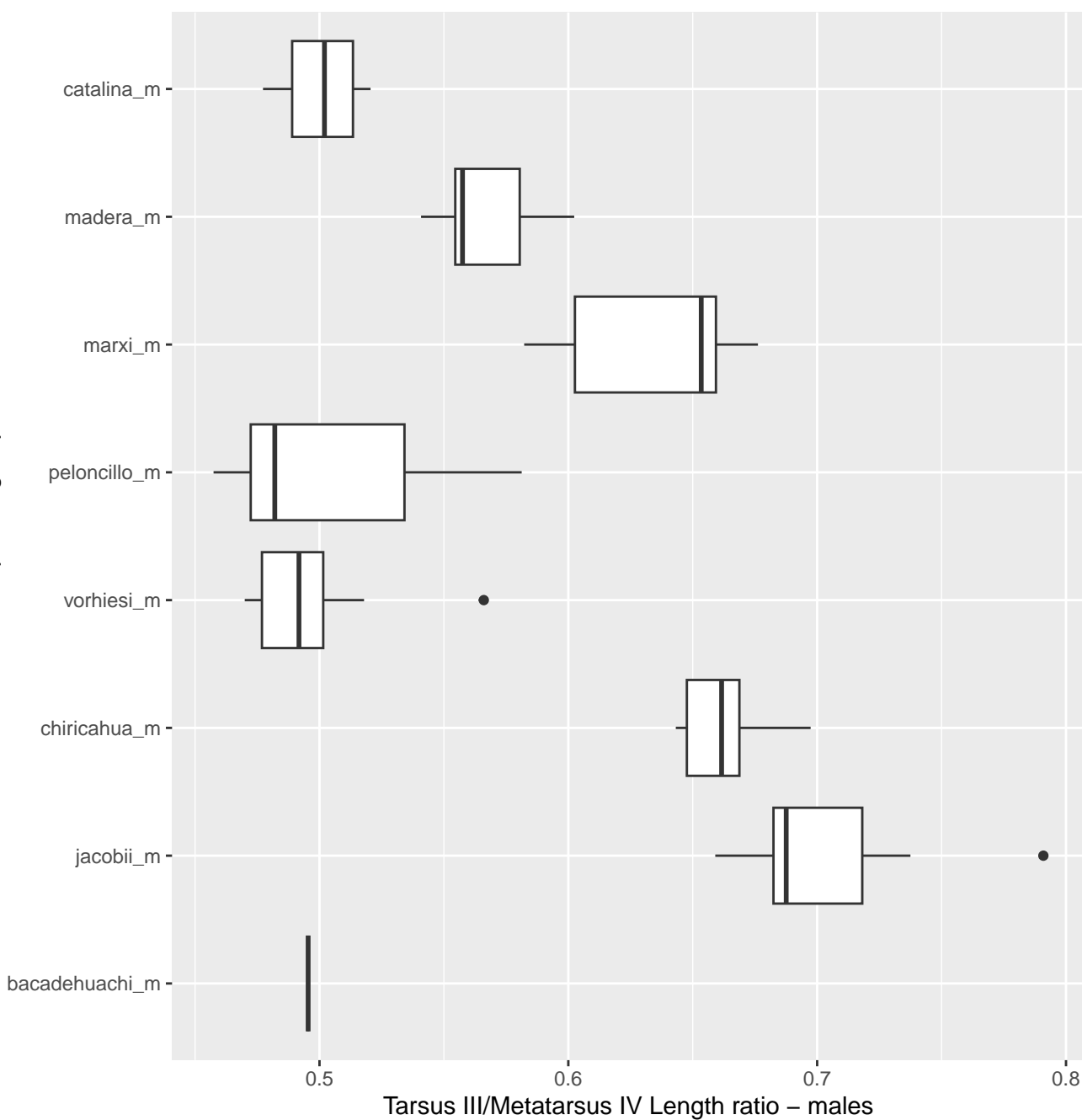

Supplement: Supplementary material 5 — Boxplots comparing various morphometric ratios based on measurements of mature male and female members of the Marxi species group [file zookeys-1210-061_article-125318__-s005.zip › Suppl5/Marxi_group_males/PDFs/A3M4_ratio_males.pdf]

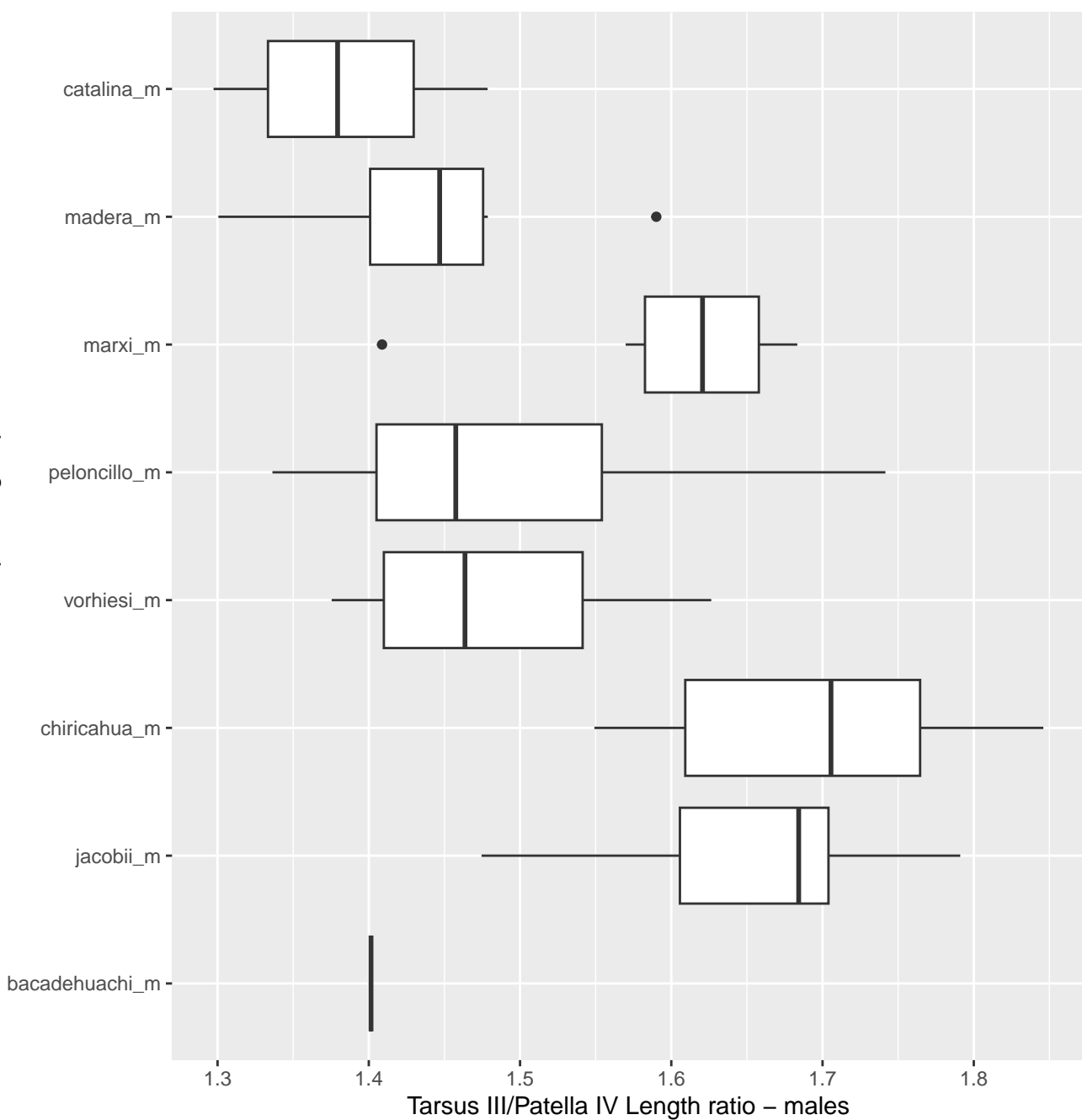

Supplement: Supplementary material 5 — Boxplots comparing various morphometric ratios based on measurements of mature male and female members of the Marxi species group [file zookeys-1210-061_article-125318__-s005.zip › Suppl5/Marxi_group_males/PDFs/A3P4_ratio_males.pdf]

Marxi species group

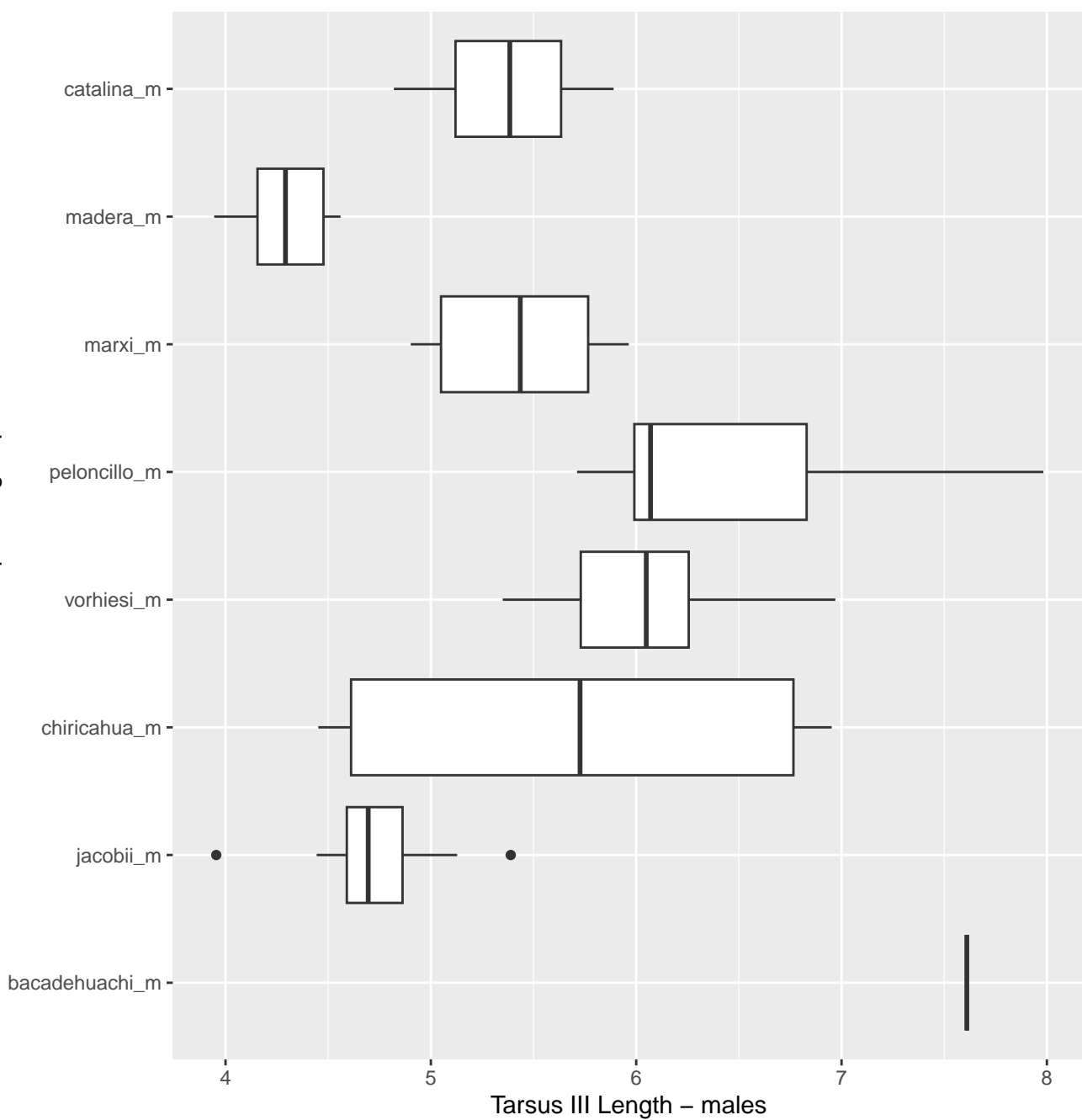

Supplement: Supplementary material 5 — Boxplots comparing various morphometric ratios based on measurements of mature male and female members of the Marxi species group [file zookeys-1210-061_article-125318__-s005.zip › Suppl5/Marxi_group_males/PDFs/A3_males.pdf]

Marxi species group

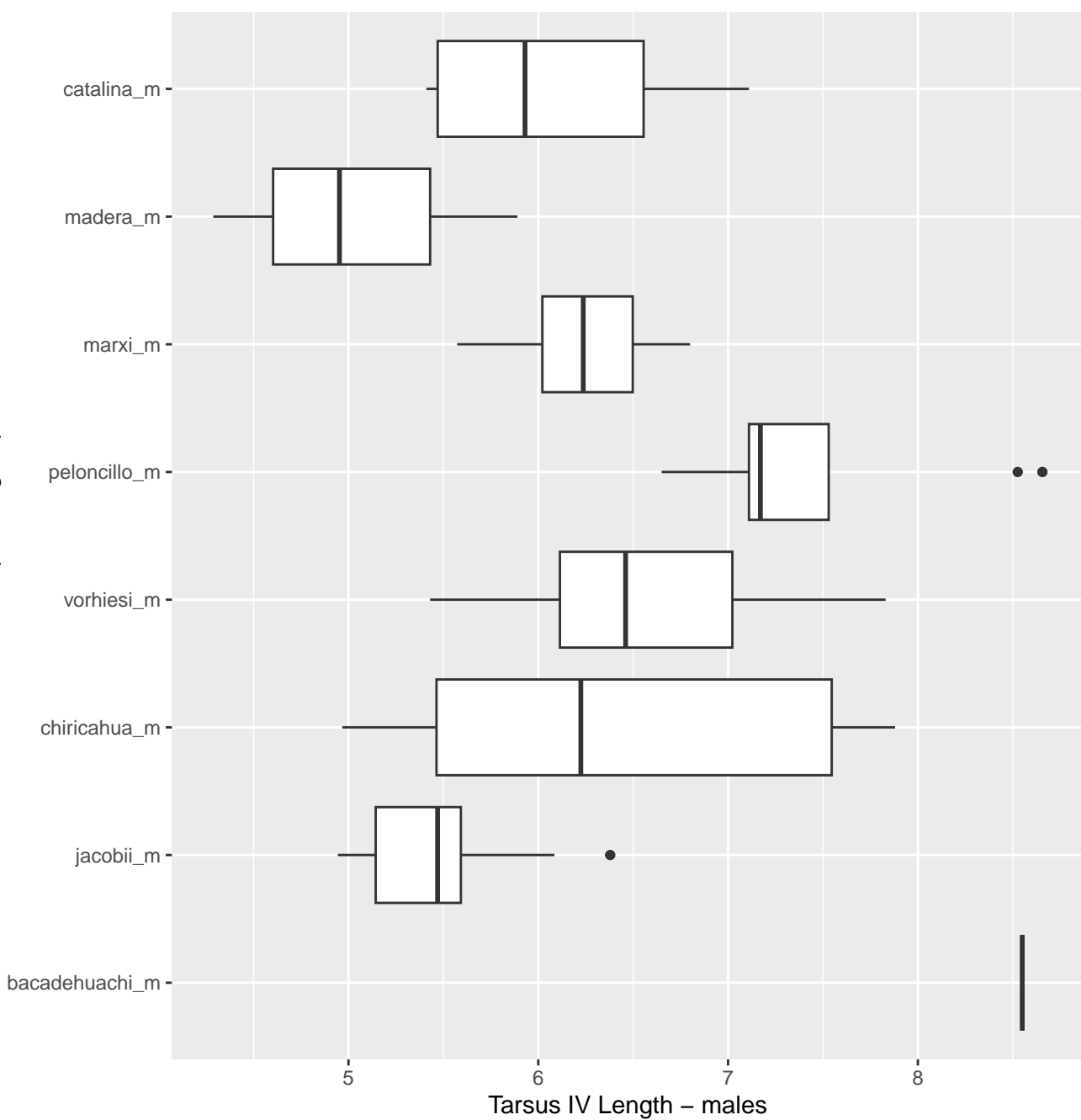

Supplement: Supplementary material 5 — Boxplots comparing various morphometric ratios based on measurements of mature male and female members of the Marxi species group [file zookeys-1210-061_article-125318__-s005.zip › Suppl5/Marxi_group_males/PDFs/A4_males.pdf]

Marxi species group

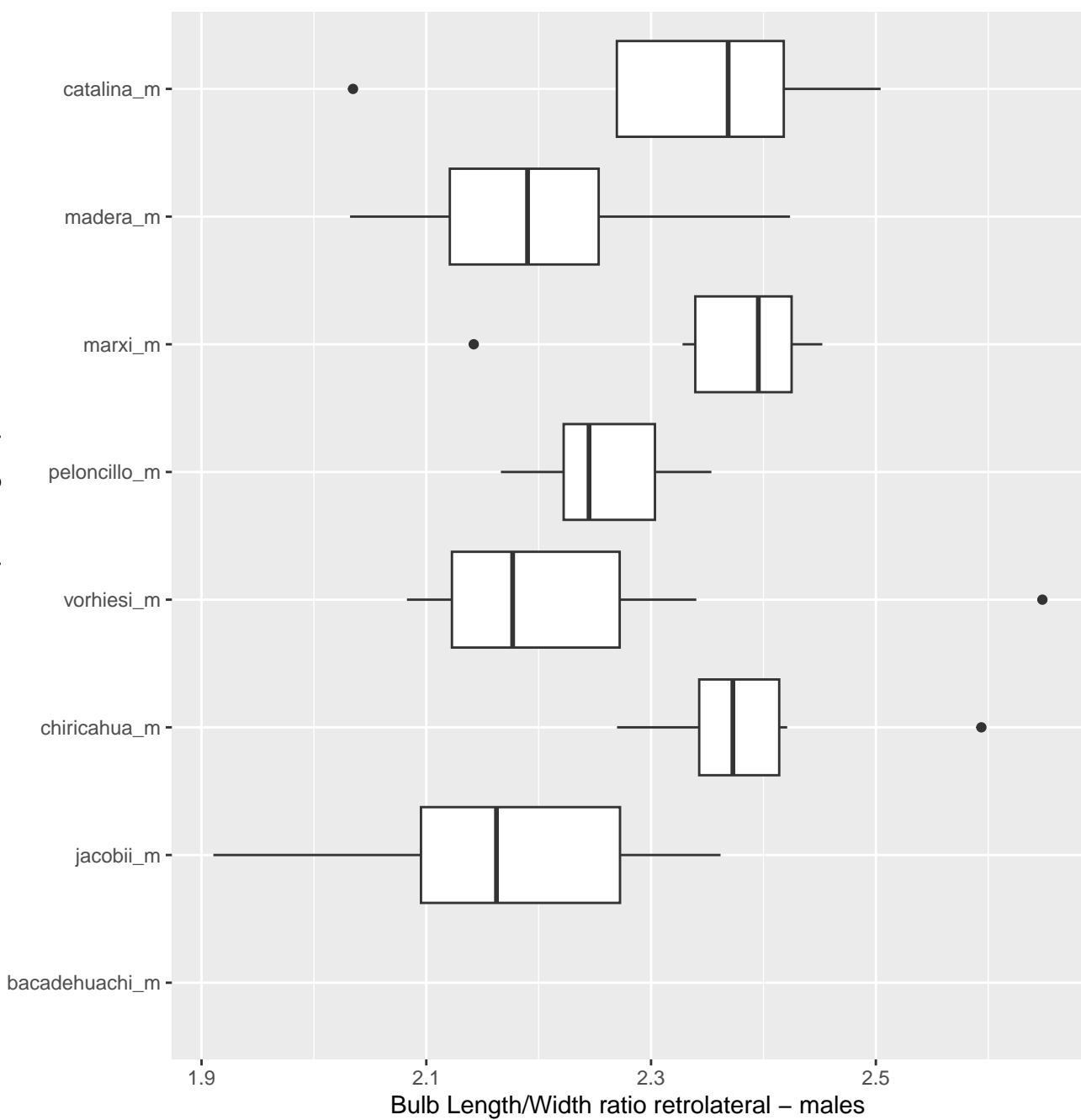

Supplement: Supplementary material 5 — Boxplots comparing various morphometric ratios based on measurements of mature male and female members of the Marxi species group [file zookeys-1210-061_article-125318__-s005.zip › Suppl5/Marxi_group_males/PDFs/Blw_r_ratio_males.pdf]

Marxi species group

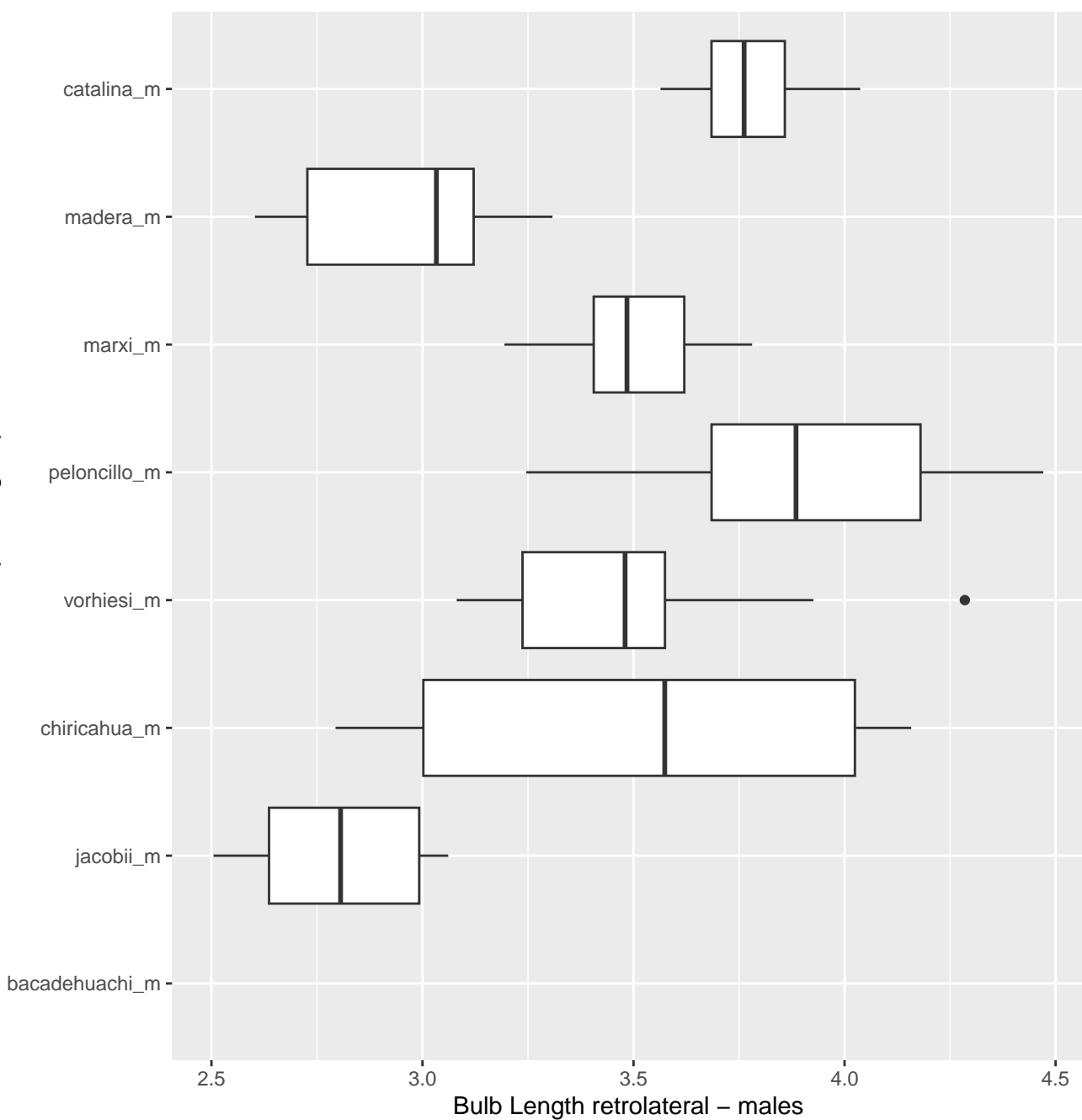

Supplement: Supplementary material 5 — Boxplots comparing various morphometric ratios based on measurements of mature male and female members of the Marxi species group [file zookeys-1210-061_article-125318__-s005.zip › Suppl5/Marxi_group_males/PDFs/Bl_r_males.pdf]

Marxi species group

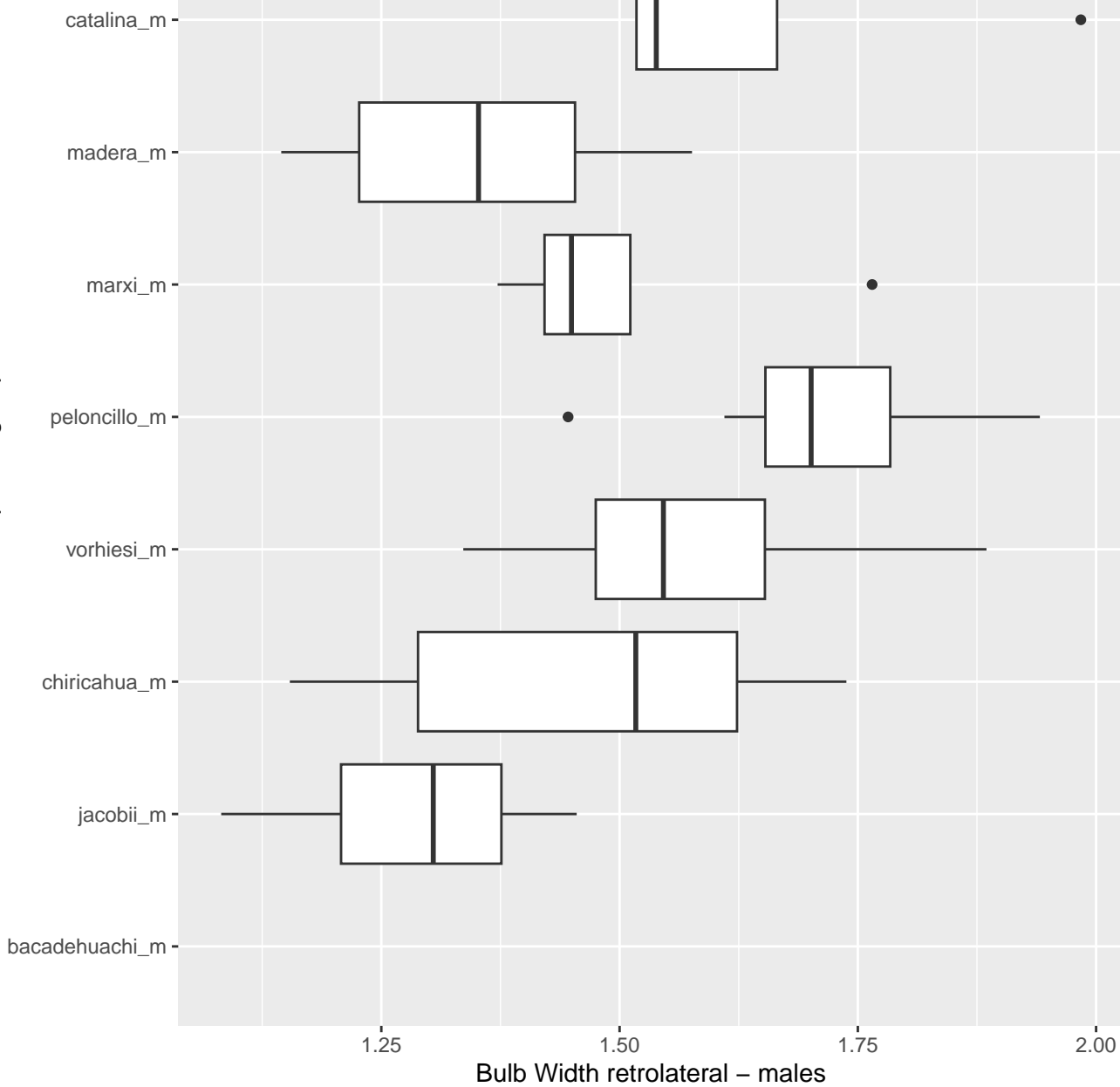

Supplement: Supplementary material 5 — Boxplots comparing various morphometric ratios based on measurements of mature male and female members of the Marxi species group [file zookeys-1210-061_article-125318__-s005.zip › Suppl5/Marxi_group_males/PDFs/Bw_r_males.pdf]

Marxi species group

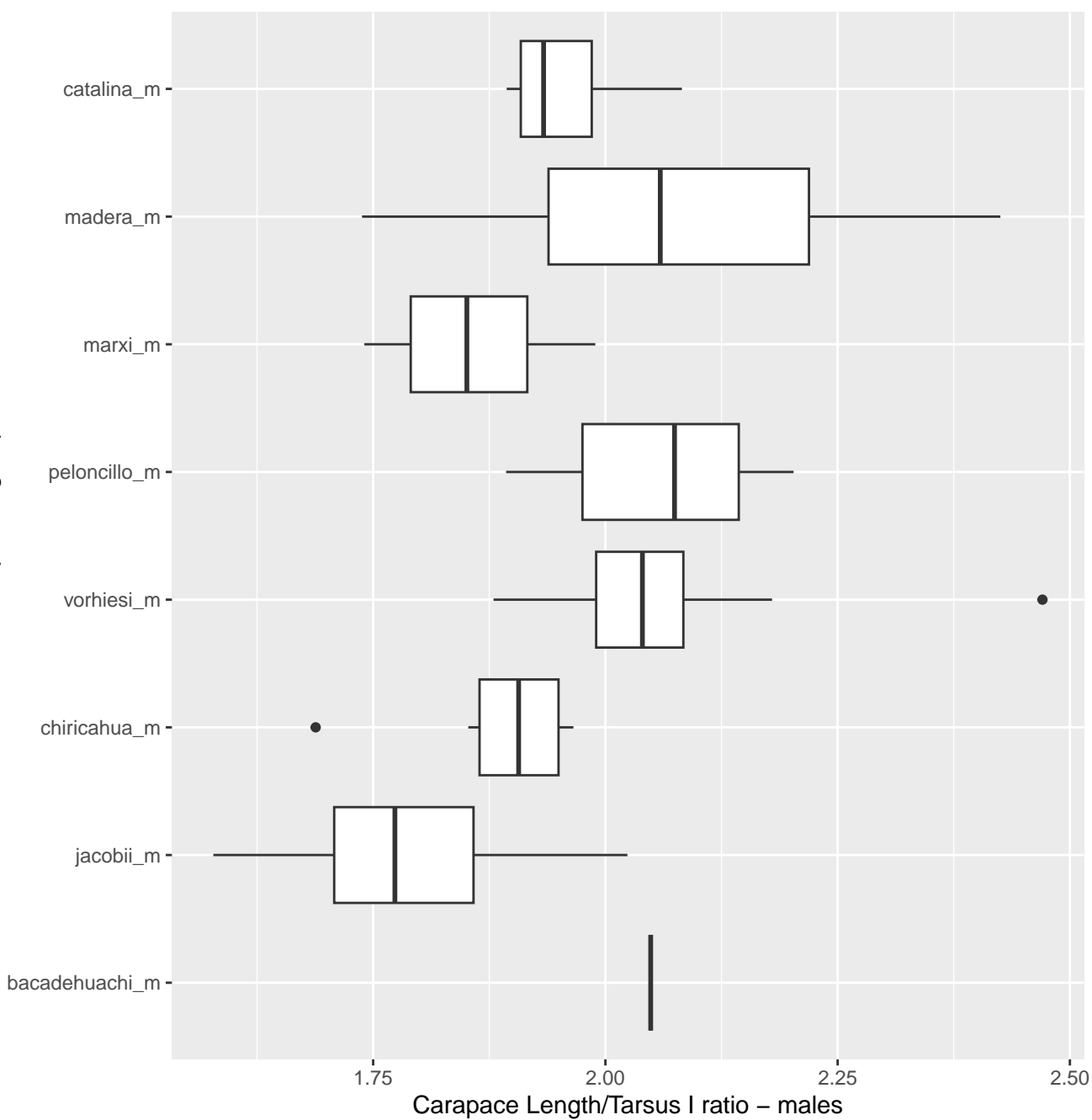

Supplement: Supplementary material 5 — Boxplots comparing various morphometric ratios based on measurements of mature male and female members of the Marxi species group [file zookeys-1210-061_article-125318__-s005.zip › Suppl5/Marxi_group_males/PDFs/ClA1_ratio_males.pdf]

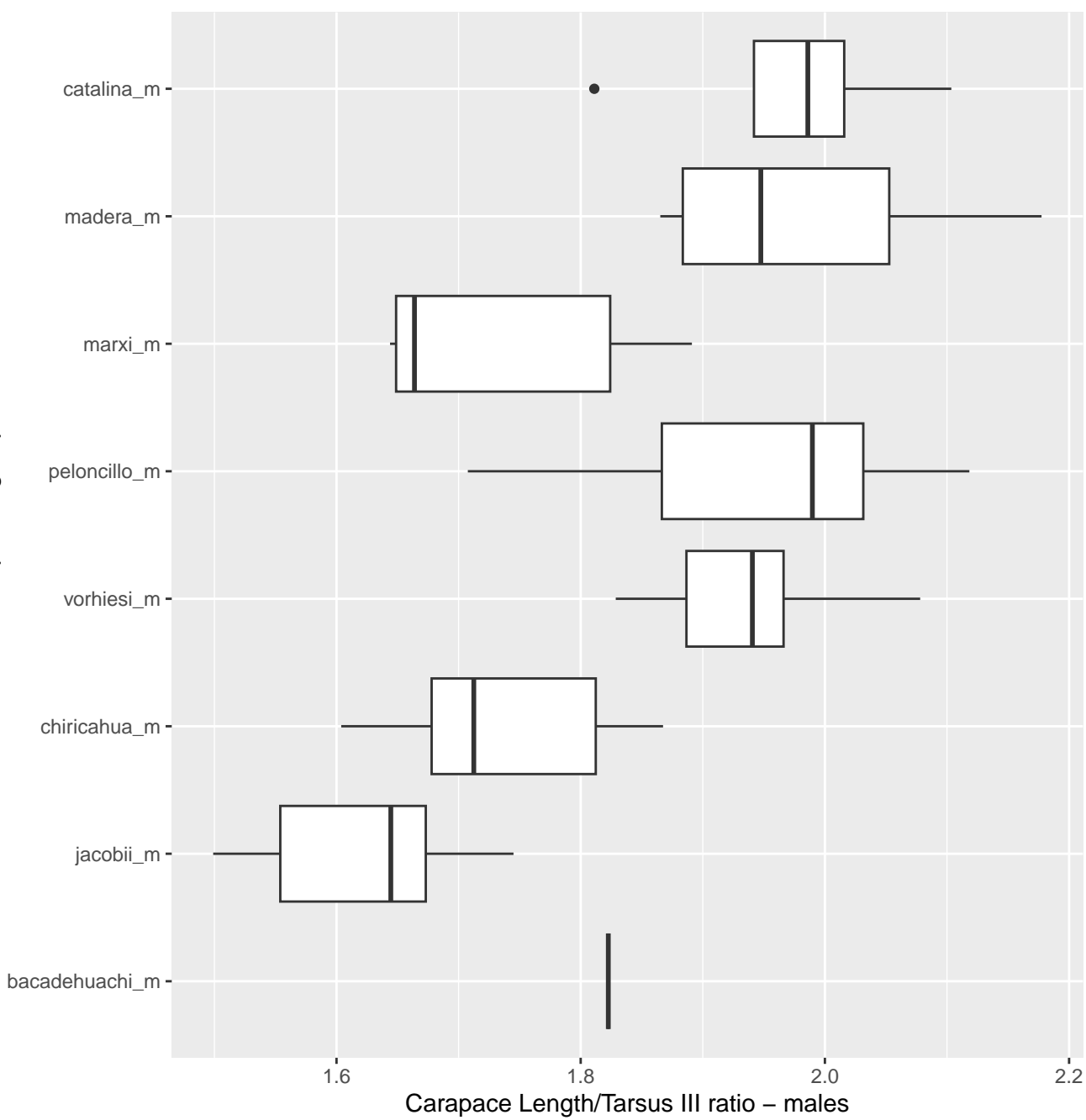

Supplement: Supplementary material 5 — Boxplots comparing various morphometric ratios based on measurements of mature male and female members of the Marxi species group [file zookeys-1210-061_article-125318__-s005.zip › Suppl5/Marxi_group_males/PDFs/ClA3_ratio_males.pdf]

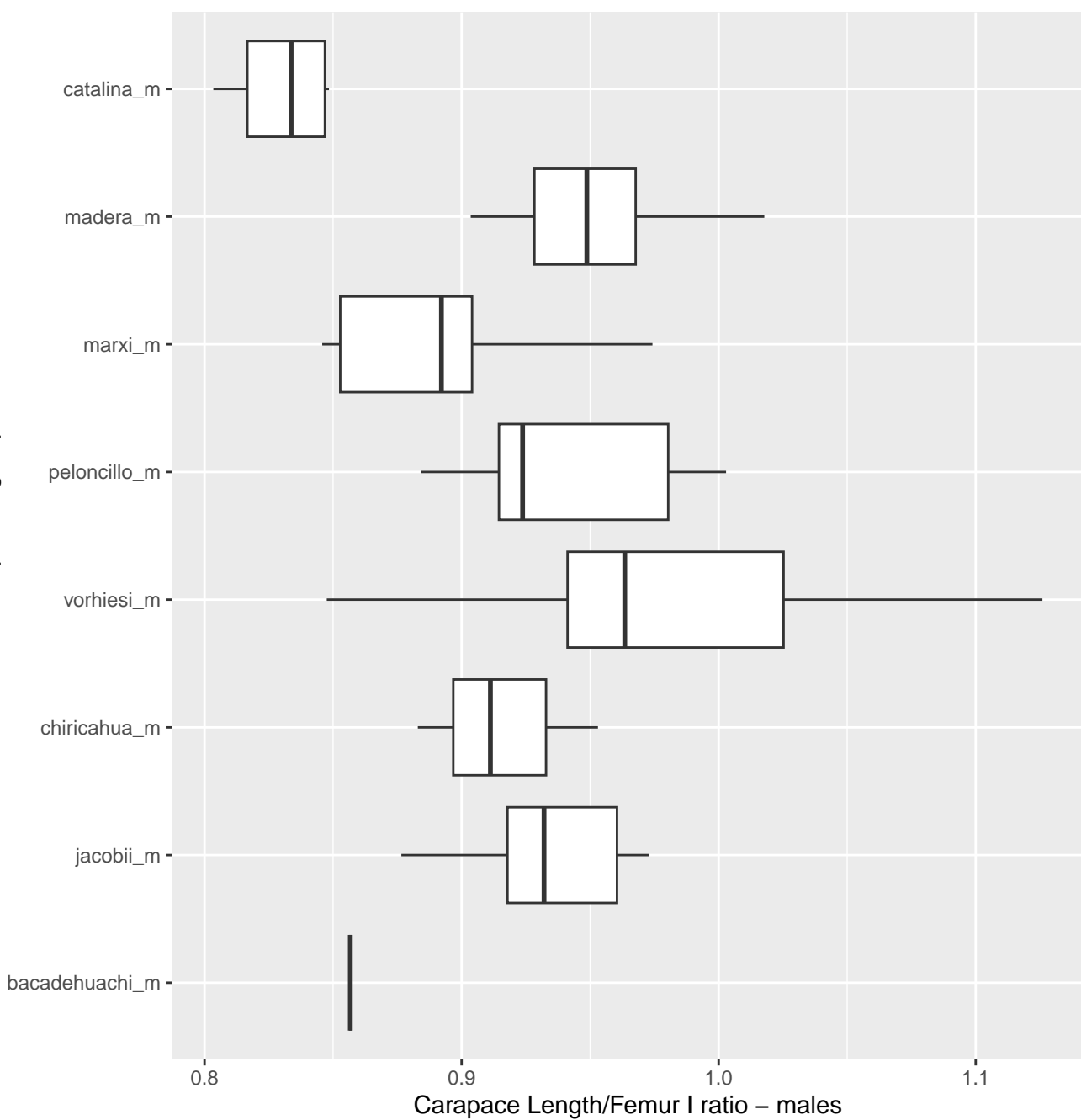

Supplement: Supplementary material 5 — Boxplots comparing various morphometric ratios based on measurements of mature male and female members of the Marxi species group [file zookeys-1210-061_article-125318__-s005.zip › Suppl5/Marxi_group_males/PDFs/ClF1_ratio_males.pdf]

Marxi species group

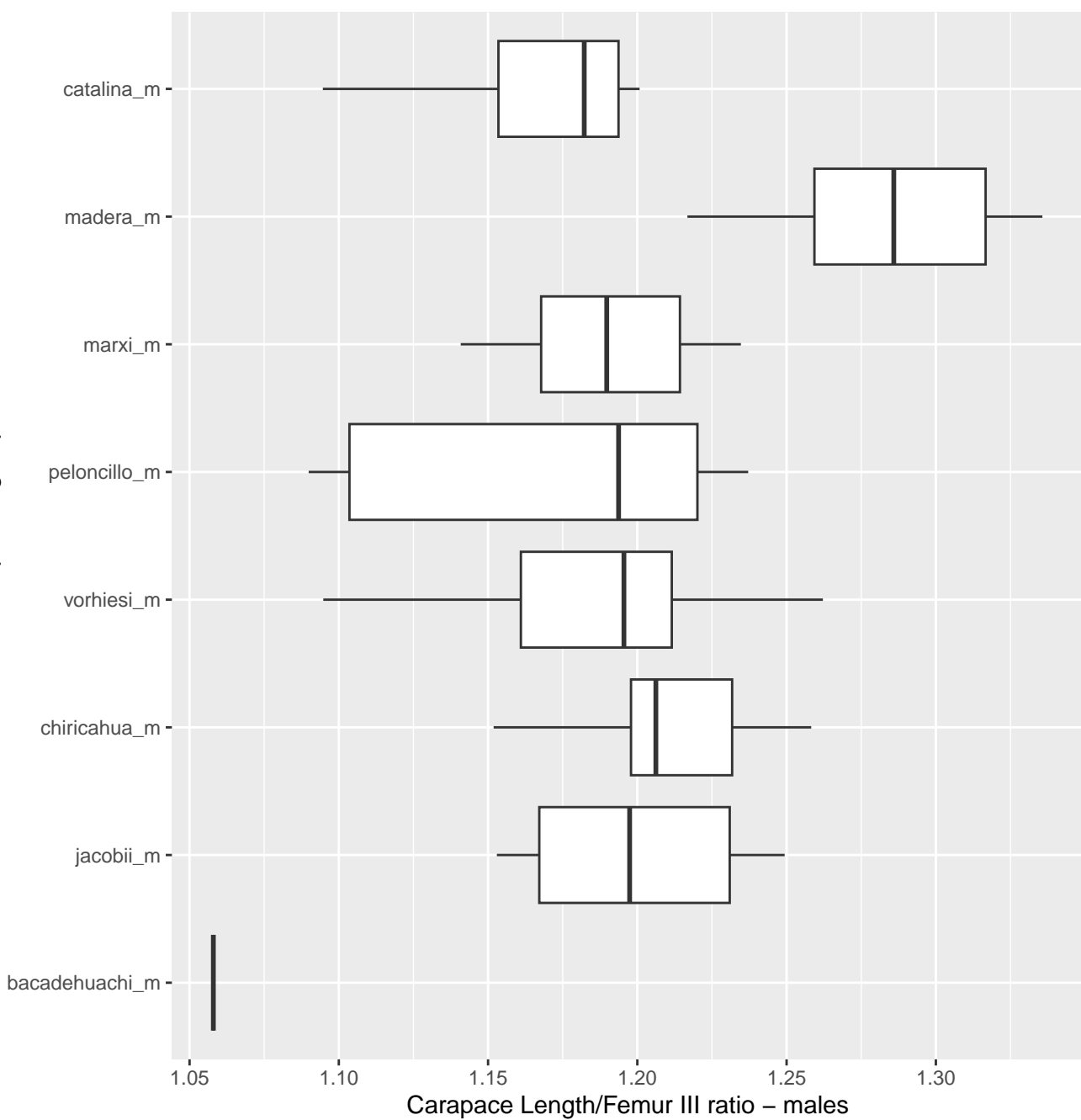

Supplement: Supplementary material 5 — Boxplots comparing various morphometric ratios based on measurements of mature male and female members of the Marxi species group [file zookeys-1210-061_article-125318__-s005.zip › Suppl5/Marxi_group_males/PDFs/ClF3_ratio_males.pdf]

Marxi species group

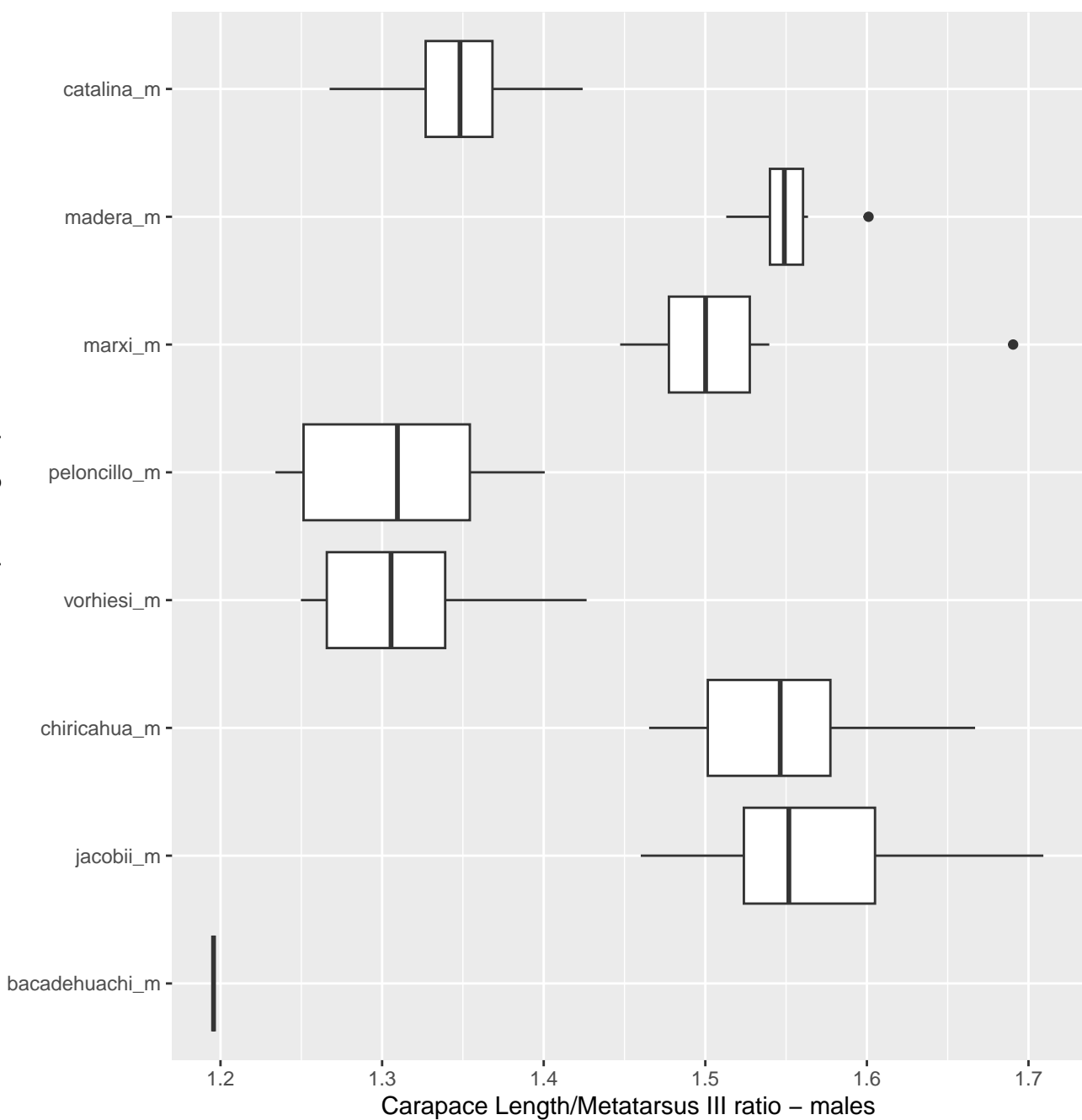

Supplement: Supplementary material 5 — Boxplots comparing various morphometric ratios based on measurements of mature male and female members of the Marxi species group [file zookeys-1210-061_article-125318__-s005.zip › Suppl5/Marxi_group_males/PDFs/ClM3_ratio_males.pdf]

Marxi species group

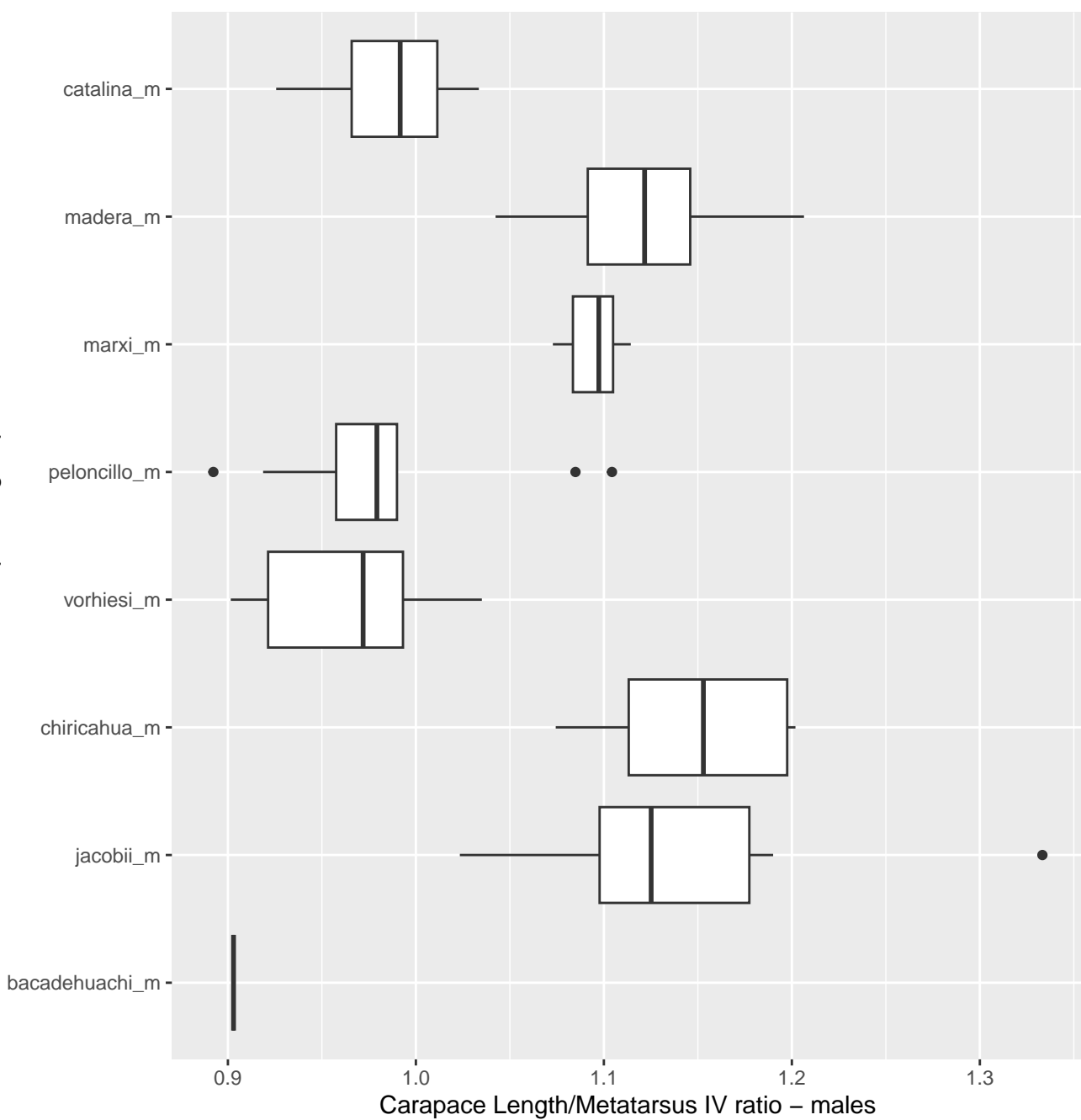

Supplement: Supplementary material 5 — Boxplots comparing various morphometric ratios based on measurements of mature male and female members of the Marxi species group [file zookeys-1210-061_article-125318__-s005.zip › Suppl5/Marxi_group_males/PDFs/ClM4_ratio_males.pdf]

Marxi species group

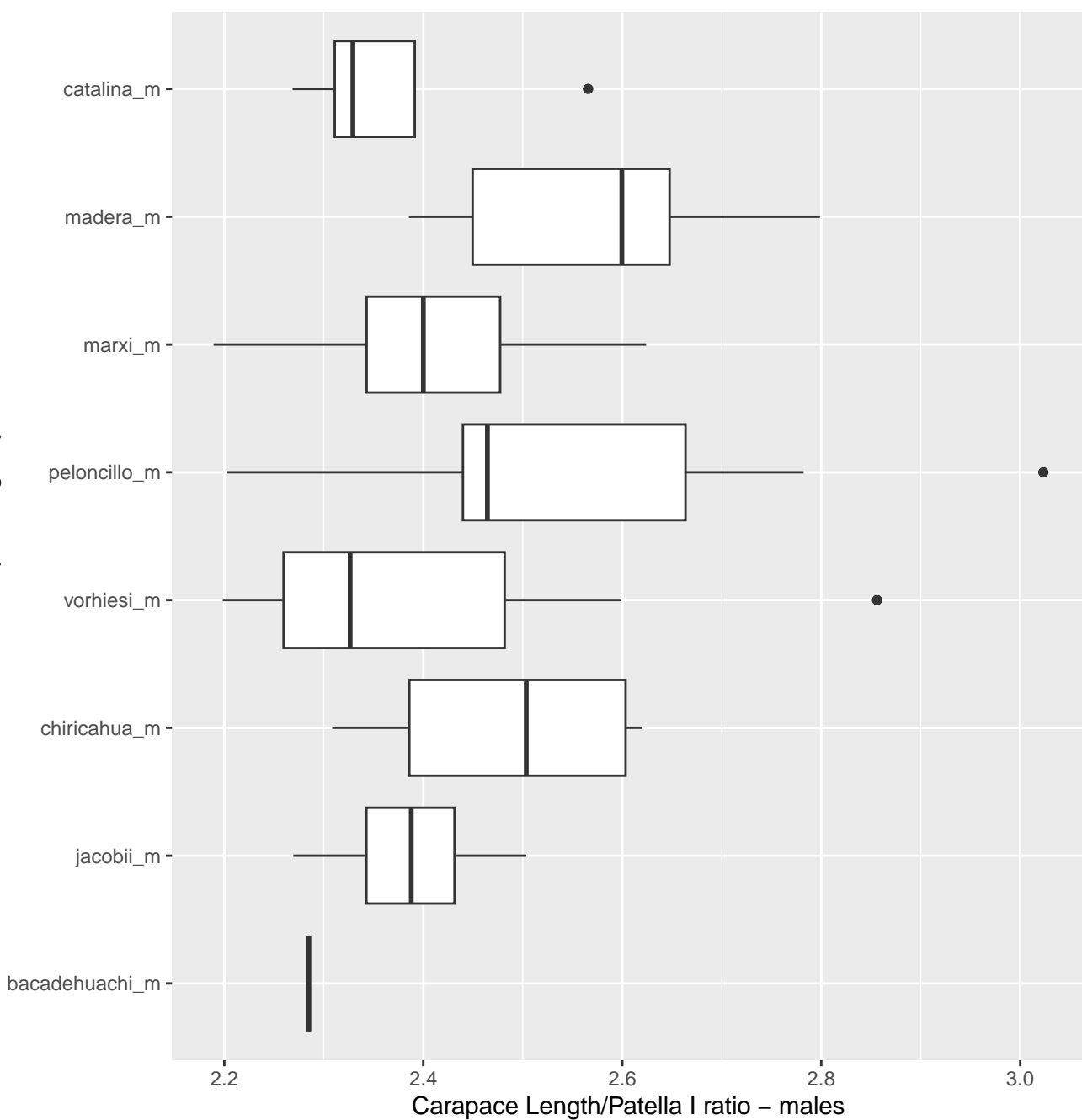

Supplement: Supplementary material 5 — Boxplots comparing various morphometric ratios based on measurements of mature male and female members of the Marxi species group [file zookeys-1210-061_article-125318__-s005.zip › Suppl5/Marxi_group_males/PDFs/ClP1_ratio_males.pdf]

Marxi species group

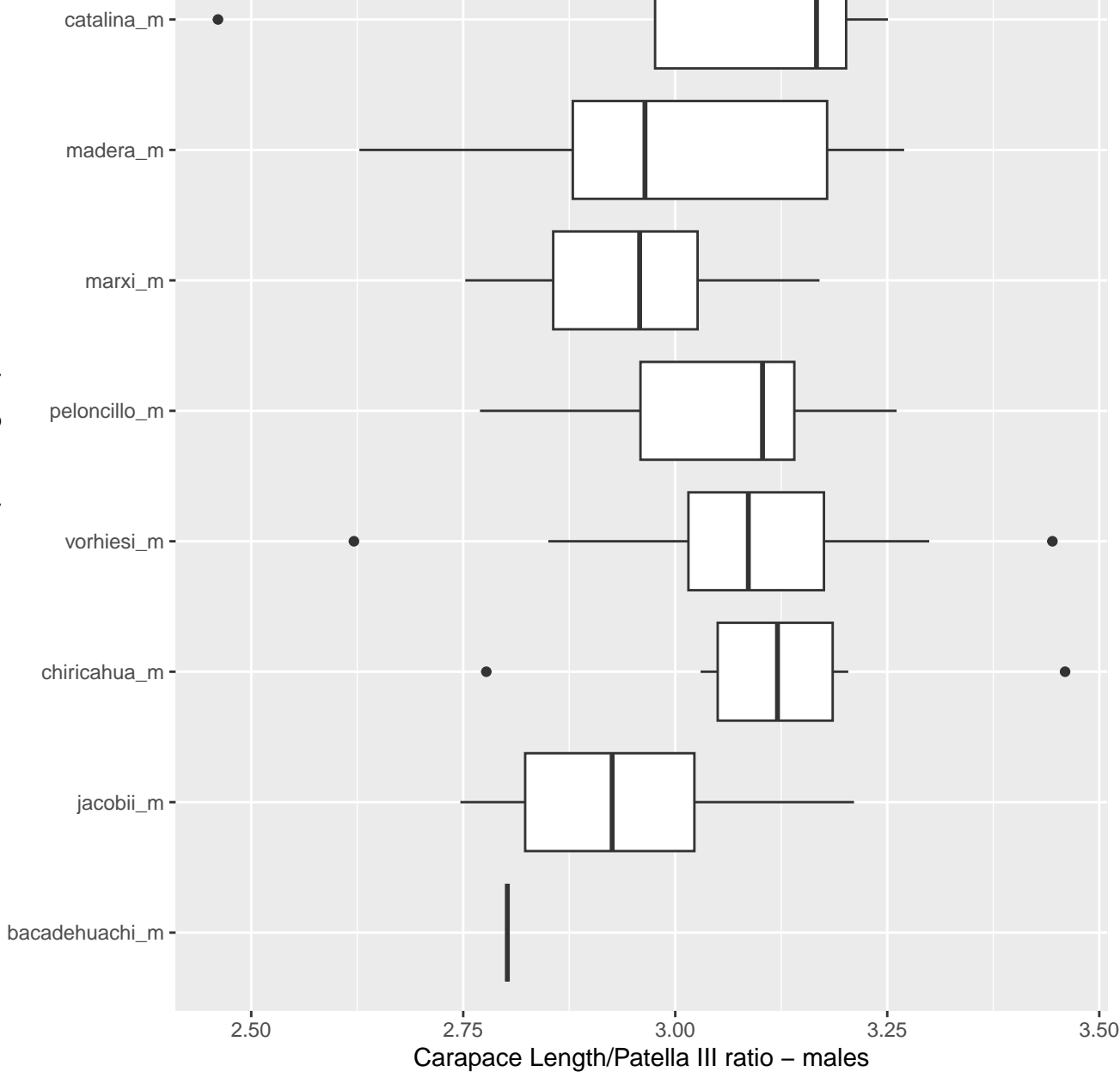

Supplement: Supplementary material 5 — Boxplots comparing various morphometric ratios based on measurements of mature male and female members of the Marxi species group [file zookeys-1210-061_article-125318__-s005.zip › Suppl5/Marxi_group_males/PDFs/ClP3_ratio_males.pdf]

Marxi species group

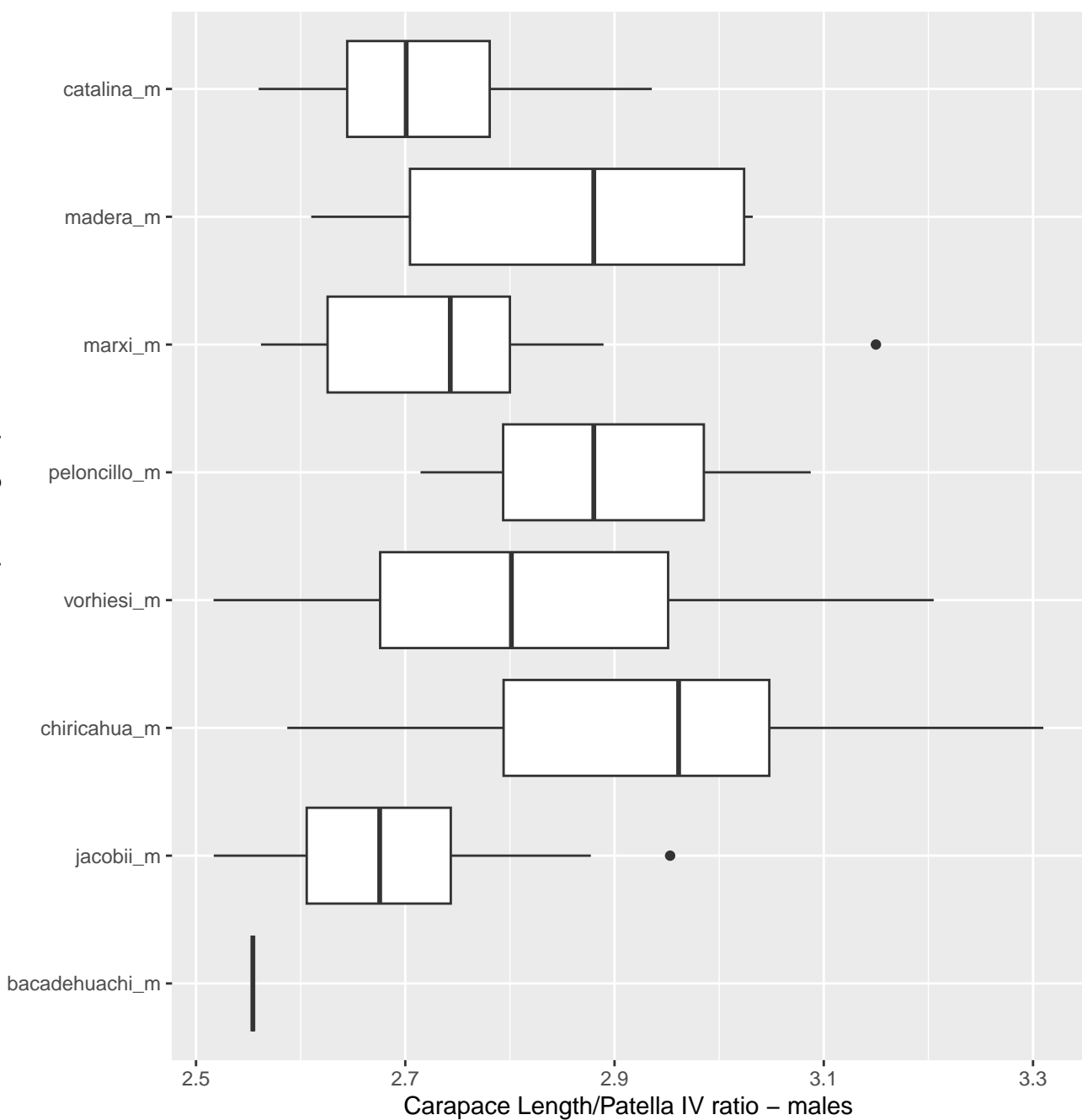

Supplement: Supplementary material 5 — Boxplots comparing various morphometric ratios based on measurements of mature male and female members of the Marxi species group [file zookeys-1210-061_article-125318__-s005.zip › Suppl5/Marxi_group_males/PDFs/ClP4_ratio_males.pdf]

Marxi species group

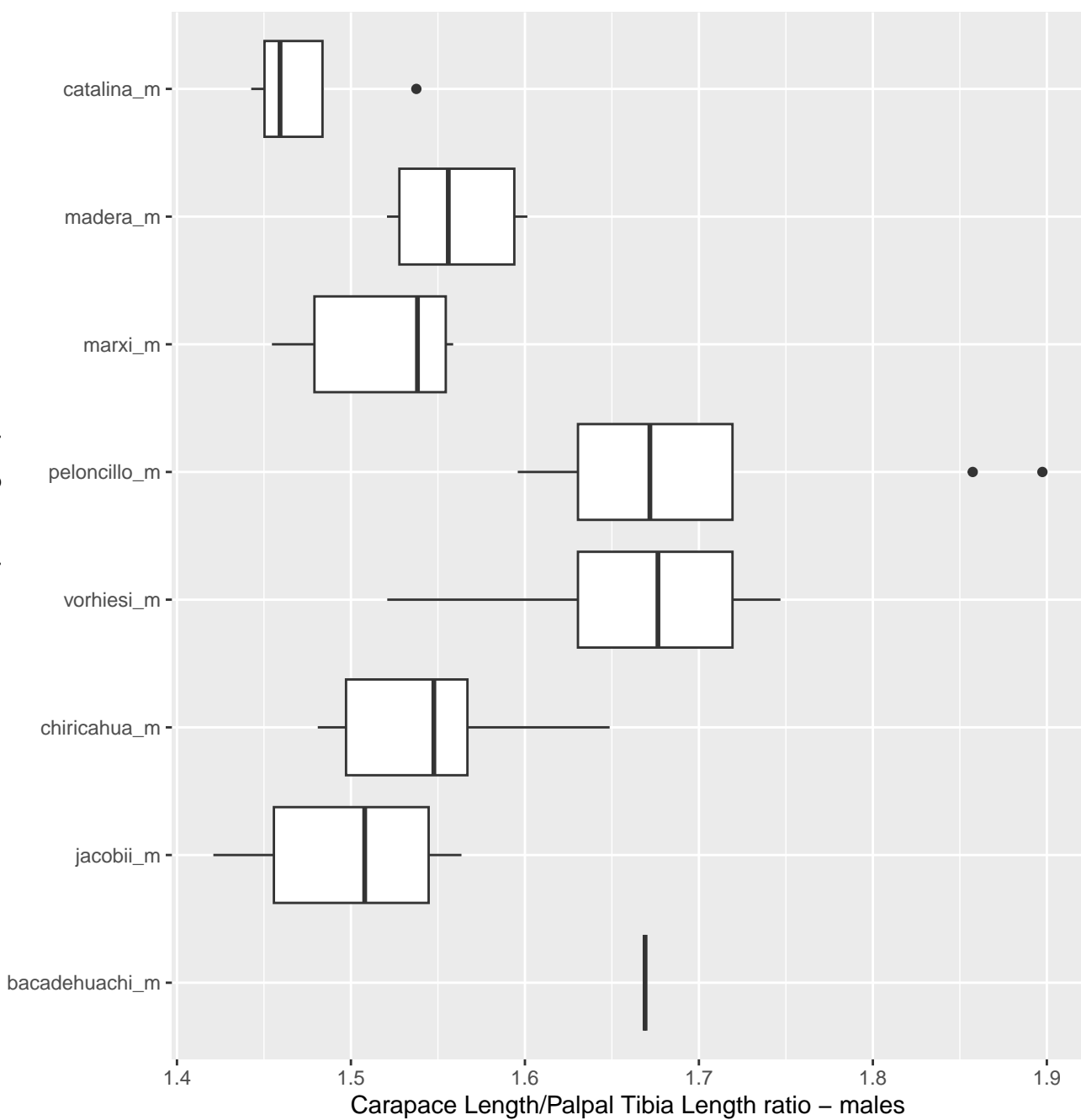

Supplement: Supplementary material 5 — Boxplots comparing various morphometric ratios based on measurements of mature male and female members of the Marxi species group [file zookeys-1210-061_article-125318__-s005.zip › Suppl5/Marxi_group_males/PDFs/ClPTl_ratio_males.pdf]

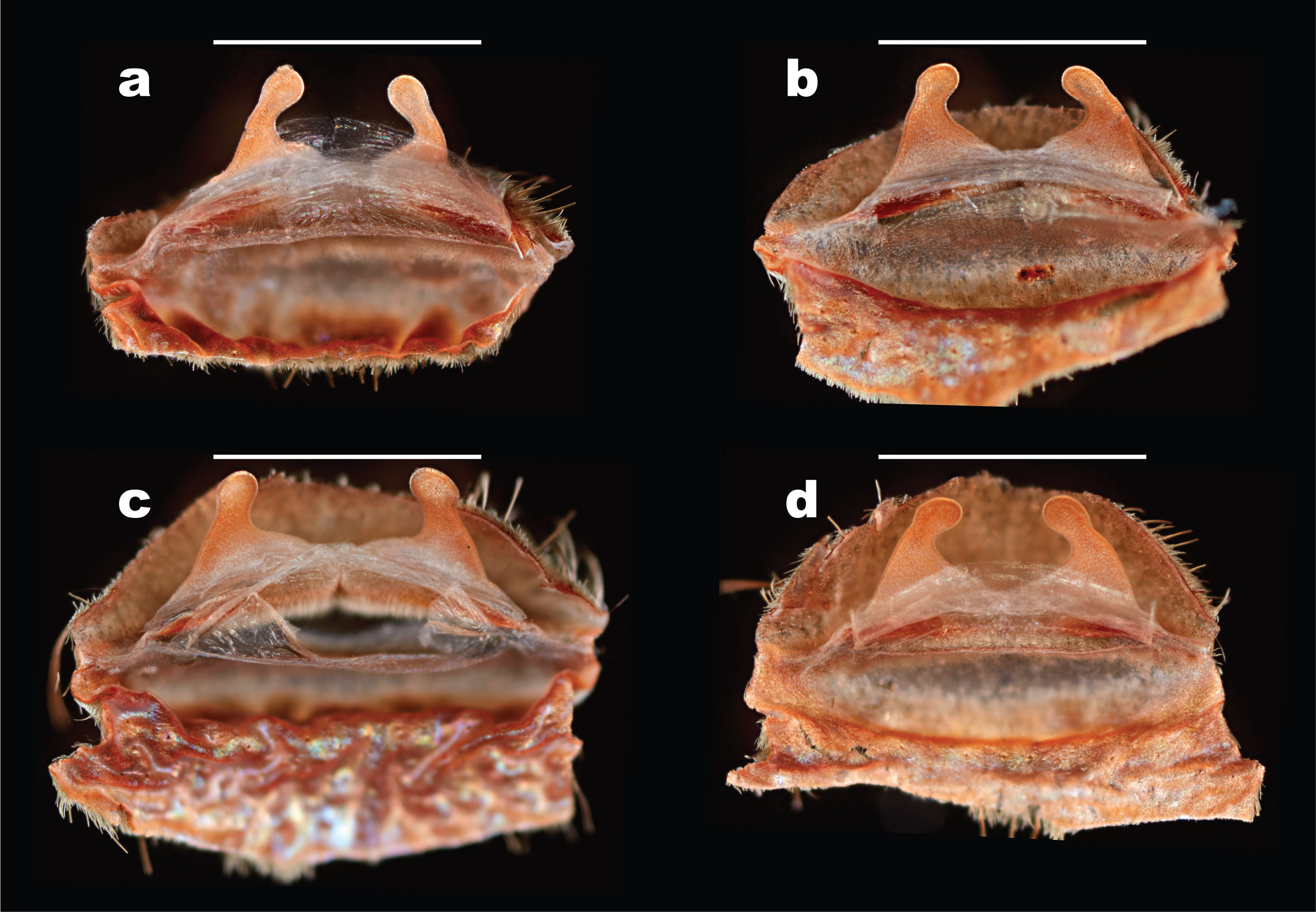

Supplement: Supplementary material 6 — Variation in spermathecae of female Aphonopelmajacobii sp. nov. [file zookeys-1210-061_article-125318__-s006.jpg]

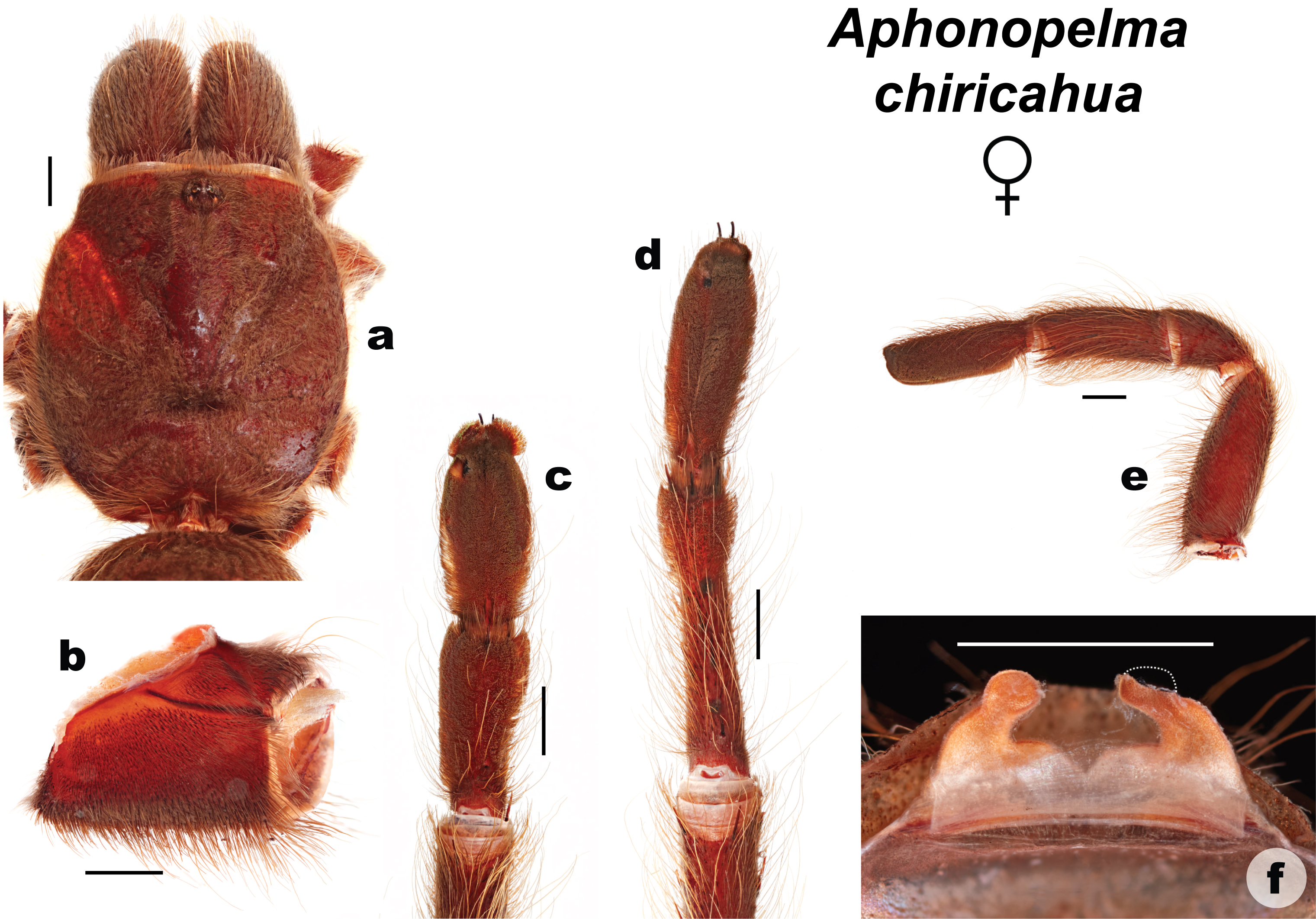

Supplement: Supplementary material 7 — Morphology of Aphonopelmachiricahua (female, APH-5050) [file zookeys-1210-061_article-125318__-s007.jpg]
