# Supplementary material for: ﻿Discovery of a new tarantula species from the Madrean Sky Islands and the first documented instance of syntopy between two montane endemics (Araneae, Theraphosidae, Aphonopelma): a case of prior mistaken identity
Source: Zookeys. 2024 Aug 16;1210:61–98. doi: 10.3897/zookeys.1210.125318 (PMC11344175; doi:10.3897/zookeys.1210.125318)
Supplement: Supplementary material 5 — Boxplots comparing various morphometric ratios based on measurements of mature male and female members of the Marxi species group [file zookeys-1210-061_article-125318__-s005.zip › Suppl5/Marxi_group_females/PDFs/P3A4_ratio_females.pdf]

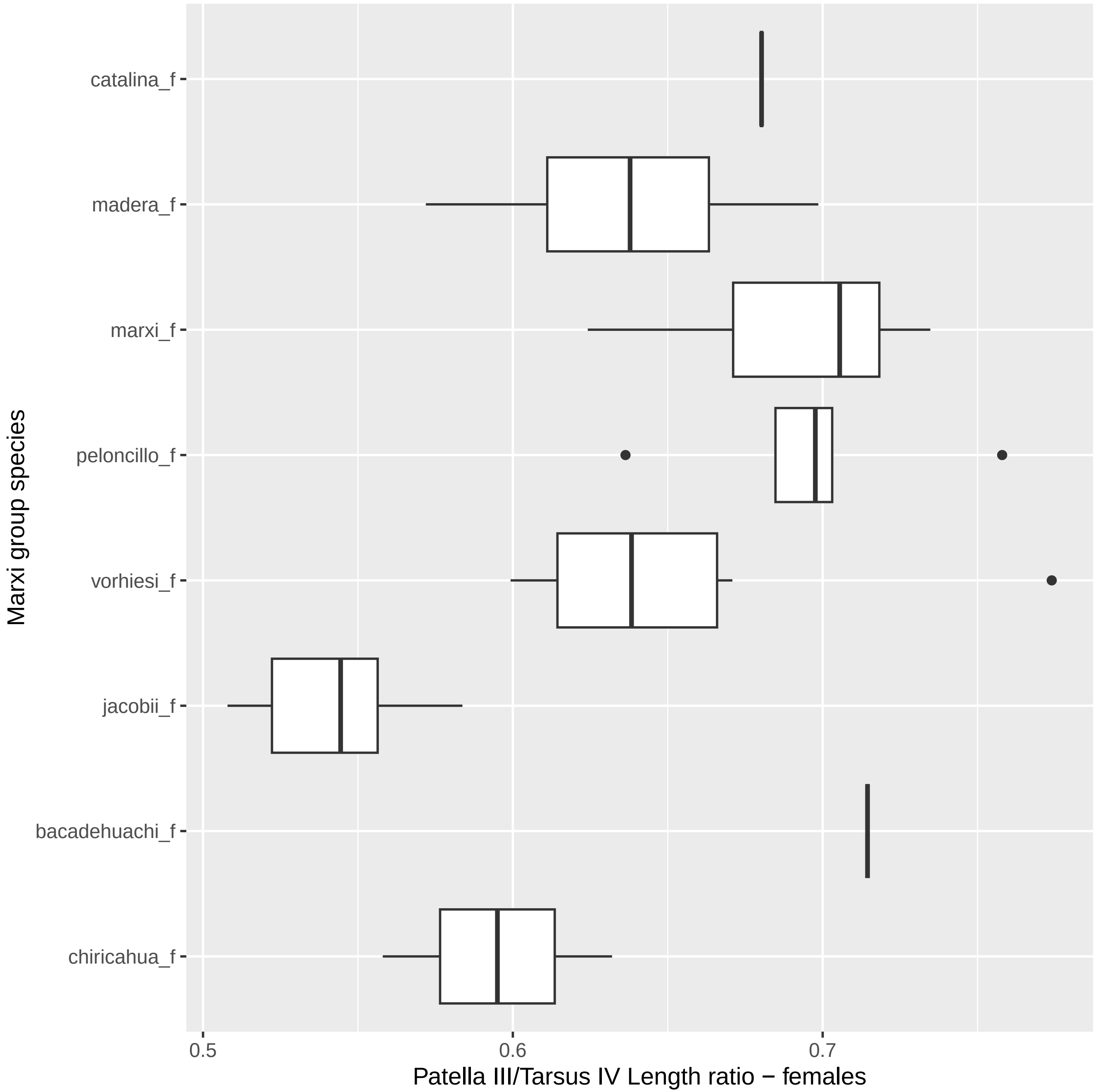

Marxi group species
catalina_f
madera_f
marxi_f
peloncillo_f
vorhiesi_f
jacobii_f
bacadehuachi_f
chiricahua_f
0.5
0.6
0.7
Patella III/Tarsus IV Length ratio − females
